# Electrochemical oxidation induced selective tyrosine bioconjugation for the modification of biomolecules†
†Electronic supplementary information (ESI) available. See DOI: 10.1039/c9sc02218j


**DOI:** 10.1039/c9sc02218j

**Published:** 2019-07-08

**Authors:** Chunlan Song, Kun Liu, Zhongjie Wang, Bo Ding, Shengchun Wang, Yue Weng, Chien-Wei Chiang, Aiwen Lei

**Affiliations:** a College of Chemistry and Molecular Sciences , Institute for Advanced Studies (IAS) , Wuhan University , Wuhan 430072 , P. R. China . Email: aiwenlei@whu.edu.cn; b National Synchrotron Radiation Research Center (NSRRC) , Hsinchu Science Park , Hsinchu , Taiwan; c National Research Center for Carbohydrate Synthesis , Jiangxi Normal University , Nanchang 330022 , P. R. China

## Abstract

Directly introducing a beneficial functional group into biomolecules under mild, clean and easy-to-handle conditions is of great importance in the field of chemical biology and pharmacology. Herein, we described an electrochemical strategy to perform the bioconjugation of tyrosine residues with phenothiazine derivatives in a rapid and simple manner. In this electrochemical system, various polypeptides and proteins were successfully labelled with excellent site- and chemo-selectivity, and metals, oxidants or additives were also avoided.

## Abstract 2

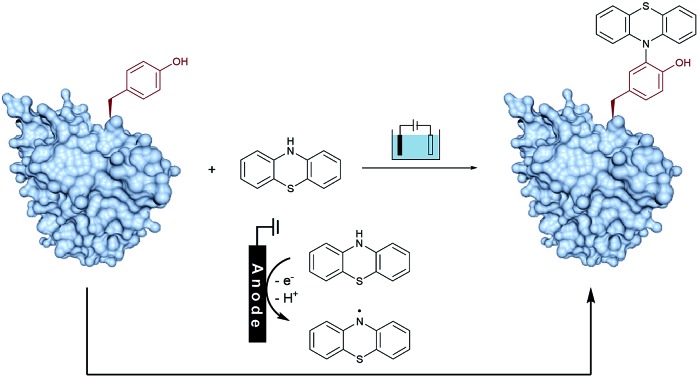
This electrochemical bioconjugation reaction provides an efficient modification of biomolecules with high chemo- and site-selectivity under mild conditions.

## Introduction

Developing a site-selective, mild and biocompatible reaction for biomolecule modification is an important pursuit in the field of chemical biology, medical chemistry and clinical pharmacology.^1^ Through the attachment of synthetic molecules to a specific position on proteins, bioconjugation can be applied to DNA labeling, antibody-drug modification and protein visualization studies.^2^ The advantage of naturally low abundance of aromatic amino acid residues on protein surfaces would lead to a higher degree of bioconjugation, without changing the overall charge state or redox sensitivity.^3^ However, due to the similarity of their redox potentials and the difficulty of C(sp^2^)–H functionalization,^4^ techniques for labeling these aromatic amino acid residues were fewer than those of cysteine and lysine.4b,c,^5^ Thus, aromatic amino acid residues still lack efficiency as well as chemo- and site-selective methods to achieve bioconjugation reactions.

As one of the important amino acids, tyrosine can be found in many polypeptides and proteins, such as tyrosine protein kinases, kisspeptin and myoglobin. Because of its low natural abundance in native proteins, tyrosine is also considered as an attractive target for labeling biomolecules.4d,^6^ Previously, some approaches have been developed for tyrosine modification, such as Mannich-type reactions,^7^ Pd catalysts,6a,^8^ Ru photocatalysts,^9^ click-like reactions,^10^*etc.*^11^ Recently, several strategies for site-selective protein modification based on oxidative coupling have been reported, which could significantly enrich the methodologies of bioconjugation chemistry.6c,^12^ Accompanied by a good momentum of organic electrosynthesis in the field of oxidative coupling reactions, electrochemical anodic oxidation provides a green option to prevent the usage of hazardous oxidants and harsh conditions, and sensitive functional groups could also be well tolerated.^13^ Merging electrochemical organic synthesis and bioconjugation chemistry is very promising, and therefore developing multifarious protocols for peptide and protein modification is highly desirable, especially when excellent selectivity and efficiency could be obtained.

**Fig. 1:**
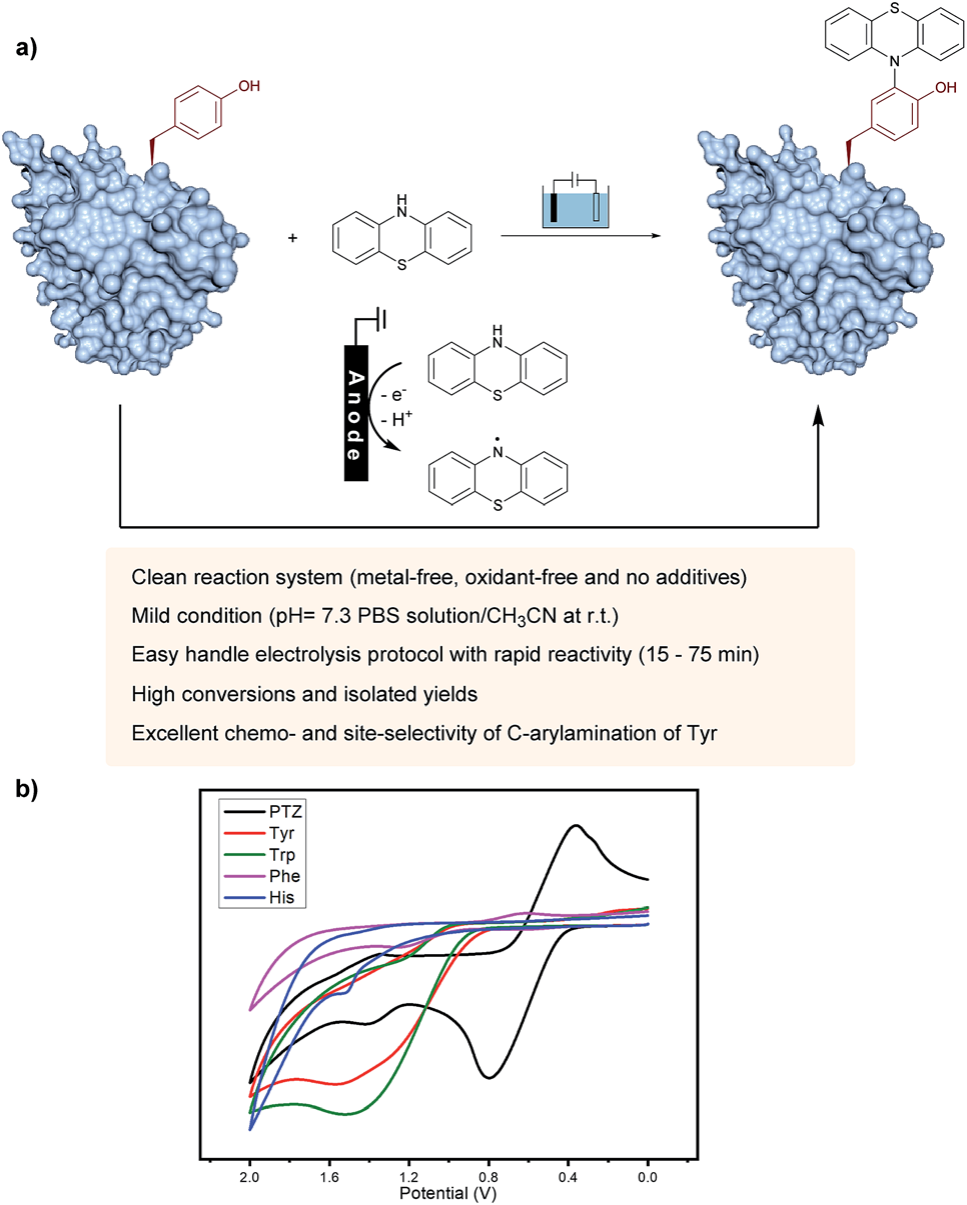
Discovery of an electrochemical oxidative tyrosine bioconjugation. (a) Proposed pathway: phenothiazine underwent single-electron oxidization by the anode to generate a radical species, and then coupled with the tyrosine residue of biomolecules to obtain the labeling product. (b) Cyclic voltammograms of 0.005 M PTZ (black), Tyr (red), Trp (green), Phe (purple) and His (blue) at a glassy carbon electrode, in 0.05 M ^*n*^Bu_4_NBF_4_ in CH_3_CN/H_2_O. Scan rate = 100 mV s^–1^. Reference electrode: Ag/AgCl.

On the other hand, as valuable labeling targets, phenothiazine derivatives have been recognized as highly bioactive drugs and chromophores.^14^ The incorporation of phenothiazine into biomolecules would not only demonstrate the advantage of phenothiazines-containing protein drugs, but also potentially lead to chemical probes. Recently, the group of Gouin has demonstrated the electrochemically promoted tyrosine-click-chemistry for protein labeling with a urazole reagent, which significantly improved both the yield and selectivity compared with the original conditions by using a pre-activated reagent.10*e* Herein, we described an electrochemically promoted labeling strategy of tyrosine-containing biomolecules with phenothiazine derivatives under simple, mild and clean conditions (Fig. 1a). Initially, in order to gain insight into the relative redox activity of phenothiazine and aromatic amino acids, cyclic voltammetry experiments have been performed (Fig. 1b). The results showed that the oxidation potential of phenothiazine was much lower than that of Tyr, Trp, Phe and His, which indicated that the reaction proceeded through the single-electron oxidation of phenothiazine (PTZ) to generate the nitrogen radical. Following radical addition to the *ortho*-position of phenol would achieve the modification of tyrosine. Due to the less electron-rich position of other aromatic amino acid residues, the radical addition would not be favored. Thus, through choosing phenothiazine as a valuable ‘tag’ to generate an active species, excellent site- and chemo-selectivity on the protein modification could be obtained.

## Results and discussion

To establish suitable conditions for this electro-oxidative bioconjugation reaction, a protected tyrosine **1a** and phenothiazine **2a** were chosen as the model reaction substrates for optimization. Under the electrolysis conditions, the reaction was conducted in a three-necked undivided cell with a graphite rod anode and a nickel plate cathode at 10 mA constant current for 75 minutes. Product **3a** could be obtained in 85% isolated yield with Na_2_SO_4_ as the electrolyte in CH_3_CN/H_2_O at room temperature (Table 1). To extend the scope of phenothiazine derivatives, a variety of substituted phenothiazines were then applied to couple with tyrosine **1a** under the optimized reaction conditions, which bear halogen (**3b**), sulfide (**3c**), trifluoromethyl (**3d**), acetyl (**3e**), cyano (**3f**) and azide (**3g**) groups. Under these reaction conditions, different electron-donating or electron-withdrawing substituted phenothiazines were all found to be suitable substrates in moderate isolated yields. Remarkably, this method could be extended to azide group substituted phenothiazine which could be applied in further bio-orthogonal reactions.

**Table 1:** Substrate scope of phenothiazines for electro-oxidative bioconjugation^*a*^

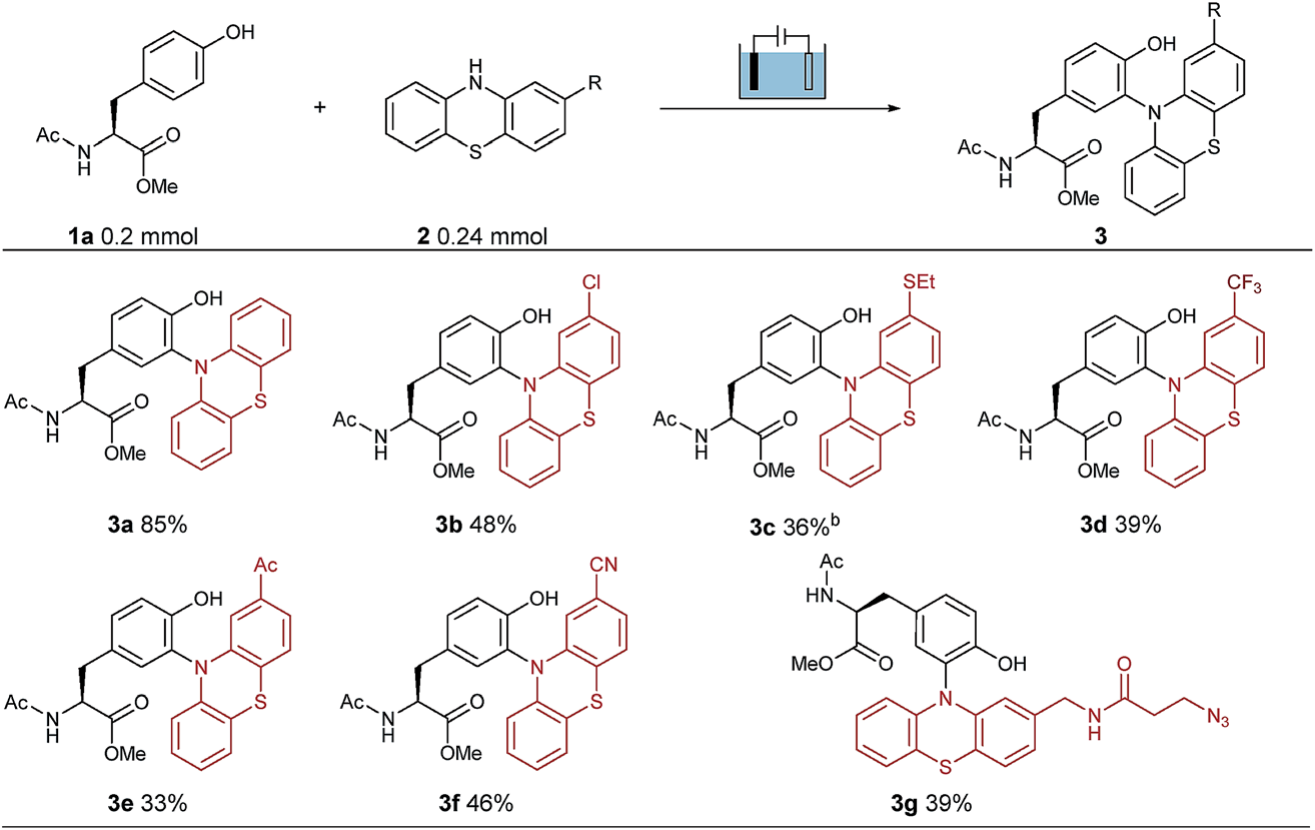

^*a*^Reaction conditions: graphite rod anode, nickel plate cathode, constant current = 10 mA, **1a** (1.0 equiv., 0.20 mmol), **2** (1.2 equiv., 0.24 mmol), Na_2_SO_4_ (2 equiv., 0.40 mmol), CH_3_CN/H_2_O (6.0 mL/4.0 mL), room temperature, N_2_, and 75 min (*Q* = 45 C, 2.3 F). Yields of isolated products are shown.

^*b*^10 mL CH_3_CN.

Furthermore, to explore the polypeptide selectivity and tolerance of this tyrosine labeling reaction, various dipeptides containing tyrosine were introduced into this system. With phenothiazine as the coupling partner, these dipeptides made a contribution to the desired bioconjugation reaction in CH_3_CN/PBS (pH = 7.3) solution without addition of Na_2_SO_4_ (Table 2). Besides the relatively inert glycine (**4a**) and leucine (**4b**), this established electrosynthesis protocol was also compatible with the thioether of methionine (**4c**), phenyl group of phenylalanine (**4d**), indole NH of tryptophan (**4e**), imidazole NH of histidine (**4f**), amino group of lysine (**4g**), hydroxyl group of serine (**4h**) and carboxylic acid of aspartic acid (**4i**), indicating a broad functional group tolerance. Meanwhile, the labeling was observed neither on the other aromatic amino acids Trp, His and Phe, nor on the amino, hydroxyl and carboxylic groups of Lys, Ser and Asp. Unfortunately, the Cys-containing dipeptide could only obtain trace amounts of the corresponding product, possibly due to the easily oxidizable property of cysteine under oxidation conditions, leading to the decomposition of the dipeptide. In most cases studied, this electrochemical bioconjugation reaction proceeded perfectly, selectively and cleanly with good isolated yields.

**Table 2:** Survey of amino acid selectivity for electro-oxidative bioconjugation^*a*^

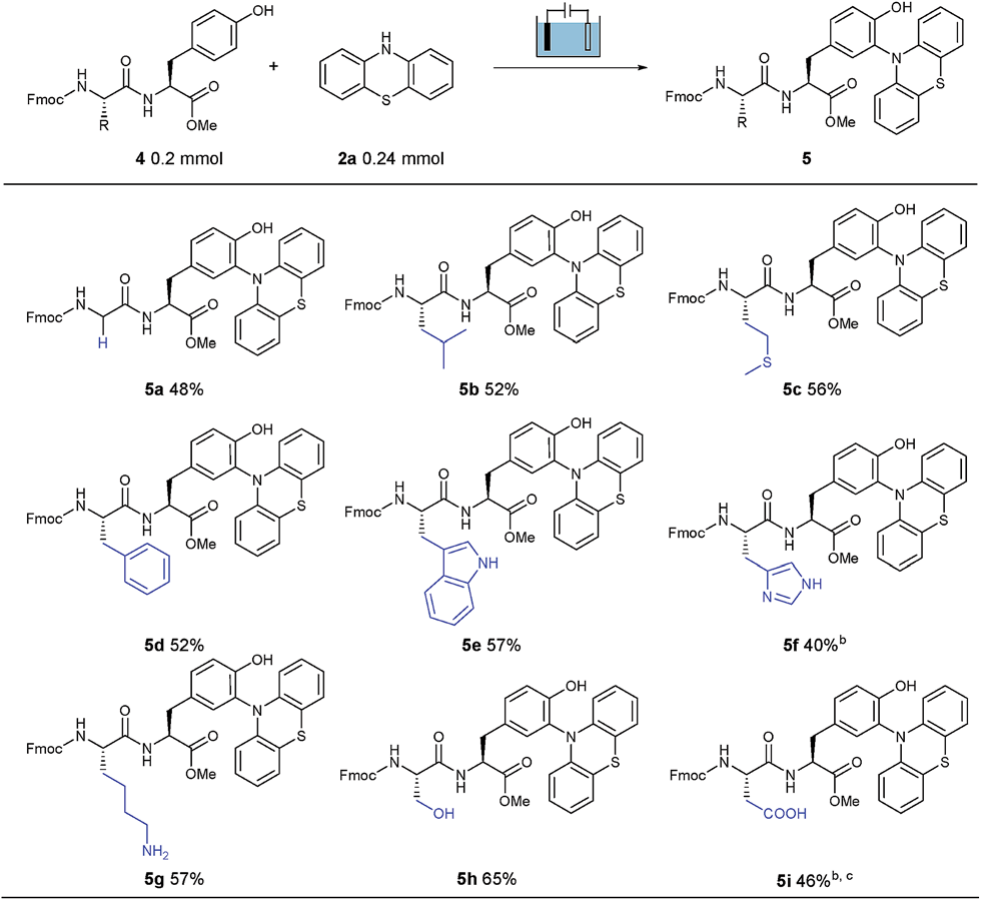

^*a*^Reaction conditions: graphite rod anode, nickel plate cathode, constant current = 10 mA, **4** (1.0 equiv., 0.20 mmol), **2a** (1.2 equiv., 0.24 mmol), CH_3_CN/PBS (pH = 7.3) (6.0 mL/4.0 mL), room temperature, N_2_, and 75 min (*Q* = 45 C, 2.3 F). Yields of isolated products are shown.

^*b*^105 min.

^*c*^CH_3_CN/PBS (pH = 8.0).

To open an electrosynthetic pathway towards biomolecules such as larger peptides and proteins, we next evaluated the applicability of this electro-oxidative bioconjugation procedure in the synthesis of phenothiazine-modified polypeptides. As a proof-of-concept, the modification of a variety of unprotected peptides **6** from 5-mers to 29-mers have been achieved effectively with excellent conversions (Table 3). We chose the Tyr-containing RGD peptide as the substrate originally, which was considered as an attractive target for tumor treatment by inducing apoptosis and caspase-3 activation. This extracellular RGD peptide was used in CH_3_CN/PBS (pH = 7.3) to furnish phenothiazine-conjugate **7a** in 99% conversion. Having established the electrochemical transformation of phenothiazine **2a** with RGD, we then turned our attention to other Tyr-containing polypeptides. For example, acyclic pentapeptide YAGFL was reacted with phenothiazine **2a** under electrolysis conditions, and the bioconjugated product **7b** was obtained in full conversion. We next examined the applicability of this electrolysis methodology for endogenous peptides with biological activity, such as angiotensin Y, kisspeptin 10, LH-RH, tyrosine protein kinases, MOG 35–55 and glucagon. The above peptides contained at least one tyrosine at the positions of the N-terminus, the C-terminus, or a loop. To our delight, all these polypeptides (**6c–6h**) were successfully tagged with phenothiazine within 30 min at room temperature. It was worth noting that if one equiv. of phenothiazine was added to the reaction solution of **6g** and **6h**, respectively, two different results could be observed. Since the two tyrosine residues of **6g** are located in different areas of the peptide, the modification could occur on the both tyrosine residues. For **6h**, two tyrosine residues are just located on the *i* and *i* + 3 positions, and the steric influence would make phenothiazine specifically labelled on a less steric hindrance position of Y10. Eventually, the modification of 29-mer peptide glucagon **6h** was conducted the single modified peptide **7h**. Meanwhile, a peptide DSIP (WAGGDASGE) **6i** which lacks tyrosine residue was used as a control experiment substrate to confirm the selectivity of the reaction. No desired bioconjugated product could be observed, demonstrating that this protocol had the ability to specifically label tyrosine residues on biomolecules with good site- and chemo-selectivity.

**Table 3:** Scope of polypeptides for the electro-oxidative bioconjugation^*a*^

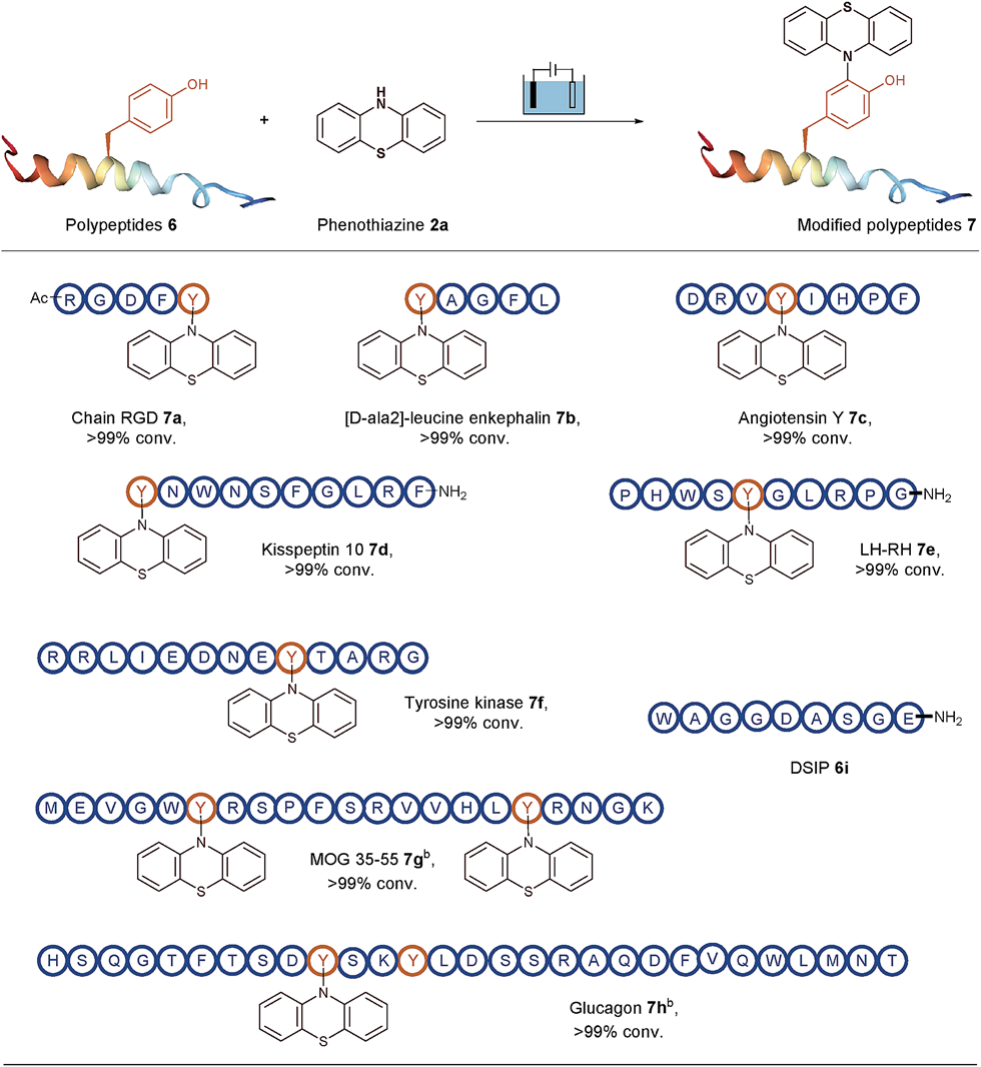

^*a*^Reaction conditions: graphite rod anode, nickel plate cathode, constant current = 10 mA, polypeptides **6** (5 mg), phenothiazine **2a** (10 mg), CH_3_CN/PBS (pH = 7.3) (0.75 mL/0.75 mL), room temperature, N_2_, and 30 min. Conversion of products are shown, as determined by HPLC.

^*b*^One equiv. of phenothiazine was used.

**Fig. 2:**
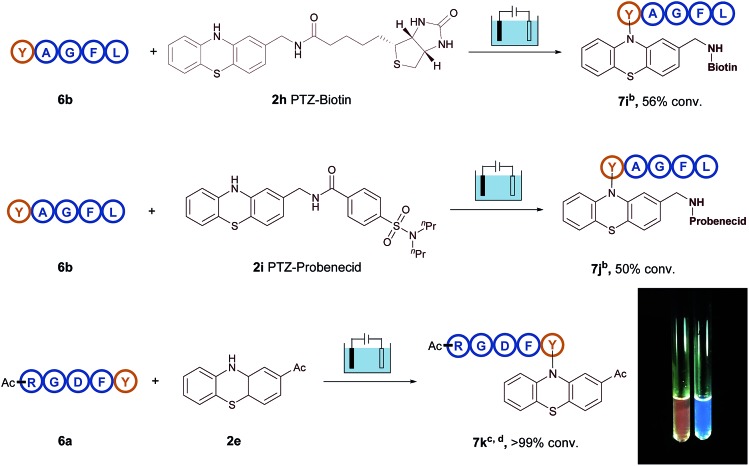
Additional application of electrochemical oxidative bioconjugation. ^a^Reaction conditions: graphite rod anode, nickel plate cathode, constant current = 10 mA, polypeptides (5 mg), phenothiazine (10 mg), CH_3_CN/PBS (pH = 7.3) (0.75 mL/0.75 mL), room temperature, N_2_, and 30 min. ^b^Conversion yields of products are shown, as determined by HPLC using diphenylamine as the standard. ^c^Conversion yields of products are shown, as determined by HPLC using anisic acid as the standard. ^d^Photographs of the solution of **6a** (right) and modified product **7k** (left) with UV lamp excitation.

Therefore, all these examples revealed the exceptional site- and chemo-selectivity of the reaction and its application to the peptide-labelled chemistry. In comparison with reported bioconjugation methods, this electrolysis method provided fast kinetics and high productivity in a metal- and additive-free manner. It is particularly noteworthy that this protocol provides a direct access to late-stage derivatization of valuable drugs (Fig. 2). For instance, when the biotin-containing phenothiazine **2h** and probenecid-containing (uricosuric drugs) phenothiazine **2i** were introduced to react with pentapeptide YAGFL **6b**, respectively, the desired products **7i** and **7j** could be obtained. Noticeably, these reactions were permitted by the direct electro-oxidative bioconjugation reaction, and exhibit a remarkable substituent tolerance. In addition, to demonstrate that the phenothiazine-labelled peptide might be utilized as a fluorophore, molecule **7k** from the reaction of 2-acetylphenothiazine **2e** and RGDFY **6a** was prepared. The photograph showed that phenothiazine-modified peptide **7k** apparently presented the ability of fluorescence emission under irradiation of UV light, clearly indicating that phenothiazine labelled biomolecules had the potential to be a possible biological fluorophore.

**Fig. 3:**
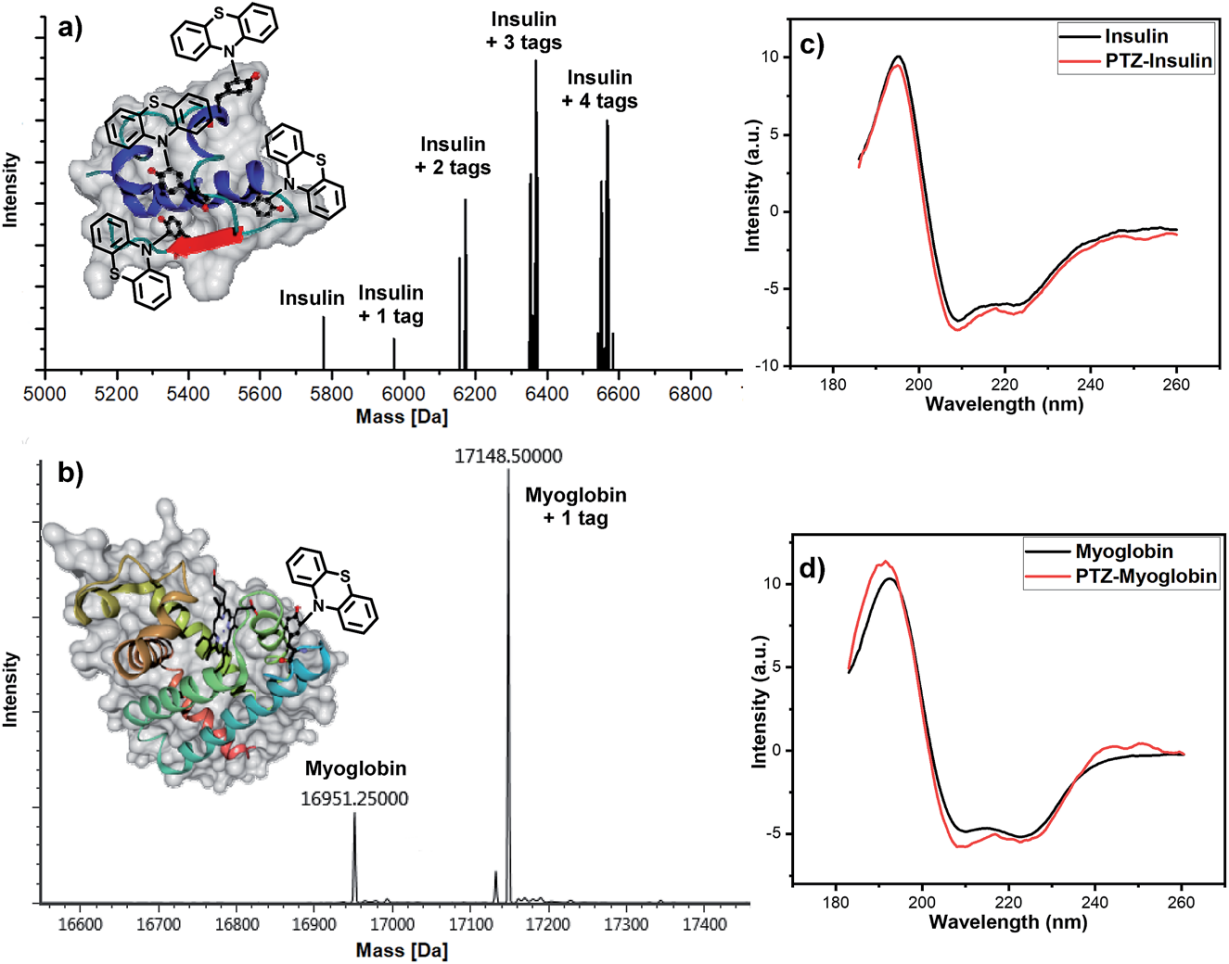
Electrochemical modification of proteins. Reaction conditions for the bioconjugation of insulin: graphite rod anode, nickel plate cathode, constant current = 10 mA, insulin (5 mg), phenothiazine (10 mg), CH_3_CN/PBS (pH = 7.3) (0.75 mL/0.75 mL), room temperature, N_2_, and 30 min. Reaction conditions for the bioconjugation of myoglobin: graphite rod anode, nickel plate cathode, constant current = 0.5 mA, myoglobin (5 mg), phenothiazine (20 mg), CH_3_CN/PBS (pH = 7.3) (0.75 mL/0.75 mL), –20 °C, N_2_, and 15 min. (a) Maldi-Tof MS analysis of modified insulin. (b) LC-MS analysis of modified myoglobin. (c) Effect of electrolytic arylamination on the structure of insulin. (d) Effect of electrolytic arylamination on the structure of myoglobin.

Afterward, we turned to discover the modification of proteins. Two proteins, insulin and myoglobin, containing at least one exposed tyrosine on the protein surface were electrolyzed with phenothiazine. When an excess amount of phenothiazine was added to the solution of insulin under electrolysis, four potentially reactive tyrosine residues on insulin were all tagged (Fig. 3a). Next, we tested the feasibility of our developed method for a larger protein, myoglobin. The electro-oxidative bioconjugation could be well performed within 15 min and generate single phenothiazine tagged myoglobin at –20 °C (Fig. 3b). Importantly, CD spectroscopy revealed that the electrochemical methodology had negligible influence on the structure of both insulin and myoglobin (Fig. 3c and d). These satisfactory results revealed that the exposed tyrosine could be well-tagged by phenothiazine under our electro-oxidative conditions without decomposition.

## Conclusions

By introducing organic electrochemistry, tyrosine residues can be well-labelled with phenothiazine derivatives with high chemo- and site-selectivity as well as excellent conversion under mild conditions. This strategy has been successfully applied to the modification of valuable biomolecules such as polypeptides, insulin and myoglobin. Moreover, this technique could promote the development of safer and more biological sustainable bioconjugation reactions that are operated under metal-, oxidant- and additive free conditions. We anticipate that advances in electrochemically induced bioconjugation will lead to an expanding library of interdisciplinary methodologies.

## Experimental

A general procedure for bioconjugation of tyrosine and phenothiazine derivatives: in an oven-dried undivided three-necked bottle (25 mL) equipped with a stir bar, protected tyrosine (0.20 mmol), phenothiazine (0.24 mmol), Na_2_SO_4_ (0.40 mmol) and CH_3_CN/H_2_O (6 mL/4 mL) were combined and added. The bottle was equipped with a graphite rod (*φ* 6 mm, about 15 mm immersion depth in solution) as the anode and a nickel plate (15 mm × 15 mm × 1.0 mm) as the cathode and then charged with nitrogen. The reaction mixture was stirred and electrolyzed at a constant current of 10 mA under room temperature for 75 min (2.3 F). When the reaction was finished, the reaction mixture was extracted with ethyl acetate (10 mL × 3). The organic layers were combined, dried over Na_2_SO_4_, and concentrated. The pure product was obtained by flash column chromatography on silica gel (dichloromethane : methanol = 100 : 1).

## Conflicts of interest

There are no conflicts to declare.

## Supplementary Material

Supplementary information
