# Supplementary material for: Electrochemical oxidation induced selective tyrosine bioconjugation for the modification of biomolecules
Source: Chem Sci. 2019 Jul 8;10(34):7982–7. doi: 10.1039/c9sc02218j (PMC6788519; doi:10.1039/c9sc02218j)

Electronic Supplementary Material (ESI) for Chemical Science.
This journal is © The Royal Society of Chemistry 2019

Electrochemical Oxidation Induced Selective Tyrosine Bioconjugation for the Modification of Biomolecules

Chunlan Song,[1] Kun Liu,[1] Zhongjie Wang,[1] Bo Ding,[1] Shengchun Wang, [1] Yue Weng,[*1,2] Chien-Wei Chiang,[*1] Aiwen Lei[*1,3]

[1] College of Chemistry and Molecular Sciences, the Institute for Advanced Studies (IAS), Wuhan University, Wuhan, Hubei 430072, People's Republic of China

[2] National Synchrotron Radiation Research Center (NSRRC), Hsinchu Science Park, Hsinchu, Taiwan

[3] National Research Center for Carbohydrate Synthesis Jiangxi Normal University, Nanchang 330022, Jiangxi, People's Republic of China

***corresponding author: E-mail: aiwenlei@whu.edu.cn; cwchiang@whu.edu.cn; wengyue@whu.edu.cn**

Supporting Information

TABLE OF CONTENTS

1. General Information 3
2. Synthesis of Starting Materials 4
3. General Procedure 14
3.1 Reaction optimization 14
3.2 General procedure for cyclic voltammetry 15
3.3 General procedure for electron paramagnetic resonance (EPR) 15
3.4 Proposed mechanism 16
3.5 Phenothiazine scope and characterization 17
3.6 Dipeptide scope and characterization 21
3.7 Polypeptide scope and characterization 27
3.8 Protein scope and characterization 51
4. References 57
5. Spectra 58
5.1 NMR spectra of products 58
5.2 UV-VIS absorption spectra of reactants and products 75
5.3 Fluorescence measurement of reactants and products 85

1. General Information

All glassware was oven dried at 110 °C for hours and cooled down under vacuum. Unless otherwise noted, materials were obtained from commercial suppliers and used without further purification. The instrument for electrolysis was dual display potentiostat (DJS-292B) (made in China). The anodic electrode was graphite rod (ϕ 6 mm) and cathodic electrode was nickel plate (15 mm×15 mm×1.0 mm). Thin layer chromatography (TLC) employed glass 0.25 mm silica gel plates. Flash chromatography columns were packed with 200-300 mesh silica gel. Gradient flash chromatography was conducted eluting with a continuous gradient from dichloromethane to the methanol. High resolution mass spectra (HRMS) for dipeptides were measured with a Waters Micromass GCT instrument and accurate masses were reported for the molecular ion + Sodium (M+Na). High resolution mass spectra (HRMS) for polypeptides were measured with an ABI 5800 instrument and accurate masses were reported for the molecular ion + Hydrogen (M+H) or molecular ion + Sodium (M+Na). The ^{1}H, ^{13}C and ^{19}F NMR spectra were recorded on a Bruker 400 MHz NMR spectrometer. For ^{1}H NMR, chemical shifts (δ) were given in ppm relatives to internal standard (TMS at 0 ppm, DMSO-d_6 at 2.50 ppm, MeOH-d_4 at 3.31 ppm, Acetone-d_6 at 2.05 ppm). For ^{13}C-NMR, chemical shifts (δ) were reported in ppm using solvent as internal standard ($CDCl_3$ at 77.00 ppm, DMSO-d_6 at 39.50 ppm, MeOH-d_4 at 49.00 ppm, Acetone-d_6 at 29.84 ppm). HPLC analyses were performed on an Agilent 1260 Infinity LC system using a 100 mm Agilent Zorbax 300SB-C18 5 μm analytical column. All of the MALDI-TOF-MS and MALDI-TOF-MS/MS spectra were acquired using 5800 MALDI-MS (AB SCIEX, Concord, Canada) equipped with a 355 nm Nd: YAG laser in the reflector positive mode. Samples of 0.6 μ L mixed with 0.6 μ L freshly prepared CHCA matrix were directly loaded onto the stainless steel MALDI plate and allowed to dry in a gentle stream of warm air. Samples were ablated with a power of 3500 while the laser rastered over the target surface. A total of 2000 laser shots were employed in each sample spot. The MS and MS/MS data processing were further performed by DataExplorer 4.0 (AB SCIEX, Concord, Canada). Protein LC-MS

analysis performed by XevoG2-XS QTof (Waters) and UPLC (Acquity UPLC I-Class) (Waters). UV-vis absorption spectra were performed on a Shimadzu UV-2700 spectrophotometer or Agilent Technologies Cary 8454. Fluorescence spectra were collected on a Hitachi F-4600 fluorescence spectrophotometer. The circular dichroism spectra were collected on Chhirascan™ CD spectroscopy (Applied Photophysics, Leatherhead, United Kingdom). CD spectra were collected from 180 nm to 280 nm and with a scanning speed of 200 nm/min. The bandwidth was 5 nm, and the response time was 2s. All spectra were taken at ambient temperature.

2. Synthesis of Starting Materials

2.1 Synthesis of protected amino acid 1a[1]

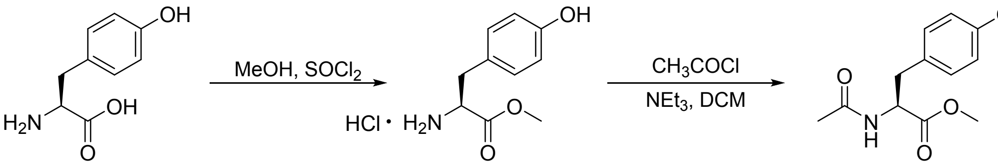

1a

In an oven-dried round-bottom flask (100mL), tyrosine (20 mmol) was dissolved in anhydrous MeOH (40 mL) at 0 °C. Carefully added (2.8 mL, 40 mmol) of $SOCl_2$ dropwise. The reaction mixture was then warmed to room temperature and stirred overnight. The solvent was removed by rotary evaporation to afford amino ester hydrochloride residue. Without further purification, the residue and triethylamine (5.6 mL, 40 mmol) was added in anhydrous DCM (40 mL) and stirred for 15 min at 0 °C. Acetyl chloride (1.4 mL, 20 mmol) was added to the reaction solution dropwise. Stirring was continued for 12 h while allowing the mixture to warm up to room temperature. The reaction was washed with saturated $NaHCO_3$ solutions (50 mL×2) and 10% HCl (50 mL×1) to remove any unreacted starting material. The combined organic extracts were dried ($MgSO_4$), filtered, and concentrated in vacuo. Purification of the residue by flash chromatography led to the desired protected amino acid **1a**.

2.2 Synthesis of starting materials dipeptides 4a-4d [2]

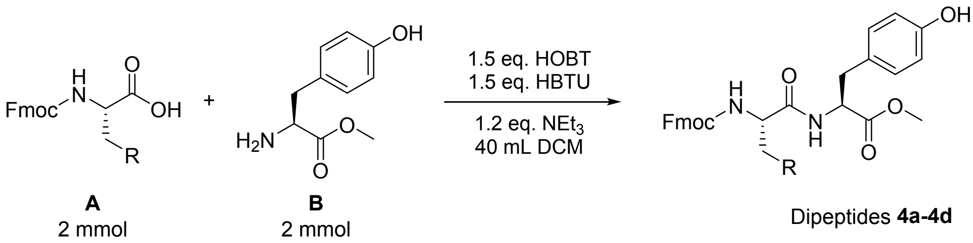

In a round bottomed flask, equipped with a stir bar, peptide **A** (2.0 mmol), HOBT (1-hydroxybenzotriazole) (3.0 mmol), HBTU (O-benzotriazole-*N, N, N', N'*-tetramethyl-uronium-hexafluorophosphate) (3.0 mmol), dichloromethane (40 mL) and triethylamine (2.4 mmol) were combined and added. The mixture was stirred for 30 min at room temperature, and then, peptide **B** (2.0 mmol) was added to the solution. The reaction was stirred overnight. After regular workup, the reaction mixture washed by saturated $NaHCO_3$ solution (40 mL x 3), 2M hydrochloric acid solution (40 mL x 3) and H_2O (40 mL x 3). The organic layers were combined, dried over Na_2SO_4, and concentrated. The resulting crude product was purified by flash chromatography (DCM/ MeOH) to afford corresponding dipeptides **4a-4d**.

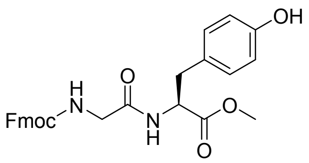

4a

Dipeptide **4a** **Fmoc-Gly-Tyr-OMe**, white solid. ^{1}H NMR (400 MHz, Acetone-d_6) δ 8.32 (s, 1H), 7.85 (d, J = 7.6 Hz, 2H), 7.72 (d, J = 7.6 Hz, 2H), 7.43 – 7.30 (m, 3H), 7.32 (t, J = 7.2 Hz, 2H), 7.03 (d, J = 8.4 Hz, 2H), 6.82 (t, J = 6.0 Hz, 1H), 6.75 (d, J = 8.0 Hz, 2H), 4.71 – 4.66 (m, 1H), 4.37 – 4.22 (m, 3H), 3.91 – 3.81 (m, 2H), 3.64 (s, 3H), 3.04 – 2.99 (m, 1H), 2.96 – 2.91 (m, 1H). ^{13}C NMR (101 MHz, Acetone-d_6) δ 172.53, 169.79, 157.45, 157.14, 144.97, 142.01, 131.14, 128.49, 128.05, 127.92, 126.13, 120.75, 116.03, 67.36, 54.60, 52.26, 47.88, 44.66, 37.47.

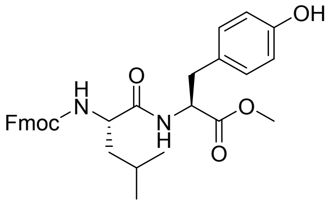

4b

Dipeptide **4b** **Fmoc-Leu-Tyr-OMe**, white solid. ^{1}H NMR (400 MHz, Acetone-d_6) δ

8.37 (s, 1H), 7.84 (d, J = 7.6 Hz, 2H), 7.72 – 7.68 (m, 2H), 7.63 – 7.58 (m, 1H), 7.39 (t, J = 7.6 Hz, 2H), 7.30 (td, J = 7.6, 1.2 Hz, 2H), 7.02 (d, J = 8.4 Hz, 2H), 6.84 – 6.80 (m, 1H), 6.75 (d, J = 8.2 Hz, 2H), 4.73 – 4.68 (m, 1H), 4.39 – 4.29 (m, 3H), 4.24 – 4.20 (m, 1H), 3.63 (s, 3H), 3.06 – 2.93 (m, 2H), 1.78 – 1.69 (m, 1H), 1.63 – 1.57 (m, 2H), 0.94 – 0.89 (m, 6H). ^{13}C NMR (101 MHz, Acetone-d_6) δ 173.18, 172.51, 157.06, 156.97, 145.02, 144.74, 141.95, 131.09, 128.43, 127.87, 126.08, 120.70, 115.96, 67.13, 54.64, 54.16, 52.21, 47.89, 41.91, 37.34, 25.25, 23.41, 21.91.

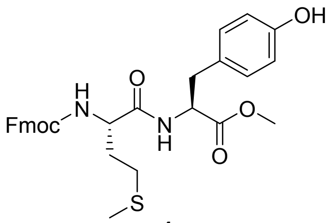

4c

Dipeptide **4c Fmoc-Met-Tyr-OMe**, white solid. ^{1}H NMR (400 MHz, Acetone-d_6) δ 8.32 (s, 1H), 7.85 (d, J = 7.6, 2H), 7.71 (t, J = 7.2 Hz, 2H), 7.52 (d, J = 7.6 Hz, 1H), 7.41 (t, J = 7.6 Hz, 2H), 7.34 – 7.30 (m, 2H), 7.05 – 6.02 (m, 2H), 6.80 (d, J = 8.4 Hz, 1H), 6.77 – 6.673 (m, 2H), 4.69 – 4.64 (m, 1H), 4.39 – 4.29 (m, 3H), 4.25 – 4.20 (m, 1H), 3.65 (s, 3H), 3.06 – 3.11 (m, 1H), 2.98 – 2.93 (m, 1H), 2.60 – 2.48 (m, 2H), 2.13 – 2.03 (m, 1H), 2.05 (s, 3H), 1.98 – 1.88 (m, 1H). ^{13}C NMR (101 MHz, Acetone-d_6) δ 172.55, 172.08, 157.13, 156.93, 145.07, 144.85, 142.03, 131.13, 128.50, 127.92, 126.14, 120.77, 116.02, 67.19, 54.82, 54.70, 52.28, 47.94, 37.26, 32.89, 30.61, 15.13.

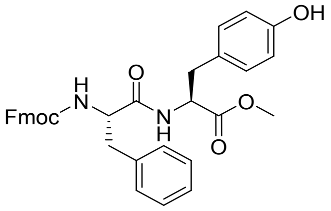

4d

Dipeptide **4d Fmoc-Phe-Tyr-OMe**, white solid. ^{1}H NMR (400 MHz, Acetone-d_6) δ 8.39 (s, 1H), 7.83 (d, J = 7.6 Hz, 2H), 7.64 – 7.62 (m, 3H), 7.39 (td, J = 7.6, 1.2 Hz, 2H), 7.31 – 7.15 (m, 7H), 7.04 – 7.00 (m, 2H), 6.81 (d, J = 8.8 Hz, 1H), 6.76 – 6.73 (m, 2H), 4.73 – 4.68 (m, 1H), 4.58 – 4.53 (m, 1H), 4.29 – 4.24 (m, 1H), 4.19 – 4.12 (m, 2H), 3.64 (s, 3H), 3.20 (dd, J = 14.0, 4.8 Hz, 1H), 3.07 – 2.90 (m, 3H). ^{13}C NMR (101 MHz, Acetone-d_6) δ 172.09, 171.71, 156.77, 156.41, 144.55, 144.47, 141.58, 138.21,

130.79, 129.85, 128.65, 128.09, 127.53, 126.85, 125.73, 120.35, 115.65, 66.87, 56.64, 54.42, 51.92, 47.44, 38.30, 37.07.

2.3 Synthesis of starting materials dipeptides 4e-4h[2]

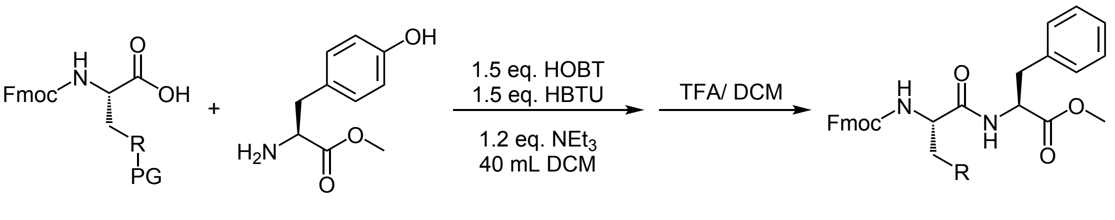

A 2 mmol **B** 2 mmol **Dipeptides 4e-4h**

In a round bottomed flask, equipped with a stir bar, peptide **A** (2.0 mmol), HOBT (1-hydroxybenzotriazole) (3.0 mmol), HBTU (O-benzotriazole-*N, N, N', N'*-tetramethyl-uronium-hexafluorophosphate) (3.0 mmol), dichloromethane (40 mL) and triethylamine (2.4 mmol) were combined and added. The mixture was stirred for 30 min at room temperature, and then, peptide **B** (2.0 mmol) was added to the solution. The reaction was stirred overnight. After regular workup, the reaction mixture washed by saturated $NaHCO_3$ solution (40 mL x 3), 2M hydrochloric acid solution (40 mL x 3) and H_2O (40 mL x 3). The organic layers were combined, dried over Na_2SO_4, and concentrated. Without further purification, the 95% TFA / DCM solution (8 mL) was added dropwise. The mixture was stirred for 2 h at room temperature. The resulting crude product was purified by flash chromatography (DCM / MeOH) to afford corresponding dipeptides **4e-4h**.

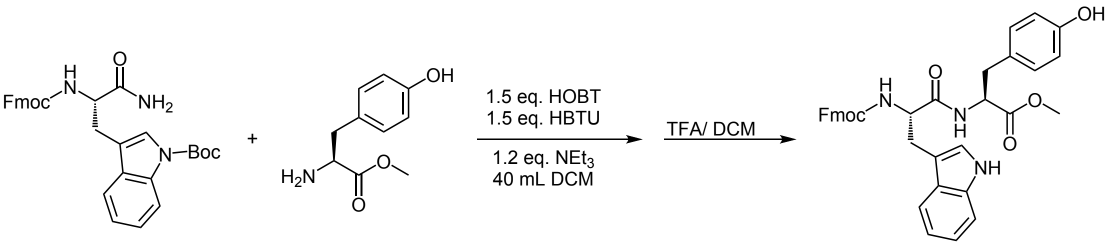

A 2 mmol **B** 2 mmol Dipeptide **4e**

Dipeptide **4e Fmoc-Trp-Tyr-OMe**, white solid. ^{1}H NMR (400 MHz, Acetone-d_6) δ 10.08 (s, 1H), 8.22 (s, 1H), 7.84 (d, *J* = 7.6 Hz, 2H), 7.68 – 7.61 (m, 3H), 7.42 – 7.37 (m, 4H), 7.30 – 7.26 (m, 2H), 7.22 (s, 1H), 7.09 (dd, *J* = 6.8, 1.2 Hz, 1H), 7.05 – 7.00 (m, 1H), 6.99 – 6.94 (m, 2H), 6.74 – 6.69 (m, 2H), 6.62 (d, *J* = 8.4 Hz, 1H), 4.69 – 4.64

(m, 1H), 4.57 – 4.54 (m, 1H), 4.34 – 4.10 (m, 3H), 3.63 (s, 3H), 3.32 – 3.27 (m, 1H), 3.18 – 3.13 (m, 1H), 3.02 – 2.99 (m, 1H), 2.94 – 2.87 (m, 1H). ^{13}C NMR (101 MHz, Acetone-d_6) δ 172.47, 172.16, 157.10, 156.79, 145.00, 144.95, 142.00, 137.55, 131.19, 128.46, 128.13, 127.94, 126.15, 124.55, 122.11, 120.73, 119.57, 119.34, 116.00, 112.15, 111.31, 67.22, 56.48, 54.75, 52.24, 47.92, 37.47, 28.77.

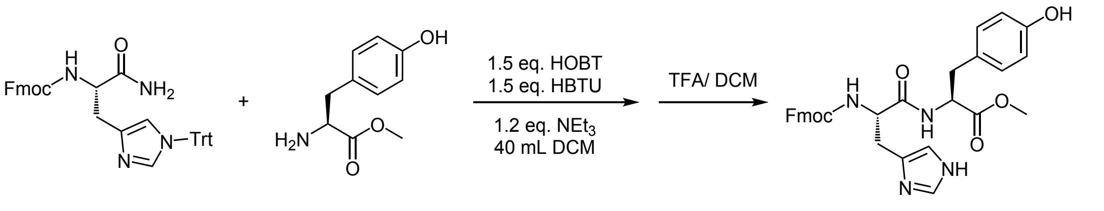

A 2 mmol **B** 2 mmol Dipeptide **4f**

Dipeptide **4f Fmoc-His-Tyr-OMe**, white solid. ^{1}H NMR (400 MHz, DMSO-d_6) δ 12.62 (s, 1H), 9.28 (s, 1H), 8.25 (d, J = 7.6 Hz, 1H), 7.88 (d, J = 7.6 Hz, 3H), 7.70 – 7.57 (m, 3H), 7.43 – 7.38 (m, 2H), 7.34 – 7.28 (m, 2H), 7.00 – 6.87 (m, 3H), 6.65 (d, J = 8.4 Hz, 2H), 4.43 – 4.37 (m, 1H), 4.34 – 4.29 (m, 1H), 4.25 – 4.14 (m, 3H), 3.56 (s, 3H), 3.03 – 2.78 (m, 4H). ^{13}C NMR (101 MHz, DMSO-d_6) δ 171.86, 171.34, 156.08, 155.81, 143.79, 140.70, 135.83, 130.11, 127.69, 127.18, 127.15, 126.84, 125.38, 125.33, 120.14, 115.11, 65.82, 54.97, 54.20, 54.10, 51.90, 46.57, 35.96.

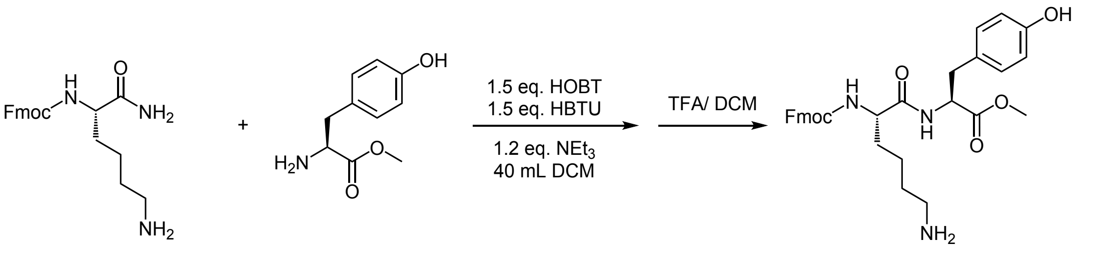

A 2 mmol **B** 2 mmol Dipeptide **4g**

Dipeptide **4g Fmoc-Lys-Tyr-OMe**, white solid. ^{1}H NMR (400 MHz, Methanol-d_4) δ 7.78 (d, J = 7.6 Hz, 2H), 7.64 (t, J = 7.6 Hz, 2H), 7.38 (t, J = 7.2 Hz, 2H), 7.29 (td, J = 7.6, 1.2 Hz, 2H), 7.00 – 6.98 (m, 2H), 6.70 – 6.67 (m, 2H), 4.64 – 4.59 (m, 1H), 4.41 – 4.33 (m, 2H), 4.20 (t, J = 6.4 Hz, 1H), 4.09 – 4.05 (m, 1H), 3.65 (s, 3H), 3.06 – 3.01 (m, 1H), 2.92 – 2.85 (m, 3H), 1.73 – 1.54 (m, 4H), 1.39 – 1.25 (m, 3H). ^{13}C NMR (101 MHz, Methanol-d_4) δ 173.01, 172.07, 156.93, 156.02, 143.95, 143.71, 141.20, 129.94, 127.43, 126.80, 124.75, 119.57, 114.88, 66.54, 54.57, 53.93, 51.33, 39.08, 36.15, 31.19, 26.69, 22.22.

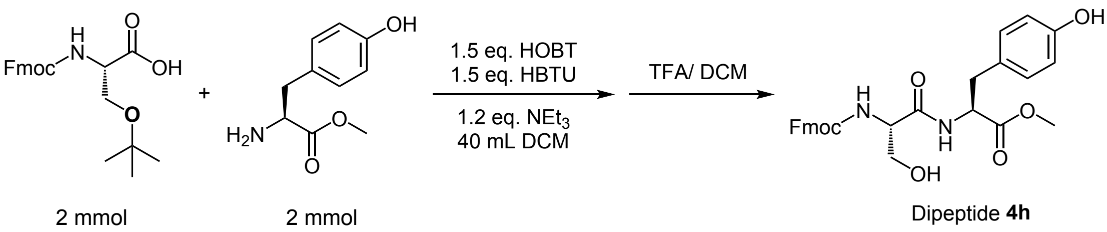

Dipeptide **4h Fmoc-Ser-Tyr-OMe**, white solid. ^{1}H NMR (400 MHz, Acetone-d_6) δ 8.32 (s, 1H), 7.88 (d, J = 7.6 Hz, 2H), 7.76 – 7.73 (m, 2H), 7.60 (d, J = 7.6 Hz, 1H), 7.43 (t, J = 7.6 Hz, 2H), 7.34 (t, J = 7.6 Hz, 2H), 7.07 (d, J = 8.0 Hz, 2H), 6.77 (d, J = 8.0 Hz, 2H), 6.69 (d, J = 8.0 Hz, 1H), 4.74 – 4.69 (m, 1H), 4.36 -4.31 (m, 3H), 4.28 – 4.22 (m, 2H), 3.86 – 3.74 (m, 2H), 3.65 (s, 3H), 3.07 – 3.02 (m, 1H), 3.02 – 2.97 (m, 1H). ^{13}C NMR (101 MHz, Acetone-d_6) δ 172.54, 171.02, 157.15, 157.05, 145.02, 144.91, 142.01, 131.20, 128.50, 127.95, 126.15, 120.76, 116.03, 67.42, 63.25, 57.53, 54.77, 52.34, 47.89, 37.35.

2.4 Synthesis of starting materials dipeptides 4i [2]

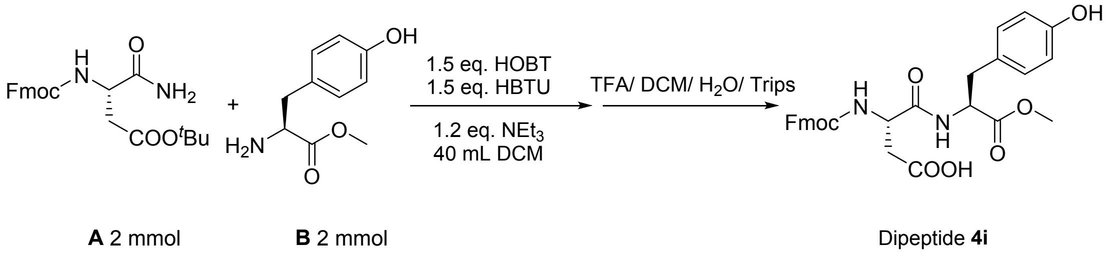

In a round bottomed flask, equipped with a stir bar, peptide **A** (2.0 mmol), HOBT (1-hydroxybenzotriazole) (3.0 mmol), HBTU (O-benzotriazole-*N, N, N', N'*-tetramethyl-uronium-hexafluorophosphate) (3.0 mmol), dichloromethane (40 mL) and triethylamine (2.4 mmol) were combined and added. The mixture was stirred for 30 min at room temperature, and then, peptide **B** (2.0 mmol) was added to the solution. The reaction was stirred overnight. After regular workup, the reaction mixture washed by saturated $NaHCO_3$ solution (40 mL x 3), 2M hydrochloric acid solution (40 mL x 3) and H_2O (40 mL x 3). The organic layers were combined, dried over Na_2SO_4, and concentrated. Without further purification, the 95% TFA /DCM /H_2O /Trips solution (8 ml) was added dropwise. The mixture was stirred for 2 h at room temperature. The resulting crude product was purified by flash chromatography (DCM/ MeOH) to afford

corresponding dipeptides **4i Fmoc-Asp-Tyr-OMe**, white solid. ^{1}H NMR (400 MHz, DMSO-d_6) δ 12.38 (s, 1H), 9.25 (s, 1H), 8.20 (d, J = 7.6 Hz, 1H), 7.89 (d, J = 7.6 Hz, 2H), 7.72 (d, J = 7.2 Hz, 2H), 7.64 (d, J = 8.4 Hz, 1H), 7.44 – 7.39 (m, 2H), 7.35 – 7.30 (m, 2H), 7.00 – 6.97 (m, 2H), 6.69 – 6.64 (m, 1H), 4.42 – 4.33 (m, 2H), 4.31 – 4.16 (m, 3H), 3.57 (s, 3H), 2.91 – 2.80 (m, 2H), 2.62 (dd, J = 16.4, 4.4 Hz, 1H), 2.49 – 2.44 (m, 1H). ^{13}C NMR (101 MHz, DMSO-d_6) δ 171.89, 171.62, 171.16, 156.03, 155.77, 143.83, 140.71, 130.02, 127.67, 127.13, 126.91, 125.33, 120.14, 115.06, 65.77, 54.12, 51.83, 51.04, 46.61, 36.19, 35.74.

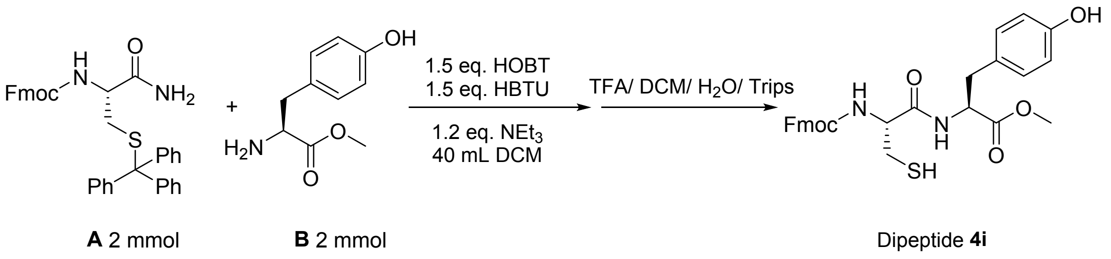

Dipeptide **Fmoc-Cys-Tyr-OMe**, white solid. ^{1}H NMR (400 MHz, MeOD) δ 7.79 (d, J = 7.6 Hz, 2H), 7.68 – 7.65 (m, 2H), 7.40 – 7.35 (m, 2H), 7.32 – 7.27 (m, 2H), 7.01 – 6.98 (m, 2H), 6.71 – 6.66 (m, 2H), 4.60 (dd, J = 8.9, 5.6 Hz, 1H), 4.42 – 4.32 (m, 2H), 4.27 – 4.18 (m, 2H), 3.66 (s, 3H), 3.06 – 3.01 (m, 1H), 2.94 – 2.88 (m, 1H), 2.83 – 2.75 (m, 1H), 2.73 – 2.68 (m, 1H). ^{13}C NMR (101 MHz, MeOD) δ 173.25, 172.45, 158.18, 157.42, 145.27, 145.08, 142.54, 131.30, 128.80, 128.20, 126.26, 120.94, 116.26, 68.01, 58.49, 55.47, 52.71, 48.32, 37.43, 27.17.

2.5 Synthesis of starting materials PTZ-N_3 2g, PTZ-Biotin 2h, PTZ-Probenecid 2i

2.5.1 Synthesis of 2-aminnomethyl phenothiazine [3]

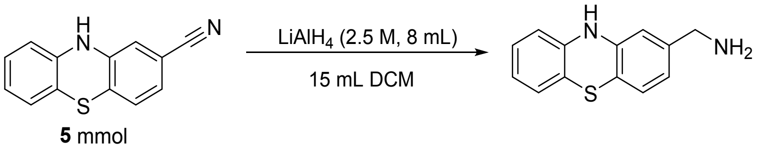

The 2-cyanophenothiazine (5 mmol) was dissolved in DCM (15 mL) and reduced with $LiAlH_4$ (2.5M, 8 mL). After 24 h at room temperature, 0.01 volumes of 1M HCl and 0.02 volumes of water were added to quench the reaction. The resulting light yellow suspension was thoroughly extracted with DCM, and dried. And the crude product was

purified by flash chromatography (DCM/ MeOH) to afford corresponding **2-aminomethyl phenothiazine**.

2.5.2 Synthesis of PTZ-N_3 2g [2][4]

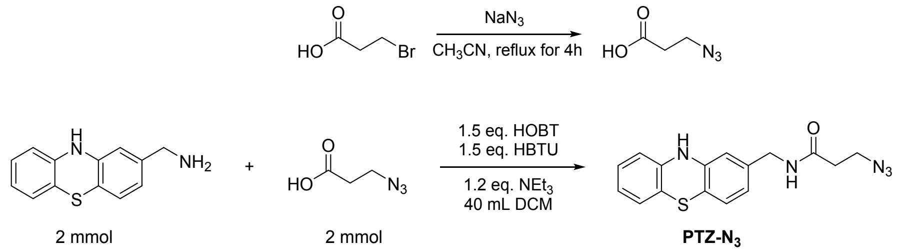

3-Bromopropionic acid (25 mmol) was dissolved in acetonitrile (40 mL), sodium azide was (50 mmol) added to the solution and the mixture was refluxed for 4h. Acetonitrile was then removed under reduced pressure and the resulting residue was suspended in ethyl acetate (50 mL) and extracted with 0.1 N HCl (3 x 40mL), water (3 x 40 mL) and brine (1 x 30 mL). The organic layer was dried over Na_2SO_4 to afford the 3-azidopropionic acid.

In a round bottomed flask, equipped with a stir bar, 3-azidopropionic acid (2.0 mmol), HOBT (1-hydroxybenzotriazole) (3.0 mmol), HBTU (O-benzotriazole-*N, N, N', N'*-tetramethyl-uronium-hexafluorophosphate) (3.0 mmol), dichloromethane (40 mL) and triethylamine (2.4 mmol) were combined and added. The mixture was stirred for 30 min at room temperature, and then, 2-aminnomethyl phenothiazine (2.0 mmol) was added to the solution. The reaction was stirred overnight. After regular workup, the reaction mixture washed with saturated $NaHCO_3$ solution (40 mL x 3), 2M hydrochloric acid solution (40 mL x 3) and H_2O (40 mL x 3). The organic layers were combined, dried over Na_2SO_4, and concentrated. The resulting crude product was purified by flash chromatography (DCM/ MeOH) to afford corresponding **PTZ-N_3**. ^{1}H NMR (400 MHz, DMSO-d_6) δ 8.60 (s, 1H), 8.45 (t, J = 6.0 Hz, 1H), 6.98 (td, J = 7.6, 1.6 Hz, 1H), 6.90 (dd, J = 7.6, 1.2 Hz, 1H), 6.85 (d, J = 8.0 Hz, 1H), 6.74 (td, J = 7.6, 1.2 Hz, 1H), 6.69 – 6.42 (m, 2H), 6.58 (d, J = 2.0 Hz, 1H), 4.13 (d, J = 6.0 Hz, 2H), 3.54 (t, J = 6.4 Hz, 2H), 2.42 (t, J = 6.4 Hz, 2H). ^{13}C NMR (101 MHz, DMSO-d_6) δ 169.41, 142.09, 142.02, 138.93, 127.51, 126.21, 126.06, 121.75, 120.80, 116.38, 114.66, 114.44, 113.40, 46.98, 41.75, 34.52.

2.5.2 Synthesis of PTZ-Biotin 2h [5]

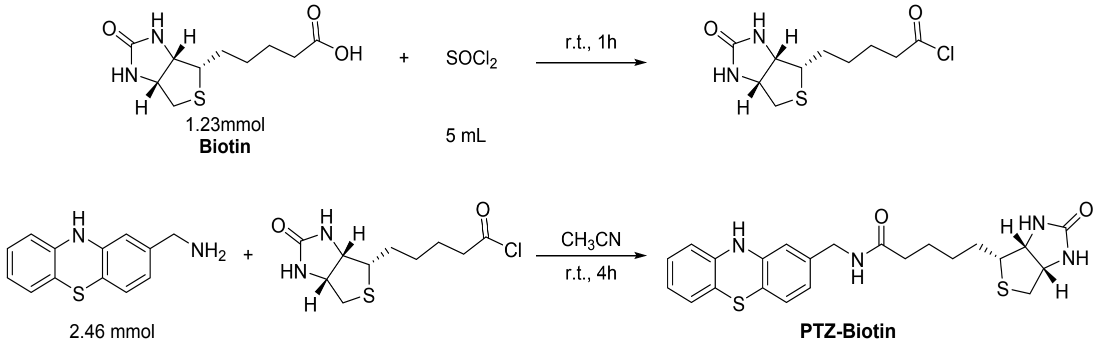

A solution of biotin (1.23 mmol) in $SOCl_2$ (5 mL) was kept at room temperature for 1 h. The reaction mixture was evaporated under vacuum and co-evaporated with anhydrous toluene (3 × 15 mL) to produce biotin acid chloride. The crude product was then dissolved in anhydrous acetonitrile (15 mL) and drop-wise added to a solution of 2- aminnomethyl phenothiazine (2.56 mmol) and Et_3N (3.68 mmol) in acetonitrile (15 mL). The reaction mixture was kept at room temperature for 4 h. Evaporation of the solvent yielded a crude product that was purified by column chromatography (DCM/MeOH) producing **PTZ-Biotin**. ^{1}H NMR (400 MHz, DMSO-d_6) δ 6.96 (td, J = 7.6, 1.6 Hz, 1H), 6.89 (dd, J = 7.6, 1.2 Hz, 1H), 6.83 (d, J = 7.6 Hz, 1H), 6.73 (td, J = 7.6, 1.6 Hz, 1H), 6.68 (dd, J = 8.0, 1.2 Hz, 1H), 6.63 (dd, J = 8.0, 1.6 Hz, 1H), 6.58 (d, J = 2.0 Hz, 1H), 4.31 – 4.28 (m, 1H), 4.13 – 4.11 (m, 3H), 3.10 – 3.05 (m, 1H), 2.82 – 2.76 (m, 1H), 2.57 (d, J = 12.4 Hz, 1H), 2.13 (t, J = 7.6 Hz, 2H), 1.67 – 1.42 (m, 4H), 1.37 – 1.26 (m, 2H). ^{13}C NMR (101 MHz, DMSO-d_6) δ 172.25, 162.99, 142.23, 142.19, 139.53, 127.63, 126.35, 126.17, 121.89, 120.93, 116.60, 114.77, 114.53, 113.48, 61.15, 59.31, 55.64, 45.81, 41.68, 35.29, 28.44, 28.21, 25.49.

2.5.2 Synthesis of PTZ-Probenecid 2i [2]

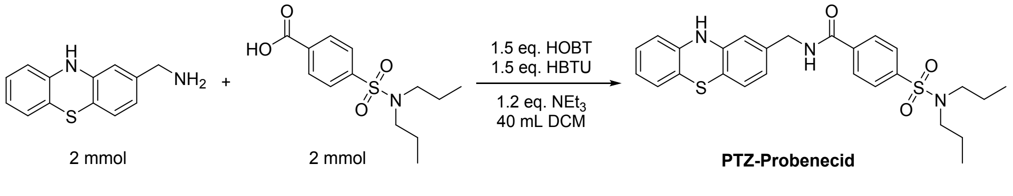

In a round bottomed flask, equipped with a stir bar, Probenecid (2.0 mmol), HOBT (1-

hydroxybenzotriazole) (3.0 mmol), HBTU (O-benzotriazole-*N, N, N', N'*-tetramethyl-uronium-hexafluorophosphate) (3.0 mmol), dichloromethane (40 mL) and triethylamine (2.4 mmol) were combined and added. The mixture was stirred for 30 min at room temperature, and then, 2-aminnomethyl phenothiazine (2.0 mmol) was added to the solution. The reaction was stirred overnight. After regular workup, the reaction mixture washed with saturated $NaHCO_3$ solution (40 mL x 3), 2M hydrochloric acid solution (40 mL x 3) and H_2O (40 mL x 3). The organic layers were combined, dried over Na_2SO_4, and concentrated. The resulting crude product was purified by flash chromatography (DCM/ MeOH) to afford corresponding **PTZ-Probenecid**. ^{1}H NMR (400 MHz, DMSO-d_6) δ 9.27 – 9.24 (m, 1H), 8.61 (s, 1H), 8.10 – 8.07 (m, 2H), 7.92 – 7.90 (m, 2H), 6.97 (td, *J* = 7.6, 1.2 Hz, 1H), 6.92 – 6.86 (m, 2H), 6.75 – 6.71 (m, 2H), 6.67 – 6.65 (m, 2H), 4.35 (d, *J* = 6.0 Hz, 2H), 3.06 – 3.02 (m, 4H), 1.51 – 1.42 (m, 4H), 0.83 – 0.78 (m, 6H). ^{13}C NMR (101 MHz, DMSO-d_6) δ 164.96, 142.14, 142.02, 141.77, 138.96, 137.73, 128.31, 127.51, 126.89, 126.21, 126.09, 121.75, 120.78, 116.37, 114.65, 114.41, 113.18, 49.64, 42.31, 21.64, 11.00.

3. General Procedure

3.1 Reaction optimization

In an oven-dried undivided three-necked bottle (25 mL) equipped with a stir bar, protected tyrosine (0.20 mmol), phenothiazine (0.24 mmol), Na_2SO_4 (0.40 mmol) and solvent (10 mL) were combined and added. The bottle was equipped graphite rod (ϕ 6 mm, about 15 mm immersion depth in solution) as the anode and nickel plate (15 mm×15 mm×1.0 mm) as the cathode and then charged with nitrogen. The reaction mixture was stirred and electrolyzed at constant current under room temperature. When the reaction finished, the reaction mixture was extracted with ethyl acetate (10 mL x 3). The organic layers were combined, dried over Na_2SO_4, and concentrated. The pure product was obtained by flash column chromatography on silica gel (dichloromethane: methanol = 100:1). A summary of optimization results is presented in **Table S1** below.

Table S1. Effects of reaction parameters

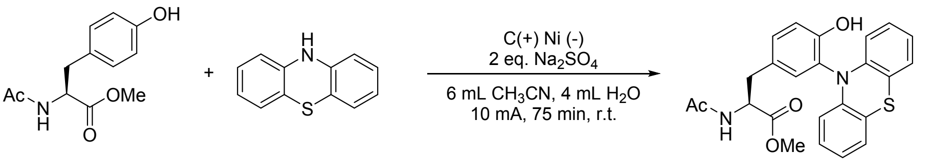

1a 0.2 mmol　　**2a** 0.24 mmol　　**3a**

Entry	Variation from the standard conditions	Isolated yields
1	none	85%
2	C(+) C (-)	51%
3	C(+) Pt (-)	35%
4	5 mA instead of 10 mA, 140 min	86%
5	15 mA instead of 10 mA, 50 min	61%
6	DMSO instead of CH_3CN	n.d.
7	DMF instead of CH_3CN	19%
8	5 mL CH_3CN, 5 mL H_2O was used	74%
9	3 mL CH_3CN, 7 mL H_2O was used	17%
10	without CH_3CN	trace
11	under air	79%
12	without electric current	n.d.

[a]Reaction conditions: graphite rod anode, nickel plate cathode, constant current = 10 mA, **1a** (1.0 equiv., 0.20 mmol), **2a** (1.2 equiv, 0.24 mmol), Na_2SO_4 (2 equiv, 0.40

mmol), CH_3CN/H_2O (6.0 mL/4.0 mL), room temperature, N_2, 75 min (Q=45 C, 2.3 F). Yields of isolated products are shown. n.d. = not detected.

3.2 General procedure for cyclic voltammetry (CV)

Cyclic voltammetry was performed in a three-electrode cell connected to a schlenk line under nitrogen at room temperature. The working electrode was a steady glassy carbon disk electrode, the counter electrode a platinum wire. The reference was an Ag/AgCl electrode submerged in saturated aqueous KCl solution and separated from a reaction by a salt bridge. 2.0 mL of acetonitrile and 2.0 mL of water containing 0.05 M $^nBu_4NBF_4$, 0.005 M substrate were poured into the electrochemical cell in all experiments. The scan rate is 0.1 V/s, ranging from 0 V to 2.0 V. These results showed the oxidation potential of phenothiazine was much lower than these amino acids.

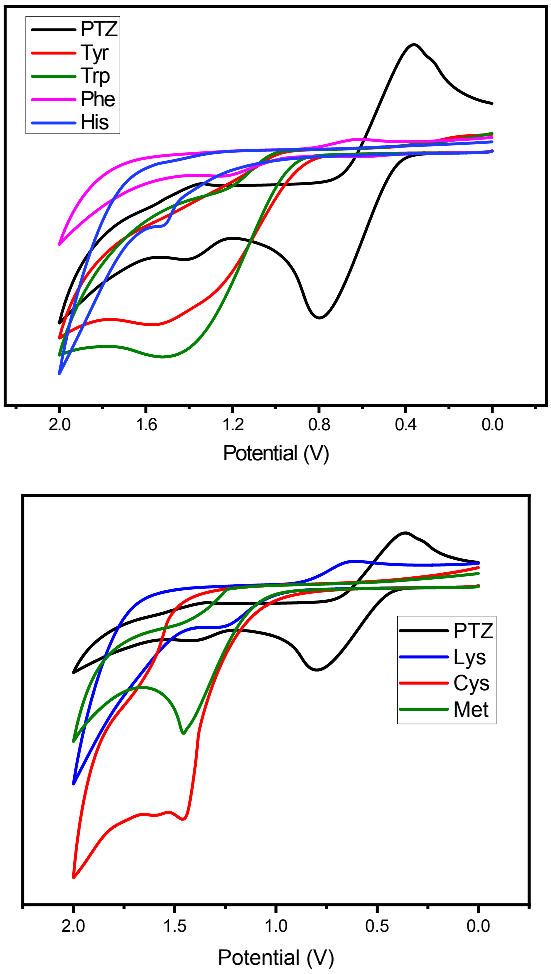

Figure S1

3.3 General procedure for the electron paramagnetic resonance (EPR) experiment

Phenothiazine **2a** was electrolyzed in CH_3CN/H_2O (6.0 mL/4.0 mL) for 15 min. The

samples were taken out by a capillary (borosilicate glass, 0.8-1.1×100 mm), and then recorded by EPR spectrometer at indicated temperature and parameters. The EPR measurement could obtain the following spectrum (Fig. S1, black line, g = 2.0054). After fitting, we proposed that this radical signal belongs to phenothiazine formed nitrogen radical (A_N= 7.2 G, 4*AH_1=3.6G, 4*AH_2= 0.8G).

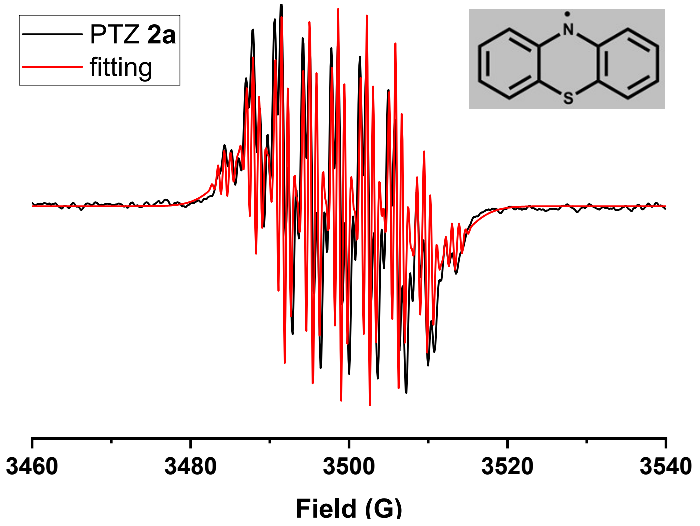

Figure S2

3.4 Proposed mechanism

According to the results of cyclic voltammetry experiments, an oxidation peak of tyrosine **1a** could be observed at 1.3 V (*vs* Ag/AgCl). Oxidation peaks of phenothiazine **2a** was observed at 0.8 V (*vs* Ag/AgCl). Firstly, we performed the reaction between **1a** and **2a** at a controlled potential of 0.8 V where only **2a** could be oxidized, and 77% yield of **3a** could be obtained. This result indicated that the anodic oxidation of phenothiazine might initiate this transformation, while tyrosine has not been oxidized. On the other hand, electron paramagnetic resonance (EPR) experiment was carried out to determine the oxidation species during the electrolysis. Notably, a clear radical signal could be observed which suggested the formation of a nitrogen radical (Figure S2, black line, g = 2.0054).

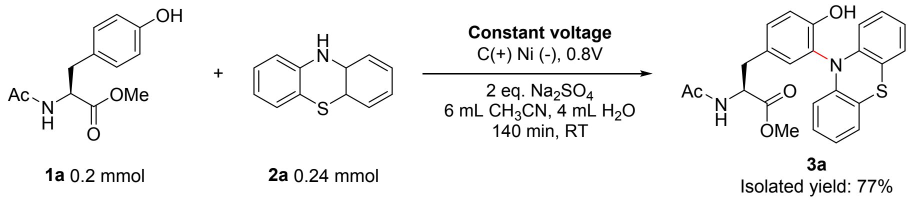

Based on the above experimental results, a plausible reaction mechanism between **1a**

and **2a** was shown in Figure S3. In the first step, **2a** could be oxidized by the carbon anode to generate a nitrogen radical **I**. Following radical addition to the *ortho*-position of phenol would achieve the intermediate **II**. Subsequent oxidation and deprotonation processes could afford the final product.

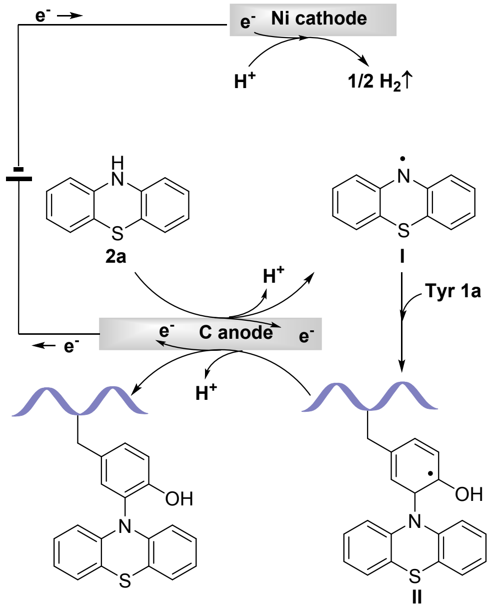

Figure S3

3.5 Phenothiazine scope and characterization

General procedure for bioconjugation of tyrosine and phenothiazine derivatives: In an oven-dried undivided three-necked bottle (25 mL) equipped with a stir bar, protected tyrosine (0.20 mmol), phenothiazine (0.24 mmol), Na_2SO_4 (0.40 mmol) and CH_3CN/H_2O (6 mL/ 4 mL) were combined and added. The bottle was equipped graphite rod (ϕ 6 mm, about 15 mm immersion depth in solution) as the anode and nickel plate (15 mm×15 mm×1.0 mm) as the cathode and then charged with nitrogen. The reaction mixture was stirred and electrolyzed at constant current of 10 mA (j_{anode} ≈11 mA/cm^2) under room temperature for 75 min (2.3 F). When the reaction finished, the reaction mixture was extracted with ethyl acetate (10 mL x 3). The organic layers were combined, dried over Na_2SO_4, and concentrated. The pure product was obtained by flash column chromatography on silica gel (dichloromethane: methanol = 100:1).

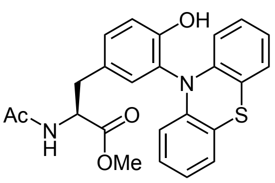

3a

Methyl (S)-2-acetamido-3-(4-hydroxy-3-(10H-phenothiazin-10-yl)phenyl)propanoate (**3a**); 73.8 mg (yield: 85%, 0.2 mmol scale), white solid. ^{1}H NMR (400 MHz, DMSO-d_6) δ 9.90 (s, 1H), 8.37 – 8.35 (m, 1H), 7.27 – 7.25 (m, 1H), 7.10 – 6.79 (m, 8H), 6.07 – 6.05 (m, 2H), 4.51 – 4.70 (m, 1H), 3.57 (s, 3H), 3.02 – 2.99 (m, 1H), 2.88 – 2.82 (m, 1H), 1.76 (s, 3H). ^{13}C NMR (101 MHz, DMSO-d_6) δ 172.28, 169.26, 154.01, 142.79, 132.21, 130.72, 129.83, 127.23, 126.22, 125.86, 122.21, 118.19, 117.34, 115.33, 53.84, 51.83, 35.95, 22.25. HRMS (ESI) calcd for $C_{24}H_{22}N_2O_4S$, [M+Na]$^+$, 457.1192, found 457.1191.

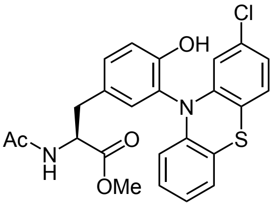

3b

Methyl (S)-2-acetamido-3-(3-(2-chloro-10H-phenothiazin-10-yl)-4-hydroxyphenyl)propanoate (**3b**); 45.0 mg (yield: 48%, 0.2 mmol scale), white solid. ^{1}H NMR (400 MHz, DMSO-d_6) δ 10.00 (s, 1H), 8.32 (d, J = 8.0 Hz, 1H), 7.26 (dd, J = 8.4, 2.4 Hz, 1H), 7.10 – 7.06 (m, 2H), 7.02 – 6.98 (m, 2H), 6.92 – 6.79 (m, 3H), 6.02 (d, J = 8.8 Hz, 1H), 5.94 (d, J = 2.0 Hz, 1H), 4.48 – 4.42 (m, 1H), 3.53 (s, 3H), 2.99 – 2.94 (m, 1H), 2.85 – 2.79 (m, 1H), 1.72 (s, 3H). ^{13}C NMR (101 MHz, DMSO-d_6) δ 172.26, 169.21, 153.76, 144.11, 141.99, 131.80, 131.70, 131.18, 130.08, 127.48, 126.34, 125.25, 122.85, 121.72, 117.88, 117.39, 117.36, 115.66, 114.64, 53.73, 51.76, 35.92, 22.16. HRMS (ESI) calcd for $C_{24}H_{21}ClN_2O_4S$, [M+Na]$^+$, 491.0803, found 491.0800.

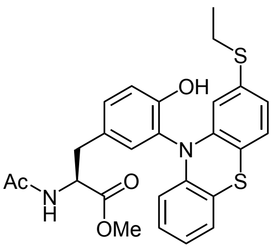

3c

Methyl (S)-2-acetamido-3-(3-(2-(ethylthio)-10H-phenothiazin-10-yl)-4-hydroxyphenyl)propanoate (**3c**); 35.6 mg (yield: 36%, 0.2 mmol scale), white solid. ^{1}H NMR (400 MHz, Acetone-d_6) δ 8.93 (s, 1H), 7.47 (d, J = 8.0 Hz, 1H), 7.31 (dd, J = 8.4, 2.0 Hz, 1H), 7.15 – 7.13 (m, 2H), 6.95 (dd, J = 7.6, 1.6 Hz, 1H), 6.90 – 6.86 (m, 2H), 6.81 – 6.75 (m, 2H), 6.18 – 6.12 (m, 2H), 4.72 – 4.66 (m, 1H), 3.60 (s, 3H), 3.13 – 3.08 (m, 1H), 2.97 – 2.92 (m, 1H), 2.73 (q, J = 7.2 Hz, 2H), 1.83 (s, 3H), 1.14 (t, J = 7.2 Hz, 3H). ^{13}C NMR (101 MHz, Acetone) δ 172.83, 170.09, 155.04, 144.37, 143.79, 136.40, 133.25, 131.91, 131.40, 127.95, 127.22, 127.14, 126.97, 123.23, 122.99, 119.85, 118.31, 117.24, 116.63, 116.29, 54.72, 52.23, 37.52, 27.60, 22.60, 14.58. HRMS (ESI) calcd for $C_{26}H_{26}N_2O_4S_2$, $[M+Na]^+$, 517.1226, found 517.1229.

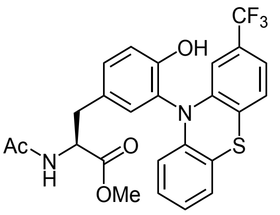

3d

Methyl (S)-2-acetamido-3-(4-hydroxy-3-(2-(trifluoromethyl)-10H-phenothiazin-10-yl)phenyl)propanoate (**3d**); 39.3 mg (yield: 39%, 0.2 mmol scale), white solid. ^{1}H NMR (400 MHz, Acetone-d_6) δ 9.27 (s, 1H), 7.55 (d, J = 8.1 Hz, 1H), 7.33 (dd, J = 8.4, 2.2 Hz, 1H), 7.20 – 7.12 (m, 3H), 7.11 – 7.06 (m, 1H), 6.97 (dd, J = 7.5, 1.6 Hz, 1H), 6.91 (t, J = 8.2, 1H), 6.86 – 6.80 (m, 1H), 6.37 (d, J = 1.7 Hz, 1H), 6.16 (dd, J = 8.2, 1.1 Hz, 1H), 4.76 – 4.66 (m, 1H), 3.58 (s, 3H), 3.13 – 3.08 (m, 1H), 2.95 (dd, J = 13.8, 8.5 Hz, 1H), 1.81 (s, 3H). ^{13}C NMR (101 MHz, Acetone-d_6) δ 171.96, 169.52, 154.19, 143.78, 142.53, 132.19, 131.43, 130.71, 128.70 (q, $J_{C\text{-}F}$ = 32.0 Hz), 127.62, 126.72, 126.26, 125.79, 125.53, 124.61, 122.99, 118.63 (q, $J_{C\text{-}F}$ = 3.9 Hz), 118.02, 117.55, 116.06, 111.29 (q, $J_{C\text{-}F}$ = 4.0 Hz), 53.83, 51.38, 36.63, 21.66. ^{19}F NMR (377 MHz,

Acetone-d_6) δ -63.41. HRMS (ESI) calcd for $C_{25}H_{21}F_3N_2O_4S$, $[M+Na]^+$, 525.1066, found 525.1066.

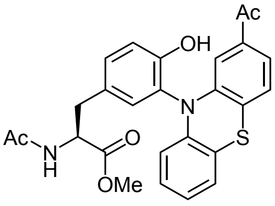

3e

Methyl (S)-2-acetamido-3-(3-(2-acetyl-10H-phenothiazin-10-yl)-4-hydroxyphenyl)propanoate (**3e**); 31.4 mg (yield: 33%, 0.2 mmol scale), yellow solid. ^1H NMR (400 MHz, Acetone-d_6) δ 9.06 (s, 1H), 7.49 – 7.45 (m, 1H), 7.40 (dd, *J* = 8.0, 1.6 Hz, 1H), 7.31 (dd, *J* = 8.6, 2.0 Hz, 1H), 7.17 – 7.13 (m, 2H), 7.05 (d, *J* = 8.0 Hz, 1H), 6.94 (dd, *J* = 7.6, 1.6 Hz, 1H), 6.91 – 6.97 (m, 1H), 6.80 (td, *J* = 7.6, 1.2 Hz, 1H), 6.72 (d, *J* = 2.0 Hz, 1H), 6.14 (dd, *J* = 8.4, 1.2 Hz, 1H), 4.72 – 4.66 (m, 1H), 3.59 (s, 3H), 3.13 – 3.08 (m, 1H), 2.99 – 2.92 (m, 1H), 2.34 (s, 3H), 1.79 (s, 3H). ^{13}C NMR (101 MHz, Acetone-d_6) δ 196.96, 172.80, 170.13, 155.03, 144.01, 143.45, 137.12, 133.11, 132.05, 131.41, 128.37, 126.96, 126.94, 126.76, 126.67, 123.52, 123.37, 118.67, 118.42, 116.69, 114.80, 54.70, 52.22, 37.48, 26.39, 22.53. HRMS (ESI) calcd for $C_{26}H_{24}N_2O_5S$, $[M+Na]^+$, 499.1298, found 499.1302.

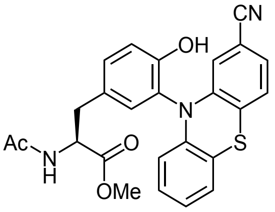

3f

Methyl (S)-2-acetamido-3-(3-(2-cyano-10H-phenothiazin-10-yl)-4-hydroxyphenyl)propanoate (**3f**); 42.3 mg (yield: 46%, 0.2 mmol scale), yellow solid. ^1H NMR (400 MHz, Acetone-d_6) δ 9.28 (s, 1H), 7.57 (d, *J* = 8.0 Hz, 1H), 7.35 (dd, *J* = 8.4, 2.0 Hz, 1H), 7.19 – 7.14 (m, 4H), 7.00 – 6.90 (m, 2H), 6.85 (td, *J* = 7.6, 1.2 Hz, 1H), 6.31 (d, *J* = 1.6 Hz, 1H), 6.17 (dd, *J* = 8.0, 1.2 Hz, 1H), 4.77 – 4.72 (m, 1H), 3.63 (s, 3H), 3.17 – 3.109 (m, 1H), 3.01 – 2.95 (m, 1H), 1.86 (s, 3H). ^{13}C NMR (101 MHz, Acetone-d_6) δ 172.75, 170.23, 154.73, 144.61, 142.88, 132.98, 132.35, 131.59, 128.58, 127.71, 127.21, 127.07, 126.45, 126.29, 123.92, 119.23, 118.58, 118.41, 117.94, 116.86, 111.12, 54.56, 52.25, 37.46, 22.56. HRMS (ESI) calcd for $C_{25}H_{21}N_3O_4S$,

$[M+Na]^+$, 482.1145, found 482.1148.

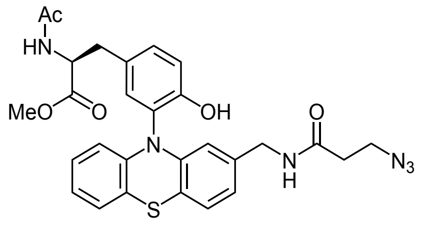

3g

Methyl (S)-2-acetamido-3-(3-(2-((3-azidopropanamido)methyl)-10H-phenothiazin-10-yl)-4-hydroxyphenyl)propanoate (**3g**); 43.7 mg (yield: 39%, 0.2 mmol scale), yellow solid. ^{1}H NMR (400 MHz, Acetone-d_6) δ 7.87 (t, J = 6.4 Hz, 1H), 7.68 (d, J = 8.8 Hz, 1H), 7.30 – 7.27 (m, 1H), 7.14 – 7.11 (m, 2H), 6.94 – 6.92 (m, 1H), 6.88 – 6.84 (m, 2H), 6.80 – 6.71 (m, 2H), 6.19 – 6.17 (m, 1H), 6.05 (s, 1H), 4.75 – 4.69 (m, 1H), 4.28 – 4.21 (m, 1H), 4.05 -3.99 (m, 1H), 3.66 (s, 3H), 3.55 – 3.47 (m, 2H), 3.18 – 3.13 (m, 1H), 2.91 – 2.86 (m, 1H), 2.38 (td, J = 6.8, 2.0 Hz, 2H), 1.77 (s, 3H). ^{13}C NMR (101 MHz, Acetone-d_6) δ 172.68, 170.51, 170.35, 155.10, 144.16, 143.85, 139.52, 133.40, 131.86, 131.37, 127.85, 127.46, 126.90, 126.77, 123.02, 122.11, 119.81, 118.33, 118.17, 116.42, 115.49, 54.73, 52.40, 48.10, 42.91, 37.70, 35.64, 22.57. HRMS (ESI) calcd for $C_{28}H_{28}N_6O_5S$, $[M+Na]^+$, 583.1734, found 583.1738.

3.6 Dipeptide scope and characterization

General procedure for bioconjugation of dipeptide and phenothiazine: In an oven-dried undivided three-necked bottle (25 mL) equipped with a stir bar, dipeptides (0.20 mmol), phenothiazine (0.24 mmol), CH_3CN (6 mL) and phosphate buffer solution (4 mL, pH= 7.3) were combined and added. The bottle was equipped graphite rod (ϕ 6 mm, about 15 mm immersion depth in solution) as the anode and nickel plate (15 mm×15 mm×1 mm) as the cathode and then charged with nitrogen. The reaction mixture was stirred and electrolyzed at constant current of 10 mA (j_{anode} ≈ 11 mA/cm^2) under room temperature for 75 min (2.3 F). When the reaction finished, the reaction mixture was extracted with ethyl acetate (10 mL x 3). The organic layers were combined, dried over Na_2SO_4, and concentrated. The pure product was obtained by flash column chromatography on silica gel (dichloromethane: methanol = 100:1).

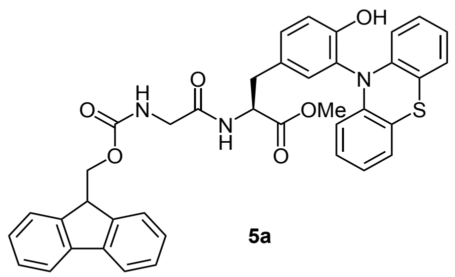

5a

Methyl (S)-2-(2-((((9H-fluoren-9-yl)methoxy)carbonyl)amino)acetamido)-3-(4-hydroxy-3-(10H-phenothiazin-10-yl)phenyl)propanoate (**5a**); 64.4 mg (yield: 48%, 0.2 mmol scale), white solid. ^{1}H NMR (400 MHz, Acetone-d_6) δ 8.82 (s, 1H), 7.84 (d, J = 7.6 Hz, 2H), 7.70 (d, J = 7.6 Hz, 2H), 7.59 (d, J = 8.0 Hz, 1H), 7.39 (t, J = 7.6 Hz, 2H), 7.31 (t, J = 8.0 Hz, 3H), 7.13 – 7.11 (m, 2H), 6.93 – 6.72 (m, 7H), 6.16 (d, J = 8.0 Hz, 2H), 4.79 – 4.73 (m, 1H), 4.36 – 4.19 (m, 3H), 3.89 – 3.77 (m, 2H), 3.58 (s, 3H), 3.16 – 3.12 (m, 1H), 3.02 – 3.97 (m, 1H). ^{13}C NMR (101 MHz, Acetone-d_6) δ 172.41, 169.72, 157.36, 154.94, 144.89, 144.87, 143.93, 141.93, 133.43, 131.87, 130.73, 128.42, 127.85, 127.33, 126.86, 126.06, 123.00, 120.69, 119.77, 118.37, 116.36, 67.28, 54.54, 52.34, 47.80, 44.54, 37.44. HRMS (ESI) calcd for $C_{39}H_{33}N_3O_6S$, $[M+Na]^+$, 694.1982, found 694.1983.

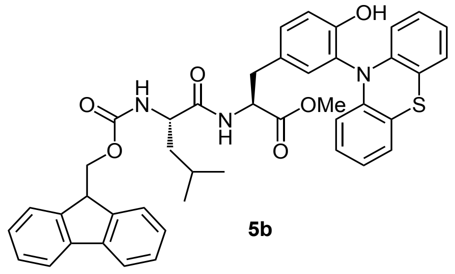

5b

Methyl (S)-2-((S)-2-((((9H-fluoren-9-yl)methoxy)carbonyl)amino)-4-methylpentanamido)-3-(4-hydroxy-3-(10H-phenothiazin-10-yl)phenyl)propanoate (**5b**); 75.6 mg (yield: 52%, 0.2 mmol scale), white solid. ^{1}H NMR (400 MHz, Acetone-d_6) δ 8.71 (s, 1H), 7.84 (d, J = 7.2 Hz, 2H), 7.71 – 7.62 (m, 3H), 7.39 (td, J = 7.2, 2.4 Hz, 2H), 7.32 – 7.27 (m, 3H), 7.12 – 7.10 (m, 2H), 6.93 (dd, J = 7.6, 1.6 Hz, 2H), 6.87 – 6.83 (m, 2H), 6.78 – 6.74 (m, 2H), 6.66 (d, J = 8.4 Hz, 1H), 6.18 (dd, J = 8.4, 1.2 Hz, 2H), 4.78 – 4.72 (m, 1H), 4.40 – 4.20 (m, 4H), 3.56 (s, 3H), 3.15 – 3.10 (m, 1H), 3.06 – 3.00 (m, 1H), 1.76 – 1.62 (m, 1H), 1.60 – 1.52 (m, 2H), 0.91 – 0.87 (m, 6H). ^{13}C NMR (101 MHz, Acetone-d_6) δ 173.08, 172.41, 156.91,

154.89, 145.03, 144.76, 143.96, 141.97, 133.32, 131.98, 130.77, 128.44, 127.89, 127.37, 126.90, 126.07, 123.04, 120.73, 119.86, 118.32, 116.42, 67.07, 54.63, 54.17, 52.31, 47.91, 41.91, 37.31, 25.26, 23.41, 21.92. HRMS (ESI) calcd for $C_{43}H_{41}N_3O_6S$, $[M+Na]^+$, 750.2608, found 750.2609.

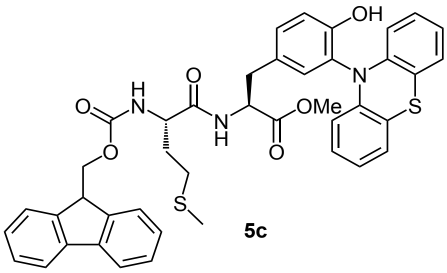

5c

Methyl (S)-2-((S)-2-((((9H-fluoren-9-yl)methoxy)carbonyl)amino)-4-(methylthio)butanamido)-3-(4-hydroxy-3-(10H-phenothiazin-10-yl)phenyl)propanoate (**5c**); 83.5 mg (yield: 56%, 0.2 mmol scale), white solid. ^1H NMR (400 MHz, Acetone-d_6) δ 8.76 (s, 1H), 7.84 (d, J = 7.6 Hz, 2H), 7.75 – 7.67 (m, 3H), 7.42 – 7.38 (m, 2H), 7.34 – 7.29 (m, 3H), 7.15 – 7.13 (m, 2H), 6.95 (dd, J = 7.6, 1.6 Hz, 2H), 6.90 – 6.75 (m, 5H), 6.20 (d, J = 7.6 Hz, 2H), 4.82 – 4.78 (m, 1H), 4.46 – 4.31 (m, 3H), 4.23 (t, J = 7.2 Hz, 1H), 3.58 (s, 3H), 3.19 – 3.14 (m, 1H), 3.09 – 3.03 (m, 1H), 2.61 – 2.49 (m, 2H), 2.16 – 2.08 (m, 1H), 2.05 (s, 3H), 1.99 – 1.92 (m, 1H). ^{13}C NMR (101 MHz, Acetone-d_6) δ 172.35, 172.22, 156.84, 154.87, 144.91, 144.67, 143.89, 141.89, 133.28, 131.88, 130.62, 128.41, 127.86, 127.34, 126.87, 126.01, 123.00, 120.69, 119.82, 118.31, 116.36, 67.15, 54.70, 54.65, 52.36, 47.82, 37.15, 32.76, 30.47, 15.13. HRMS (ESI) calcd for $C_{42}H_{39}N_3O_6S_2$, $[M+Na]^+$, 768.2172, found 768.2185.

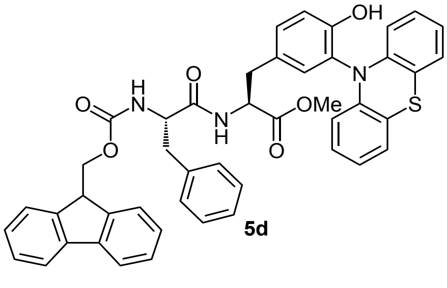

5d

Methyl (S)-2-((S)-2-((((9H-fluoren-9-yl)methoxy)carbonyl)amino)-3-phenylpropanamido)-3-(4-hydroxy-3-(10H-phenothiazin-10-yl)phenyl)propanoate (**5d**); 79.2 mg (yield: 52%, 0.2 mmol scale), white solid. ^1H

NMR (400 MHz, Acetone-d_6) δ 8.78 (s, 1H), 7.82 (d, J = 7.6 Hz, 2H), 7.75 (d, J = 7.6 Hz, 1H), 7.59 (dd, J = 10.8, 7.6 Hz, 2H), 7.38 (t, J = 7.6 Hz, 2H), 7.30 – 7.22 (m,7H), 7.17 (d, J = 6.4 Hz, 1H), 7.13 – 7.11 (m, 2H), 6.92 (d, J = 7.6 Hz, 2H), 6.87 – 6.73 (m, 5H), 6.18 (d, J = 8.0 Hz, 2H), 4.80 – 4.75 (m, 1H), 4.58 – 4.52 (m, 1H), 4.30 – 4.24 (m, 1H), 4.19 – 4.11 (m, 2H), 3.56 (s, 3H), 3.20 – 3.15 (m, 2H), 3.06 – 3.01 (m, 1H), 2.96 – 2.93 (m, 1H). ^{13}C NMR (101 MHz, Acetone-d_6) δ 172.31, 172.10, 156.71, 154.95, 144.84, 143.96, 141.90, 138.38, 133.35, 131.97, 130.69, 130.16, 128.99, 128.42, 127.90, 127.86, 127.22, 126.90, 126.12, 126.05, 123.03, 120.69, 119.83, 118.37, 116.40, 67.22, 57.00, 54.76, 52.37, 47.76, 38.70, 37.40. HRMS (ESI) calcd for $C_{46}H_{39}N_3O_6S$, $[M+Na]^+$, 784.2452, found 784.2465.

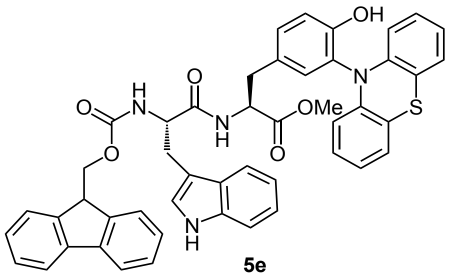

5e

Methyl (S)-2-((S)-2-((((9H-fluoren-9-yl)methoxy)carbonyl)amino)-3-(1H-indol-3-yl)propanamido)-3-(4-hydroxy-3-(10H-phenothiazin-10-yl)phenyl)propanoate

(**5e**); 91.2 mg (yield: 57%, 0.2 mmol scale), white solid. ^{1}H NMR (400 MHz, Acetone-d_6) δ 10.09 (s, 1H), 8.77 (s, 1H), 7.81 (d, J = 7.6 Hz, 2H), 7.66 (d, J = 8.0 Hz, 2H), 7.59 (t, J = 8.0 Hz, 2H), 7.37 (t, J = 7.6 Hz, 3H), 7.28 – 7.21 (m, 4H), 7.11 – 6.99 (m, 4H), 6.92 (dd, J = 7.2, 1.6 Hz, 2H), 6.84 (td, J = 7.6, 1.6 Hz, 2H), 6.74 (td, J = 7.6, 1.2 Hz, 2H), 6.64 (d, J = 7.6 Hz, 1H), 6.17 (d, J = 8.0 Hz, 2H), 4.81 – 4.76 (m, 1H), 4.63 – 4.58 (m, 1H), 4.32 – 4.12 (m, 3H), 3.54 (s, 3H), 3.34 – 3.29 (m, 1H), 3.21 – 3.16 (m, 1H), 3.12 – 3.07 (m, 1H), 3.03 – 2.97 (m, 1H). ^{13}C NMR (101 MHz, Acetone-d_6) δ 172.45, 172.30, 156.76, 154.90, 144.86, 144.77, 143.94, 141.88, 137.43, 133.33, 131.98, 130.64, 128.54, 128.40, 127.88, 127.35, 126.89, 126.06, 124.56, 123.02, 122.07, 120.67, 119.80, 119.55, 119.28, 118.32, 116.39, 112.11, 111.02, 67.21, 56.47, 54.73, 52.34, 47.79, 37.42, 28.81. HRMS (ESI) calcd for $C_{48}H_{40}N_4O_6S$, $[M+Na]^+$, 823.2561, found 823.2578.

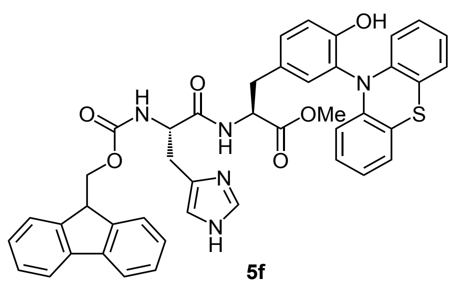

5f

Methyl (S)-2-((S)-2-((((9H-fluoren-9-yl)methoxy)carbonyl)amino)-3-(1H-imidazol-4-yl)propanamido)-3-(4-hydroxy-3-(10H-phenothiazin-10-yl)phenyl)propanoate (**5f**); 60.1 mg (yield: 40%, 0.2 mmol scale), white solid. ^{1}H NMR (400 MHz, Acetone-d_6) δ 7.92 (d, J = 7.6 Hz, 1H), 7.81 (d, J = 7.6 Hz, 2H), 7.62 (t, J = 8.0 Hz, 2H), 7.36 (t, J = 7.6 Hz, 2H), 7.29 – 7.22 (m, 3H), 7.12 – 7.09 (m, 2H), 7.04 – 6.93 (m, 2H), 6.90 (dd, J = 7.6, 1.6 Hz, 2H), 6.85 – 6.81 (m, 2H), 6.72 (td, J = 7.6, 1.2 Hz, 2H), 7.16 (t, J = 7.6 Hz, 2H), 4.74 – 4.68 (m, 1H), 4.55 – 4.49 (m, 1H), 4.28 – 4.13 (m, 3H), 3.52 (s, 3H), 3.14 – 2.92 (m, 4H). ^{13}C NMR (101 MHz, Acetone-d_6) δ 171.71, 171.19, 156.07, 154.30, 144.12, 144.03, 143.23, 141.12, 132.51, 131.19, 129.80, 128.89, 127.64, 127.13, 126.67, 126.43, 126.07, 125.37, 122.19, 119.90, 119.55, 119.02, 117.61, 115.69, 66.53, 54.95, 54.05, 51.61, 47.00, 36.53, 29.51. HRMS (ESI) calcd for $C_{43}H_{37}N_5O_6S$, $[M+Na]^+$, 774.2357, found 774.2357.

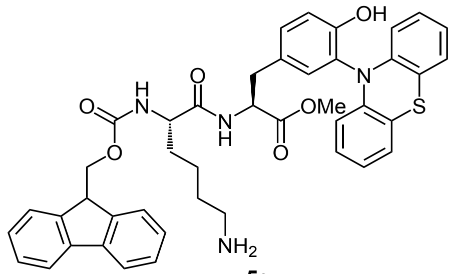

5g

Methyl (S)-2-((S)-2-((((9H-fluoren-9-yl)methoxy)carbonyl)amino)-6-aminohexanamido)-3-(4-hydroxy-3-(10H-phenothiazin-10-yl)phenyl)propanoate (**5g**); 84.7 mg (yield: 57%, 0.2 mmol scale), white solid. ^{1}H NMR (400 MHz, Methanol-d_4) δ 7.78 (d, J = 7.6 Hz, 2H), 7.67 – 7.62 (m, 2H), 7.38 (t, J = 7.6 Hz, 2H), 7.29 (td, J = 7.6, 1.2 Hz, 2H), 7.22 (dd, J = 8.4, 2.0 Hz, 1H), 7.05 – 7.03 (m, 2H), 6.91 (dd, J = 7.6, 1.6 Hz, 2H), 6.84 – 6.80 (m, 2H), 6.75 – 6.71 (m, 2H), 6.15 (d, J = 8.4 Hz, 2H), 4.65 (dd, J = 8.0, 6.0 Hz, 1H), 4.40 – 4.32 (m, 2H), 4.18 (t, J = 6.8 Hz, 1H), 4.12 – 4.08 (m, 1H), 3.59 (s, 3H), 3.13 – 3.08 (m, 1H), 3.01 – 2.96 (m, 1H), 2.76 (t, J = 7.6 Hz,

2H), 1.76 – 1.69 (m, 1H), 1.65 – 1.51 (m, 3H), 1.43 – 1.29 (m, 3H). ^{13}C NMR (101 MHz, Methanol-d_4) δ 173.16, 171.89, 156.87, 154.72, 143.94, 143.69, 143.32, 141.17, 132.16, 130.78, 129.29, 127.43, 126.82, 126.71, 125.87, 124.84, 121.88, 119.58, 119.24, 117.24, 115.38, 66.55, 54.68, 54.03, 51.42, 46.98, 39.77, 36.07, 31.37, 28.63, 22.41. HRMS (ESI) calcd for $C_{43}H_{42}N_4O_6S$, [M+Na]$^+$, 743.2898, found 743.2895.

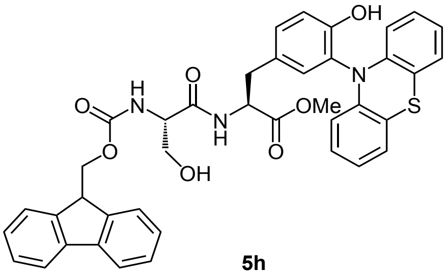

5h

Methyl (S)-2-((S)-2-((((9H-fluoren-9-yl)methoxy)carbonyl)amino)-3-hydroxypropanamido)-3-(4-hydroxy-3-(10H-phenothiazin-10-yl)phenyl)propanoate (5h); 91.1 mg (yield: 65%, 0.2 mmol scale), white solid. ^{1}H NMR (400 MHz, Acetone-d_6) δ 8.72 (s, 1H), 7.85 (d, J = 7.6 Hz, 2H), 7.72 – 7.68 (m, 3H), 7.40 (t, J = 7.6 Hz, 2H), 7.33 – 7.29 (m, 3H), 7.14 – 7.11 (m, 2H), 6.92 (dd, J = 7.6, 1.6 Hz, 2H), 6.89 – 6.84 (m, 2H), 6.76 (dd, J = 7.6, 1.2 Hz, 2H), 6.59 (d, J = 8.0 Hz, 1H), 6.17 (dd, J = 8.0, 1.2 Hz, 2H), 4.80 – 4.75 (m, 1H), 4.33 – 4.22 (m, 5H), 3.86 – 3.73 (m, 2H), 3.58 (s, 3H), 3.18 – 3.13 (m, 1H), 3.08 – 3.03 (m, 1H). ^{13}C NMR (101 MHz, Acetone-d_6) δ 172.48, 171.02, 157.00, 154.97, 145.00, 144.89, 144.01, 142.00, 133.49, 132.05, 130.73, 128.50, 127.94, 127.45, 126.92, 126.15, 123.07, 120.76, 119.88, 118.42, 116.45, 67.42, 63.25, 57.52, 54.76, 52.46, 47.88, 37.35. HRMS (ESI) calcd for $C_{40}H_{35}N_3O_7S$, [M+Na]$^+$, 724.2088, found 724.2092.

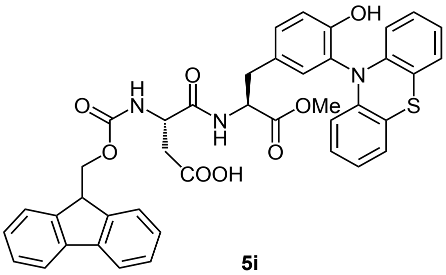

5i

(S)-3-((((9H-Fluoren-9-yl)methoxy)carbonyl)amino)-4-(((S)-3-(4-hydroxy-3-(10H-phenothiazin-10-yl)phenyl)-1-methoxy-1-oxopropan-2-yl)amino)-4-oxobutanoic acid (5i); 67.1 mg (yield: 46%, 0.2 mmol scale), white solid. ^{1}H NMR

(400 MHz, DMSO-d_6) δ 9.93 (s, 1H), 8.39 (d, J = 7.2 Hz, 1H), 7.89 (d, J = 7.2 Hz, 2H), 7.71 (d, J = 7.6 Hz, 2H), 7.59 (d, J = 8.0 Hz, 1H), 7.41 (t, J = 7.6 Hz, 2H), 7.34 – 7.30 (m, 2H), 7.23 (dd, J = 8.4, 2.4 Hz, 1H), 7.10 – 7.04 (m, 2H), 6.95 (dd, J = 7.6, 1.6 Hz, 2H), 6.87 (td, J = 7.6, 1.6 Hz, 2H), 6.75 (td, J = 7.6, 1.2 Hz, 2H), 6.05 (d, J = 8.0 Hz, 2H), 4.52 – 4.35 (m, 2H), 4.26 – 4.11 (m, 3H), 3.47 (s, 3H), 2.96 – 2.90 (m, 2H), 2.60 – 2.55 (m, 1H), 2.47 – 2.42 (m, 1H). ^{13}C NMR (101 MHz, DMSO-d_6) δ 171.87, 171.39, 155.75, 154.08, 143.84, 143.80, 142.79, 140.72, 131.98, 130.77, 129.38, 127.68, 127.25, 127.13, 126.14, 125.96, 125.36, 122.13, 120.14, 118.17, 117.30, 115.40, 65.75, 54.14, 51.76, 51.20, 46.62, 36.71, 35.68. HRMS (ESI) calcd for $C_{41}H_{35}N_3O_8S$, $[M+Na]^+$, 752.2037, found 752.2039.

3.7 Polypeptide scope and characterization

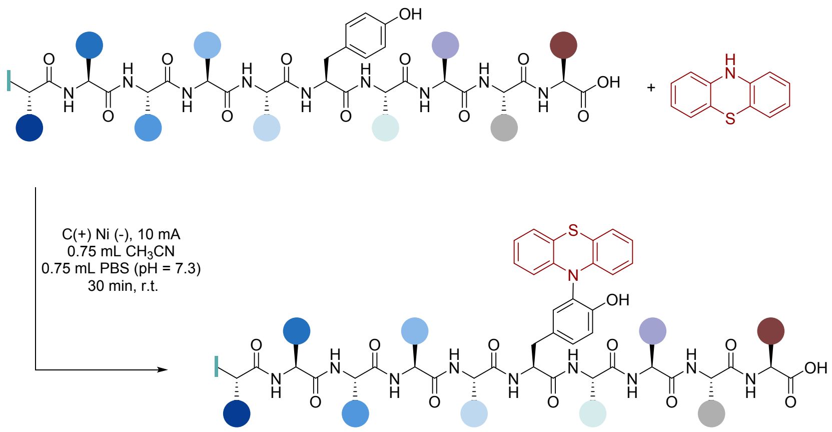

General procedure for bioconjugation of polypeptides and phenothiazine: In an oven-dried undivided three-necked bottle (15 mL) equipped with a stir bar, polypeptides (5 mg), phenothiazine (10 mg or 1 eq.), CH_3CN (0.75 mL) and phosphate buffer solution (0.75 mL, pH= 7.3) were combined and added. The bottle was equipped graphite rod (ϕ 6 mm, about 15 mm immersion depth in solution) as the anode and nickel plate (7.5 mm×15 mm×1 mm) as the cathode and then charged with nitrogen. The reaction mixture was stirred and electrolyzed at constant current of 10 mA (j_{anode} ≈ 11 mA/cm^2) under room temperature for 30 min. After completion of the reaction, the solution was analyzed by MALDI-TOF-MS/MS spectroscopy. The reaction was analyzed by reverse phase HPLC using a gradient of 5% to 50% buffer B over 30

minutes on an Agilent Zorbax 300SB-C18 5 μm column of 100 mm length. HPLC analysis used buffers A (water + 0.1% TFA) and B (9:1 acetonitrile: water + 0.1% TFA). Conversion reported as a % conversion as determined.

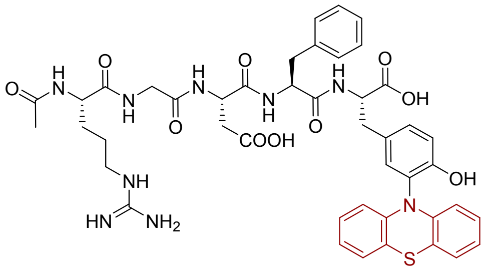

Chain RGD 7a: Ac-RGDFY(PTZ)

HPLC: >99% conversion.

Product **7a** is a peak that elute at 50% buffer B (9:1 acetonitrile: water + 0.1% TFA) with retention times of 9.063 min. Reactant **6a** is a peak that elutes at 50% buffer B with a retention time of 6.823 min. Anisic acid was used as the standard.

HRMS (ESI-TOF) calcd for $C_{44}H_{49}N_9O_{10}S$, $[M+H]^+$, 896.3396, found 896.3051.

HPLC Spectra:

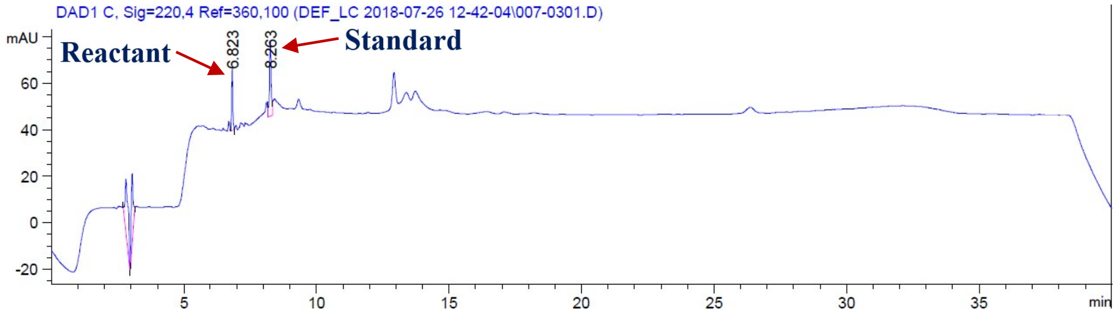

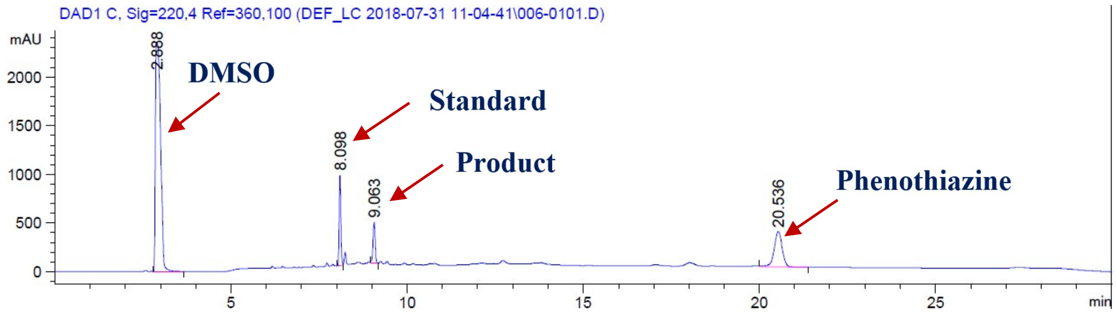

MALDI-TOF-MS/MS Spectra:

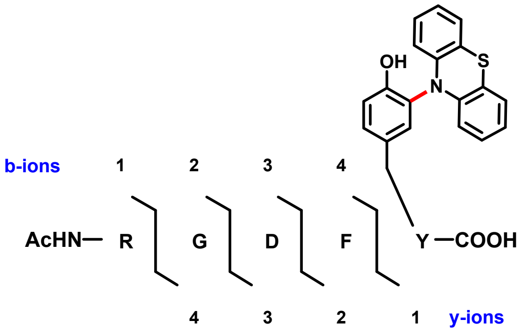

b-ions
1
2
3
4
AcHN— R G D F Y —COOH
4
3
2
1
y-ions
OH
S
N

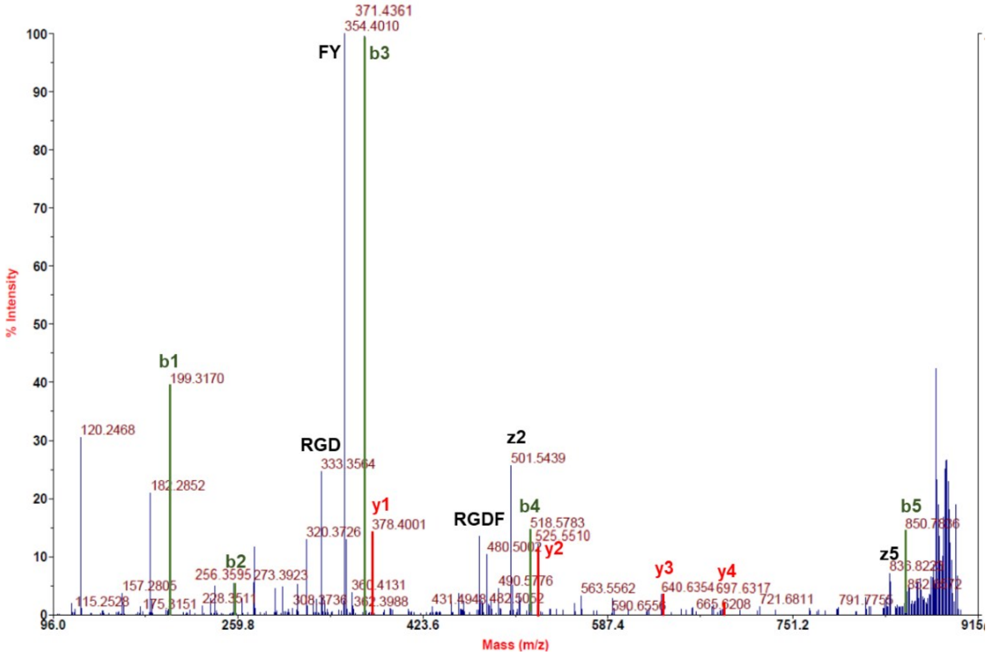

% Intensity
Mass (m/z)
FY
b3
371.4361
354.4010
b1
199.3170
120.2468
RGD
333.3564
182.2852
z2
501.5439
y1
378.4001
RGDF
b4
518.5783
525.5510
y2
b5
850.7836
z5
836.8228
b2
256.3595
273.3923
y3
y4
640.6354
697.6317
721.6811
563.5562
490.5776

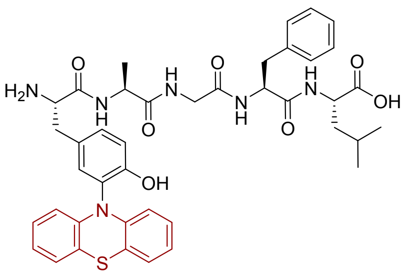

[D-ala2]-leucine encephalin 7b: Y(PTZ)AGFL

HPLC: >99% conversion.

Product **7b** is a peak that elute at 50% buffer B (9:1 acetonitrile: water + 0.1% TFA) with retention times of 9.093 min. Reactant **6b** is a peak that elutes at 50% buffer B with a retention time of 7.096 min. Anisic acid was used as the standard.

HRMS (ESI-TOF) calcd for $C_{41}H_{46}N_6O_7S$, $[M+H]^+$, 767.3221, found 767.2795.

HPLC Spectra:

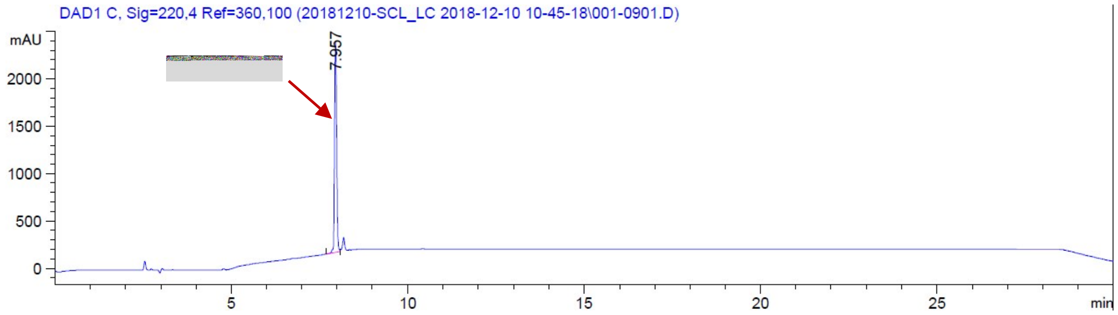

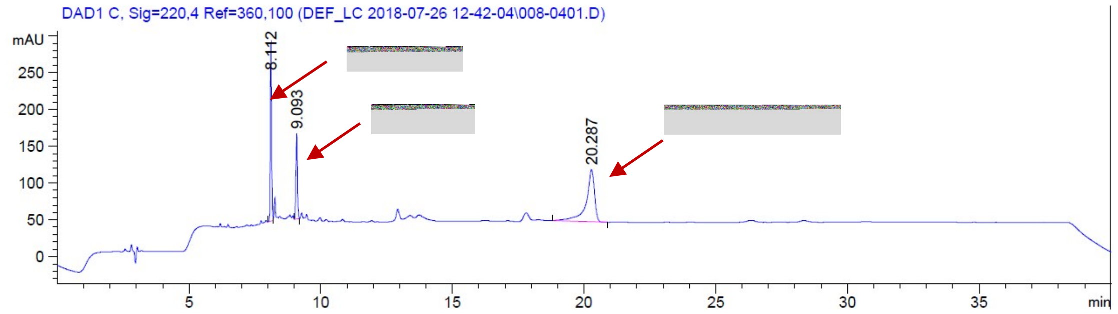

MALDI-TOF-MS/MS Spectra:

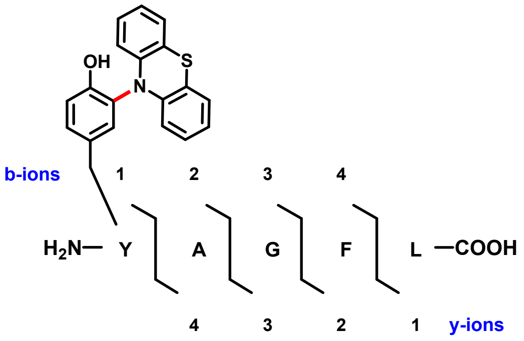

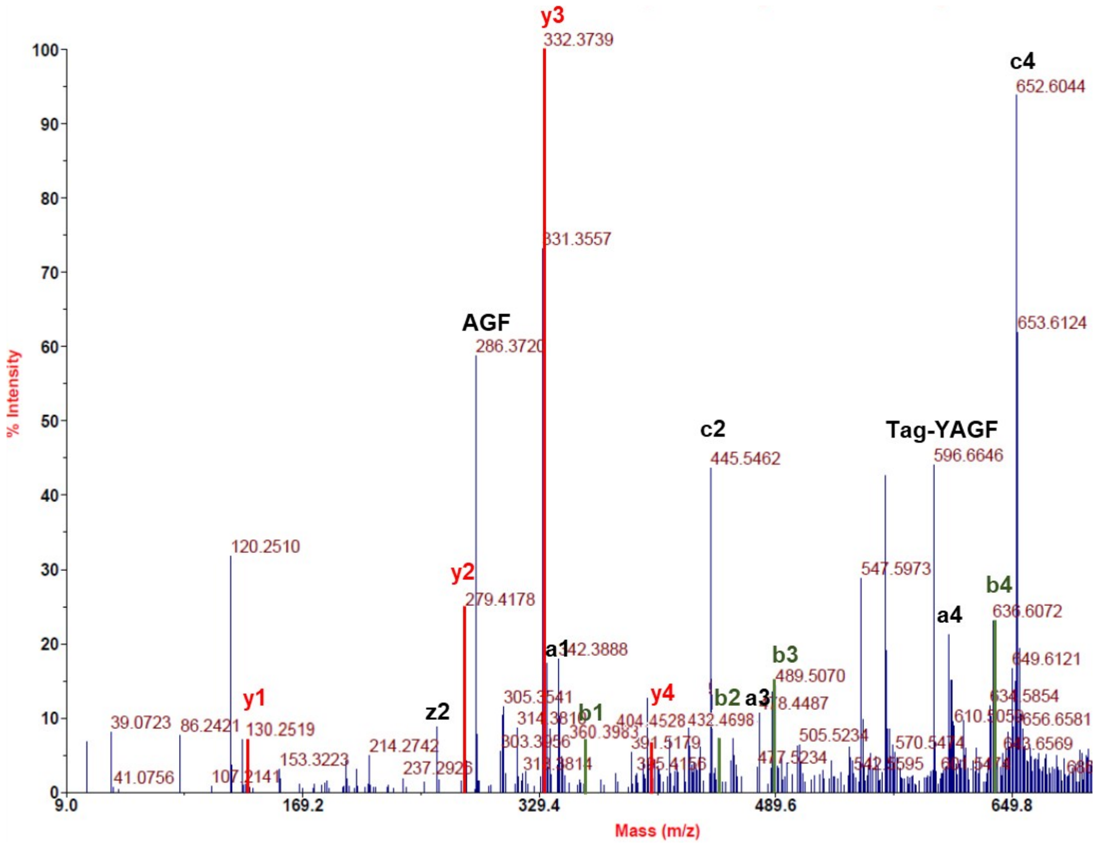
y3
332.3739
c4
652.6044
331.3557
AGF
286.3720
653.6124
c2
445.5462
Tag-YAGF
596.6646
120.2510
y2
279.4178
547.5973
b4
636.6072
a4
a1
342.3888
b3
489.5070
649.6121
y1
y4
b2
a3
z2
b1
305.3541
634.5854
39.0723
86.2421
130.2519
610.5059
656.6581
505.5234
214.2742
360.3983
570.5474
153.3223
237.2926
477.5234
41.0756
% Intensity
Mass (m/z)
9.0
169.2
329.4
489.6
649.8

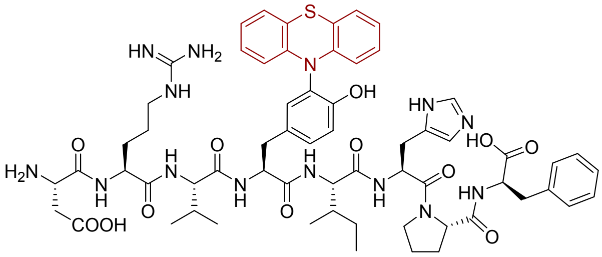

Angiotensin Y 7c: DRVY(PTZ)IHPF

HPLC: >99% conversion.

Product **7c** is a peak that elute at 50% buffer B (9:1 acetonitrile: water + 0.1% TFA) with retention times of 8.848 min. Reactant **6c** is a peak that elutes at 50% buffer B with a retention time of 7.544 min.

HRMS (ESI-TOF) calcd for $C_{62}H_{78}N_{14}O_{12}S$, $[M+H]^+$, 1243.5717, found 1243.5503.

HPLC Spectra:

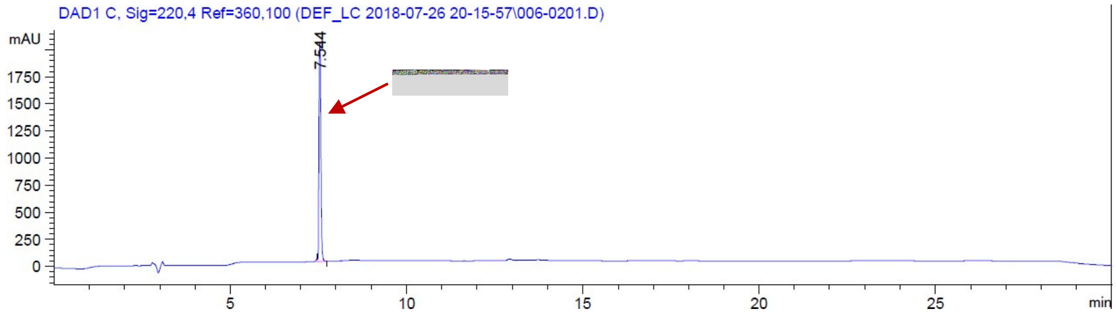

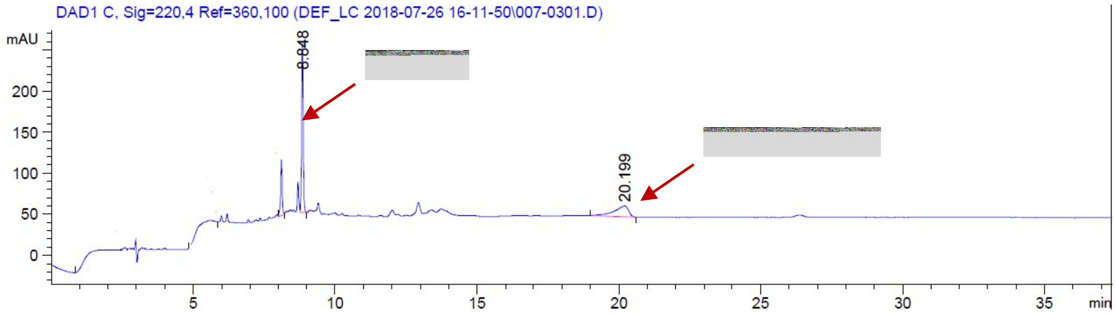

MALDI-TOF-MS/MS Spectra:

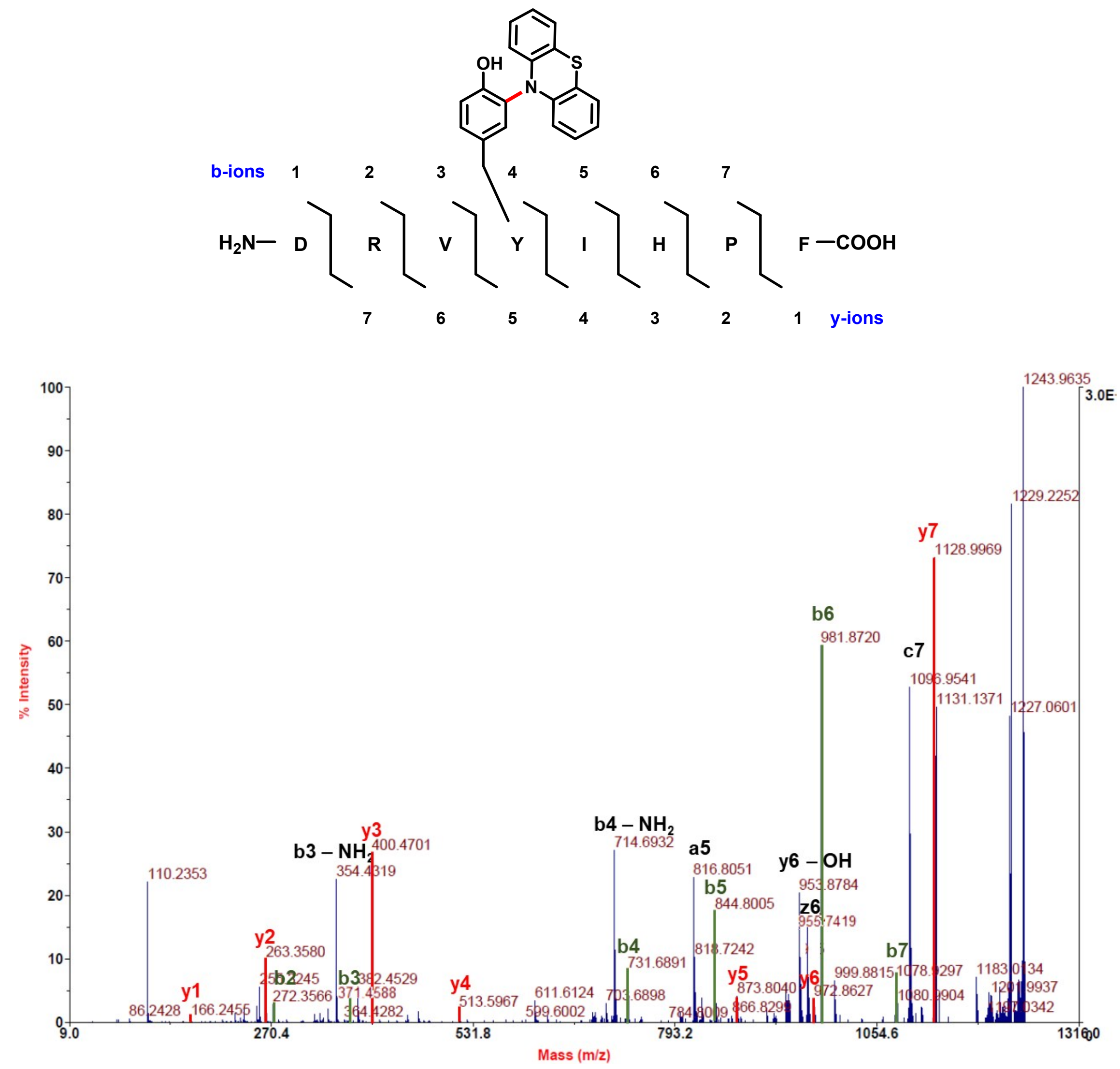
OH
N
S
b-ions
1
2
3
4
5
6
7
H2N—
D
R
V
Y
I
H
P
F
—COOH
7
6
5
4
3
2
1
y-ions
% Intensity
Mass (m/z)
1243.9635
3.0E
1229.2252
y7
1128.9969
b6
981.8720
c7
1096.9541
1131.1371
1227.0601
b4 – NH2
714.6932
y3
400.4701
b3 – NH2
354.4319
a5
816.8051
y6 – OH
953.8784
110.2353
b5
844.8005
z6
955.7419
y2
263.3580
b4
731.6891
818.7242
b7
y5
y6
999.8815
1078.9297
1183.0134
y1
b2
b3
382.4529
y4
611.6124
272.3566
371.4588
513.5967
703.6898
873.8040
972.8627
1080.9904
86.2428
166.2455
364.4282
599.6002
866.8299
9.0
270.4
531.8
793.2
1054.6

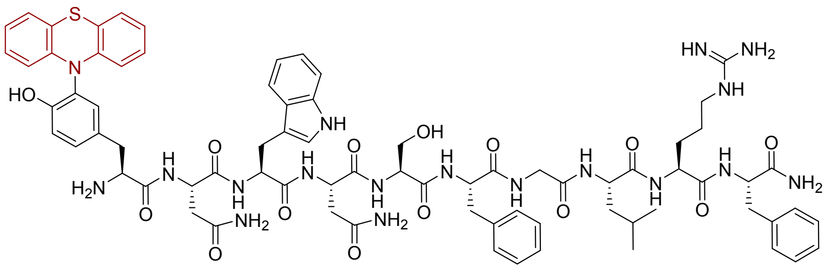

Kisspeptin 10 7d: Y(PTZ)NWNSFGLRF-NH$_2$

HPLC: >99% conversion.

Product **7d** is a peak that elute at 50% buffer B (9:1 acetonitrile: water + 0.1% TFA) with retention times of 9.553 min. Reactant **6d** is a peak that elutes at 50% buffer B with a retention time of 8.267 min. Anisic acid was used as the standard.

HRMS (ESI-TOF) calcd for $C_{75}H_{90}N_{18}O_{14}S$, $[M+H]^+$, 1499.6677, found 1499.5795.

HPLC Spectra

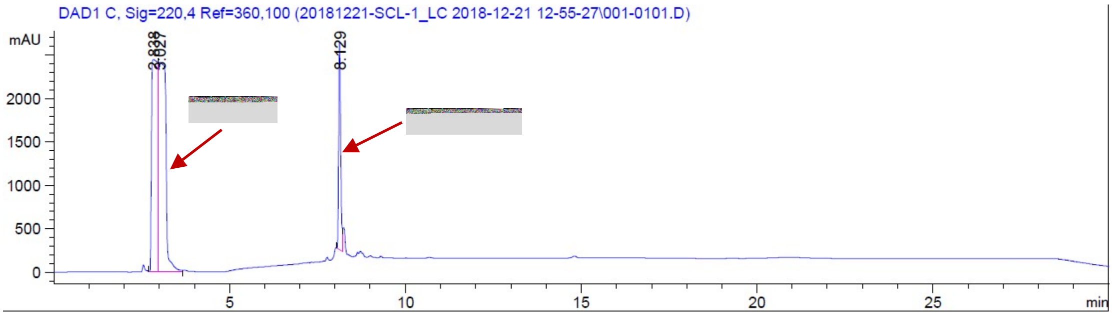

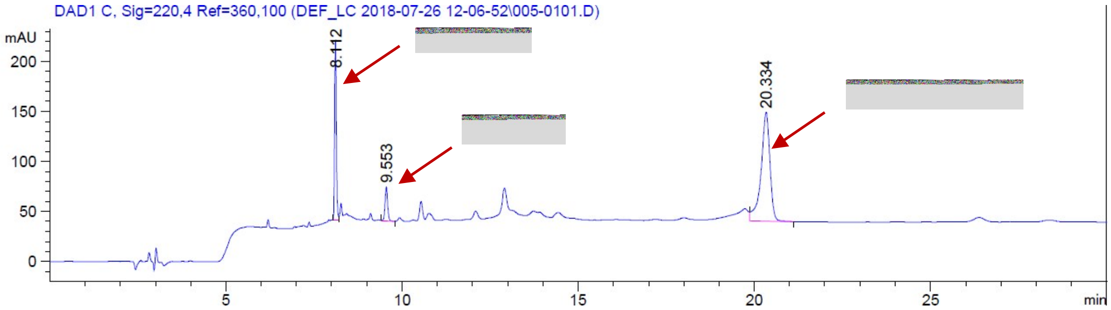

MALDI-TOF-MS/MS Spectra:

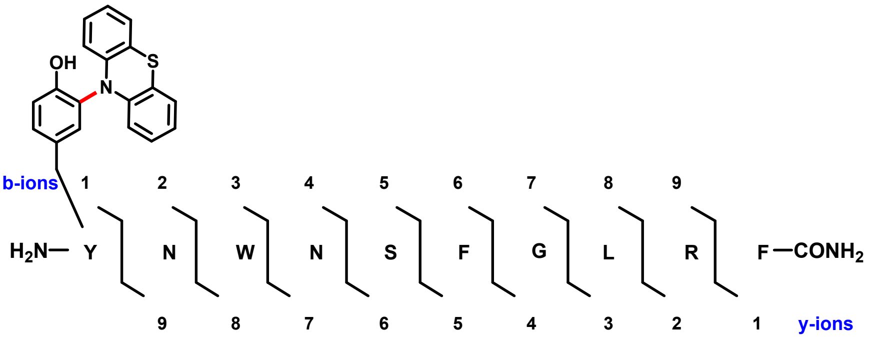
OH
S
N
b-ions
1 2 3 4 5 6 7 8 9
H_2N— Y N W N S F G L R F—$CONH_2$
9 8 7 6 5 4 3 2 1
y-ions

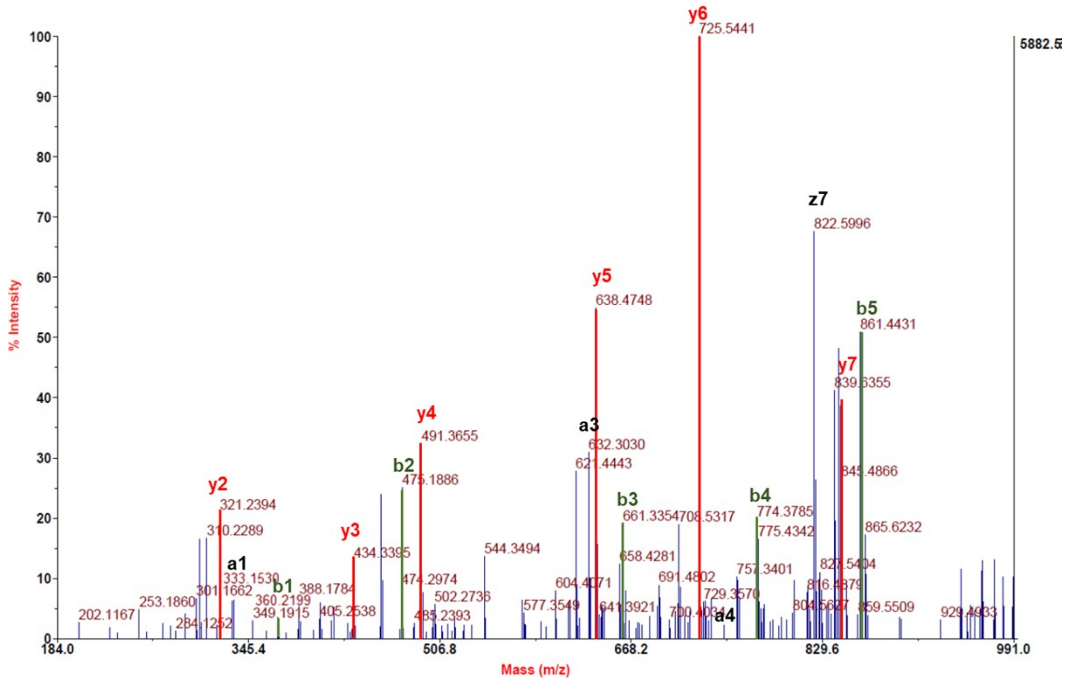
% Intensity
Mass (m/z)
y6 725.5441
5882.5
z7 822.5996
y5 638.4748
b5 861.4431
y7 839.6355
y4 491.3655
a3 632.3030
621.4443
b2 475.1886
845.4866
y2 321.2394
b3 661.3354
708.5317
b4 774.3785
775.4342
865.6232
310.2289
y3 434.3395
544.3494
a1 333.1530
658.4281
757.3401
827.5404
b1
388.1784
301.1662
474.2974
691.4802
816.4879
360.2199
502.2736
604.4071
729.3570
253.1860
349.1915
405.2538
577.3549
641.3921
859.5509
202.1167
284.1252
485.2393
700.4094
a4
929.4933
184.0
345.4
506.8
668.2
829.6
991.0

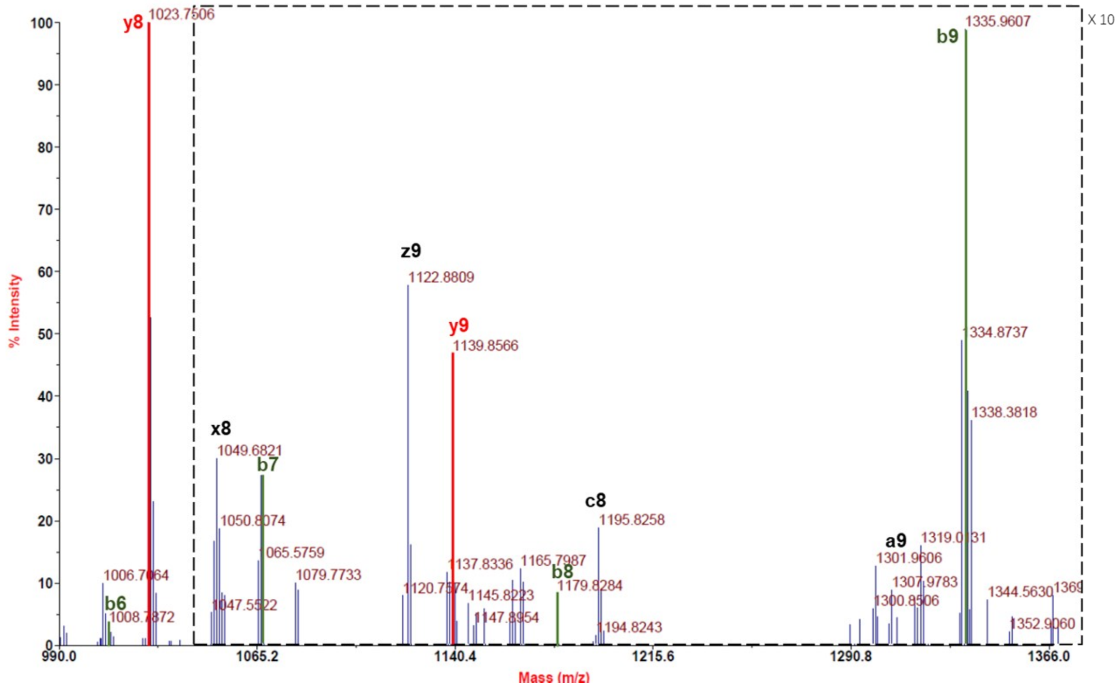
% Intensity
Mass (m/z)
y8 1023.7506
X 10
b9 1335.9607
z9 1122.8809
y9 1139.8566
1334.8737
1338.3818
x8 1049.6821
b7
c8 1195.8258
1050.8074
1065.5759
a9 1319.0131
1301.9606
1137.8336
1165.7987
b8
1006.7064
1079.7733
1120.7574
1179.8284
1307.9783
b6
1145.8223
1344.5630
1008.7872
1047.5522
1147.8954
1300.8506
1352.9060
1194.8243
990.0
1065.2
1140.4
1215.6
1290.8
1366.0

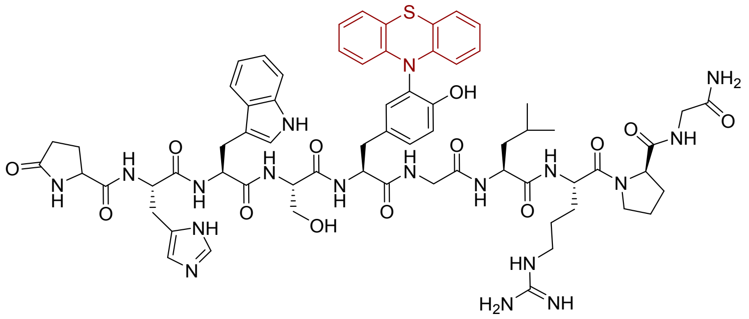

LH-RH 7e: PHWSY(PTZ)GLRPG-NH$_2$

HPLC: >99% conversion.

Product **7e** is a peak that elute at 50% buffer B (9:1 acetonitrile: water + 0.1% TFA) with retention times of 8.656 min. Reactant **6e** is a peak that elutes at 50% buffer B with a retention time of 7.183 min.

HRMS (ESI-TOF) calcd for $C_{67}H_{82}N_{18}O_{13}S$, $[M+H]^+$, 1379.6102, found 1379.5334.

HPLC Spectra:

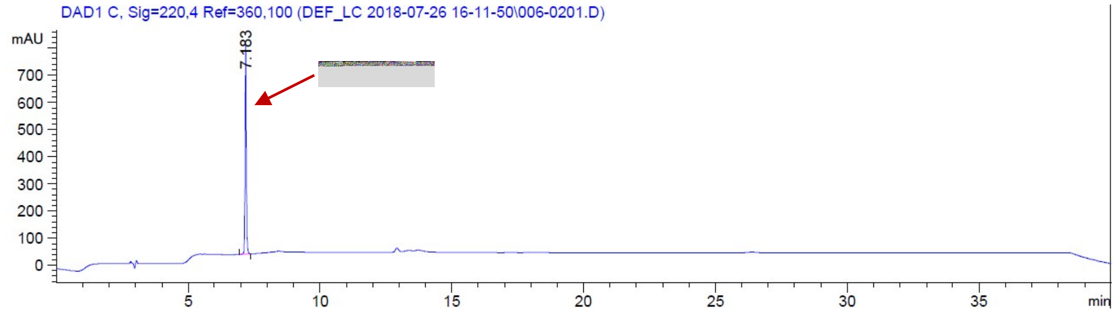

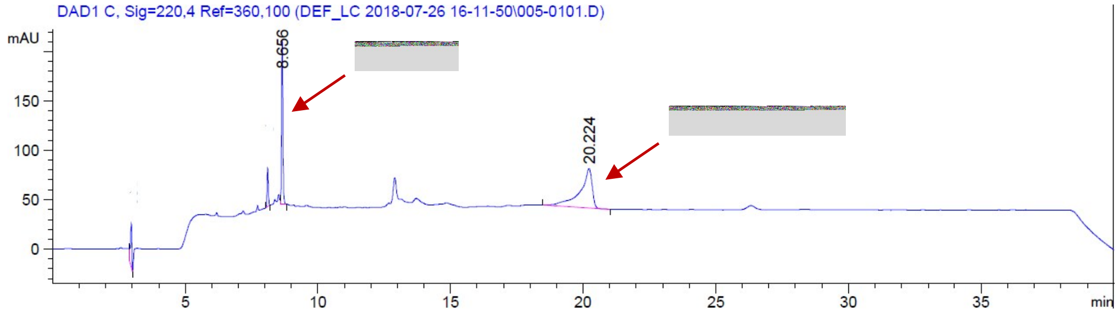

MALDI-TOF-MS/MS Spectra：

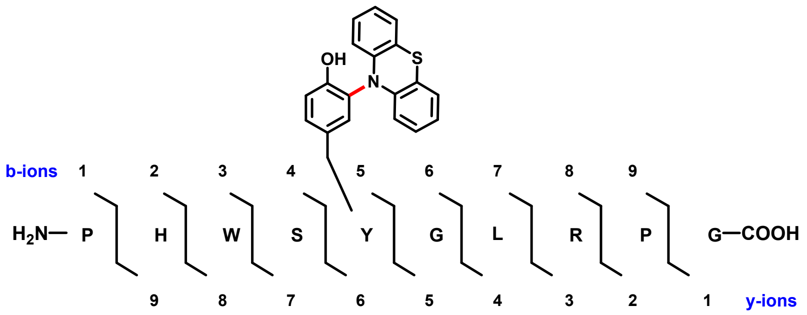
OH
N
S
b-ions
1 2 3 4 5 6 7 8 9
H_2N— P H W S Y G L R P G—COOH
9 8 7 6 5 4 3 2 1
y-ions

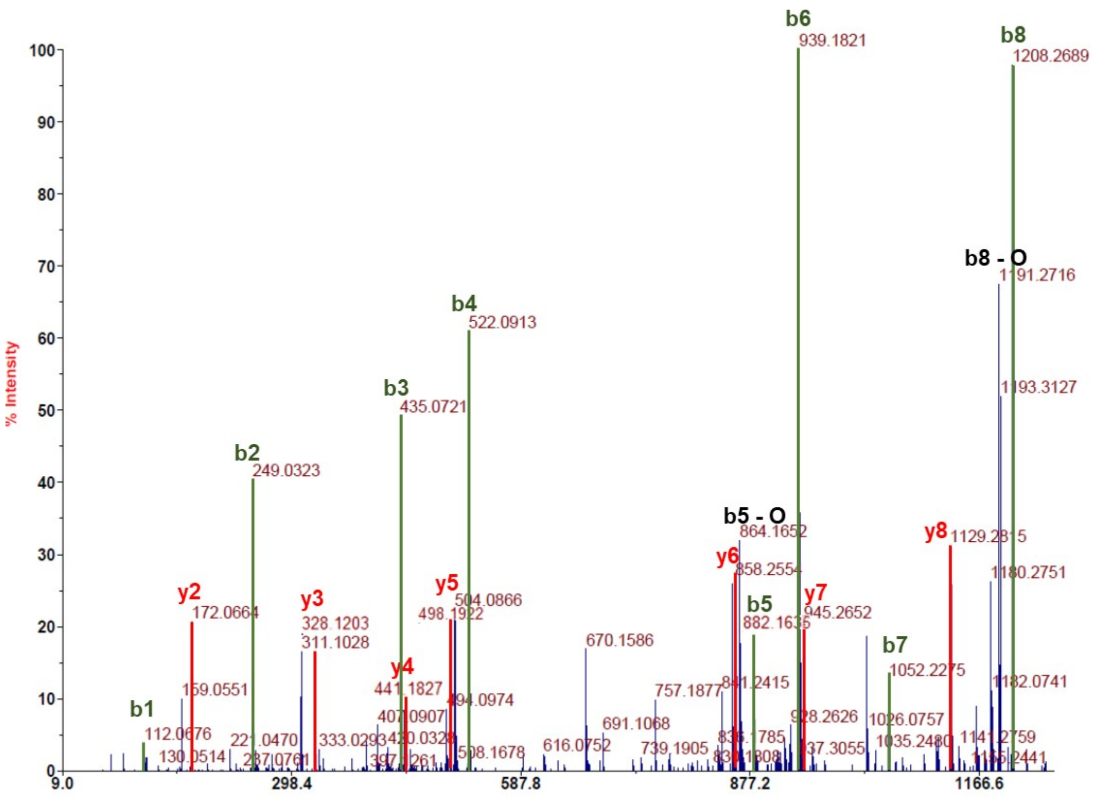
% Intensity
b6 939.1821
b8 1208.2689
b8 - O 1191.2716
1193.3127
b4 522.0913
b3 435.0721
b2 249.0323
b5 - O 864.1652
y8 1129.2815
y6 858.2554
1180.2751
y2 172.0664
y3 328.1203
311.1028
y5 504.0866
498.1922
b5 882.1635
y7 945.2652
670.1586
b7 1052.2275
y4 441.1827
159.0551
b1
494.0974
757.1877
1182.0741
112.0676
691.1068
928.2626
1026.0757
221.0470
333.0293
130.0514
508.1678
616.0752
739.1905
1035.2480
1141.2759
9.0 298.4 587.8 877.2 1166.6
0 10 20 30 40 50 60 70 80 90 100

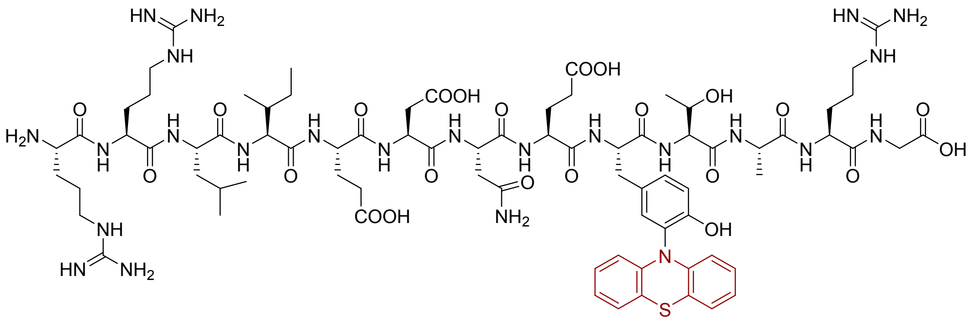

Tyrosine kinase 7f: RRLIEDNEY(PTZ)TARG

HPLC: >99% conversion.

Products **7f** are two peaks that elute at 50% buffer B (9:1 acetonitrile: water + 0.1% TFA) with retention times of 8.754 min and 9.063 min. Reactant **6f** is a peak that elutes at 50% buffer B with a retention time of 7.451 min.

HRMS (ESI-TOF) calcd for $C_{78}H_{116}N_{24}O_{23}S$, $[M+H]^+$, 1789.8439, found 1789.8015.

HPLC Spectra:

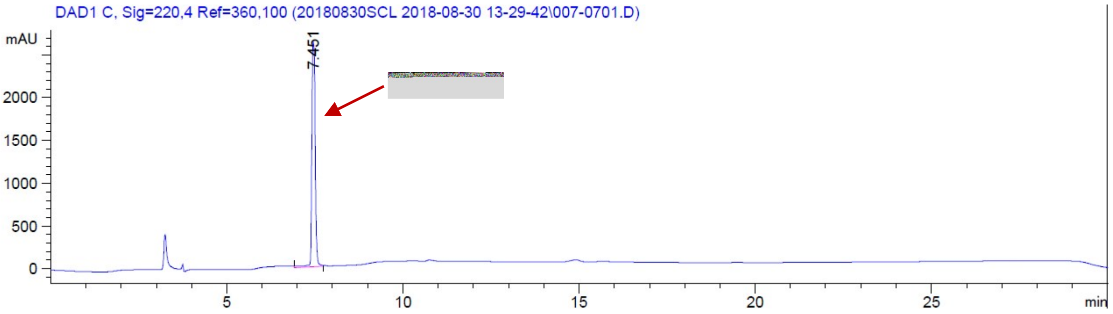

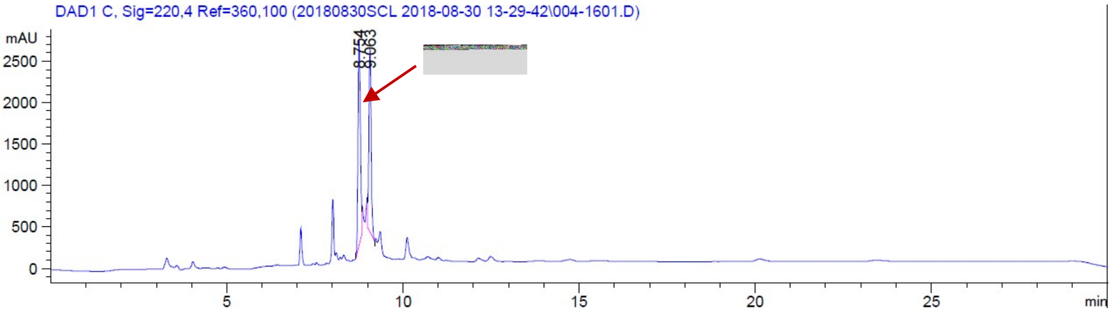

MALDI-TOF-MS/MS Spectra:

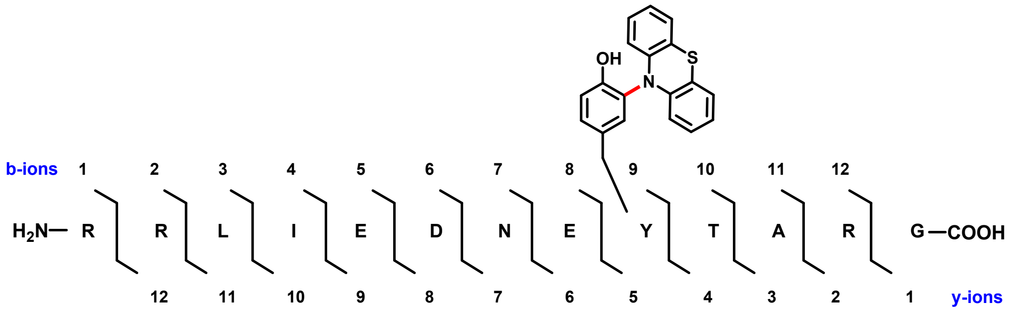

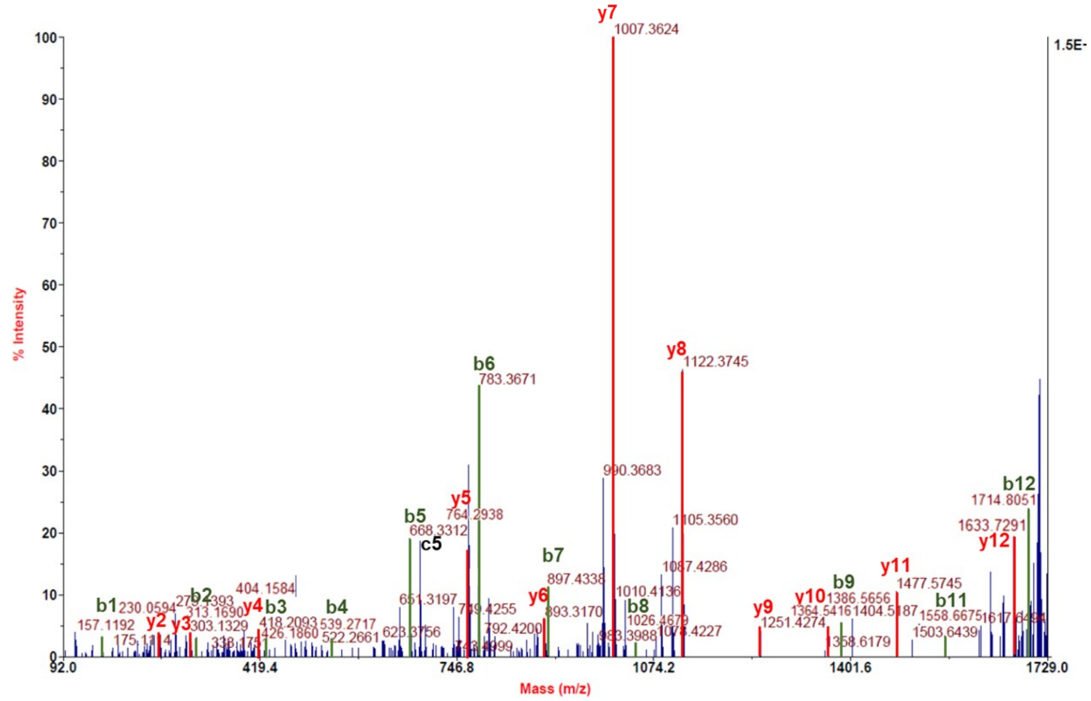

y7
1007.3624
y8
1122.3745
b6
783.3671
b12
1714.8051
y12
1633.7291
y5
764.2938
b5
668.3312
c5
b7
897.4338
990.3683
1105.3560
1087.4286
y11
1477.5745
b9
1386.5656
y10
1364.5416
1404.5187
b11
1558.6675
1503.6439
y9
1251.4274
1358.6179
b8
1026.4679
1010.4136
y6
893.3170
792.4200
651.3197
623.3756
b4
539.2717
522.2661
b3
418.2093
426.1860
y4
404.1584
b2
313.1690
y3
303.1329
y2
b1
230.0594
157.1192
% Intensity
Mass (m/z)
1.5E-

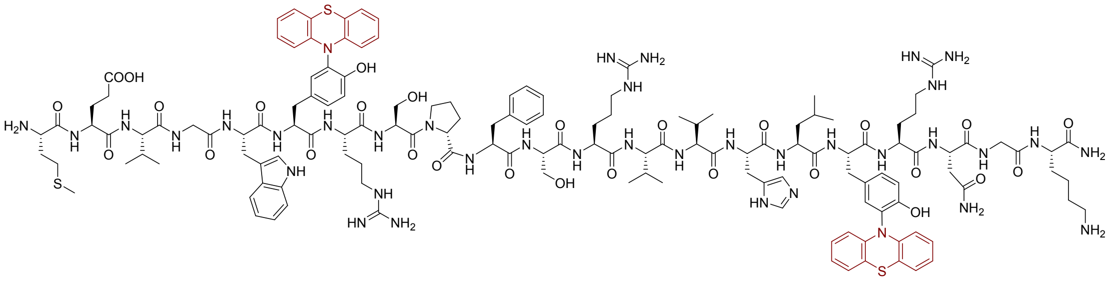

MOG 35-55 7g: MEVGWY(PTZ)RSPFSRVVHLY(PTZ)RNGK

HPLC: >99% conversion.

Product **7g** is a peak that elute at 50% buffer B (9:1 acetonitrile: water + 0.1% TFA) with retention times of 9.103 min. Reactant **6g** is a peak that elutes at 50% buffer B with a retention time of 8.484 min.

HRMS (ESI-TOF) calcd for $C_{130}H_{184}N_{36}O_{29}S_2$, $[M+H]^+$, 2974.4003, found 2976.8679.

HPLC Spectra:

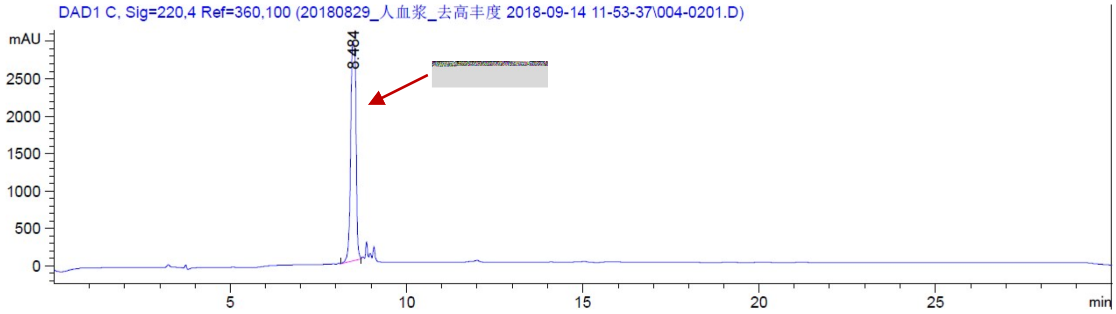

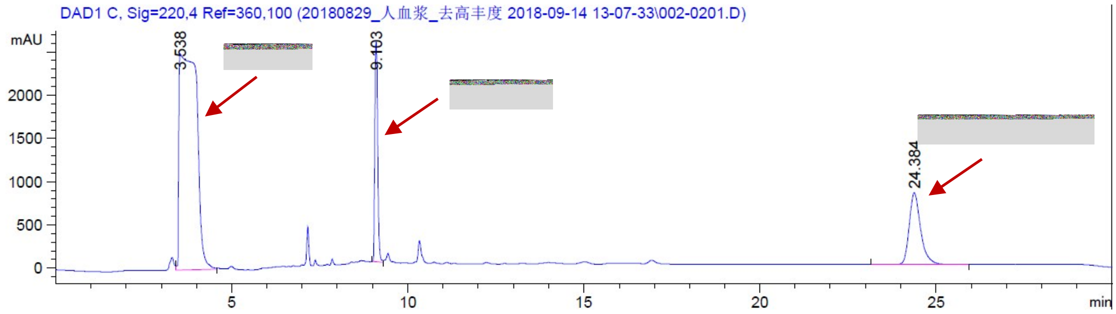

MALDI-TOF-MS/MS Spectra:

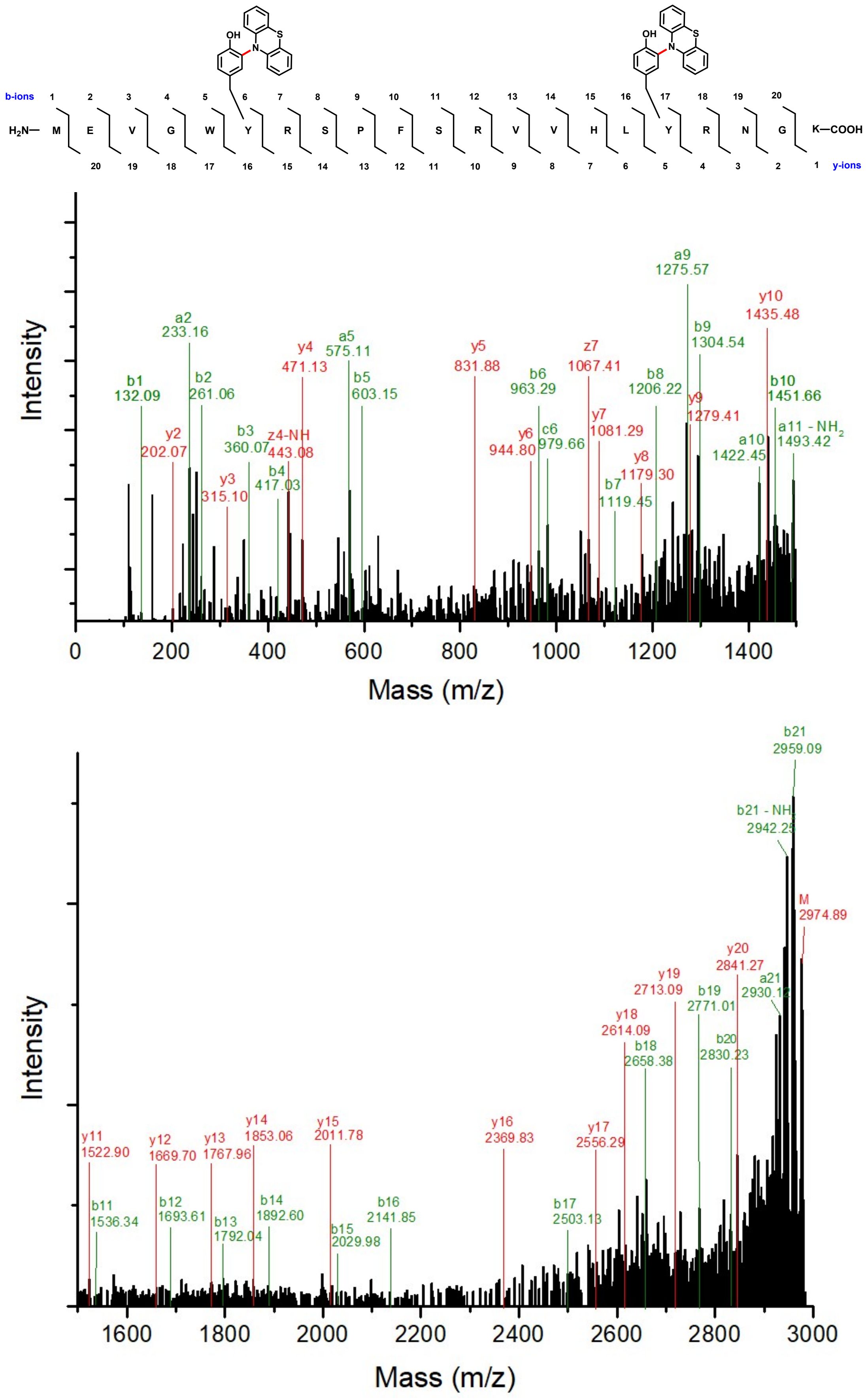
b-ions
H2N— M E V G W Y R S P F S R V V H L Y R N G K—COOH
y-ions
OH
N
S
Intensity
Mass (m/z)
b1 132.09
y2 202.07
a2 233.16
b2 261.06
y3 315.10
b3 360.07
b4 417.03
z4-NH 443.08
y4 471.13
a5 575.11
b5 603.15
y5 831.88
y6 944.80
b6 963.29
c6 979.66
z7 1067.41
y7 1081.29
b7 1119.45
y8 1179.30
b8 1206.22
a9 1275.57
y9 1279.41
b9 1304.54
a10 1422.45
y10 1435.48
b10 1451.66
a11 - NH2 1493.42
y11 1522.90
b11 1536.34
y12 1669.70
b12 1693.61
y13 1767.96
b13 1792.04
y14 1853.06
b14 1892.60
y15 2011.78
b15 2029.98
b16 2141.85
y16 2369.83
b17 2503.13
y17 2556.29
y18 2614.09
b18 2658.38
y19 2713.09
b19 2771.01
b20 2830.23
y20 2841.27
a21 2930.12
b21 - NH3 2942.25
b21 2959.09
M 2974.89

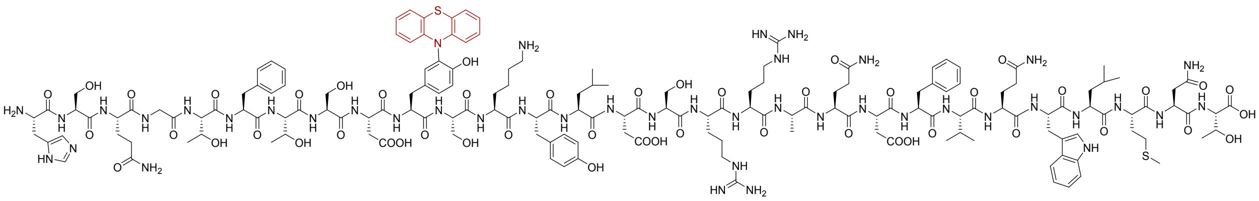

Glucagon 7h: HSQGTFTSDY(PTZ)SKYLDSRRAQDFVQWLMNT

HPLC: >99% conversion.

Product **7h** is a peak that elute at 50% buffer B (9:1 acetonitrile: water + 0.1% TFA) with retention times of 9.09 min. Reactant **6h** is a peak that elutes at 50% buffer B with a retention time of 9.143 min.

HRMS (ESI-TOF) calcd for $C_{165}H_{232}N_{44}O_{49}S_2$, $[M+H]^+$, 3678.6529, found 3678.6371.

HPLC Spectra:

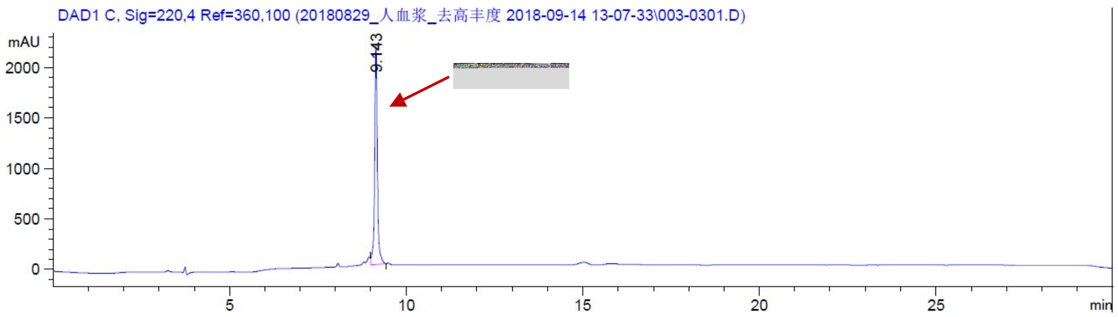

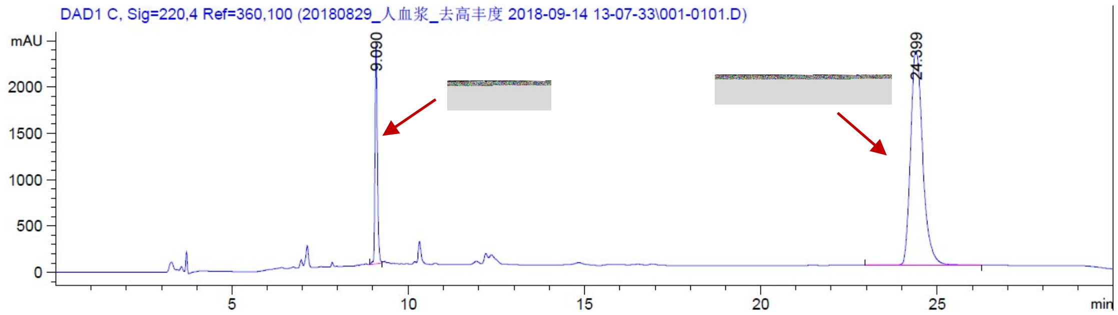

MALDI-TOF-MS/MS Spectra:

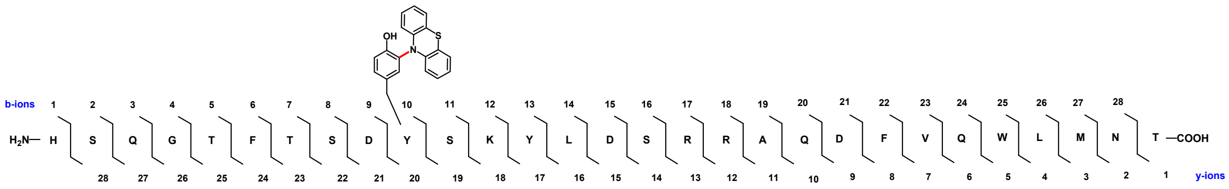

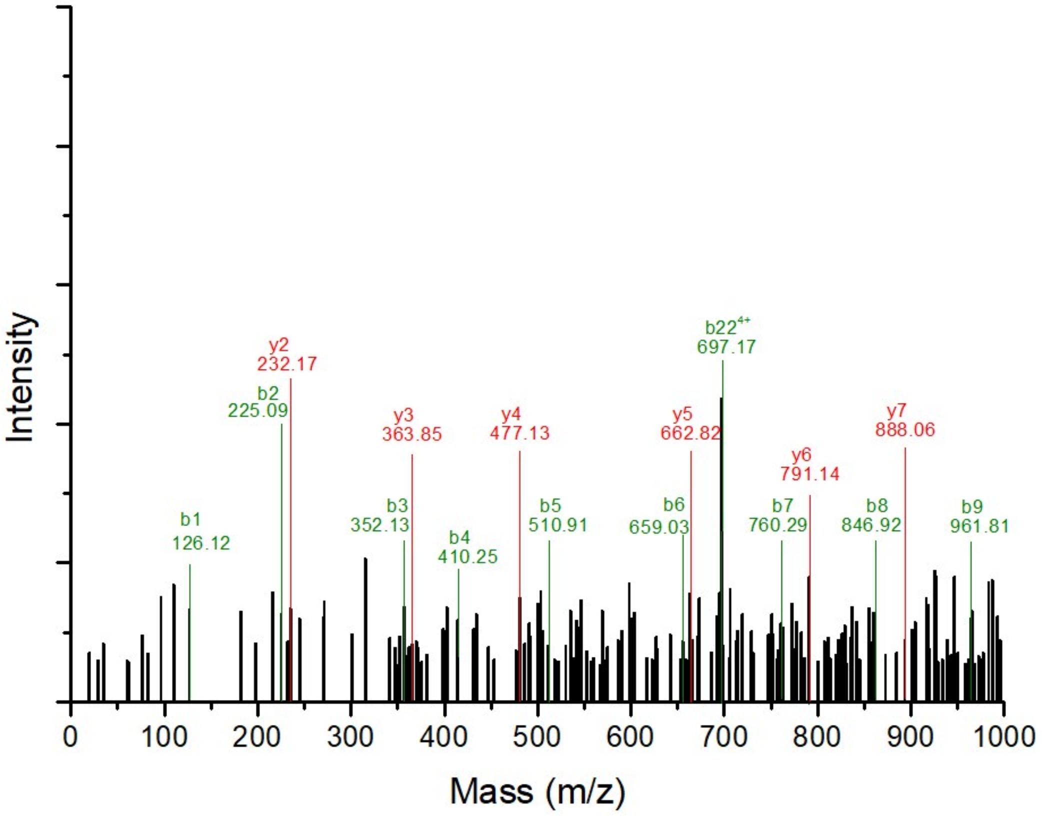
Intensity
Mass (m/z)
b1 126.12
b2 225.09
y2 232.17
b3 352.13
y3 363.85
b4 410.25
y4 477.13
b5 510.91
b6 659.03
y5 662.82
b22^{4+} 697.17
b7 760.29
y6 791.14
b8 846.92
y7 888.06
b9 961.81

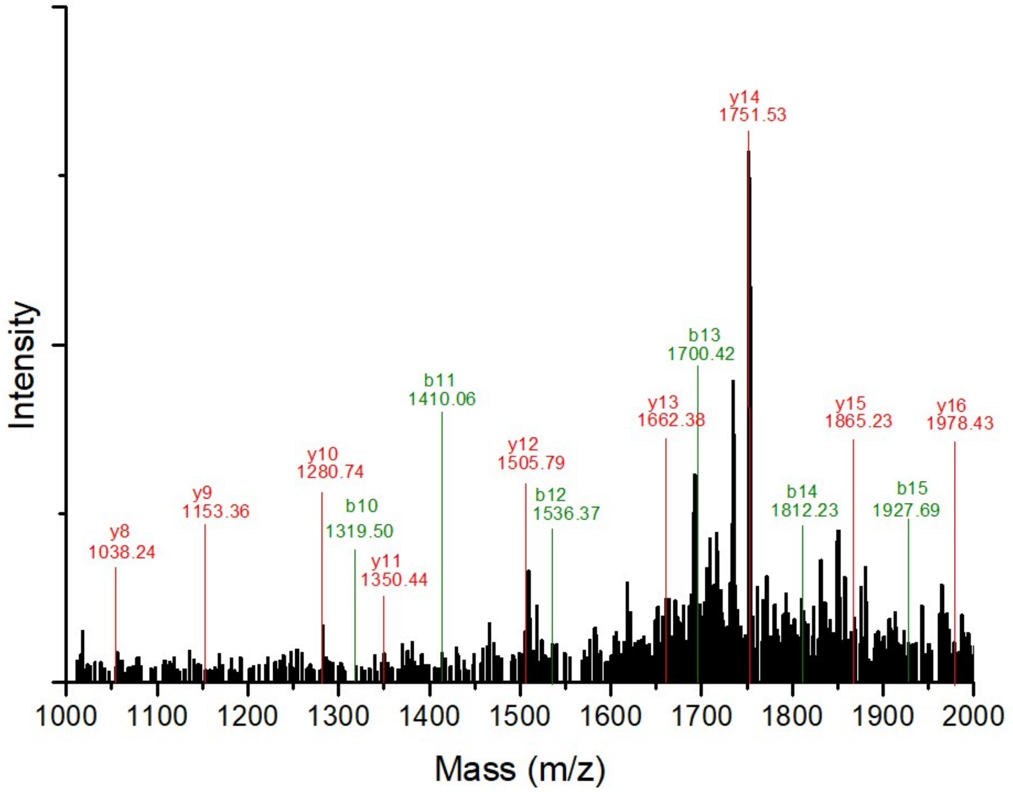
Intensity
Mass (m/z)
y8 1038.24
y9 1153.36
y10 1280.74
b10 1319.50
y11 1350.44
b11 1410.06
y12 1505.79
b12 1536.37
y13 1662.38
b13 1700.42
y14 1751.53
b14 1812.23
y15 1865.23
b15 1927.69
y16 1978.43

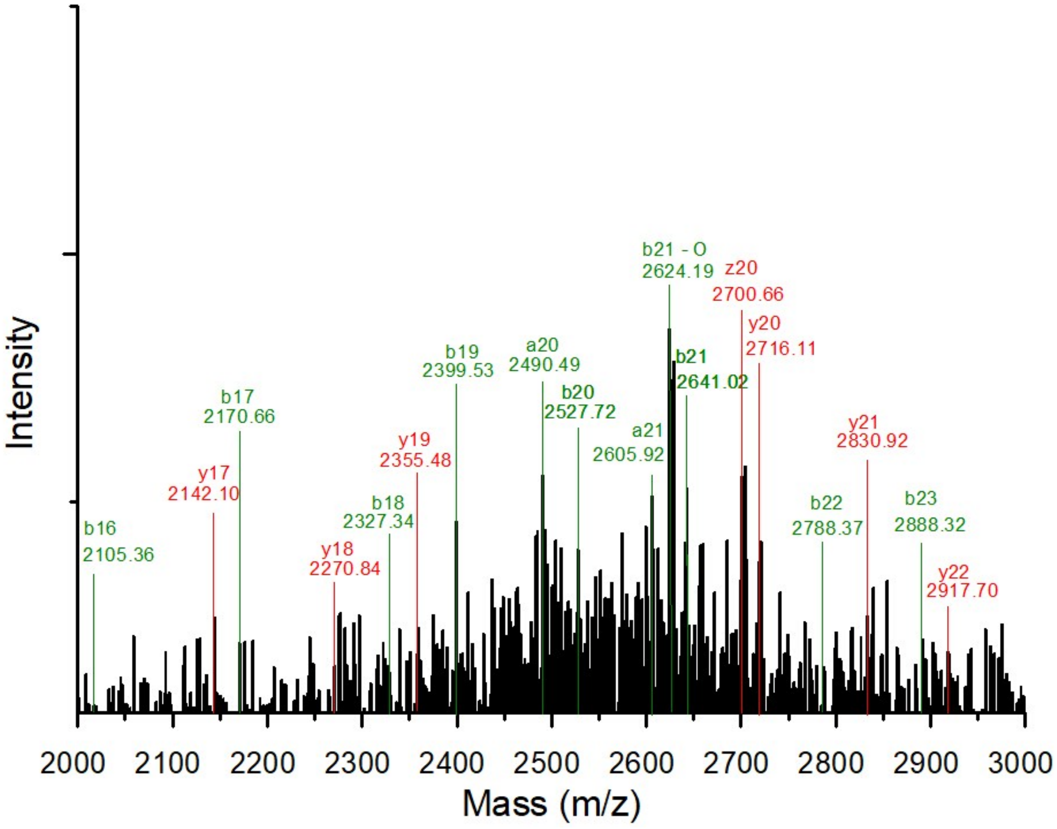
Intensity
b16
2105.36
y17
2142.10
b17
2170.66
y18
2270.84
b18
2327.34
y19
2355.48
b19
2399.53
a20
2490.49
b20
2527.72
a21
2605.92
b21 - O
2624.19
b21
2641.02
z20
2700.66
y20
2716.11
b22
2788.37
y21
2830.92
b23
2888.32
y22
2917.70
2000 2100 2200 2300 2400 2500 2600 2700 2800 2900 3000
Mass (m/z)

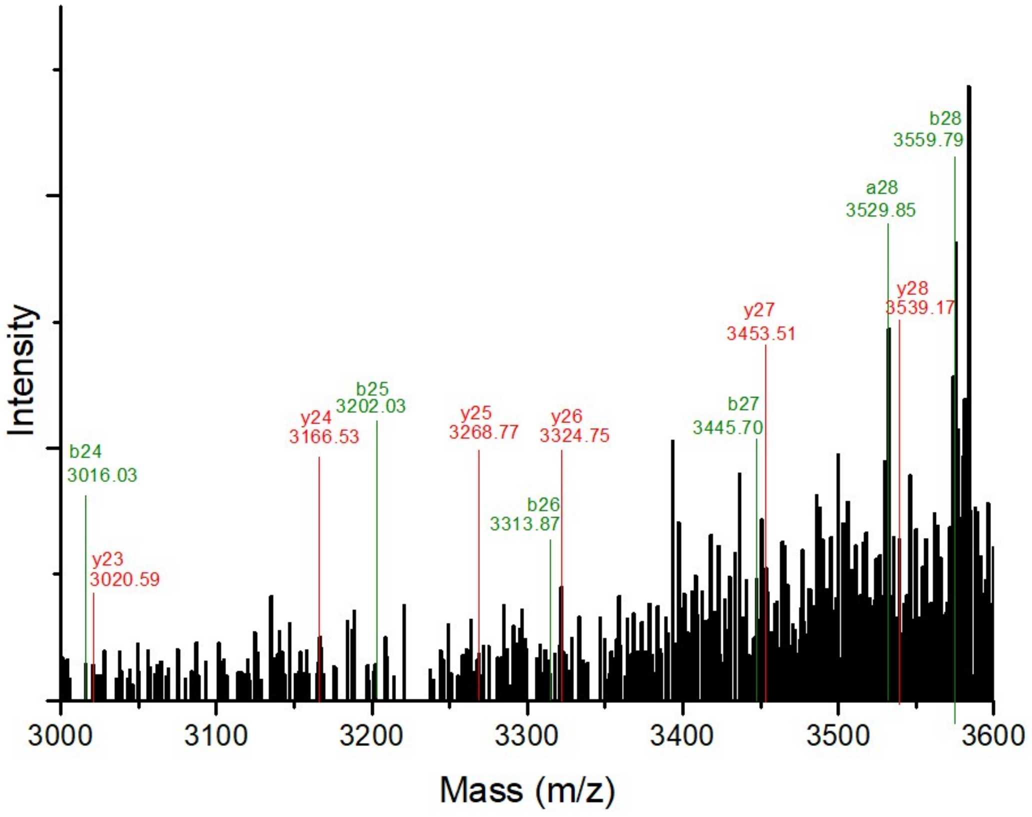
Intensity
b24
3016.03
y23
3020.59
y24
3166.53
b25
3202.03
y25
3268.77
b26
3313.87
y26
3324.75
b27
3445.70
y27
3453.51
a28
3529.85
y28
3539.17
b28
3559.79
3000 3100 3200 3300 3400 3500 3600
Mass (m/z)

DSIP 6i: WAGGDASGE-CONH$_2$

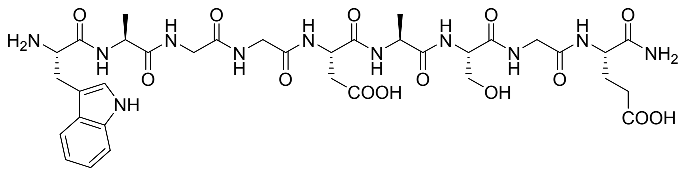

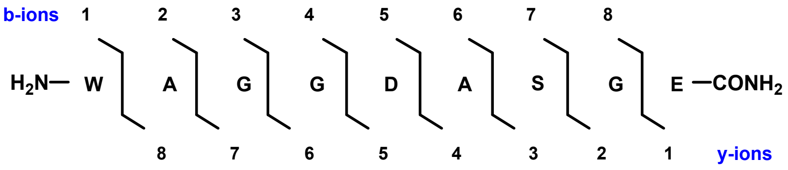

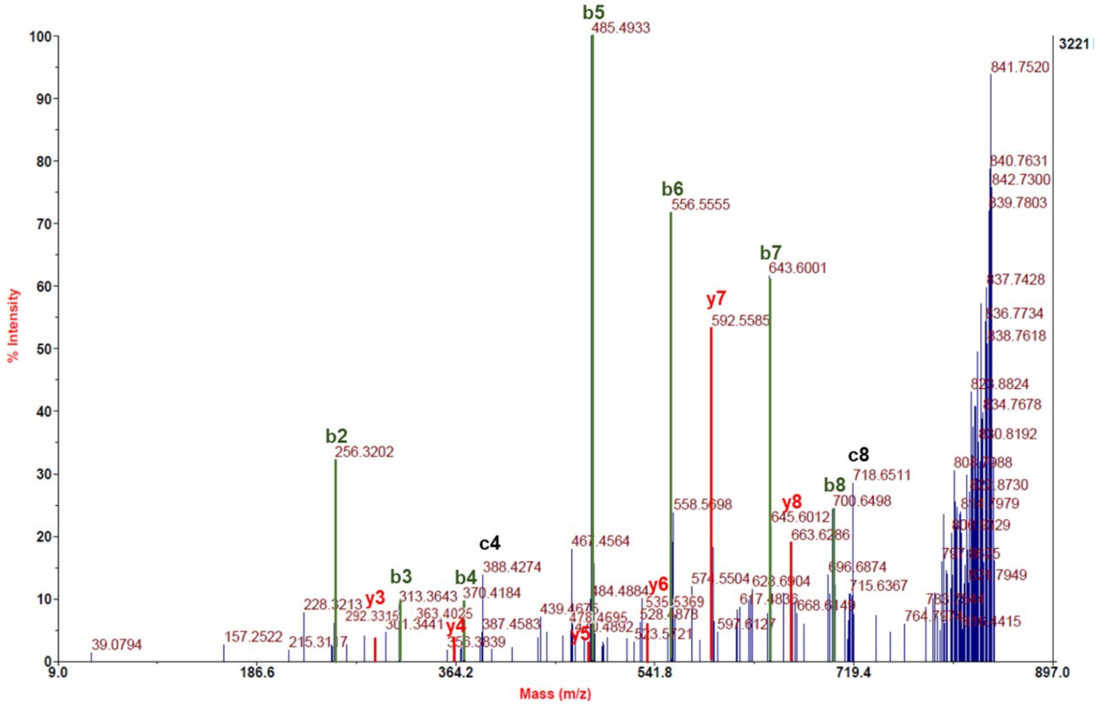

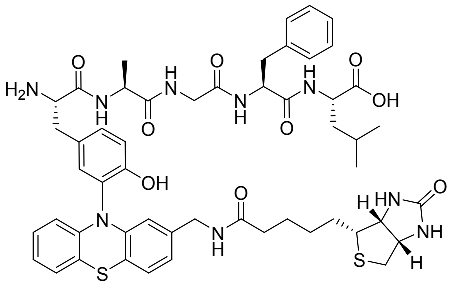

YAGFL-PTZ-Biotin 7i: Y(PTZ-Biotin)AGFL

HPLC: 56% conversion.

Product **7i** is a peak that elute at 50% buffer B (9:1 acetonitrile: water + 0.1% TFA) with retention times of 9.09 min. Reactant **6b** is a peak that elutes at 50% buffer B with a retention time of 7.98 min. Reactant PTZ-Probenecid is a peak that elutes at 50% buffer B with a retention time of 10.39 min. Diphenylamine was used as the standard.

HRMS (ESI-TOF) calcd for $C_{52}H_{63}N_9O_9S_2$, $[M+H]^+$, 1022.4263, found 1022.4273. Diphenylamine was used as the standard.

HPLC Spectra:

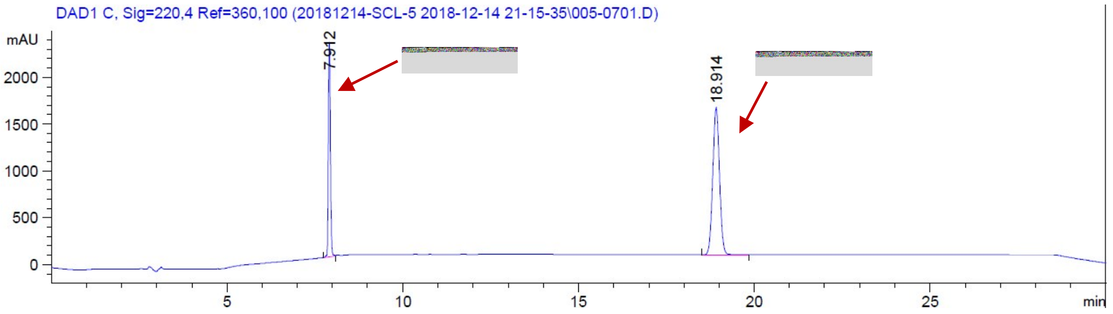

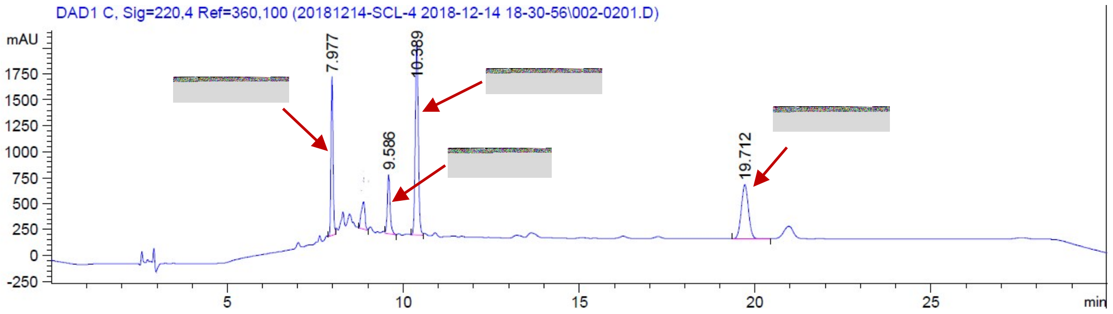

MALDI-TOF-MS/MS Spectra:

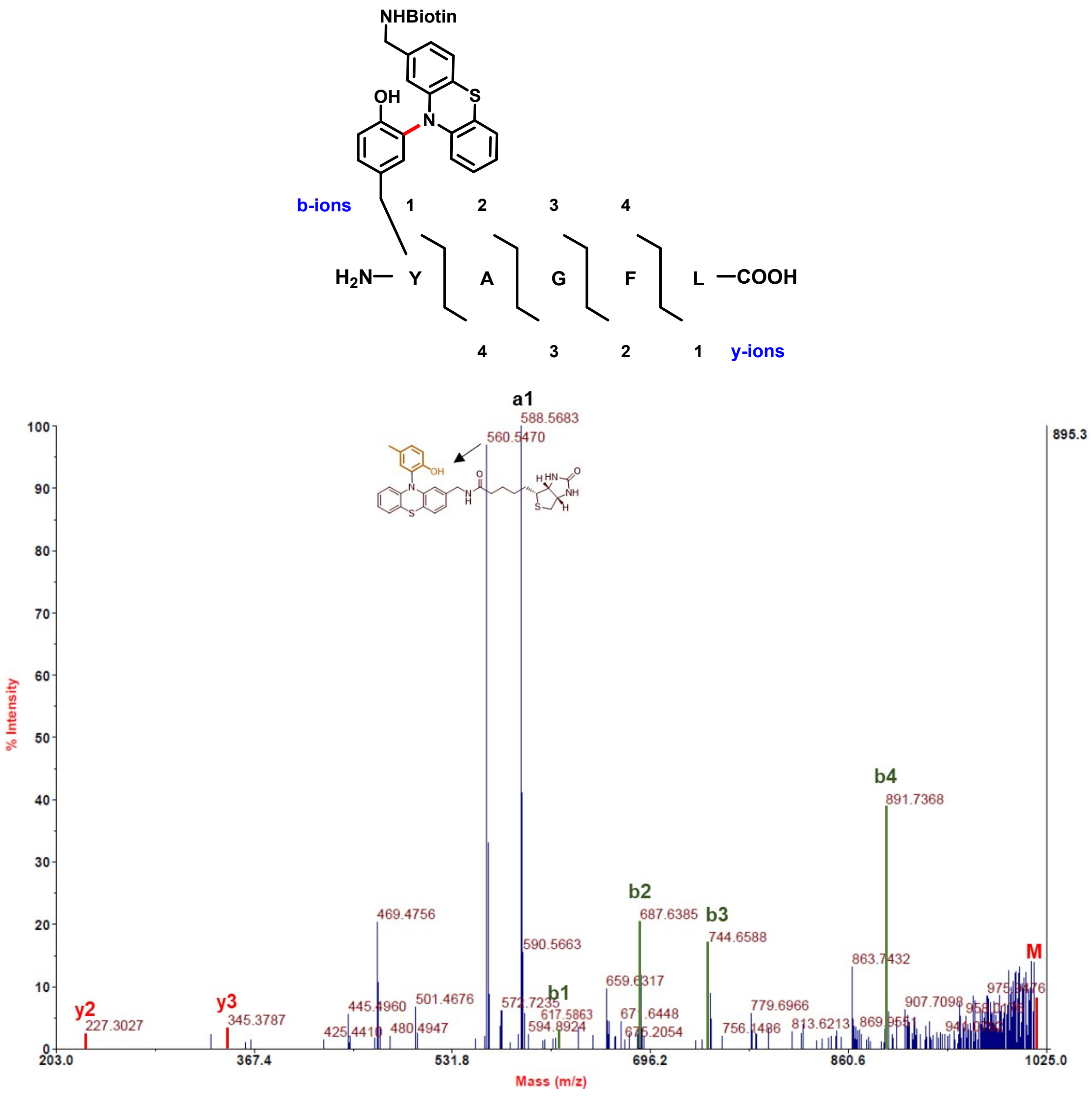
NHBiotin
OH
N
S
b-ions
1 2 3 4
H2N— Y A G F L —COOH
4 3 2 1 y-ions
a1
588.5683
560.5470
895.3
b4
891.7368
b2
687.6385
b3
744.6588
469.4756
590.5663
863.7432
M
659.6317
975.8476
y2
227.3027
y3
345.3787
445.4960
501.4676
572.7235
b1
617.5863
594.8924
779.6966
907.7098
425.4410
480.4947
756.1486
813.6213
869.9551
% Intensity
Mass (m/z)
203.0 367.4 531.8 696.2 860.6 1025.0

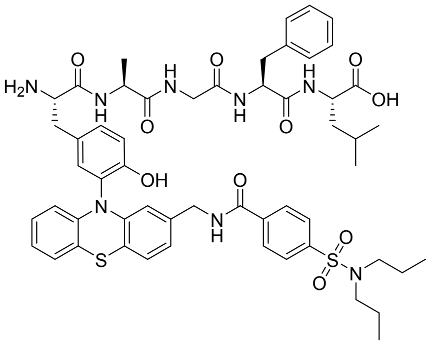

YAGFL-PTZ- Probenecid 7j: Y(PTZ- Probenecid)AGFL

HPLC: 50% conversion.

Product **7j** is a peak that elute at 50% buffer B (9:1 acetonitrile: water + 0.1% TFA) with retention times of 9.61 min. Reactant **6b** is a peak that elutes at 50% buffer B with a retention time of 7.97 min. Reactant PTZ-Probenecid is a peak that elutes at 50% buffer B with a retention time of 13.11 min. Diphenylamine was used as the standard.

HRMS (ESI-TOF) calcd for $C_{55}H_{66}N_8O_{10}S_2$, $[M+H]^+$, 1064.4416, found 1063.5520.

HPLC Spectra:

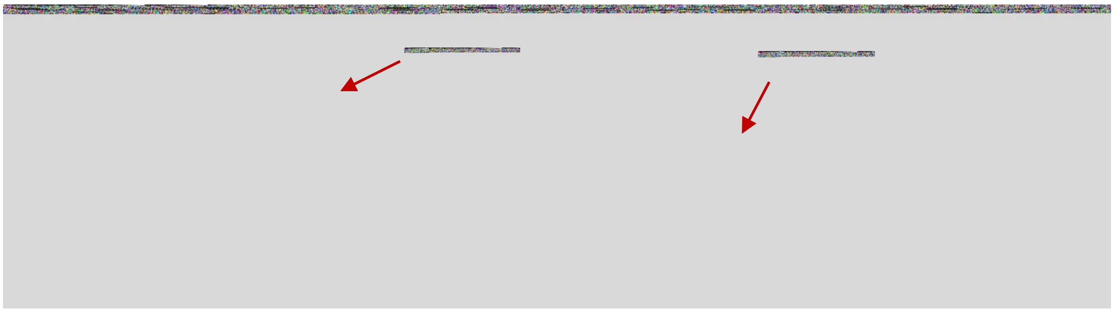

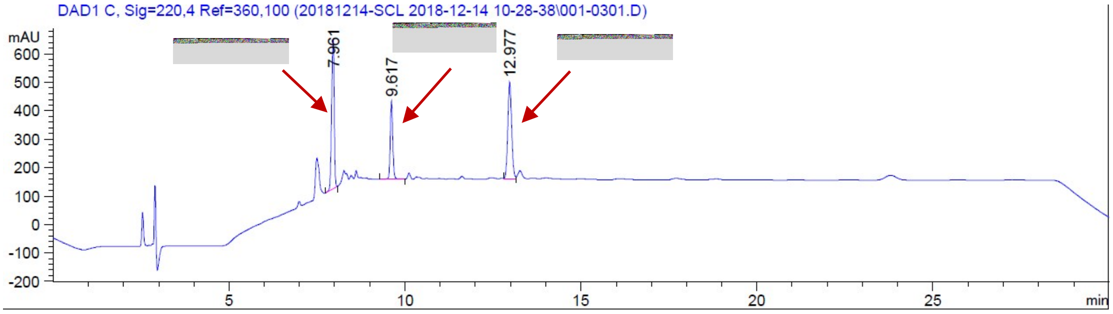

MALDI-TOF-MS/MS Spectra:

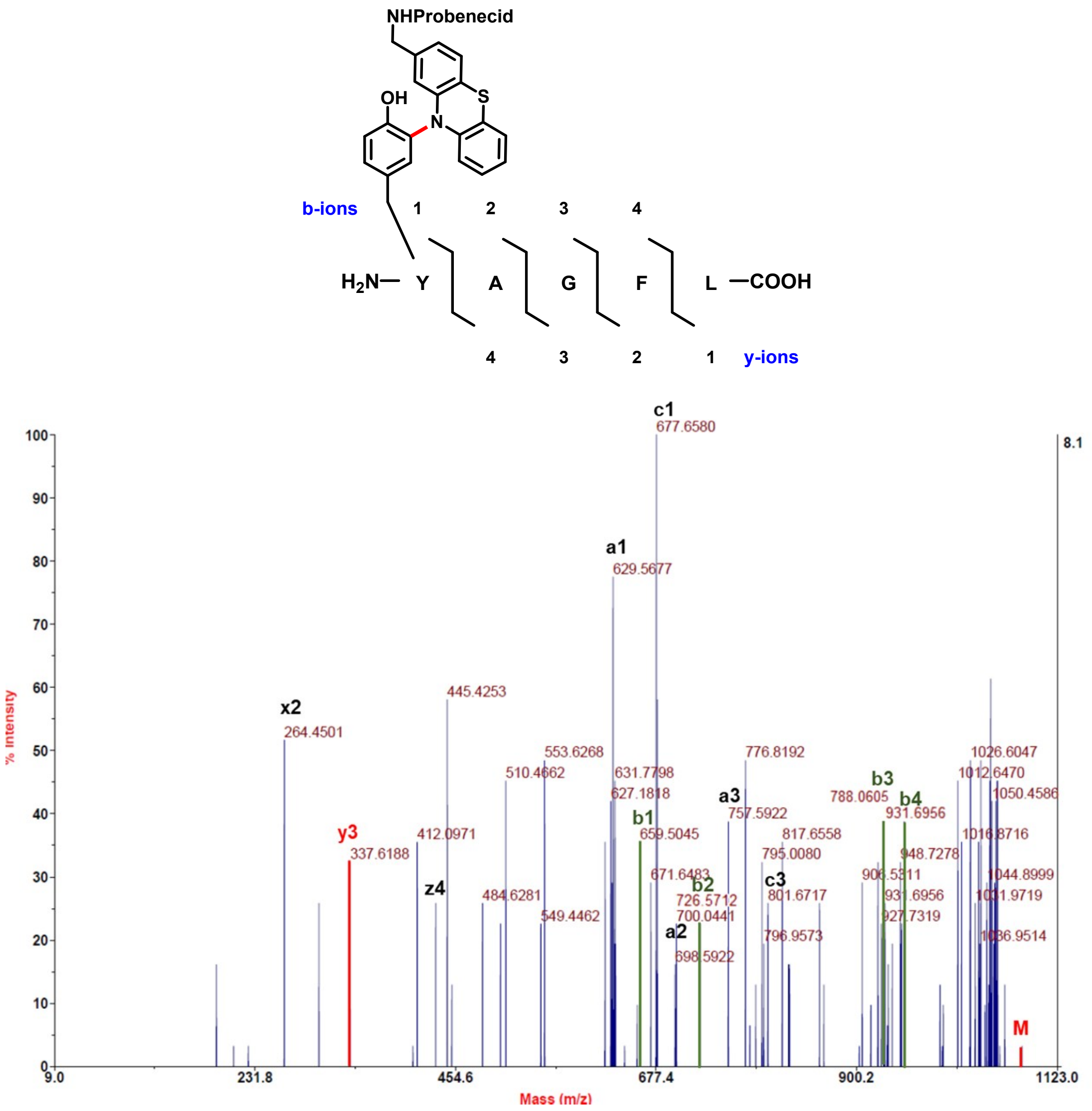
NHProbenecid
OH
S
N
b-ions
1
2
3
4
H2N— Y A G F L —COOH
4
3
2
1
y-ions
% Intensity
Mass (m/z)
c1
677.6580
a1
629.5677
x2
264.4501
445.4253
553.6268
510.4662
631.7798
627.1818
776.8192
1026.6047
1012.6470
1050.4586
b3
b4
788.0605
931.6956
a3
757.5922
b1
659.5045
817.6558
y3
337.6188
412.0971
795.0080
948.7278
1016.8716
906.5311
1044.8999
z4
484.6281
671.6483
b2
726.5712
c3
801.6717
931.6956
1031.9719
549.4462
700.0441
927.7319
a2
796.9573
1036.9514
698.5922
M
8.1
9.0
231.8
454.6
677.4
900.2
1123.0

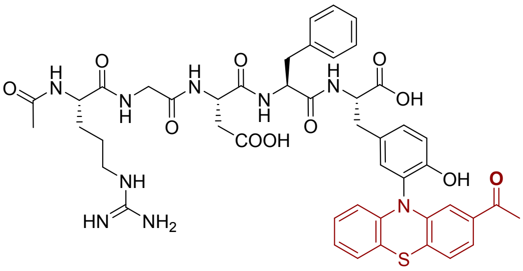

Chain RGD 7k: Ac-RGDFY(PTZ-Ac)

HPLC: 99% conversion.

Product **7k** is a peak that elute at 50% buffer B (9:1 acetonitrile: water + 0.1% TFA) with retention times of 9.82 min. Reactant **6a** is a peak that elutes at 50% buffer B with a retention time of 6.82 min. Anisic acid was used as the standard.

HRMS (ESI-TOF) calcd for $C_{46}H_{51}N_9O_{11}S$, $[M+H]^+$, 938.3502, found 938.3533.

HPLC Spectra:

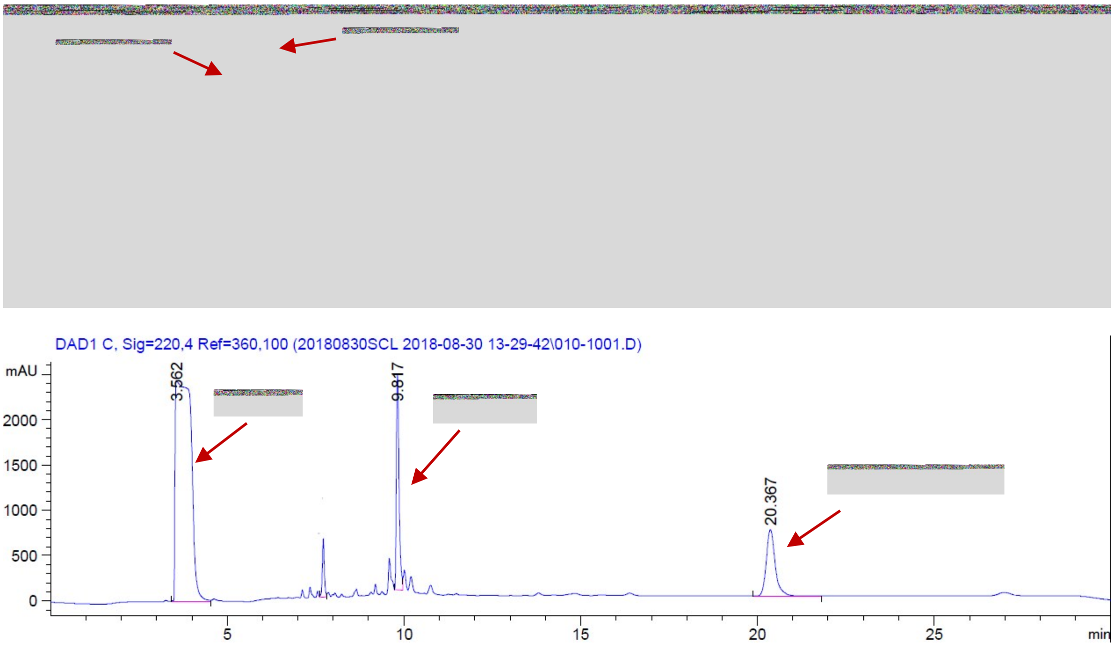

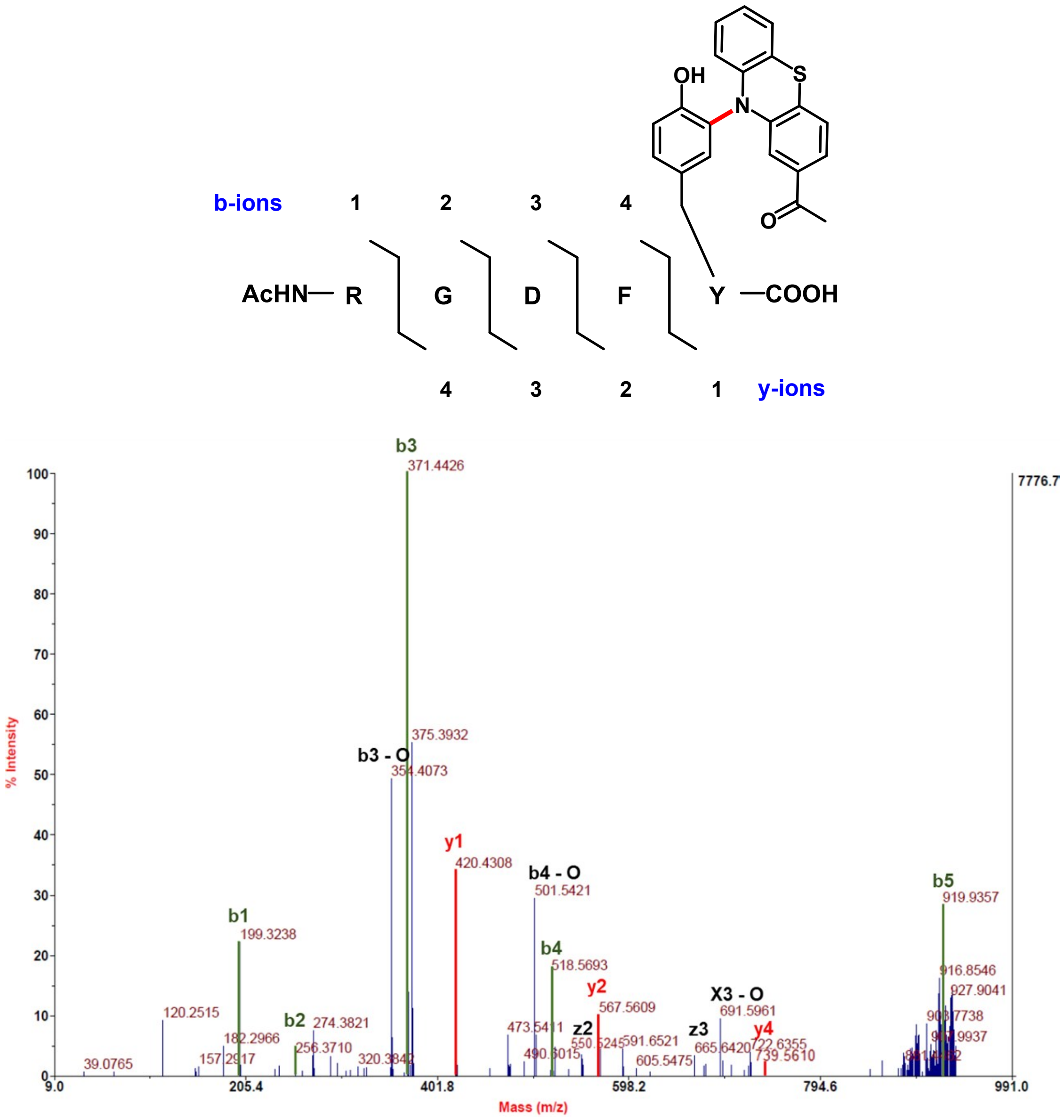

3.8 Protein scope and characterization

3.8.1 General procedure for bioconjugation of pig insulin and phenothiazine: In an oven-dried undivided three-necked bottle (15 mL) equipped with a stir bar, pig insulin (5 mg), phenothiazine (10 mg), CH_3CN (0.75 mL) and phosphate buffer solution (0.75 mL, pH= 7.3) were combined and added. The bottle was equipped graphite rod (ϕ 6 mm, about 15 mm immersion depth in solution) as the anode and nickel plate (7.5 mm×15 mm×1 mm) as the cathode and then charged with nitrogen. The reaction mixture was stirred and electrolyzed at constant current of 10 mA ($j_{anode} \approx 11$ mA/cm^2) under room temperature for 30 min. After completion of the reaction, the solution was

analyzed by MALDI-TOF MS spectroscopy.

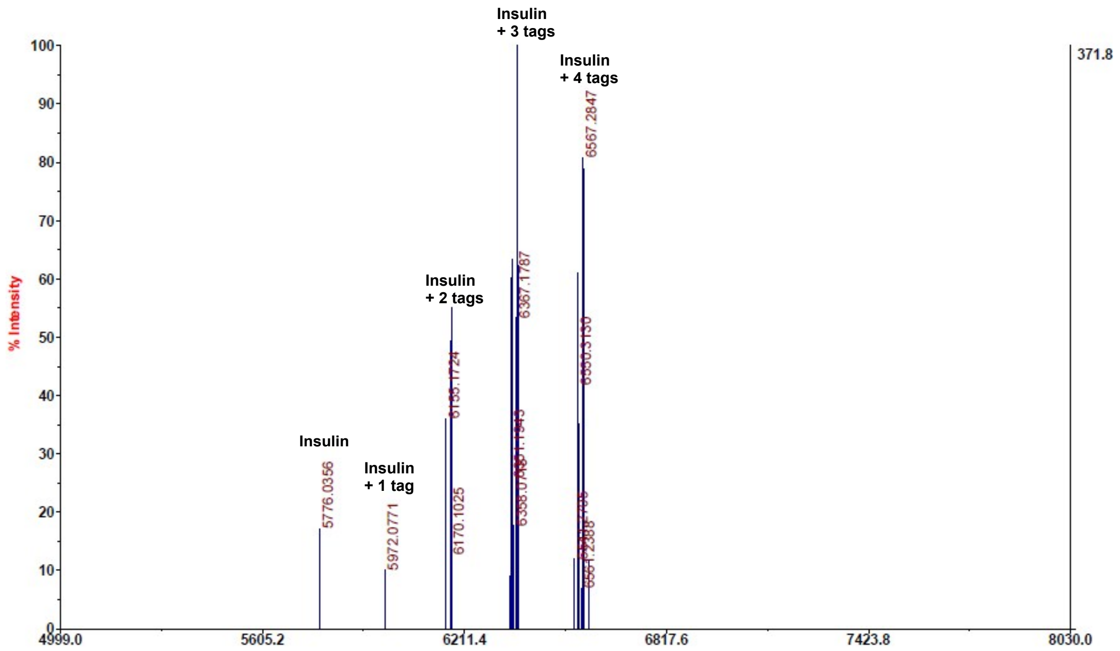

Pig insulin

H-G-I-V-E-Q-C-C-T-S-I-C-S-L-Y-Q-L-E-N-Y-C-N-OH

H-F-V-N-Q-H-L-C-G-S-H-L-V-E-A-L-Y-L-V-C-G-E-R-G-F-F-Y-T-P-K-A-OH

Control experiment 1 (no electric current): In an oven-dried undivided three-necked bottle (15 mL) equipped with a stir bar, pig insulin (5 mg), phenothiazine (10 mg), CH_3CN (0.75 mL) and phosphate buffer solution (0.75 mL, pH= 7.3) were combined and added. The bottle was equipped graphite rod (ϕ 6 mm, about 15 mm immersion depth in solution) as the anode and nickel plate (7.5 mm×15 mm×1 mm) as the cathode and then charged with nitrogen. The reaction mixture was stirred under room temperature for 30 min. After completion of the reaction, the solution was analyzed by MALDI-TOF MS spectroscopy, which shows a great conversion of phenothiazine-conjugated reaction.

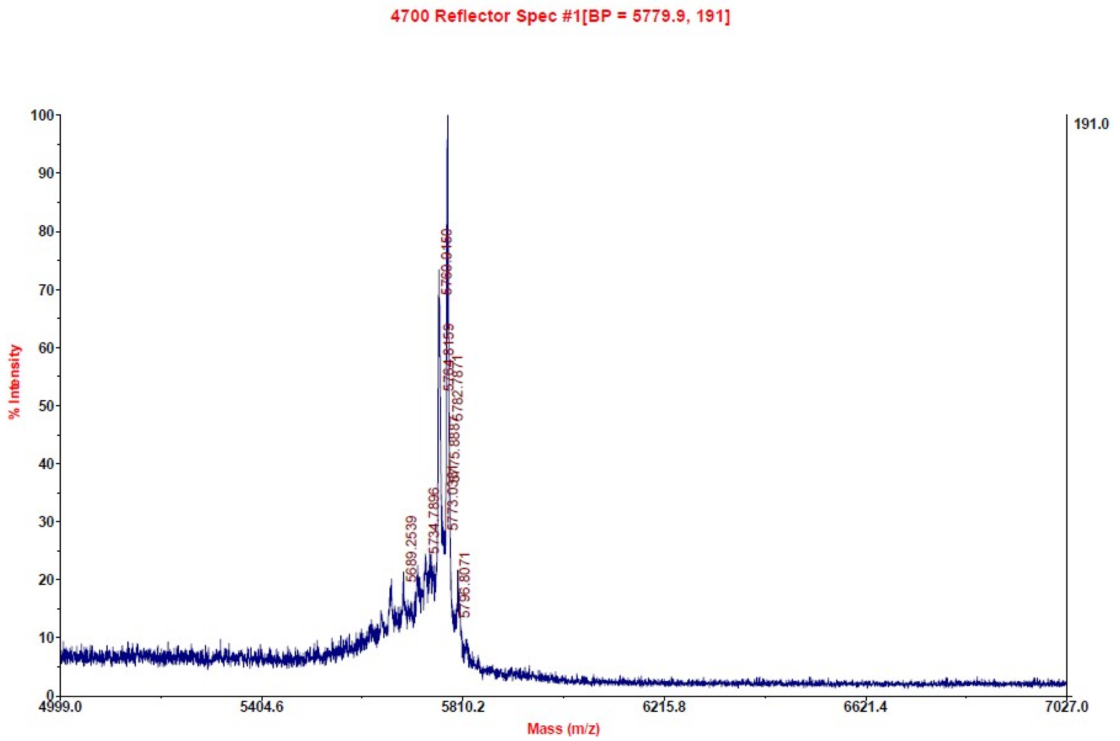

Control experiment 2 (no phenothiazine): In an oven-dried undivided three-necked bottle (15 mL) equipped with a stir bar, pig insulin (5 mg), CH_3CN (0.75 mL) and phosphate buffer solution (0.75 mL, pH= 7.3) were combined and added. The bottle was equipped graphite rod (ϕ 6 mm, about 15 mm immersion depth in solution) as the anode and nickel plate (7.5 mm×15 mm×1 mm) as the cathode and then charged with nitrogen. The reaction mixture was stirred and electrolyzed at constant current of 10 mA (j_{anode} ≈ 11 mA/cm^2) under room temperature for 30 min. After completion of the reaction, the solution was analyzed by MALDI-TOF MS spectroscopy.

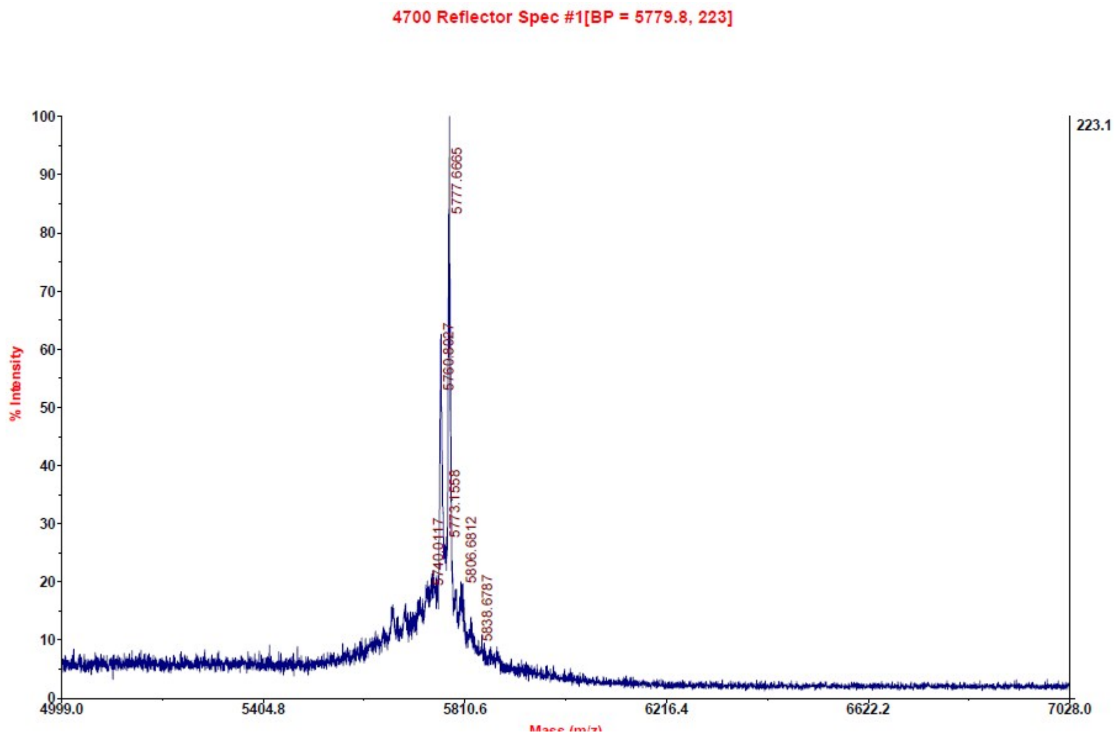

Effect of electrolytic arylamination on structure of insulin

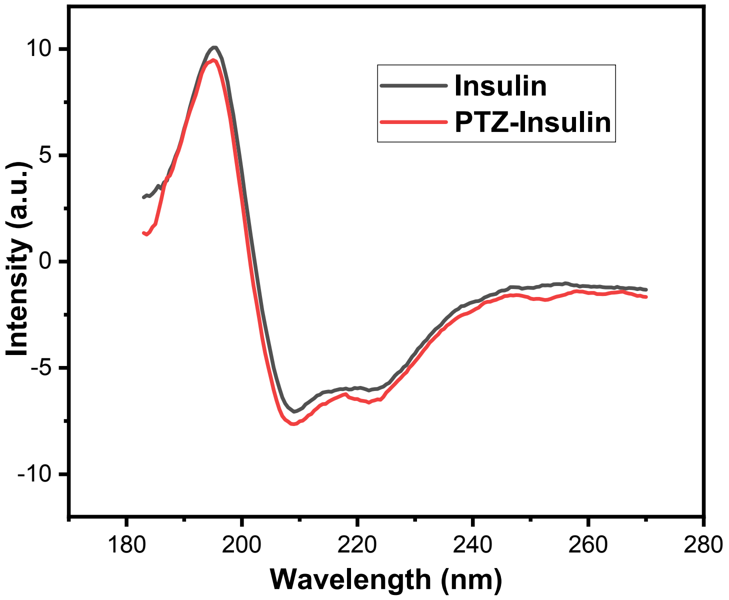

Comparison of CD spectra between Insulin and PTZ-Insulin sample (100 µg/mL in PBS buffer).

3.8.2 General procedure for bioconjugation of myoglobin and phenothiazine: In an oven-dried undivided three-necked bottle (15 mL) equipped with a stir bar, myoglobin (from horse heart) (5 mg), phenothiazine (20 mg), CH_3CN (0.75 mL) and

phosphate buffer solution (0.75 mL, pH= 7.3) were combined and added. The bottle was equipped graphite rod (ϕ 6 mm, about 15 mm immersion depth in solution) as the anode and nickel plate (7.5 mm×15 mm×1 mm) as the cathode and then charged with nitrogen. The reaction mixture was stirred and electrolyzed at constant current of 0.5 mA under -20°C for 15 min. After completion of the reaction, the solution was analyzed by LC-MS analysis. The found mass are shown on the top of deconvoluted peaks. The deconvoluted MS data of full protein peaks are plotted in the following figures. The major peaks correspond to myoglobin carrying one phenothiazine modification. For myoglobin: expected mass 16951.5 Da, found 16951.5 Da. For singly-modified myoglobin: expected mass 17148.5 Da, found 17148.5 Da.

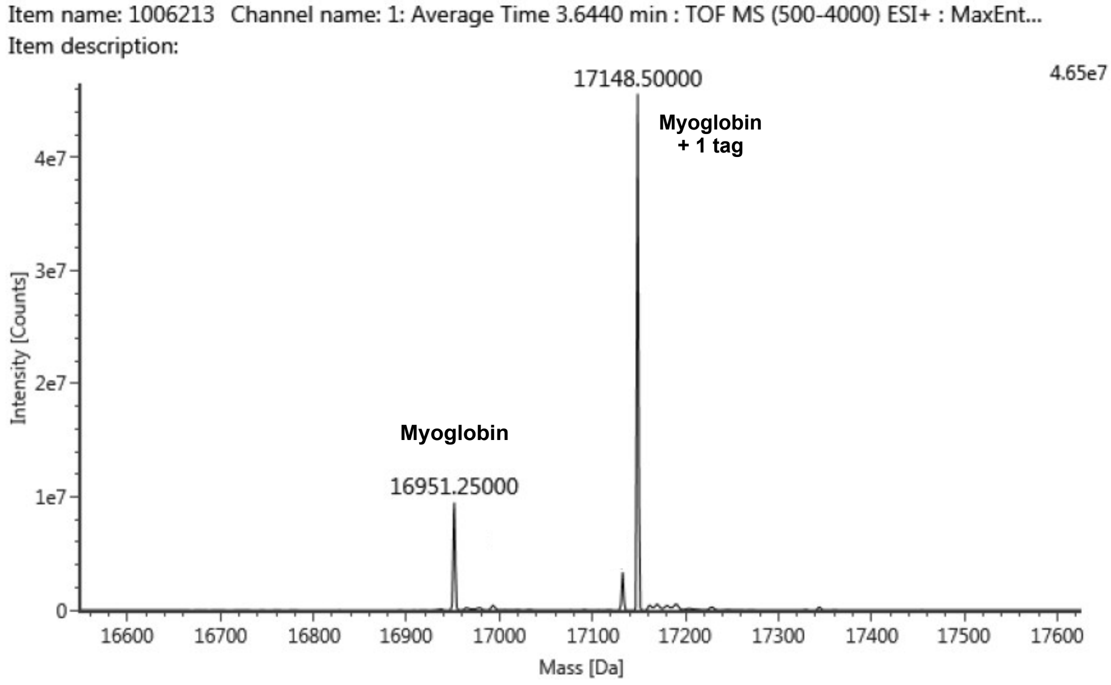

Effect of electrolytic arylamination on structure of myoglobin

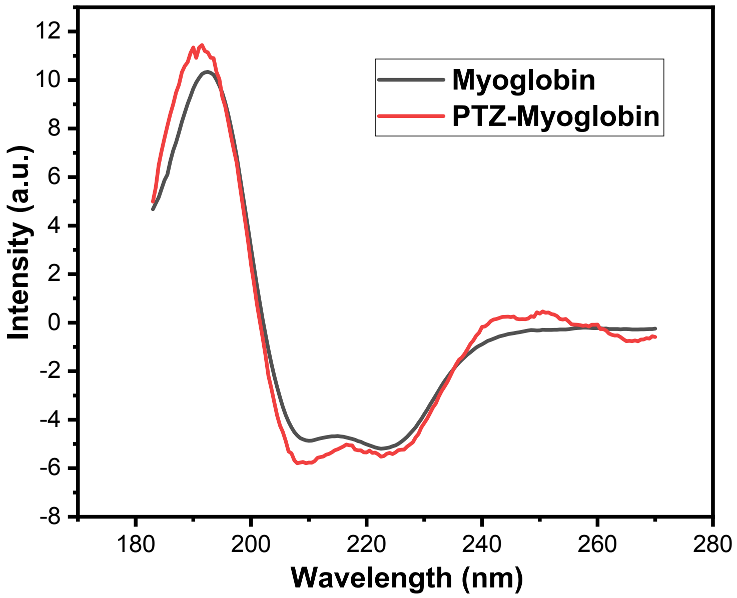

Comparison of CD spectra between Myoglobin and PTZ-Myoglobin sample (100 µg/mL in PBS buffer).

4. References

(1) E. Pedroso, A. Grandas, M. D. Ludevid, E. Giralt, Determination of the preferred tautomeric form of 4-nitrohistidine. *J. Heterocyclic Chem*. **1986**, *23*, 921–924.

(2) R. A. Serwa, J.-M. Swiecicki, D. Homann, C. P. R. Hackenberger, Phosphoramidate-peptide synthesis by solution- and solid-phase Staudinger-phosphite reactions. *J. Pept. Sci.* **2010**, *16*, 563–567.

(3) M. J. Ohlow, S. Sohre, M. Granold, M. Schreckenberger, B. Moosmann, Why have clinical trials of antioxidants to prevent neurodegeneration failed? - A cellular investigation of novel phenothiazine-type antioxidants reveals competing objectives for pharmaceutical neuroprotection. *Pharm. Res.* **2017**, *34*, 378-393.

(4) M. Di Antonio, G. Biffi, A. Mariani, E. A. Raiber, R. Rodriguez, S. Balasubramanian, Selective RNAVersus DNA G-Quadruplex Targeting by In Situ Click Chemistry. *Angew. Chem. Int. Ed.* **2012**, *51*, 11073-11078.

(5) I. A. Inverarity, R. F. H. Viguier, P. Cohen, A. N. Hulme, Biotinylated Anisomycin, A Comparison of Classical and "Click" Chemistry Approaches. *Bioconj. Chem.* **2007**, *18*, 1593–1603.

5. Spectra

5.1 NMR Spectra of Products

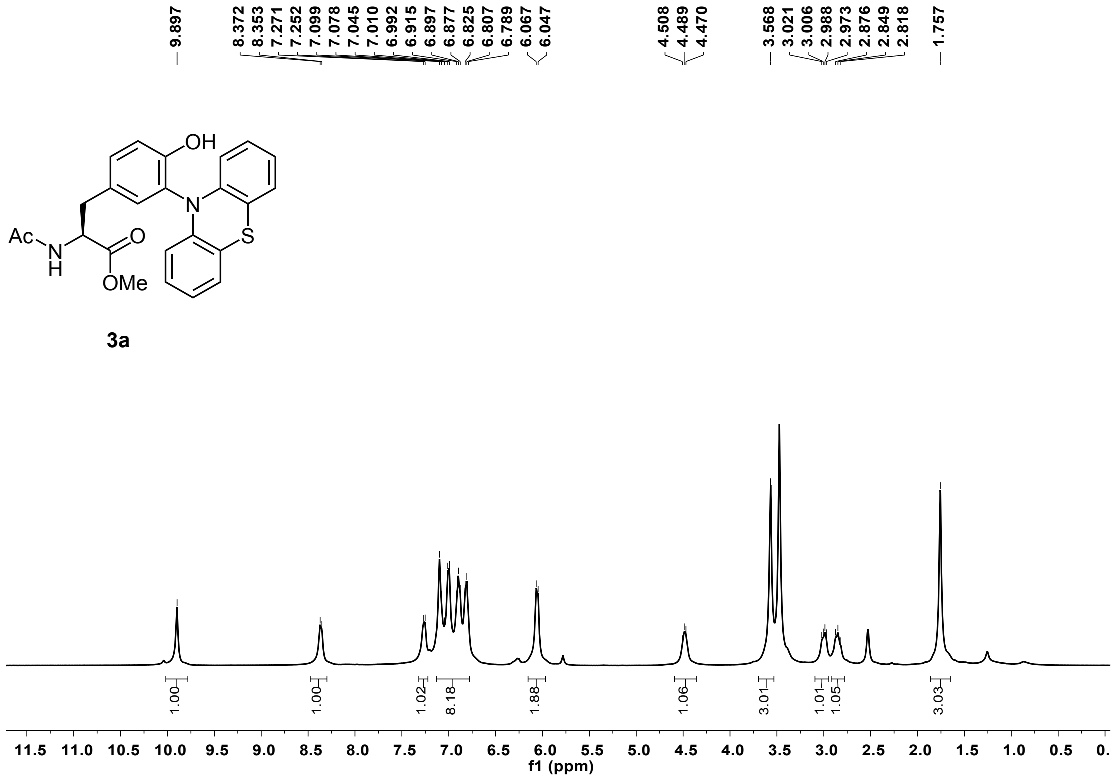

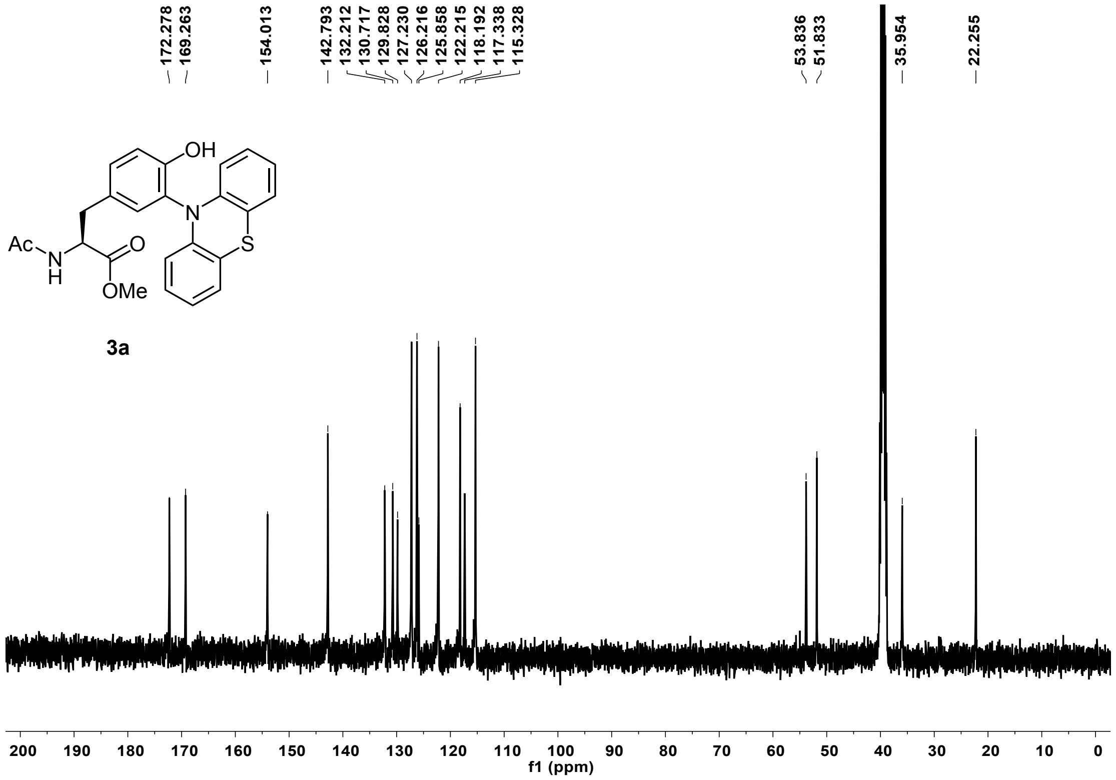

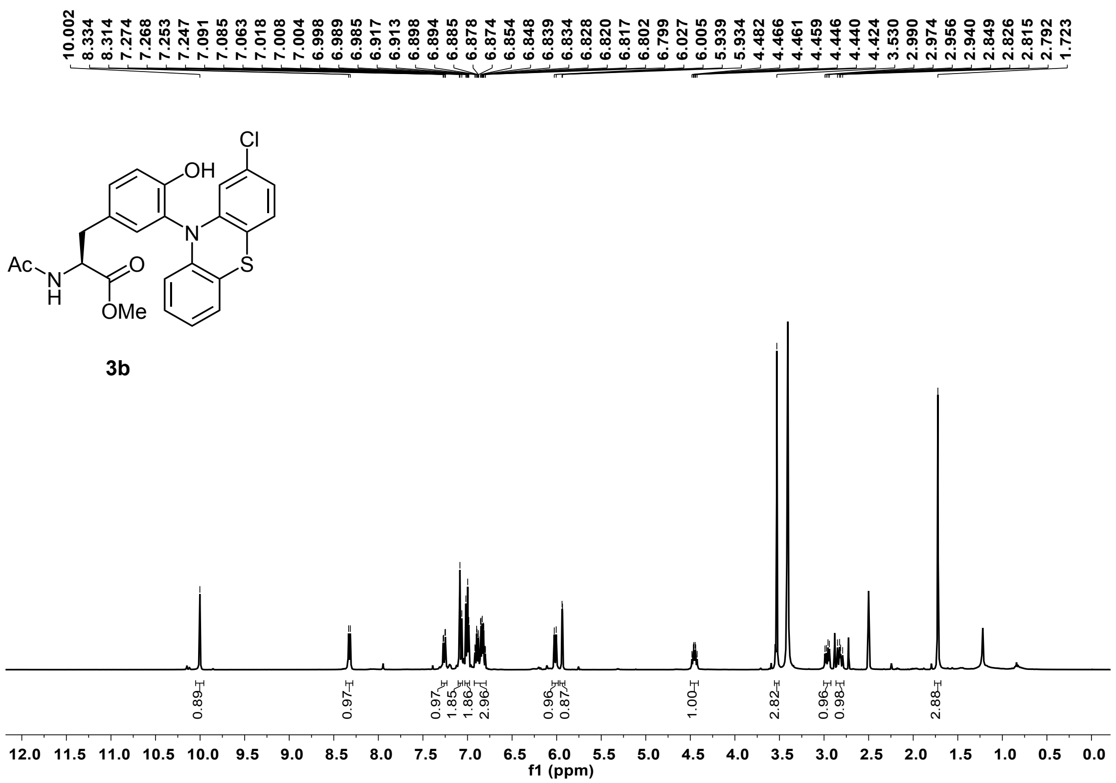
10.002 8.334 8.314 7.274 7.268 7.253 7.247 7.091 7.085 7.063 7.018 7.008 7.004 6.998 6.989 6.985 6.917 6.913 6.898 6.894 6.885 6.878 6.874 6.854 6.848 6.839 6.834 6.828 6.820 6.817 6.802 6.799 6.027 6.005 5.939 5.934 4.482 4.466 4.461 4.459 4.446 4.440 4.424 3.530 2.990 2.974 2.956 2.940 2.849 2.826 2.815 2.792 1.723
Cl
OH
N
S
Ac
N
H
O
OMe
3b
0.89
0.97
0.97
1.85
1.86
2.96
0.96
0.87
1.00
2.82
0.96
0.98
2.88
12.0 11.5 11.0 10.5 10.0 9.5 9.0 8.5 8.0 7.5 7.0 6.5 6.0 5.5 5.0 4.5 4.0 3.5 3.0 2.5 2.0 1.5 1.0 0.5 0.0
f1 (ppm)

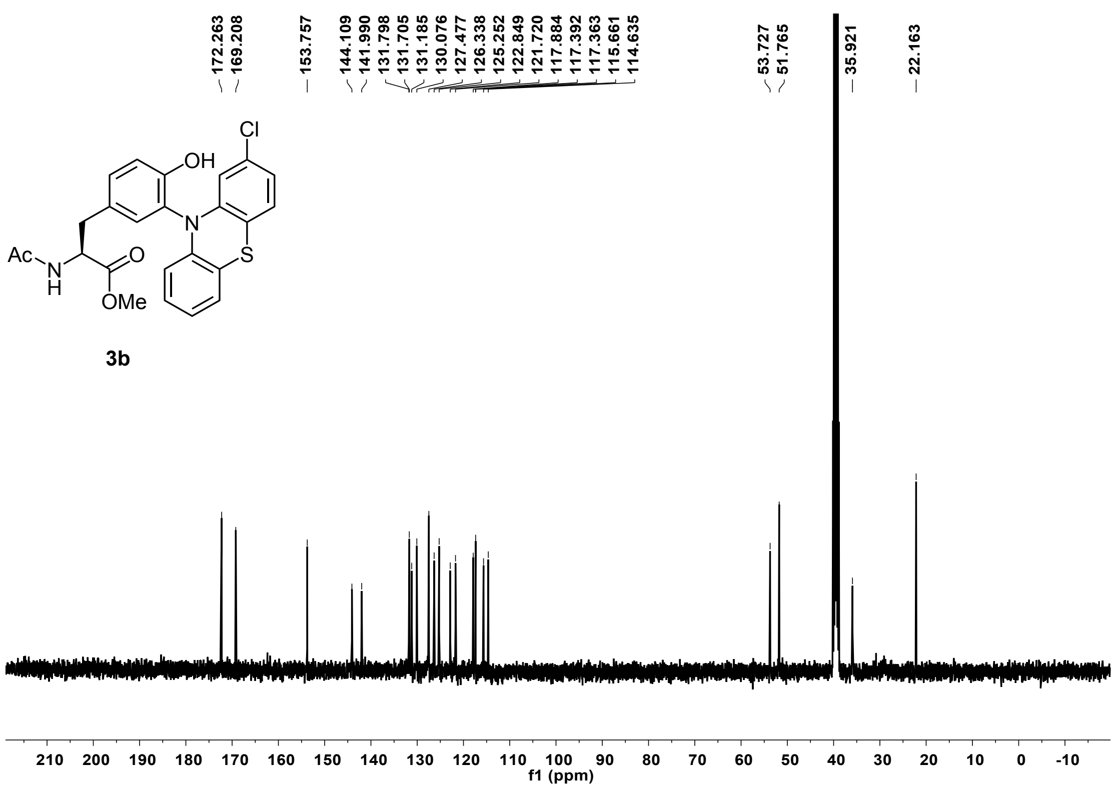
172.263 169.208 153.757 144.109 141.990 131.798 131.705 131.185 130.076 127.477 126.338 125.252 122.849 121.720 117.884 117.392 117.363 115.661 114.635 53.727 51.765 35.921 22.163
Cl
OH
N
S
Ac
N
H
O
OMe
3b
210 200 190 180 170 160 150 140 130 120 110 100 90 80 70 60 50 40 30 20 10 0 -10
f1 (ppm)

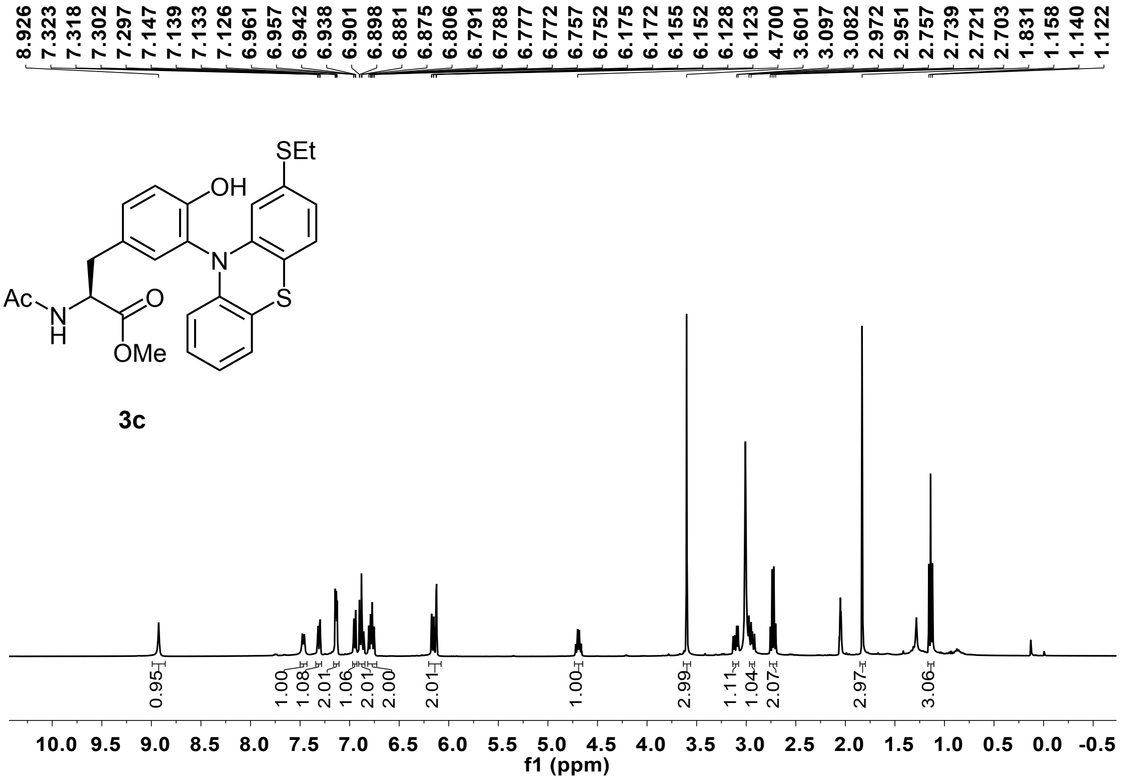
8.926 7.323 7.318 7.302 7.297 7.147 7.139 7.133 7.126 6.961 6.957 6.942 6.938 6.901 6.898 6.881 6.875 6.806 6.791 6.788 6.777 6.772 6.757 6.752 6.175 6.172 6.155 6.152 6.128 6.123 4.700 3.601 3.097 3.082 2.972 2.951 2.757 2.739 2.721 2.703 1.831 1.158 1.140 1.122
SEt
OH
N
S
Ac
N
H
O
OMe
3c
0.95 1.00 1.08 2.01 1.06 2.01 2.00 2.01 1.00 2.99 1.11 1.04 2.07 2.97 3.06
10.0 9.5 9.0 8.5 8.0 7.5 7.0 6.5 6.0 5.5 5.0 4.5 4.0 3.5 3.0 2.5 2.0 1.5 1.0 0.5 0.0 -0.5
f1 (ppm)

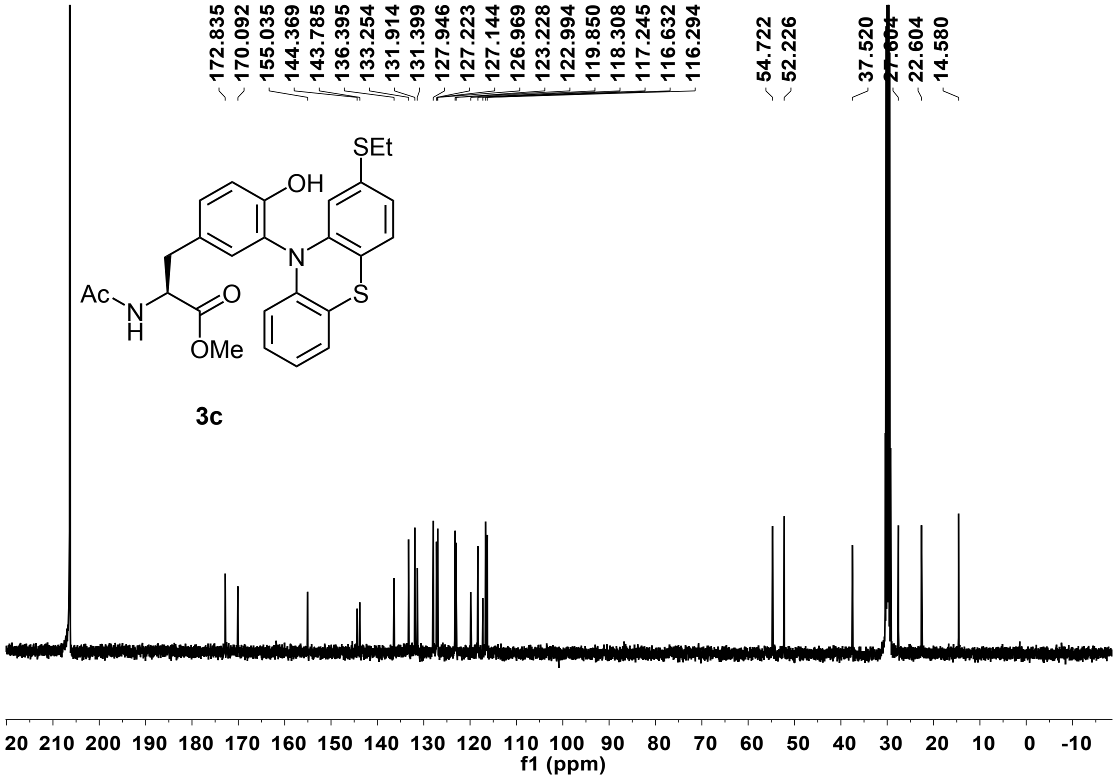
172.835 170.092 155.035 144.369 143.785 136.395 133.254 131.914 131.399 127.946 127.223 127.144 126.969 123.228 122.994 119.850 118.308 117.245 116.632 116.294
54.722 52.226
37.520 22.604 14.580
SEt
OH
N
S
Ac
N
H
O
OMe
3c
20 210 200 190 180 170 160 150 140 130 120 110 100 90 80 70 60 50 40 30 20 10 0 -10
f1 (ppm)

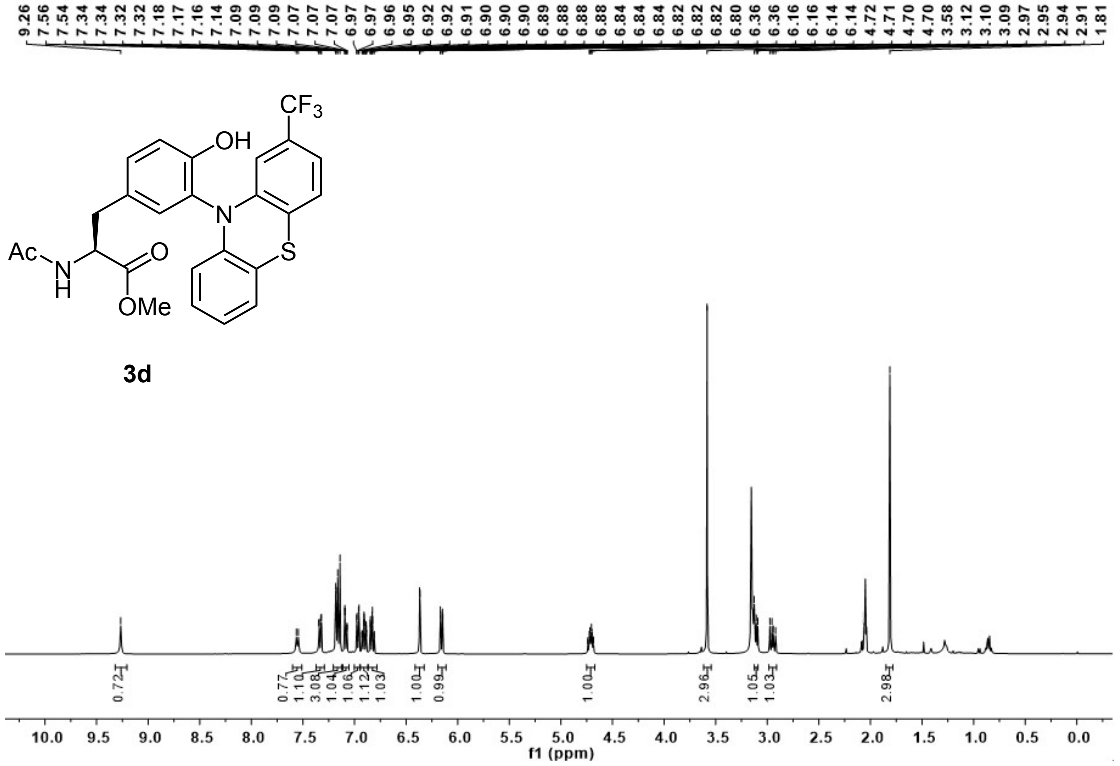
9.26 7.56 7.54 7.34 7.34 7.32 7.32 7.18 7.17 7.16 7.14 7.09 7.09 7.09 7.07 7.07 7.07 6.97 6.97 6.96 6.95 6.92 6.92 6.91 6.90 6.90 6.89 6.88 6.88 6.88 6.84 6.84 6.84 6.82 6.82 6.82 6.80 6.36 6.36 6.16 6.16 6.14 6.14 4.72 4.71 4.70 4.70 3.58 3.12 3.10 3.09 2.97 2.95 2.94 2.91 1.81
CF3
OH
N
S
Ac
N
H
O
OMe
3d
0.72 0.77 1.10 3.08 1.04 1.06 1.12 1.03 1.00 0.99 1.00 2.96 1.05 1.03 2.98
10.0 9.5 9.0 8.5 8.0 7.5 7.0 6.5 6.0 5.5 5.0 4.5 4.0 3.5 3.0 2.5 2.0 1.5 1.0 0.5 0.0
f1 (ppm)

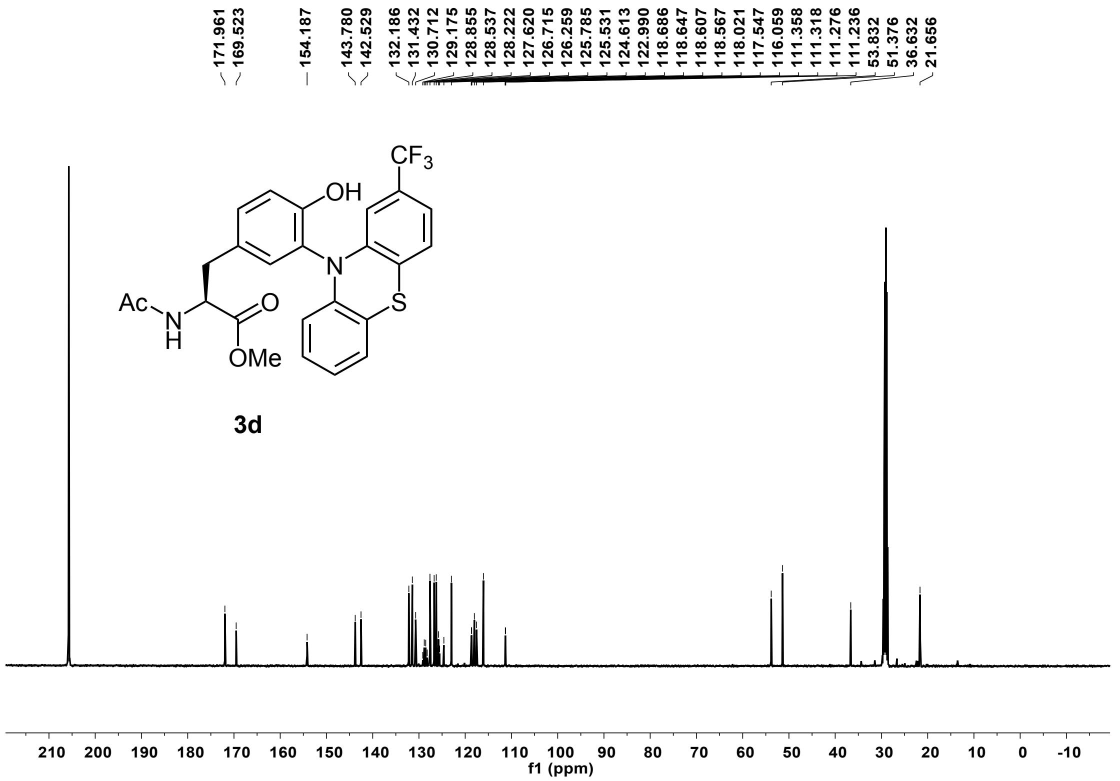
171.961 169.523 154.187 143.780 142.529 132.186 131.432 130.712 129.175 128.855 128.537 128.222 127.620 126.715 126.259 125.785 125.531 124.613 122.990 118.686 118.647 118.607 118.567 118.021 117.547 116.059 111.358 111.318 111.276 111.236 53.832 51.376 36.632 21.656
CF3
OH
N
S
Ac
N
H
O
OMe
3d
210 200 190 180 170 160 150 140 130 120 110 100 90 80 70 60 50 40 30 20 10 0 -10
f1 (ppm)

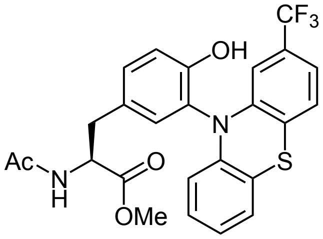
CF3
OH
N
S
Ac
N
H
O
OMe

3d

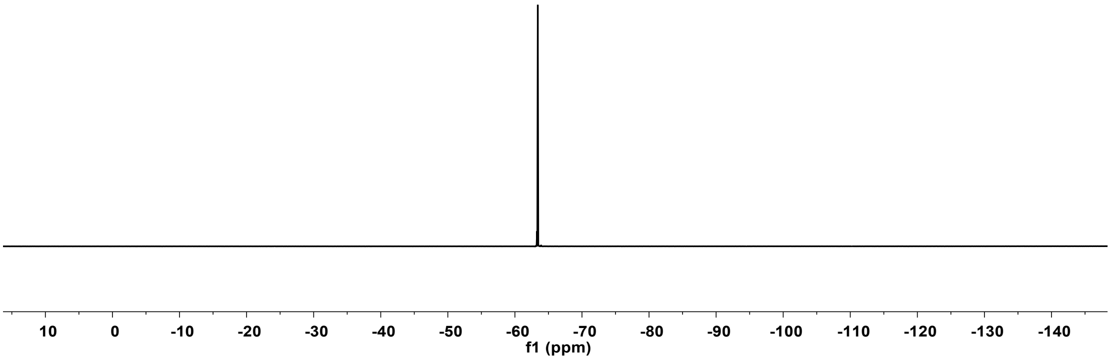
-63.411
10
0
-10
-20
-30
-40
-50
-60
-70
-80
-90
-100
-110
-120
-130
-140
f1 (ppm)

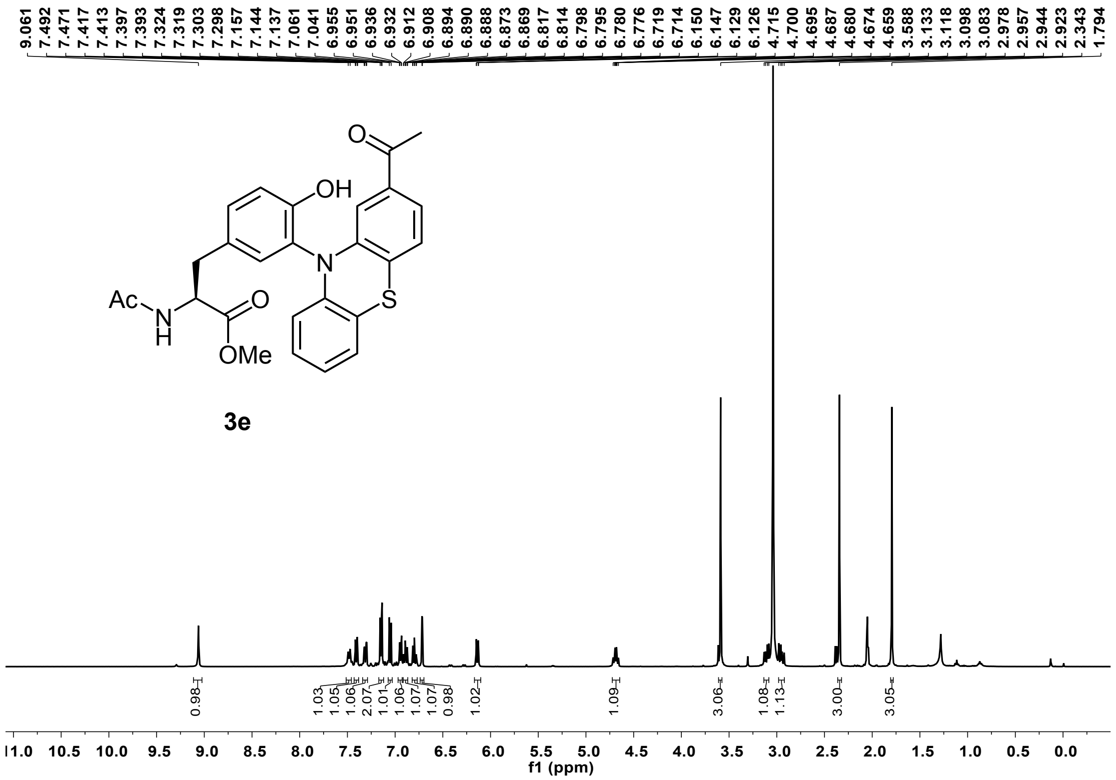
9.061 7.492 7.471 7.417 7.413 7.397 7.393 7.324 7.319 7.303 7.298 7.157 7.144 7.137 7.061 7.041 6.955 6.951 6.936 6.932 6.912 6.908 6.894 6.890 6.888 6.873 6.869 6.817 6.814 6.798 6.795 6.780 6.776 6.719 6.714 6.150 6.147 6.129 6.126 4.715 4.700 4.695 4.687 4.680 4.674 4.659 3.588 3.133 3.118 3.098 3.083 2.978 2.957 2.944 2.923 2.343 1.794
OH
N
S
O
Ac
N
H
O
OMe
3e
0.98 1.03 1.05 1.06 2.07 1.01 1.06 1.07 0.98 1.02 1.09 3.06 1.08 1.13 3.00 3.05
11.0 10.5 10.0 9.5 9.0 8.5 8.0 7.5 7.0 6.5 6.0 5.5 5.0 4.5 4.0 3.5 3.0 2.5 2.0 1.5 1.0 0.5 0.0
f1 (ppm)

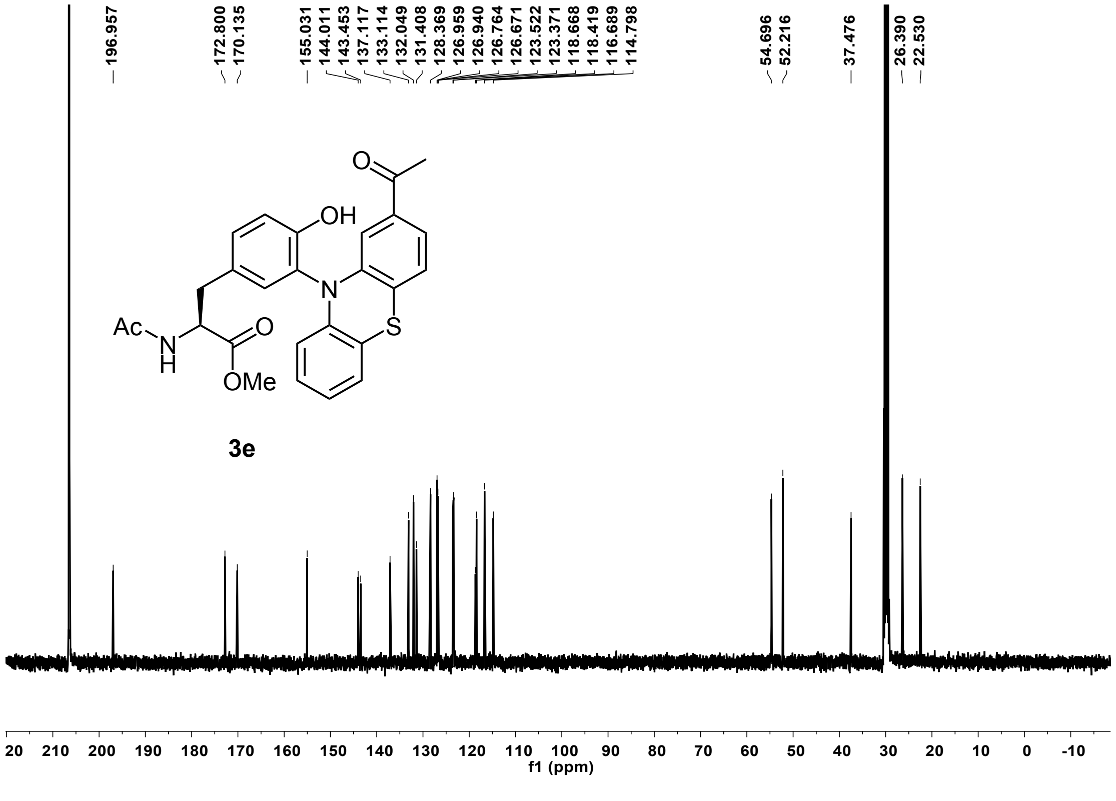
196.957 172.800 170.135 155.031 144.011 143.453 137.117 133.114 132.049 131.408 128.369 126.959 126.940 126.764 126.671 123.522 123.371 118.668 118.419 116.689 114.798 54.696 52.216 37.476 26.390 22.530
OH
N
S
O
Ac
N
H
O
OMe
3e
20 210 200 190 180 170 160 150 140 130 120 110 100 90 80 70 60 50 40 30 20 10 0 -10
f1 (ppm)

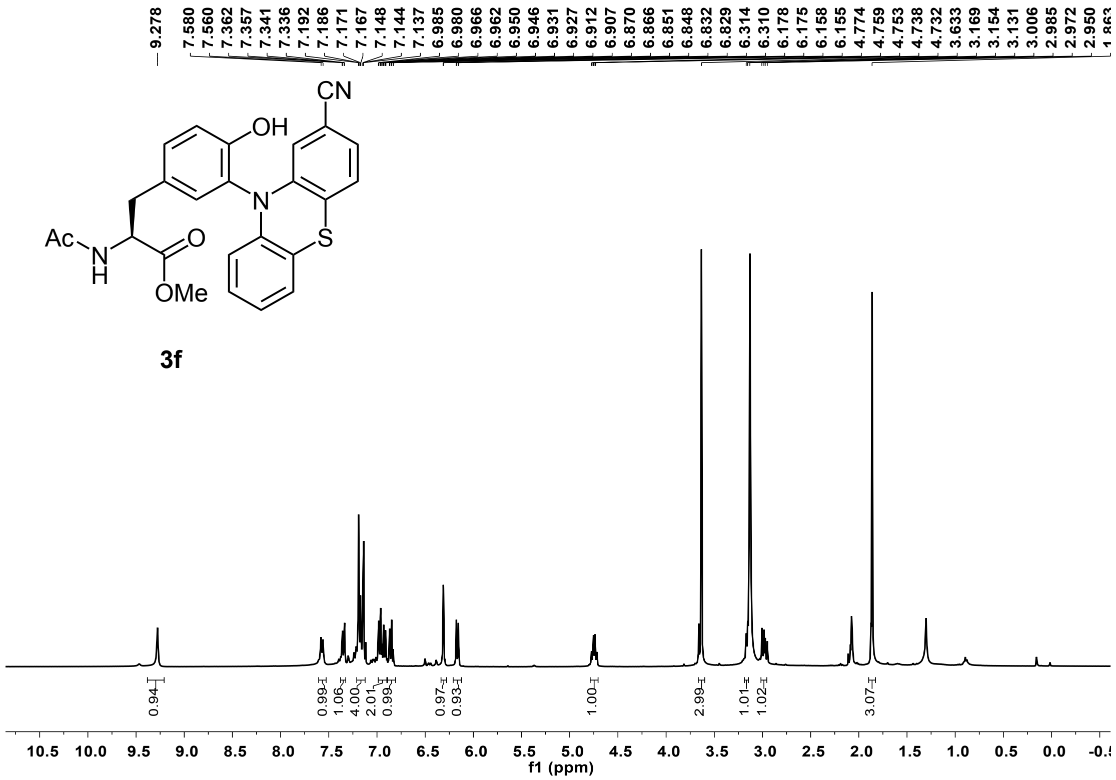
9.278
7.580 7.560 7.362 7.357 7.341 7.336 7.192 7.186 7.171 7.167 7.148 7.144 7.137 6.985 6.980 6.966 6.962 6.950 6.946 6.931 6.927 6.912 6.907 6.870 6.866 6.851 6.848 6.832 6.829 6.314 6.310 6.178 6.175 6.158 6.155 4.774 4.759 4.753 4.738 4.732 3.633 3.169 3.154 3.131 3.006 2.985 2.972 2.950
CN
OH
N
S
Ac
N
H
O
OMe
3f
0.94
0.99
1.06
4.00
2.01
0.99
0.97
0.93
1.00
2.99
1.01
1.02
3.07
10.5 10.0 9.5 9.0 8.5 8.0 7.5 7.0 6.5 6.0 5.5 5.0 4.5 4.0 3.5 3.0 2.5 2.0 1.5 1.0 0.5 0.0
f1 (ppm)

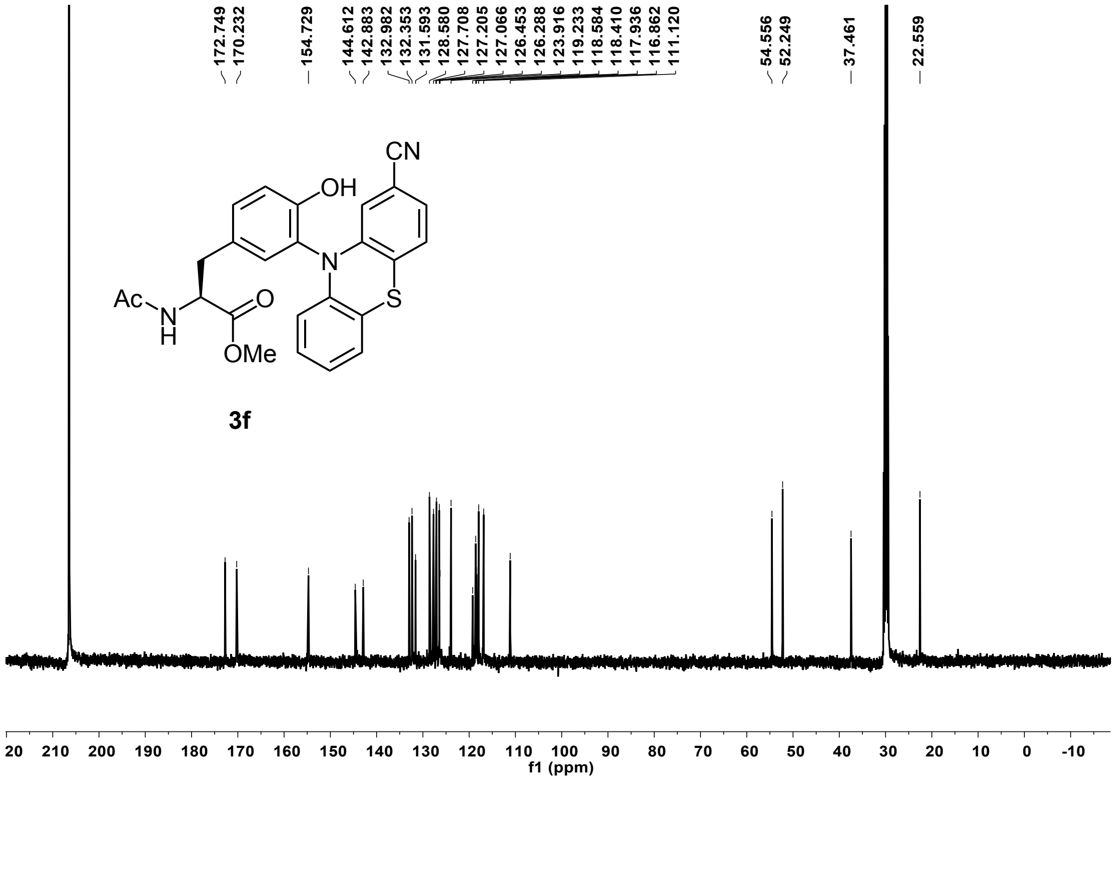
172.749
170.232
154.729
144.612
142.883
132.982
132.353
131.593
128.580
127.708
127.205
127.066
126.453
126.288
123.916
119.233
118.584
118.410
117.936
116.862
111.120
54.556
52.249
37.461
22.559
CN
OH
N
S
Ac
N
H
O
OMe
3f
20 210 200 190 180 170 160 150 140 130 120 110 100 90 80 70 60 50 40 30 20 10 0 -10
f1 (ppm)

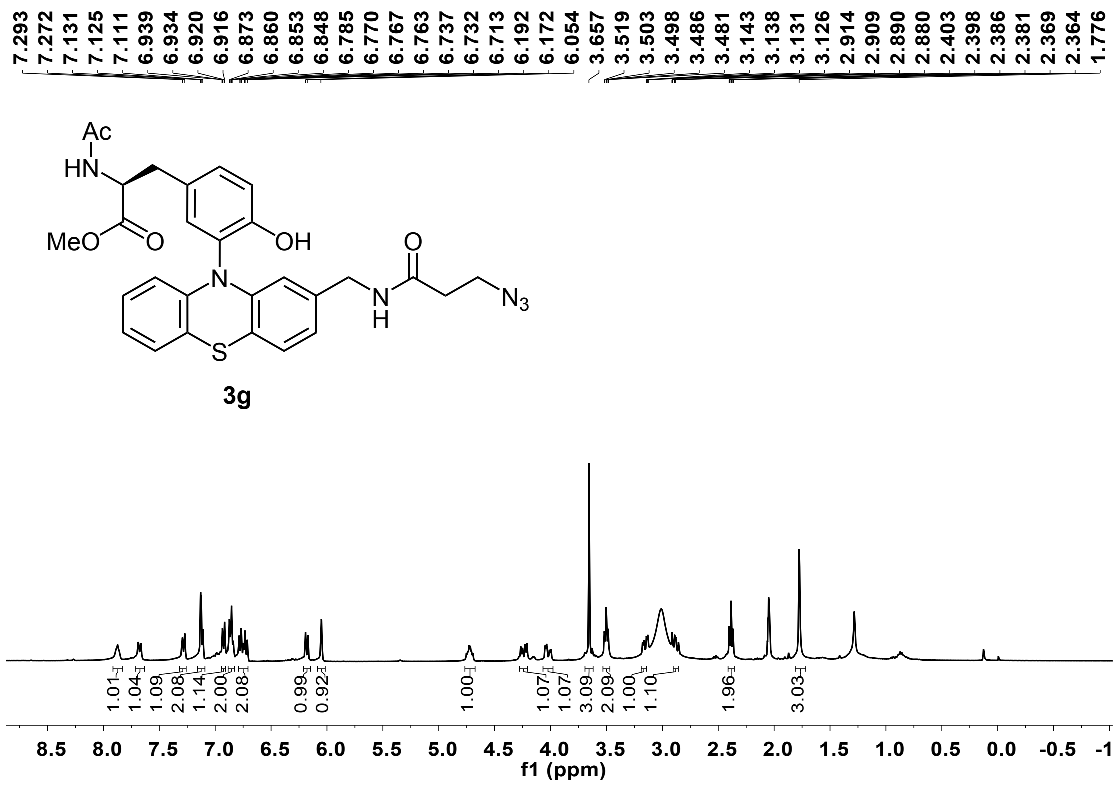
7.293
7.272
7.131
7.125
7.111
6.939
6.934
6.920
6.916
6.873
6.860
6.853
6.848
6.785
6.770
6.767
6.763
6.737
6.732
6.713
6.192
6.172
6.054
3.657
3.519
3.503
3.498
3.486
3.481
3.143
3.138
3.131
3.126
2.914
2.909
2.890
2.880
2.403
2.398
2.386
2.381
2.369
2.364
1.776
Ac
HN
MeO
O
OH
N
S
N
H
O
N3
3g
1.01
1.04
1.09
2.08
1.14
2.00
2.08
0.99
0.92
1.00
1.07
1.07
3.09
2.09
1.00
1.10
1.96
3.03
8.5 8.0 7.5 7.0 6.5 6.0 5.5 5.0 4.5 4.0 3.5 3.0 2.5 2.0 1.5 1.0 0.5 0.0 -0.5 -1
f1 (ppm)

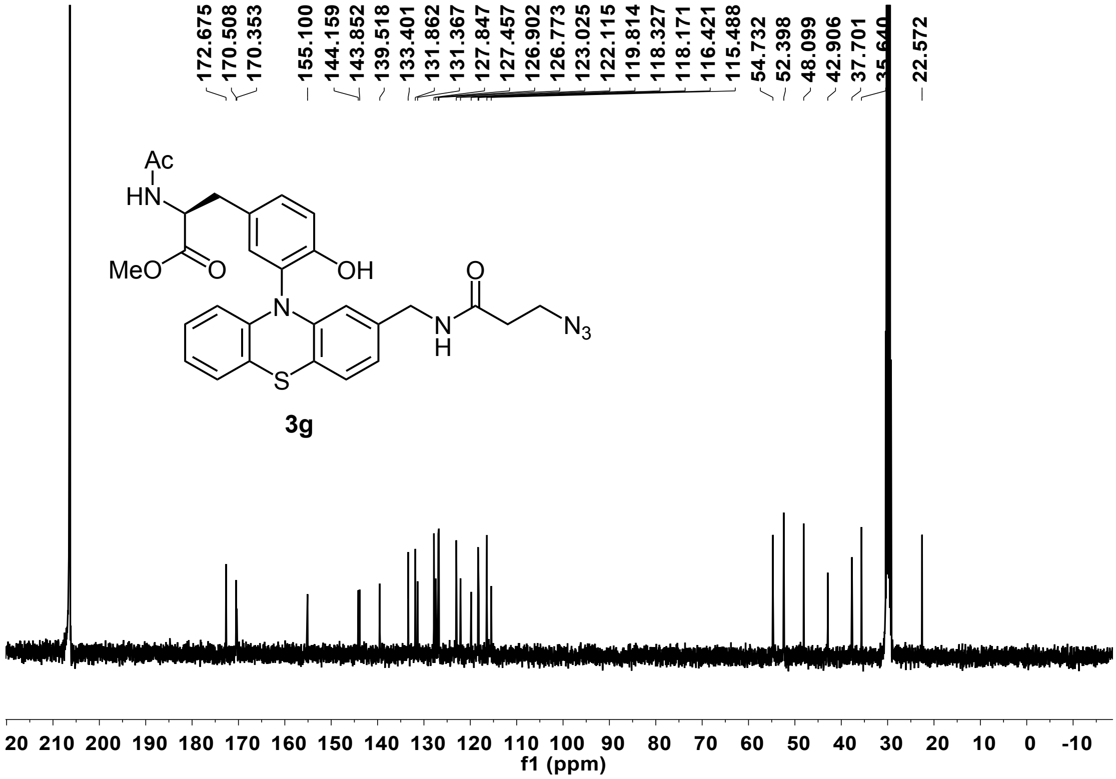
172.675
170.508
170.353
155.100
144.159
143.852
139.518
133.401
131.862
131.367
127.847
127.457
126.902
126.773
123.025
122.115
119.814
118.327
118.171
116.421
115.488
54.732
52.398
48.099
42.906
37.701
22.572
Ac
HN
MeO
O
OH
N
S
N
H
O
N3
3g
20 210 200 190 180 170 160 150 140 130 120 110 100 90 80 70 60 50 40 30 20 10 0 -10
f1 (ppm)

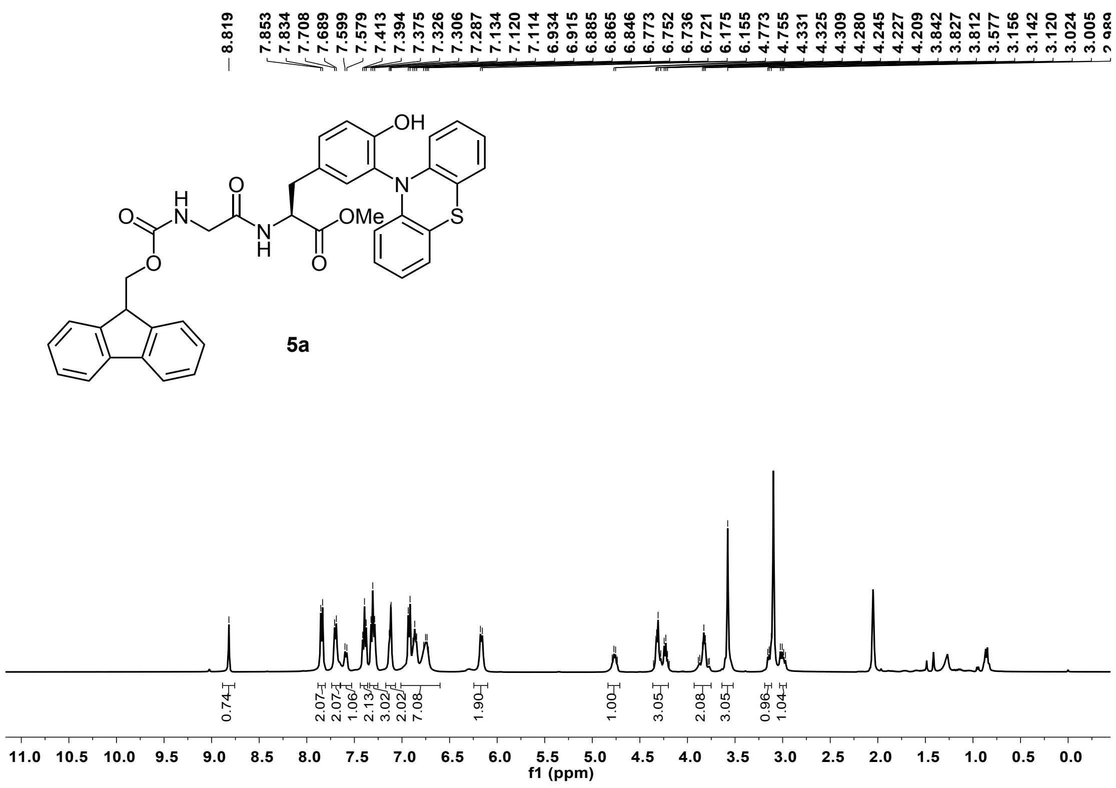

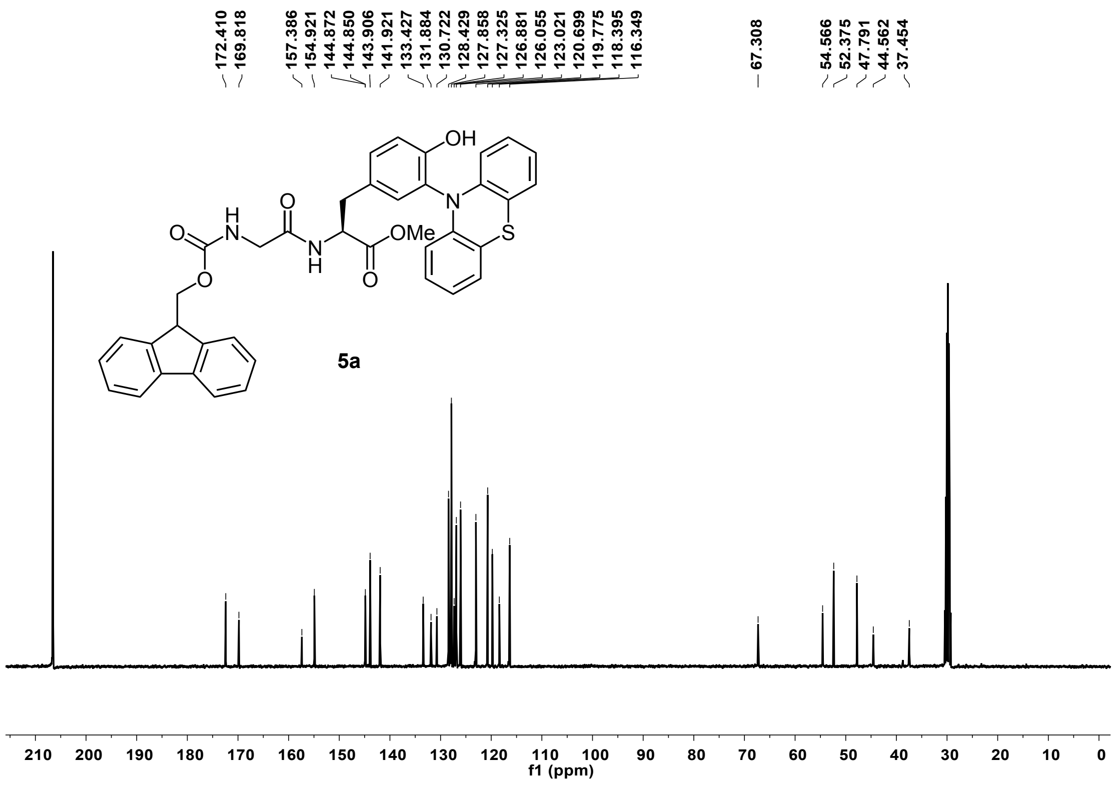

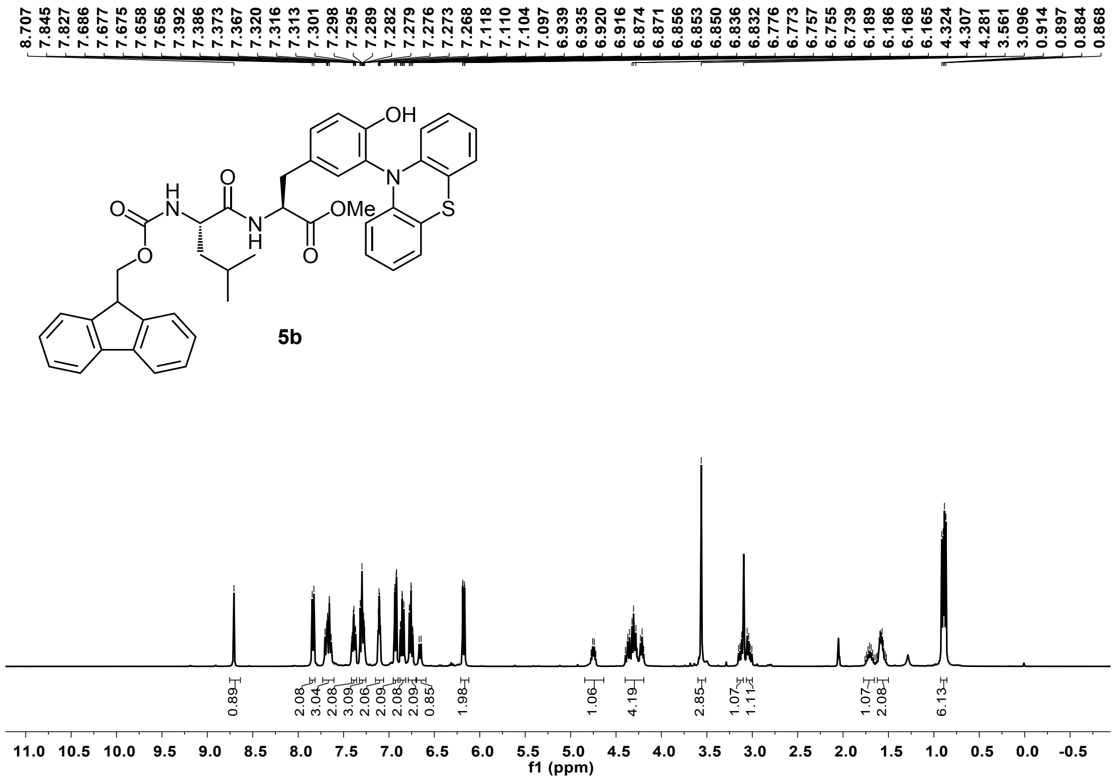
8.707
7.845
7.827
7.686
7.677
7.675
7.658
7.656
7.392
7.386
7.373
7.367
7.320
7.316
7.313
7.301
7.298
7.295
7.289
7.282
7.279
7.276
7.273
7.268
7.118
7.110
7.104
7.097
6.939
6.935
6.920
6.916
6.874
6.871
6.856
6.853
6.850
6.836
6.832
6.776
6.773
6.757
6.755
6.739
6.189
6.186
6.168
6.165
4.324
4.307
4.281
3.561
3.096
0.914
0.897
0.884
0.868
OH
N
S
O
H
N
OMe
5b
0.89
2.08
3.04
2.08
3.09
2.06
2.09
2.08
2.09
0.85
1.98
1.06
4.19
2.85
1.07
1.11
1.07
2.08
6.13
11.0 10.5 10.0 9.5 9.0 8.5 8.0 7.5 7.0 6.5 6.0 5.5 5.0 4.5 4.0 3.5 3.0 2.5 2.0 1.5 1.0 0.5 0.0 -0.5
f1 (ppm)

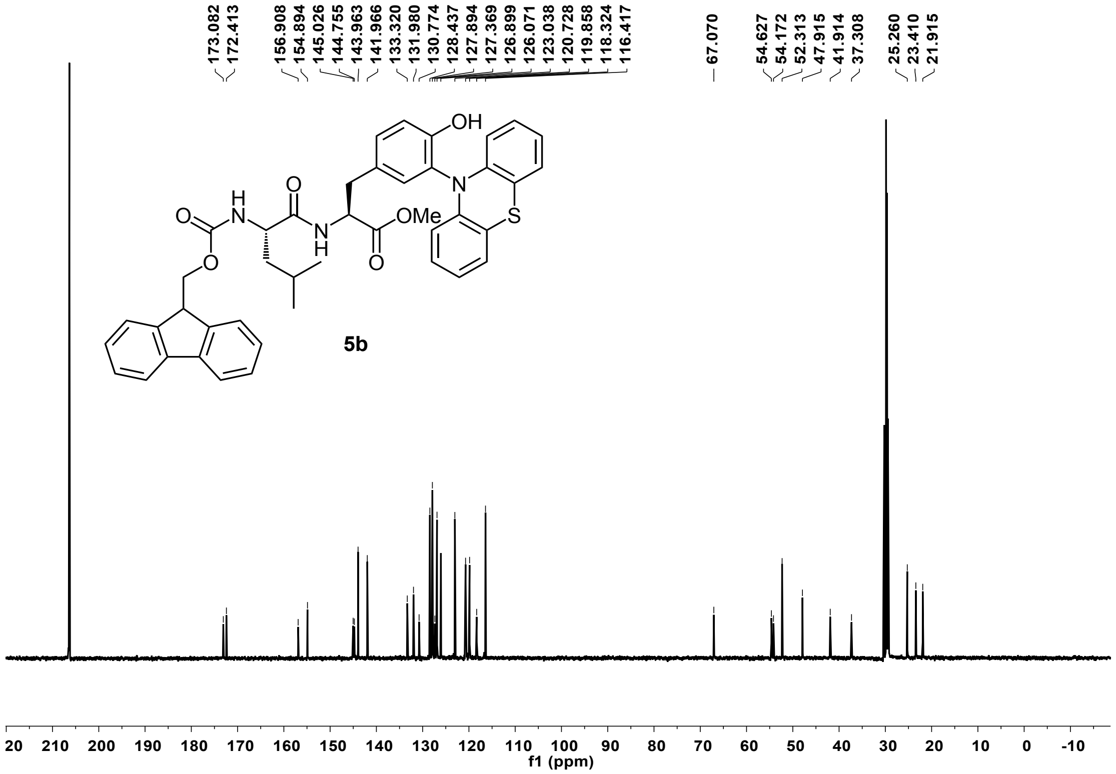
173.082
172.413
156.908
154.894
145.026
144.755
143.963
141.966
133.320
131.980
130.774
128.437
127.894
127.369
126.899
126.071
123.038
120.728
119.858
118.324
116.417
67.070
54.627
54.172
52.313
47.915
41.914
37.308
25.260
23.410
21.915
OH
N
S
O
H
N
OMe
5b
20 210 200 190 180 170 160 150 140 130 120 110 100 90 80 70 60 50 40 30 20 10 0 -10
f1 (ppm)

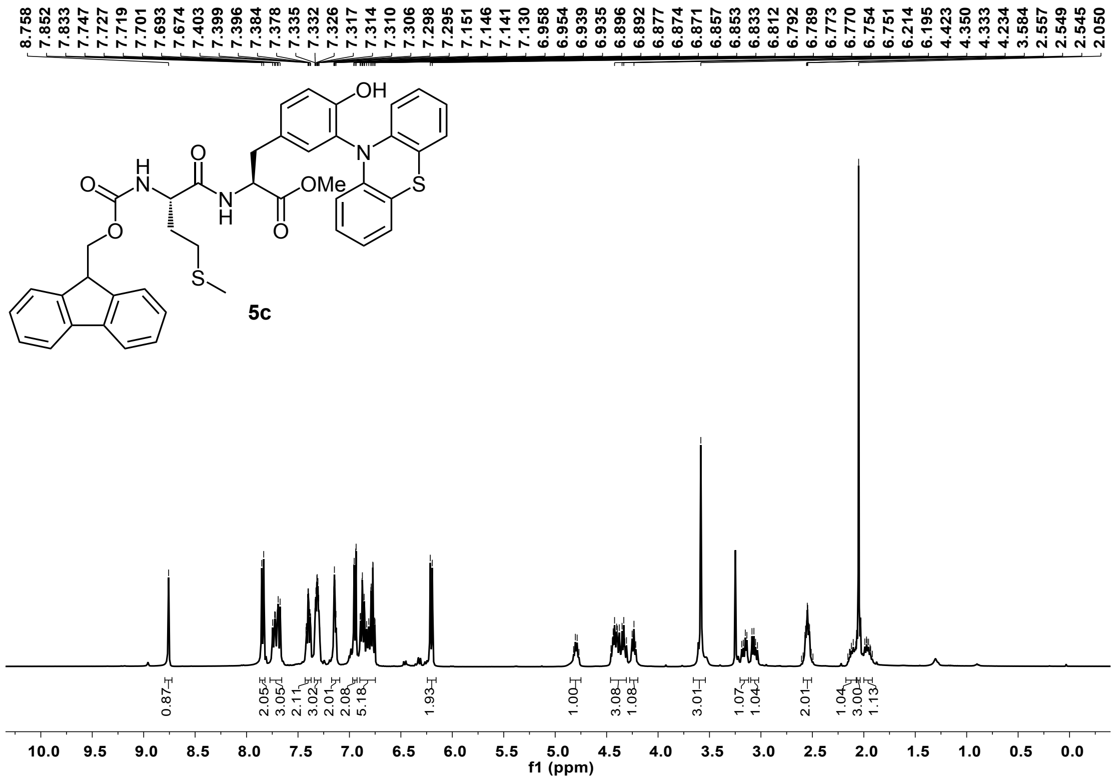
8.758 7.852 7.833 7.747 7.727 7.719 7.701 7.693 7.674 7.403 7.399 7.396 7.384 7.378 7.335 7.332 7.326 7.317 7.314 7.310 7.306 7.298 7.295 7.151 7.146 7.141 7.130 6.958 6.954 6.939 6.935 6.896 6.892 6.877 6.874 6.871 6.857 6.853 6.833 6.812 6.792 6.789 6.773 6.770 6.754 6.751 6.214 6.195 4.423 4.350 4.333 4.234 3.584 2.557 2.549 2.545 2.050
OH
N
S
O
H
N
OMe
O
H
S
5c
0.87
2.05
3.05
2.11
3.02
2.01
2.08
5.18
1.93
1.00
3.08
1.08
3.01
1.07
1.04
2.01
1.04
3.00
1.13
10.0 9.5 9.0 8.5 8.0 7.5 7.0 6.5 6.0 5.5 5.0 4.5 4.0 3.5 3.0 2.5 2.0 1.5 1.0 0.5 0.0
f1 (ppm)

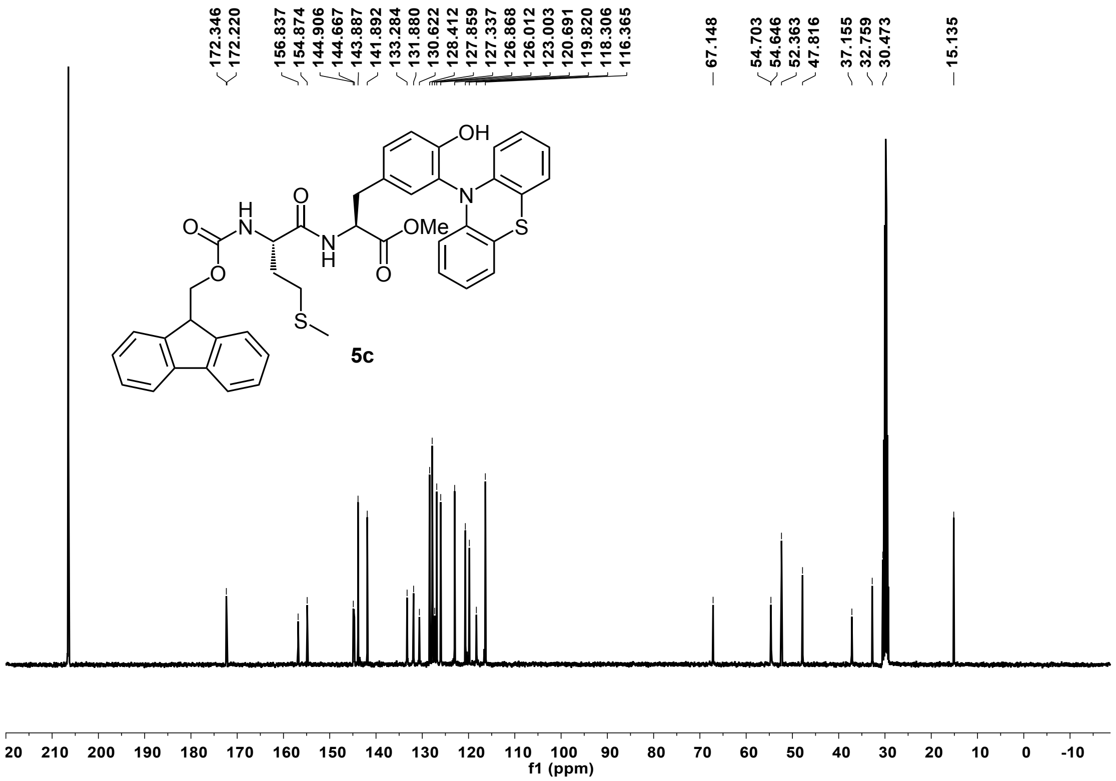
172.346 172.220
156.837 154.874 144.906 144.667 143.887 141.892 133.284 131.880 130.622 128.412 127.859 127.337 126.868 126.012 123.003 120.691 119.820 118.306 116.365
67.148
54.703 54.646 52.363 47.816
37.155 32.759 30.473
15.135
OH
N
S
O
H
N
OMe
O
H
S
5c
20 210 200 190 180 170 160 150 140 130 120 110 100 90 80 70 60 50 40 30 20 10 0 -10
f1 (ppm)

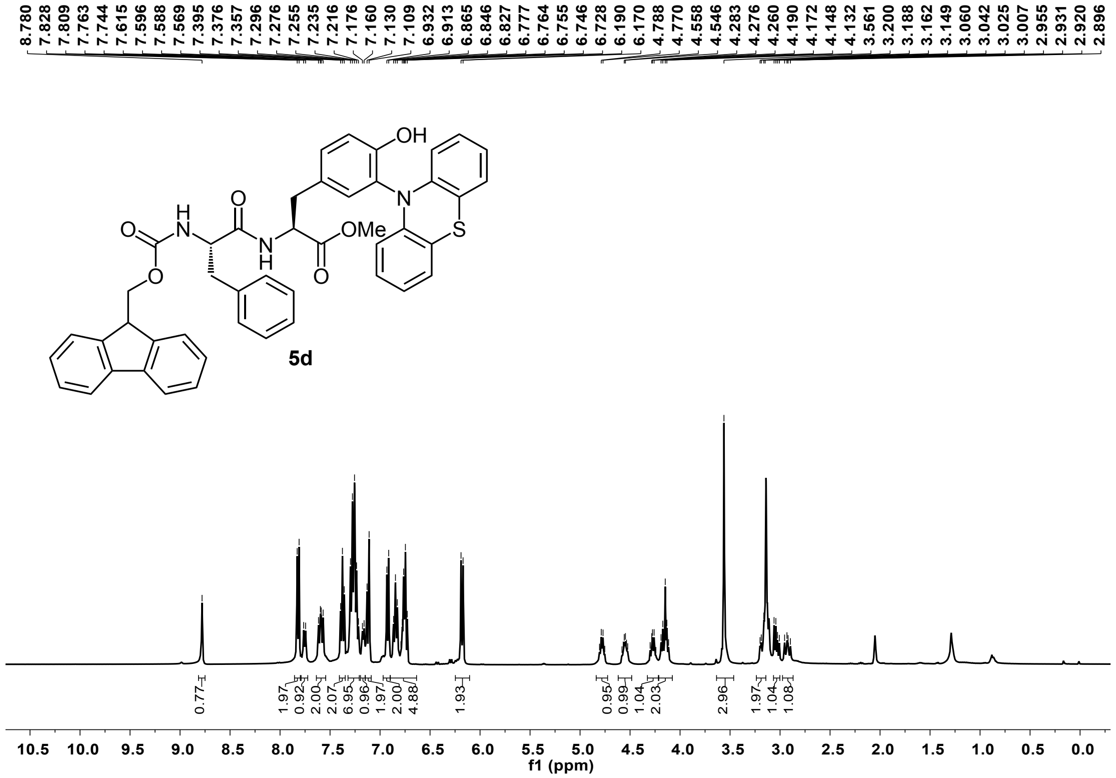
8.780 7.828 7.809 7.763 7.744 7.615 7.596 7.588 7.569 7.395 7.376 7.357 7.296 7.276 7.276 7.255 7.235 7.216 7.176 7.160 7.130 7.109 6.932 6.913 6.865 6.846 6.827 6.777 6.764 6.755 6.746 6.728 6.190 6.170 4.788 4.770 4.558 4.546 4.283 4.276 4.260 4.190 4.172 4.148 4.132 3.561 3.200 3.188 3.162 3.149 3.060 3.042 3.025 3.007 2.955 2.931 2.920 2.896
OH
N
S
H
N
O
O
O
N
H
OMe
O
5d
0.77 1.97 0.92 2.00 2.07 6.95 0.96 1.97 2.00 4.88 1.93 0.95 0.99 1.04 2.03 2.96 1.97 1.04 1.08
10.5 10.0 9.5 9.0 8.5 8.0 7.5 7.0 6.5 6.0 5.5 5.0 4.5 4.0 3.5 3.0 2.5 2.0 1.5 1.0 0.5 0.0
f1 (ppm)

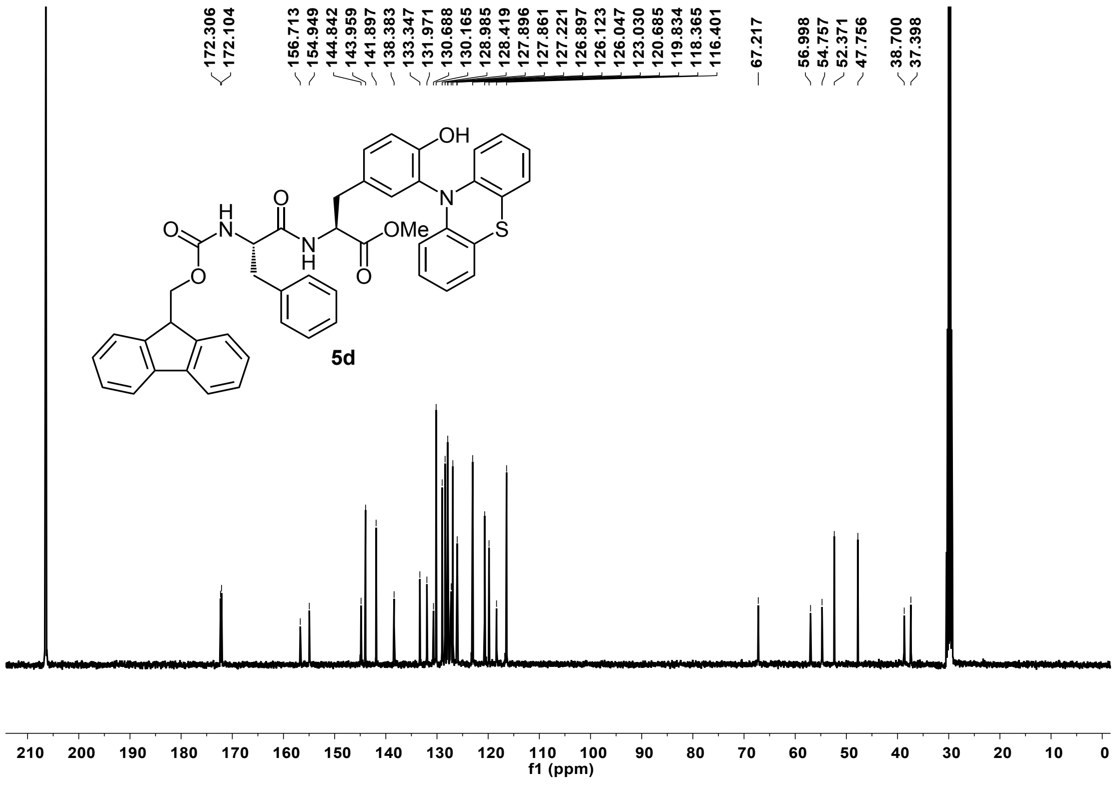
172.306 172.104 156.713 154.949 144.842 143.959 141.897 138.383 133.347 131.971 130.688 130.165 128.985 128.419 127.896 127.861 127.221 126.897 126.123 126.047 123.030 120.685 119.834 118.365 116.401 67.217 56.998 54.757 52.371 47.756 38.700 37.398
OH
N
S
H
N
O
O
O
N
H
OMe
O
5d
210 200 190 180 170 160 150 140 130 120 110 100 90 80 70 60 50 40 30 20 10 0
f1 (ppm)

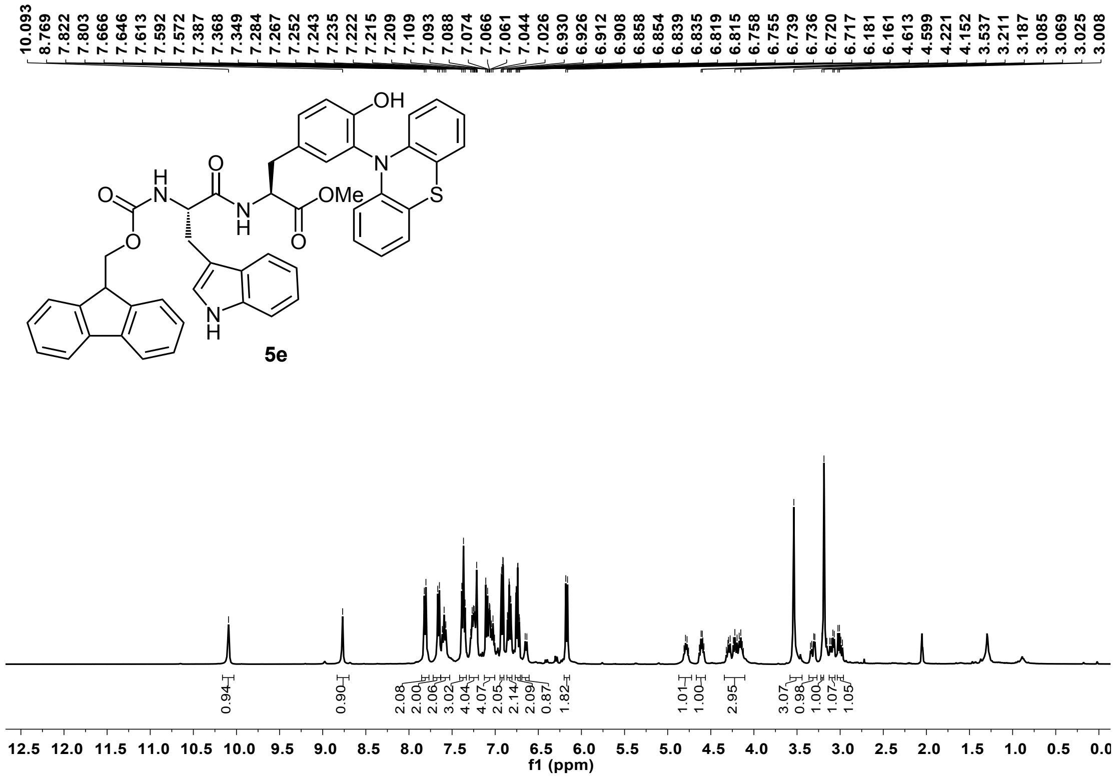
10.093
8.769
7.822
7.803
7.666
7.646
7.613
7.592
7.572
7.387
7.368
7.349
7.284
7.267
7.252
7.243
7.235
7.222
7.215
7.209
7.109
7.093
7.088
7.074
7.066
7.061
7.044
7.026
6.930
6.926
6.912
6.908
6.858
6.854
6.839
6.835
6.819
6.815
6.758
6.755
6.739
6.736
6.720
6.717
6.181
6.161
4.613
4.599
4.221
4.152
3.537
3.211
3.187
3.085
3.069
3.025
3.008
OH
N
S
H
N
O
O
N
H
OMe
O
O
NH
5e
0.94
0.90
2.08
2.00
2.06
3.02
4.04
4.07
2.05
2.14
2.09
0.87
1.82
1.01
1.00
2.95
3.07
0.98
1.00
1.07
1.05
12.5 12.0 11.5 11.0 10.5 10.0 9.5 9.0 8.5 8.0 7.5 7.0 6.5 6.0 5.5 5.0 4.5 4.0 3.5 3.0 2.5 2.0 1.5 1.0 0.5 0.0
f1 (ppm)

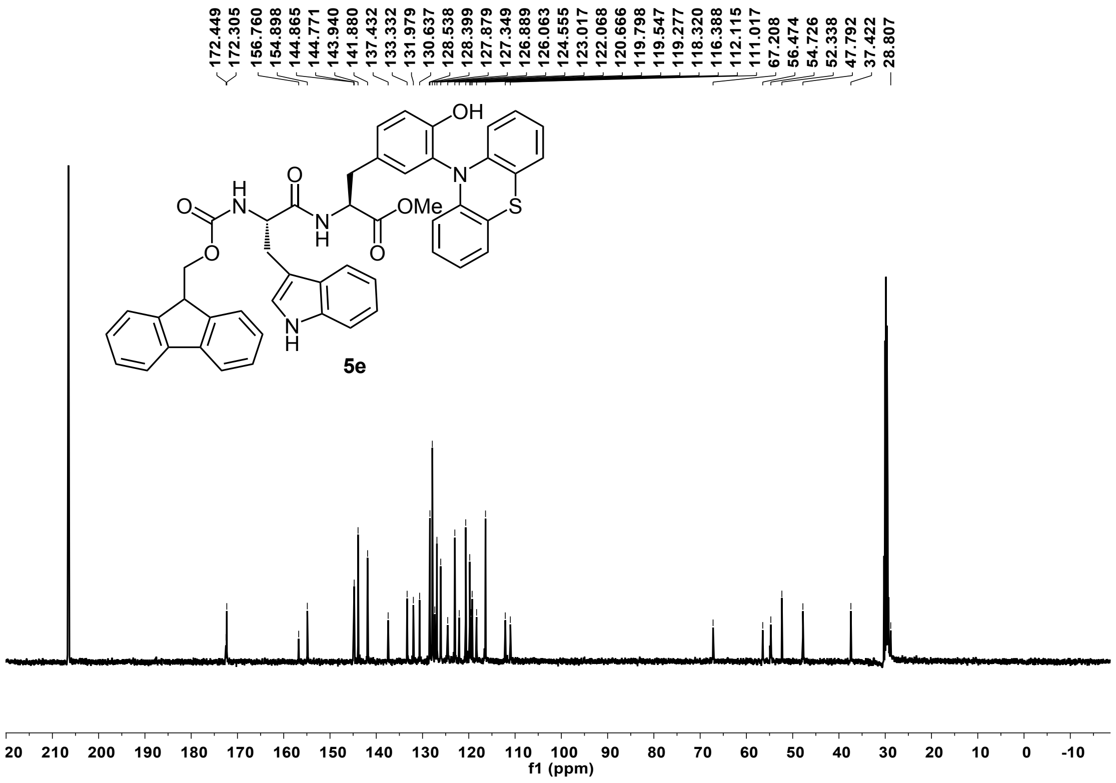
172.449
172.305
156.760
154.898
144.865
144.771
143.940
141.880
137.432
133.332
131.979
130.637
128.538
128.399
127.879
127.349
126.889
126.063
124.555
123.017
122.068
120.666
119.798
119.547
119.277
118.320
116.388
112.115
111.017
67.208
56.474
54.726
52.338
47.792
37.422
28.807
OH
N
S
H
N
O
O
N
H
OMe
O
O
NH
5e
20 210 200 190 180 170 160 150 140 130 120 110 100 90 80 70 60 50 40 30 20 10 0 -10
f1 (ppm)

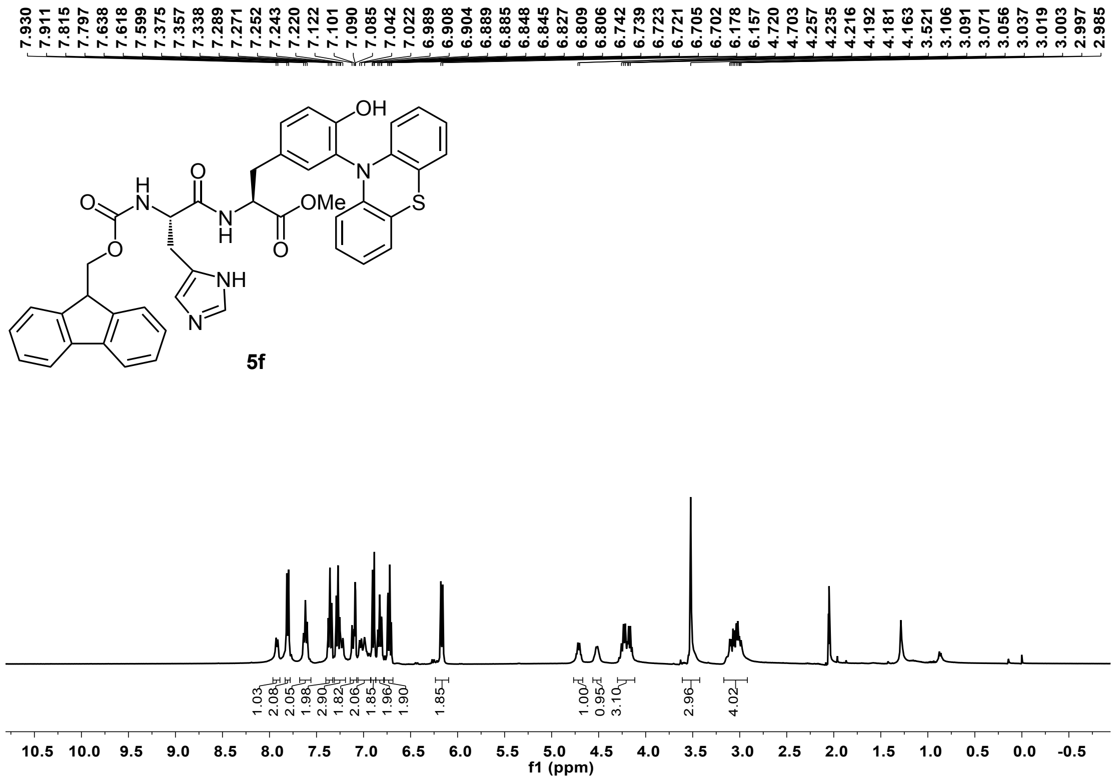
7.930
7.911
7.815
7.797
7.638
7.618
7.599
7.375
7.357
7.338
7.289
7.271
7.252
7.243
7.220
7.122
7.101
7.090
7.085
7.042
7.022
6.989
6.908
6.904
6.889
6.885
6.848
6.845
6.827
6.809
6.806
6.742
6.739
6.723
6.721
6.705
6.702
6.178
6.157
4.720
4.703
4.257
4.235
4.216
4.192
4.181
4.163
3.521
3.106
3.091
3.071
3.056
3.037
3.019
3.003
2.997
2.985
OH
N
S
H
N
O
OMe
H
O
NH
N
5f
1.03
2.08
2.05
1.98
2.90
1.82
2.06
1.85
1.96
1.90
1.85
1.00
0.95
3.10
2.96
4.02
f1 (ppm)

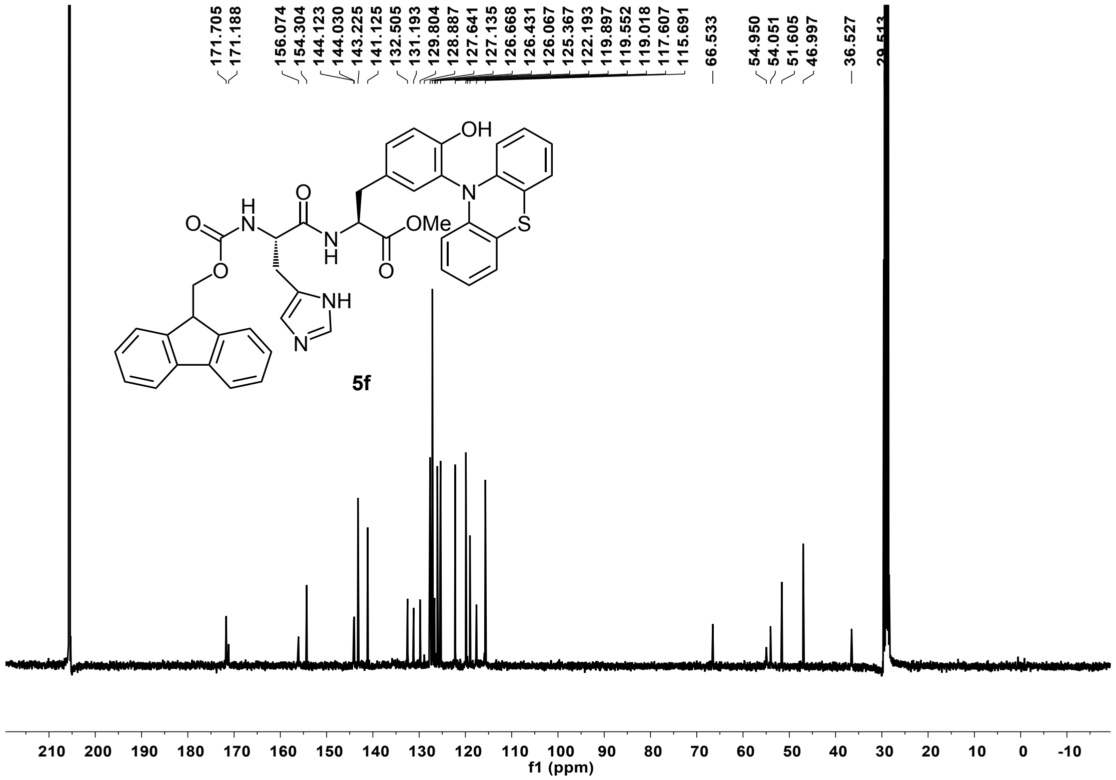
171.705
171.188
156.074
154.304
144.123
144.030
143.225
141.125
132.505
131.193
129.804
128.887
127.641
127.135
126.668
126.431
126.067
125.367
122.193
119.897
119.552
119.018
117.607
115.691
66.533
54.950
54.051
51.605
46.997
36.527
OH
N
S
H
N
O
OMe
H
O
NH
N
5f
f1 (ppm)

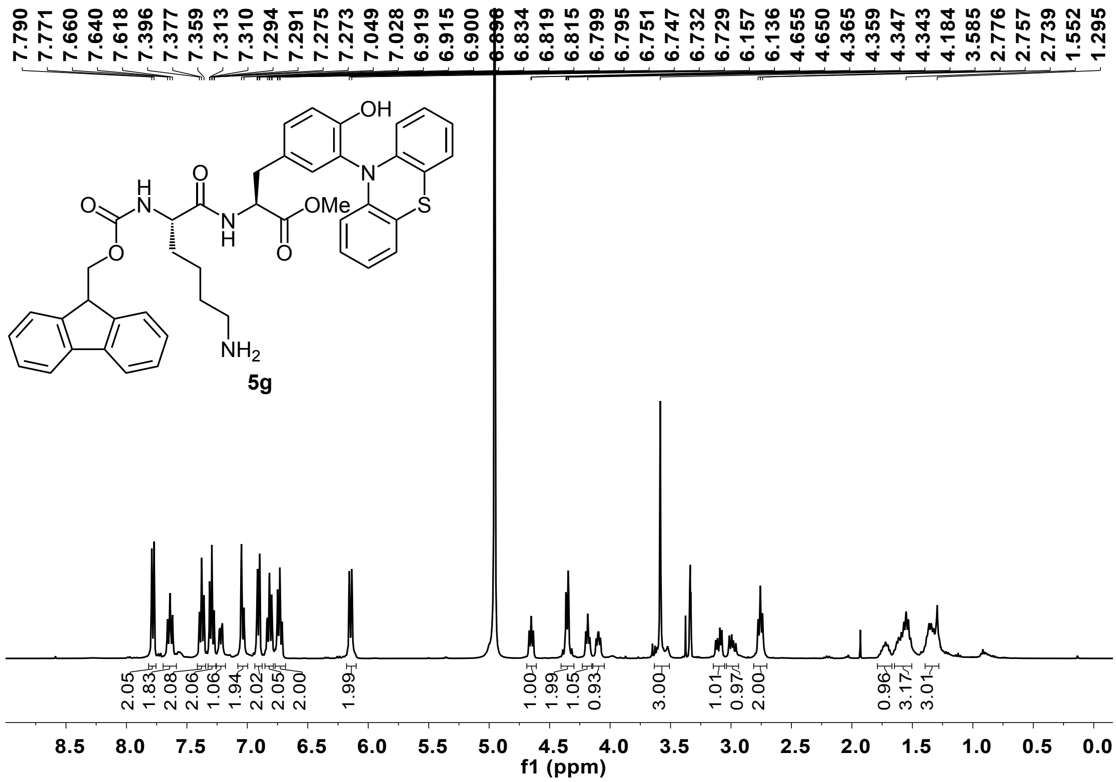
7.790
7.771
7.660
7.640
7.618
7.396
7.377
7.359
7.313
7.310
7.294
7.291
7.275
7.273
7.049
7.028
6.919
6.915
6.900
6.834
6.819
6.815
6.799
6.795
6.751
6.747
6.732
6.729
6.157
6.136
4.655
4.650
4.365
4.359
4.347
4.343
4.184
3.585
2.776
2.757
2.739
1.552
1.295
OH
N
S
O
H
N
OMe
O
NH2
5g
2.05
1.83
2.08
2.06
1.06
1.94
2.02
2.05
2.00
1.99
1.00
1.99
1.05
0.93
3.00
1.01
0.97
2.00
0.96
3.17
3.01
8.5 8.0 7.5 7.0 6.5 6.0 5.5 5.0 4.5 4.0 3.5 3.0 2.5 2.0 1.5 1.0 0.5 0.0
f1 (ppm)

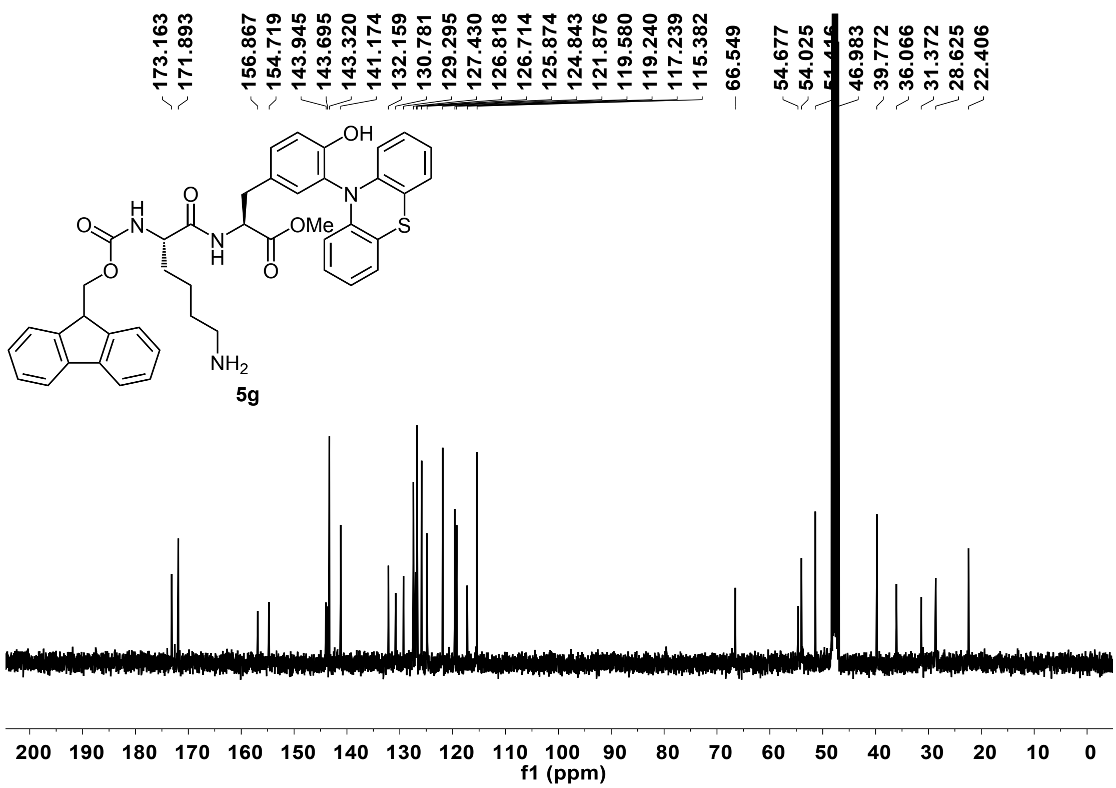
173.163
171.893
156.867
154.719
143.945
143.695
143.320
141.174
132.159
130.781
129.295
127.430
126.818
126.714
125.874
124.843
121.876
119.580
119.240
117.239
115.382
66.549
54.677
54.025
46.983
39.772
36.066
31.372
28.625
22.406
OH
N
S
O
H
N
OMe
O
NH2
5g
200 190 180 170 160 150 140 130 120 110 100 90 80 70 60 50 40 30 20 10 0
f1 (ppm)

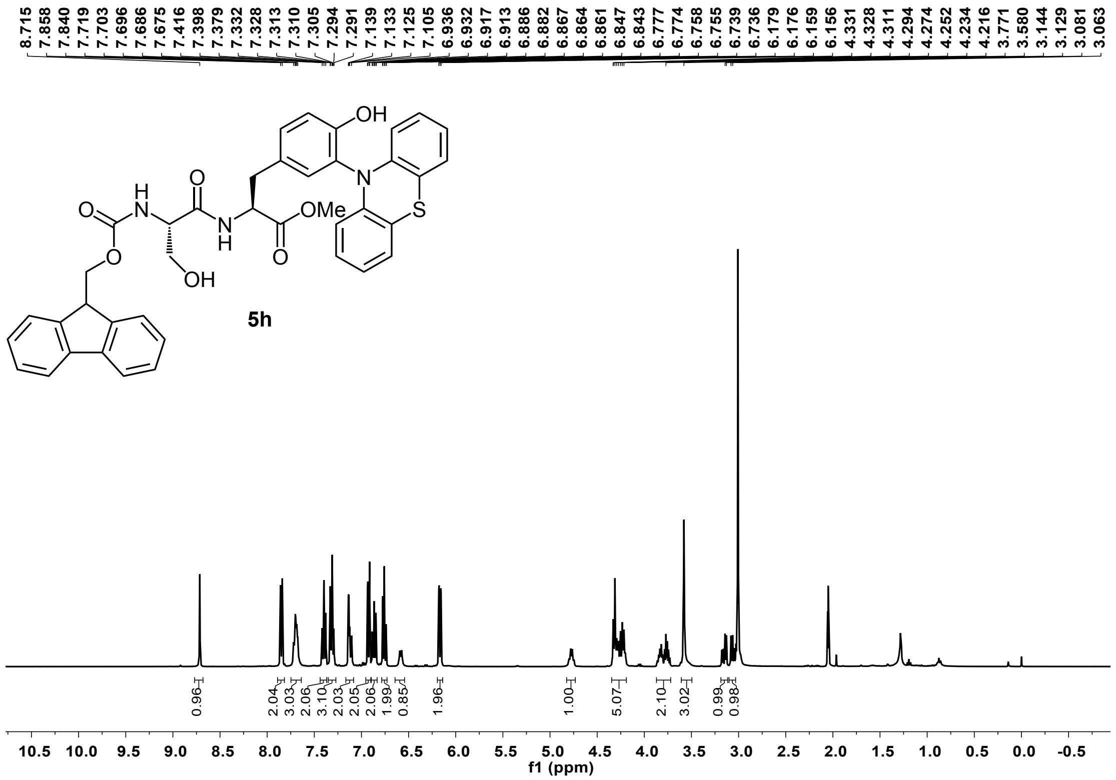
8.715
7.858
7.840
7.719
7.703
7.696
7.686
7.675
7.416
7.398
7.379
7.332
7.328
7.313
7.310
7.305
7.294
7.291
7.139
7.133
7.125
7.105
6.936
6.932
6.917
6.913
6.886
6.882
6.867
6.864
6.861
6.847
6.843
6.777
6.774
6.758
6.755
6.739
6.736
6.179
6.176
6.159
6.156
4.331
4.328
4.311
4.294
4.274
4.252
4.234
4.216
3.771
3.580
3.144
3.129
3.081
3.063
OH
N
S
H
N
O
O
O
N
H
OMe
O
O
OH
5h
0.96
2.04
3.03
2.06
3.10
2.03
2.05
2.06
1.99
0.85
1.96
1.00
5.07
2.10
3.02
0.99
0.98
10.5 10.0 9.5 9.0 8.5 8.0 7.5 7.0 6.5 6.0 5.5 5.0 4.5 4.0 3.5 3.0 2.5 2.0 1.5 1.0 0.5 0.0 -0.5
f1 (ppm)

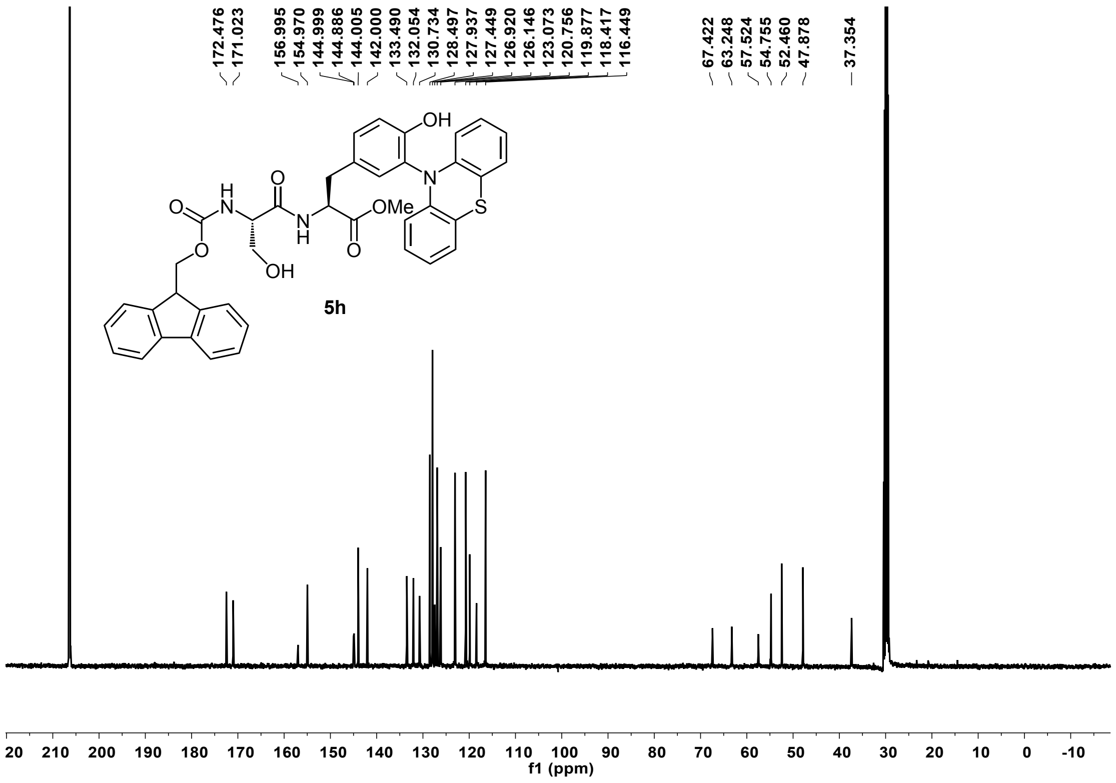
172.476
171.023
156.995
154.970
144.999
144.886
144.005
142.000
133.490
132.054
130.734
128.497
127.937
127.449
126.920
126.146
123.073
120.756
119.877
118.417
116.449
67.422
63.248
57.524
54.755
52.460
47.878
37.354
OH
N
S
H
N
O
O
O
N
H
OMe
O
O
OH
5h
20 210 200 190 180 170 160 150 140 130 120 110 100 90 80 70 60 50 40 30 20 10 0 -10
f1 (ppm)

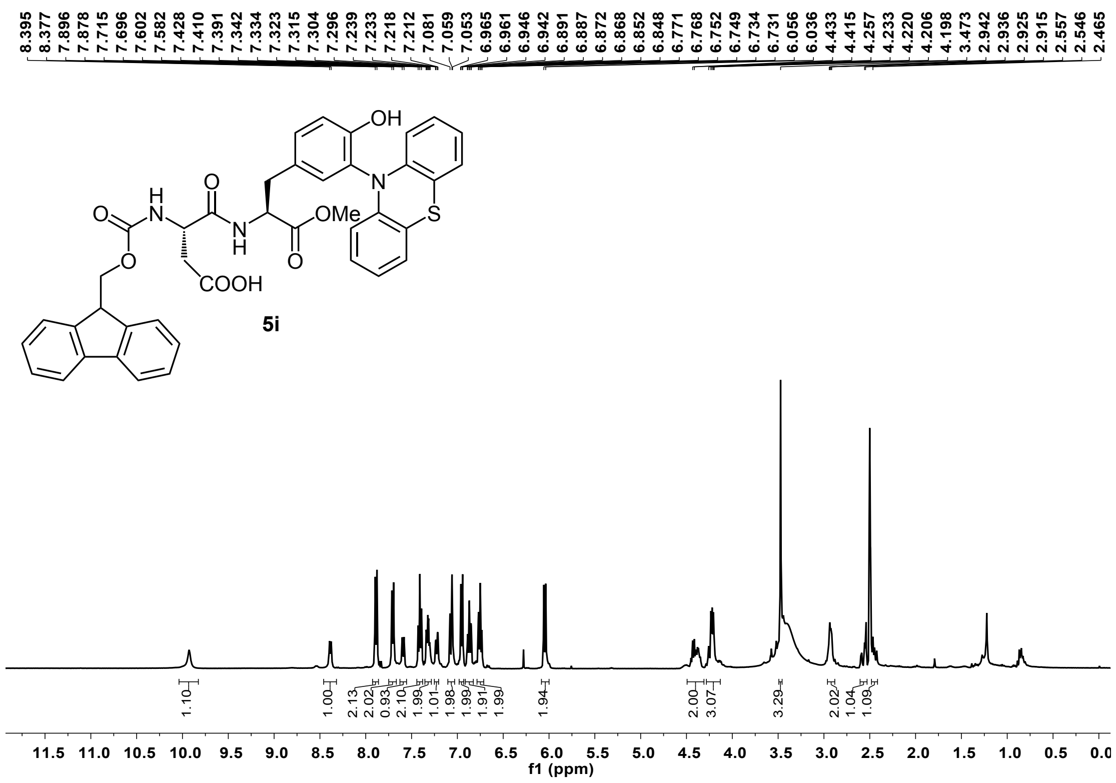
8.395
8.377
7.896
7.878
7.715
7.696
7.602
7.582
7.428
7.410
7.391
7.342
7.334
7.323
7.315
7.304
7.296
7.239
7.233
7.218
7.212
7.081
7.059
7.053
6.965
6.961
6.946
6.942
6.891
6.887
6.872
6.868
6.852
6.848
6.771
6.768
6.752
6.749
6.734
6.731
6.056
6.036
4.433
4.415
4.257
4.233
4.220
4.206
4.198
3.473
2.942
2.936
2.925
2.915
2.557
2.546
2.465
OH
N
S
O
H
N
O
N
H
OMe
O
O
COOH
5i
1.10
1.00
2.13
2.02
0.93
2.10
1.99
1.01
1.98
1.99
1.91
1.99
1.94
2.00
3.07
3.29
2.02
1.04
1.09
11.5 11.0 10.5 10.0 9.5 9.0 8.5 8.0 7.5 7.0 6.5 6.0 5.5 5.0 4.5 4.0 3.5 3.0 2.5 2.0 1.5 1.0 0.5 0.0
f1 (ppm)

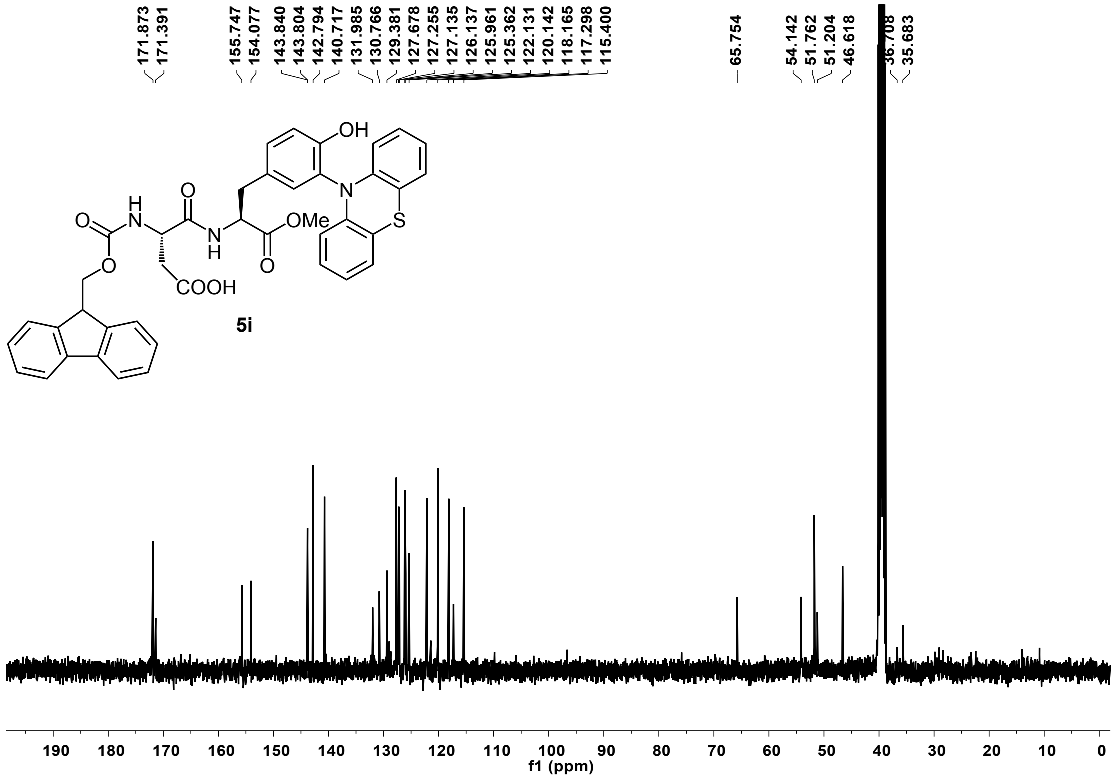
171.873
171.391
155.747
154.077
143.840
143.804
142.794
140.717
131.985
130.766
129.381
127.678
127.255
127.135
126.137
125.961
125.362
122.131
120.142
118.165
117.298
115.400
65.754
54.142
51.762
51.204
46.618
36.708
35.683
OH
N
S
O
H
N
O
N
H
OMe
O
O
COOH
5i
190 180 170 160 150 140 130 120 110 100 90 80 70 60 50 40 30 20 10 0
f1 (ppm)

5.2 UV-VIS Absorption Spectra of Reactants and Products
Normalized UV-VIS Spectra of Reactants and Products

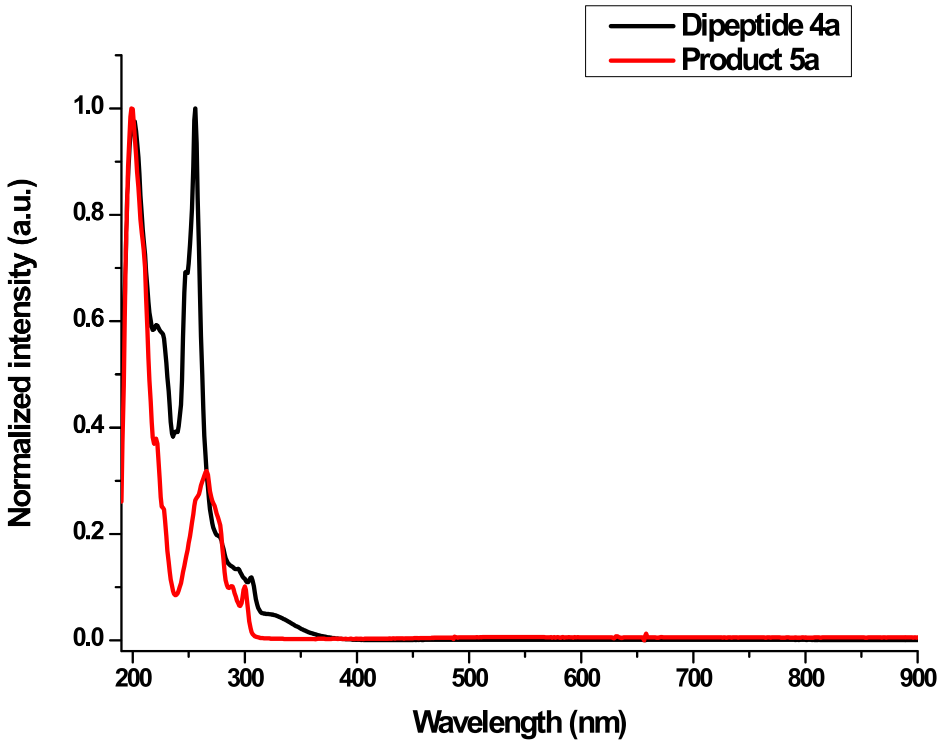

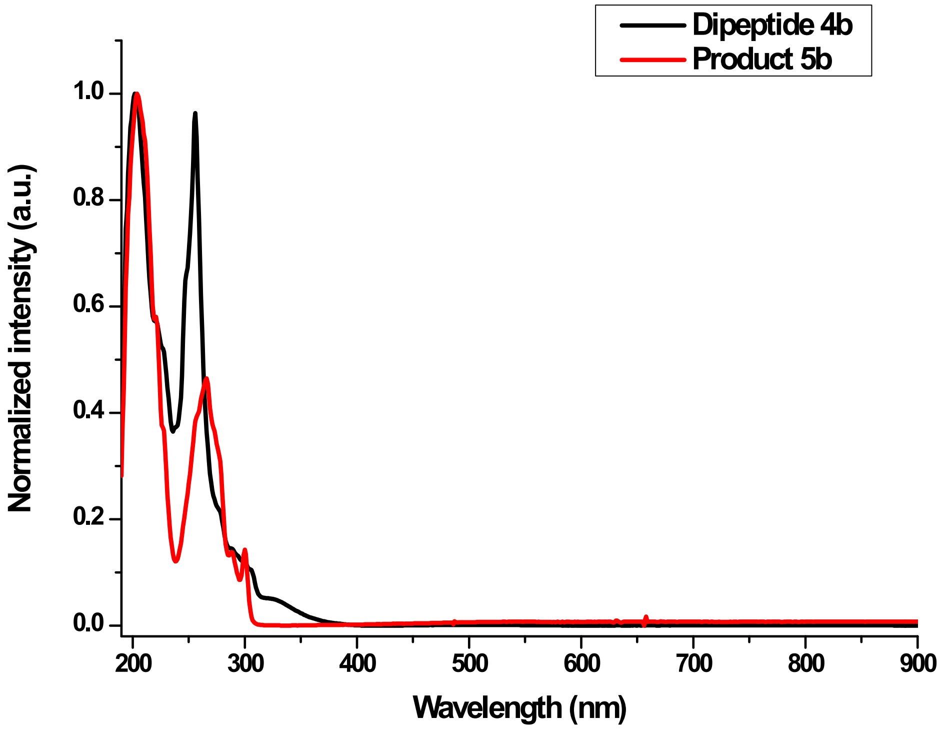

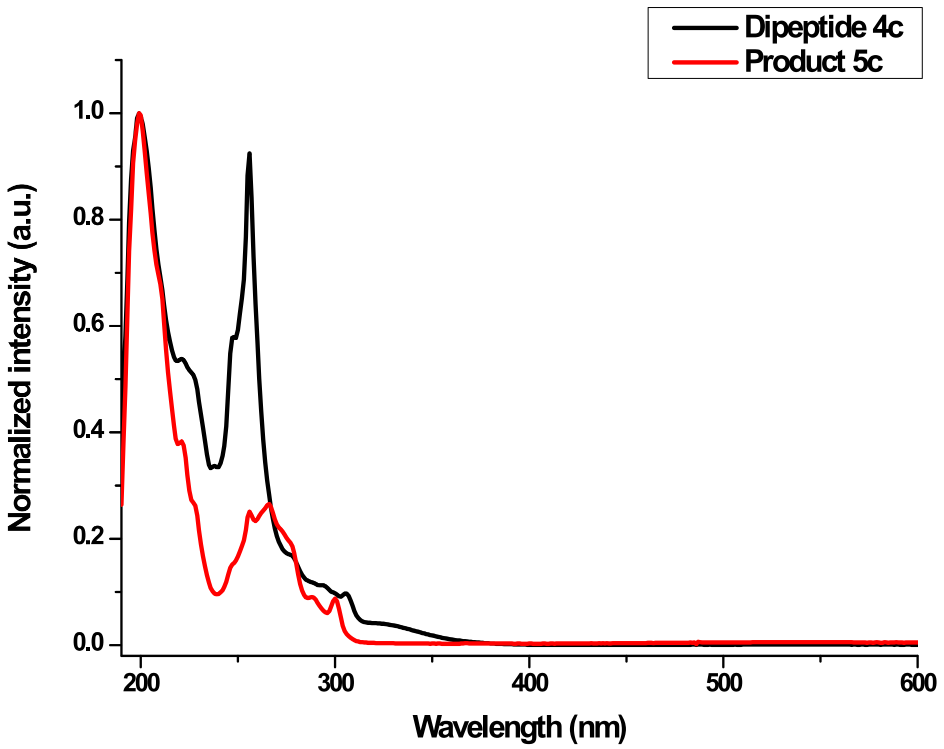
Dipeptide 4c
Product 5c
Normalized intensity (a.u.)
1.0
0.8
0.6
0.4
0.2
0.0
200
300
400
500
600
Wavelength (nm)

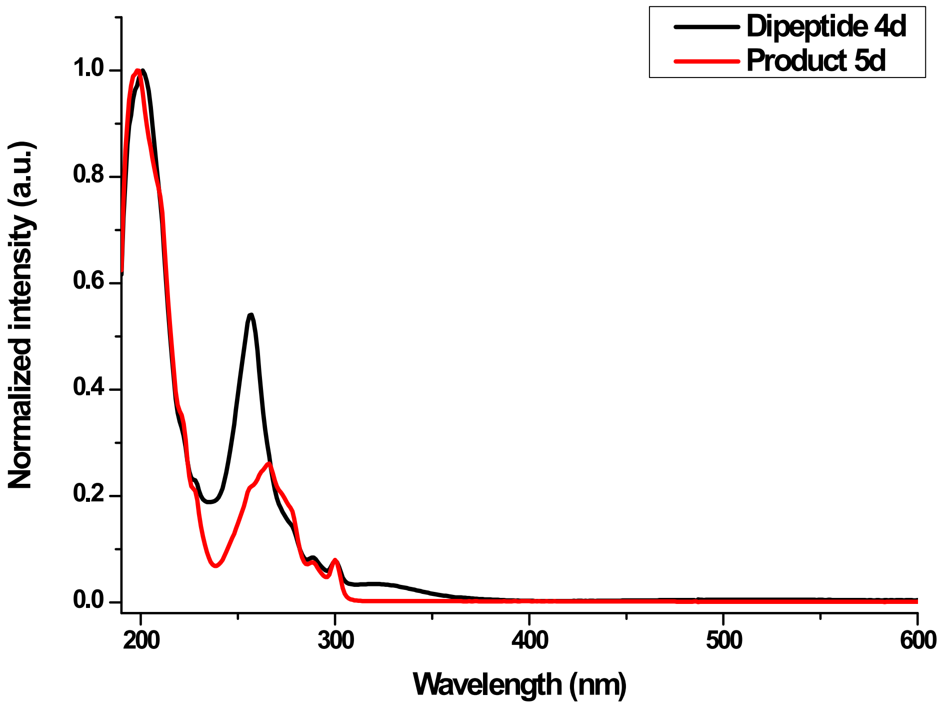
Dipeptide 4d
Product 5d
Normalized intensity (a.u.)
1.0
0.8
0.6
0.4
0.2
0.0
200
300
400
500
600
Wavelength (nm)

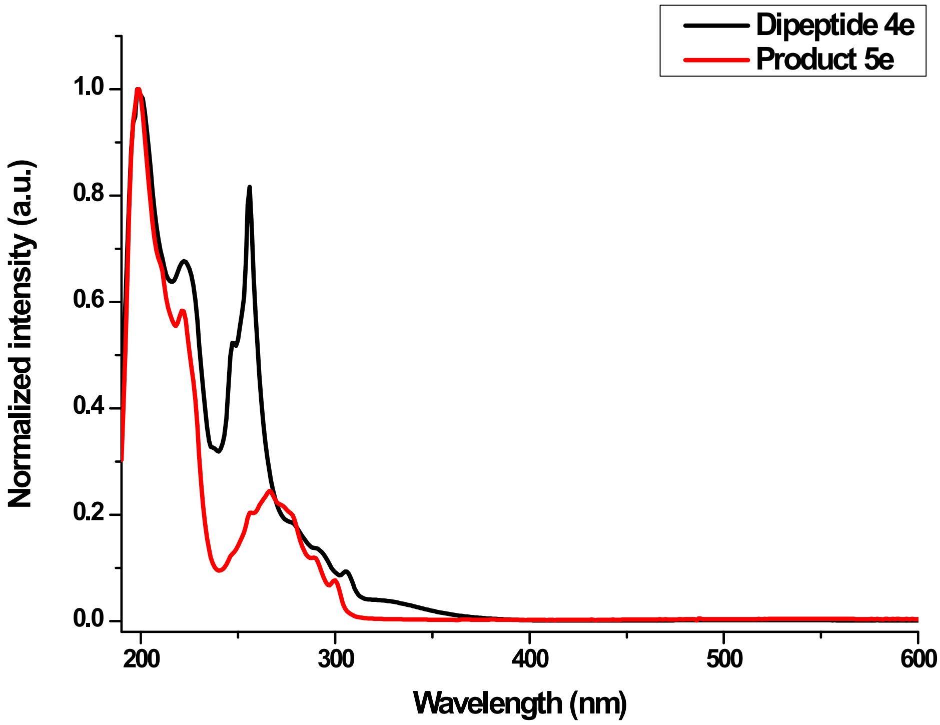
Dipeptide 4e
Product 5e
Normalized intensity (a.u.)
1.0
0.8
0.6
0.4
0.2
0.0
200
300
400
500
600
Wavelength (nm)

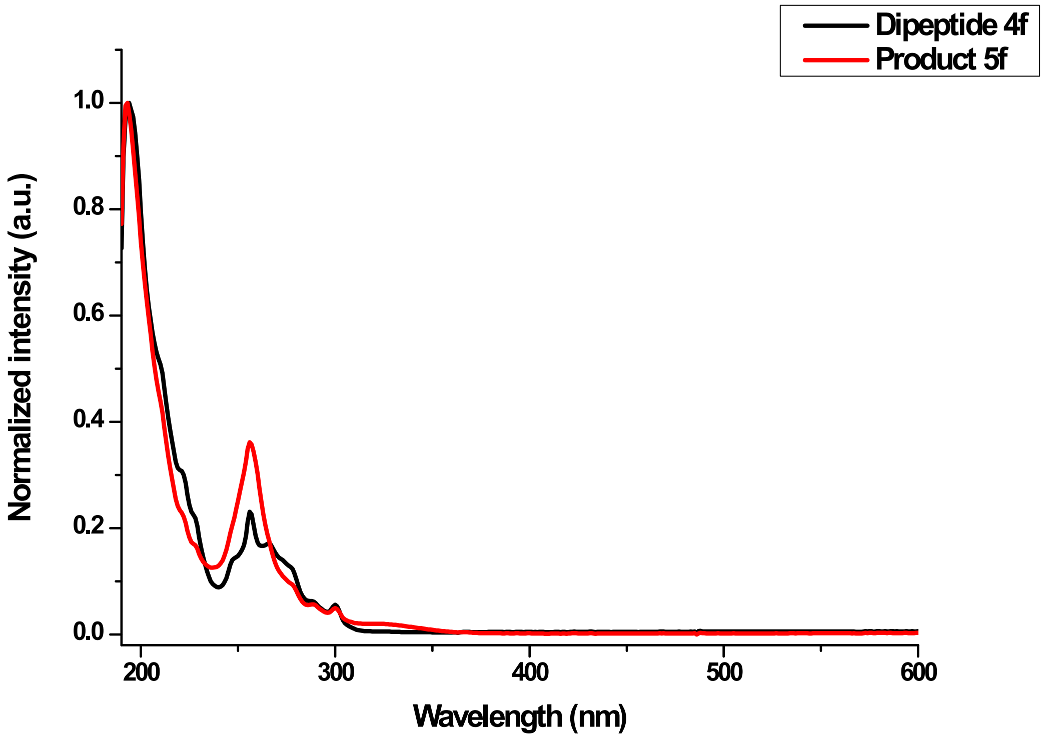
Dipeptide 4f
Product 5f
Normalized intensity (a.u.)
1.0
0.8
0.6
0.4
0.2
0.0
200
300
400
500
600
Wavelength (nm)

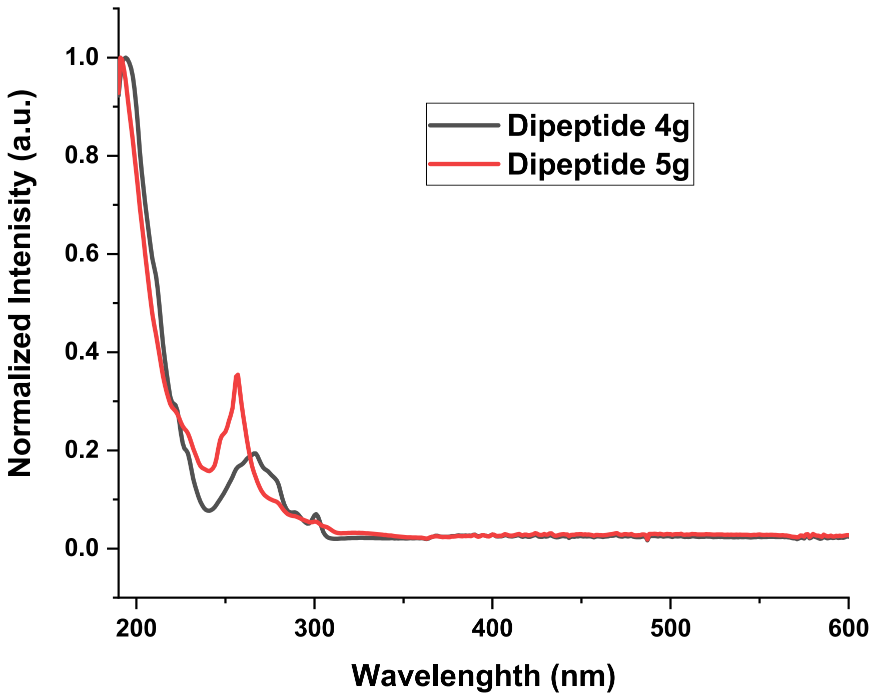

Dipeptide 4g
Dipeptide 5g
Normalized Intenisity (a.u.)
Wavelenghth (nm)
1.0
0.8
0.6
0.4
0.2
0.0
200
300
400
500
600

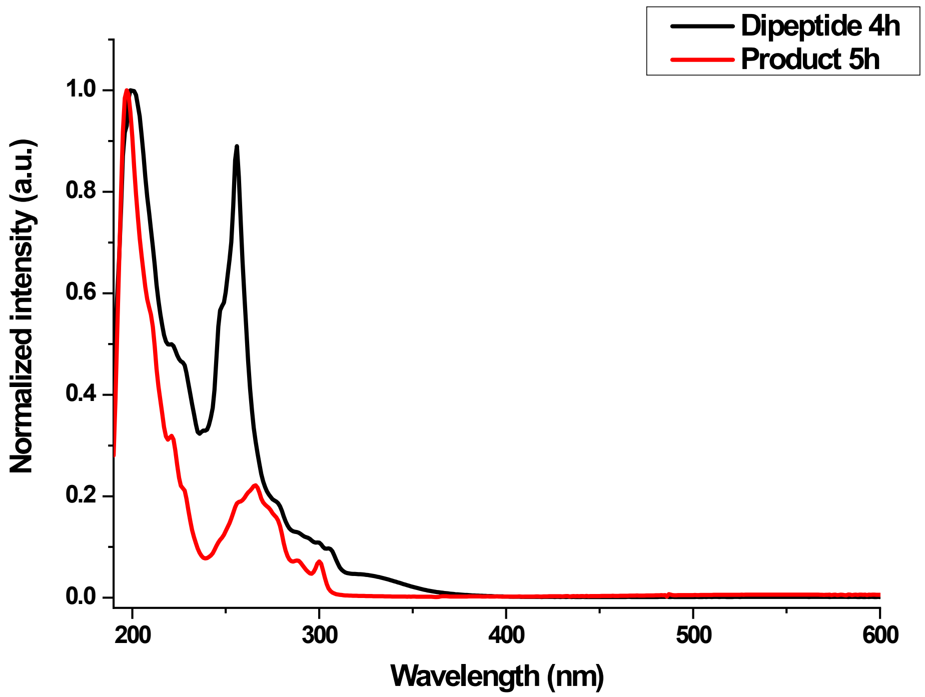

Dipeptide 4h
Product 5h
Normalized intensity (a.u.)
Wavelength (nm)
1.0
0.8
0.6
0.4
0.2
0.0
200
300
400
500
600

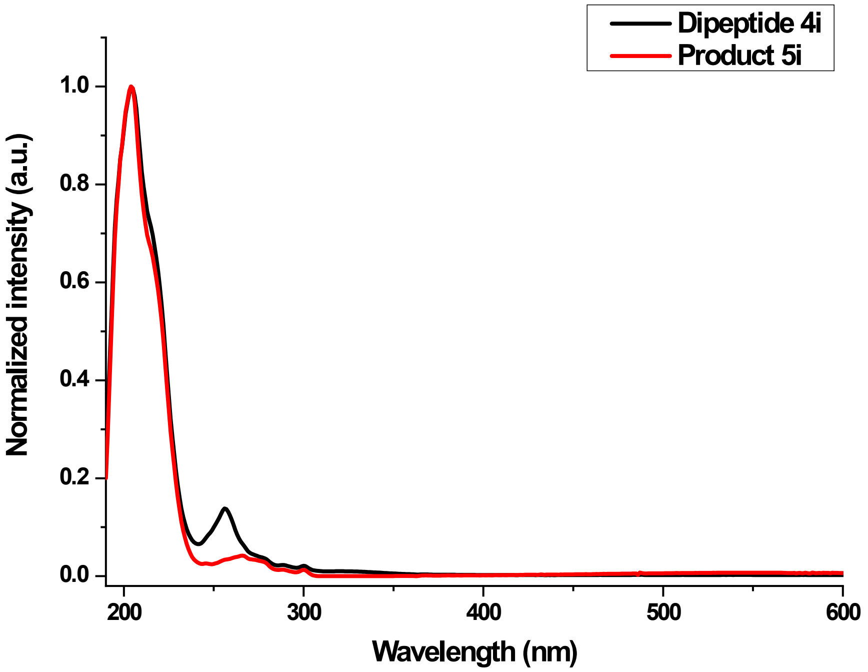
Dipeptide 4i
Product 5i
Normalized intensity (a.u.)
1.0
0.8
0.6
0.4
0.2
0.0
200
300
400
500
600
Wavelength (nm)

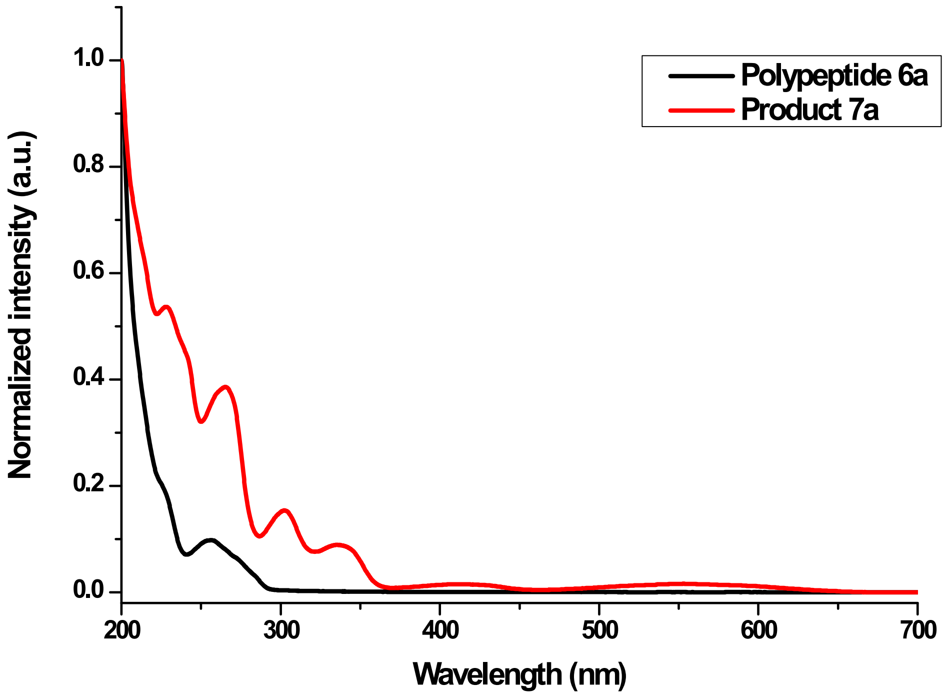
Polypeptide 6a
Product 7a
Normalized intensity (a.u.)
1.0
0.8
0.6
0.4
0.2
0.0
200
300
400
500
600
700
Wavelength (nm)

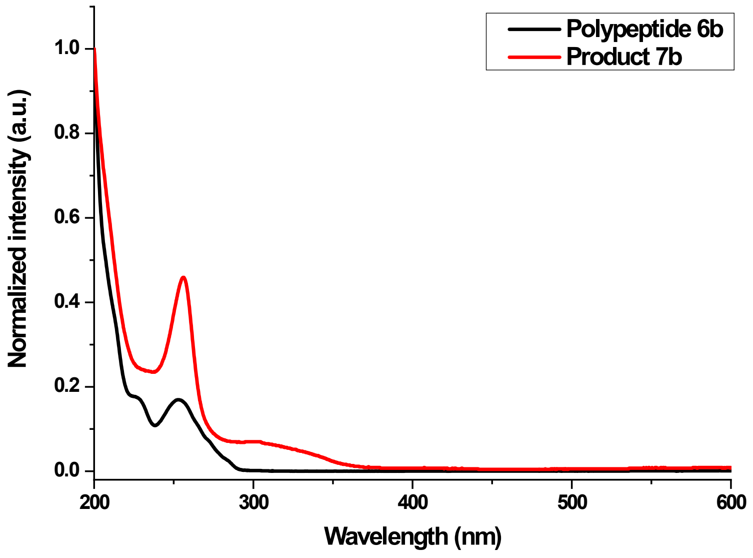
Polypeptide 6b
Product 7b
Normalized intensity (a.u.)
1.0
0.8
0.6
0.4
0.2
0.0
200
300
400
500
600
Wavelength (nm)

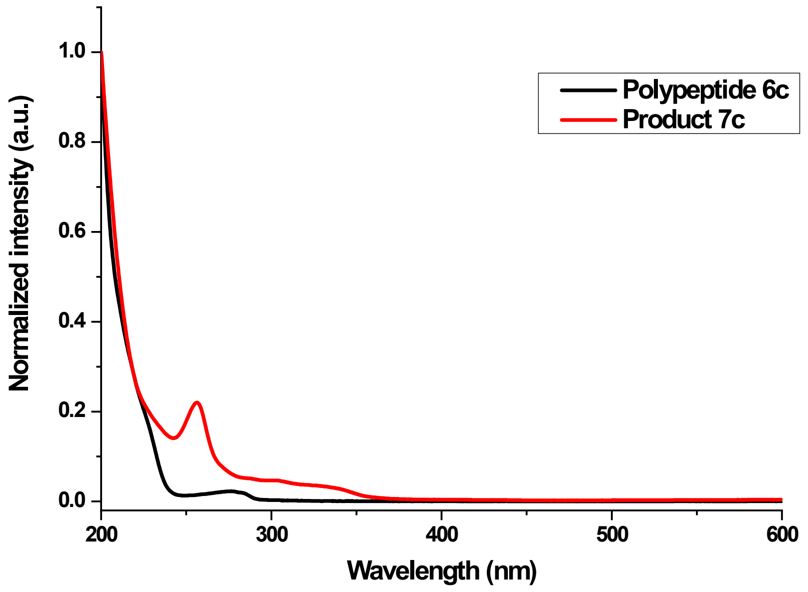
Polypeptide 6c
Product 7c
Normalized intensity (a.u.)
1.0
0.8
0.6
0.4
0.2
0.0
200
300
400
500
600
Wavelength (nm)

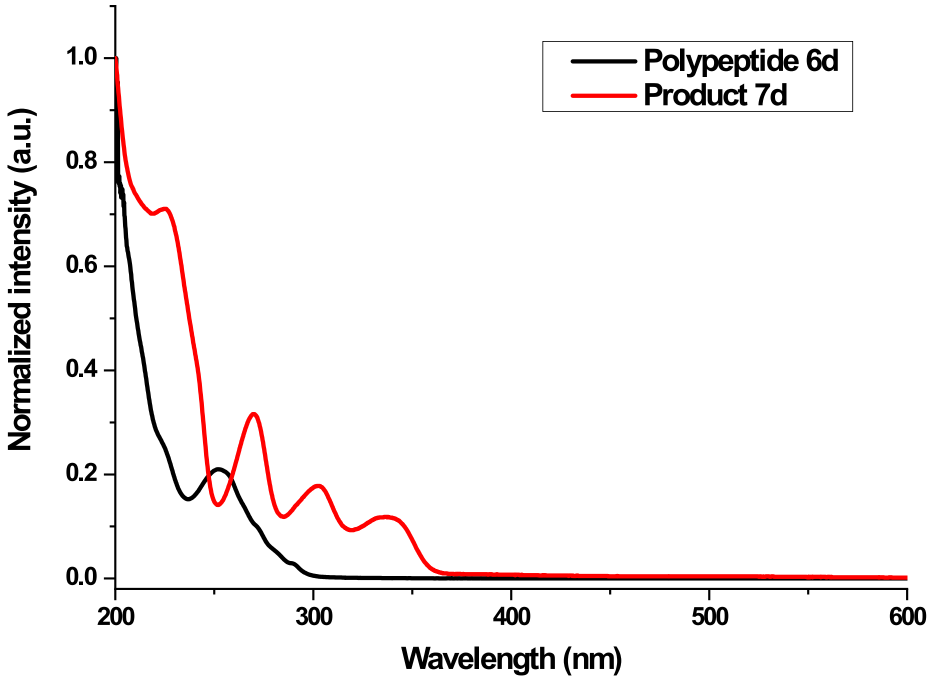
Polypeptide 6d
Product 7d
Normalized intensity (a.u.)
1.0
0.8
0.6
0.4
0.2
0.0
200
300
400
500
600
Wavelength (nm)

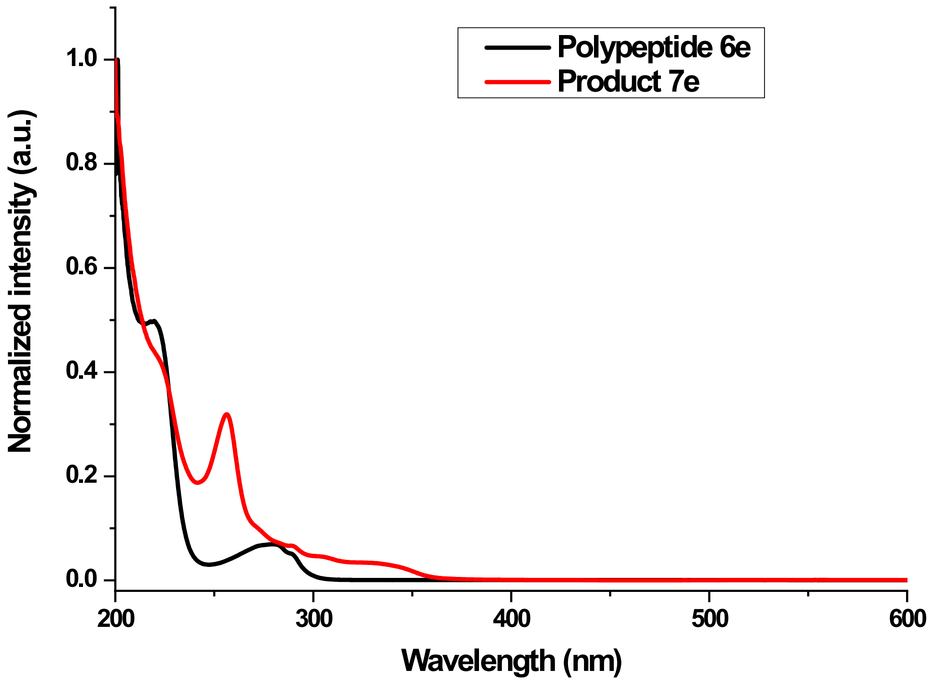
Polypeptide 6e
Product 7e
Normalized intensity (a.u.)
1.0
0.8
0.6
0.4
0.2
0.0
200
300
400
500
600
Wavelength (nm)

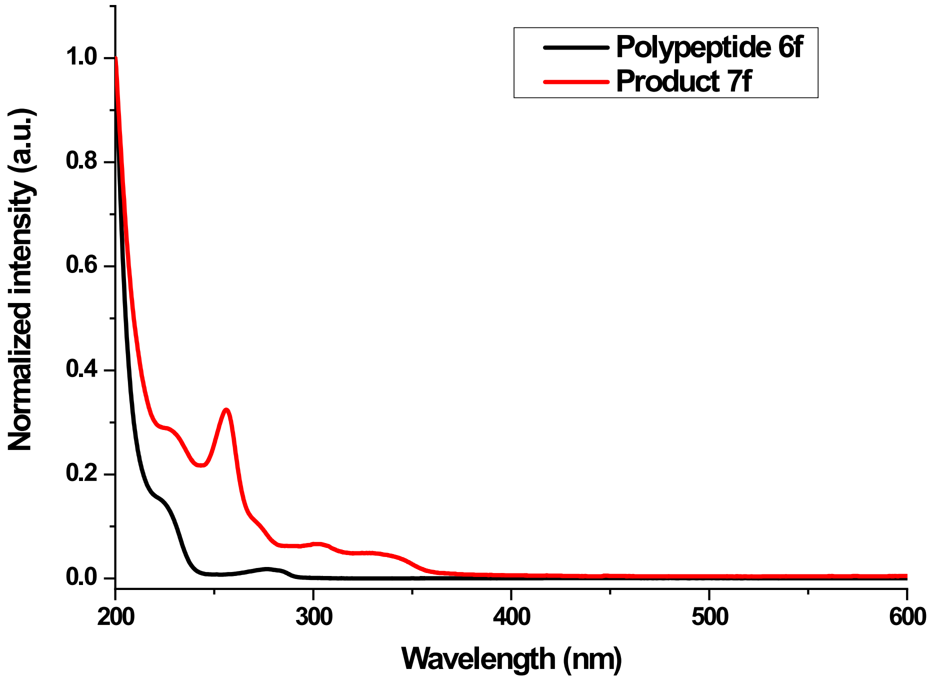
Polypeptide 6f
Product 7f
Normalized intensity (a.u.)
Wavelength (nm)
0.0
0.2
0.4
0.6
0.8
1.0
200
300
400
500
600

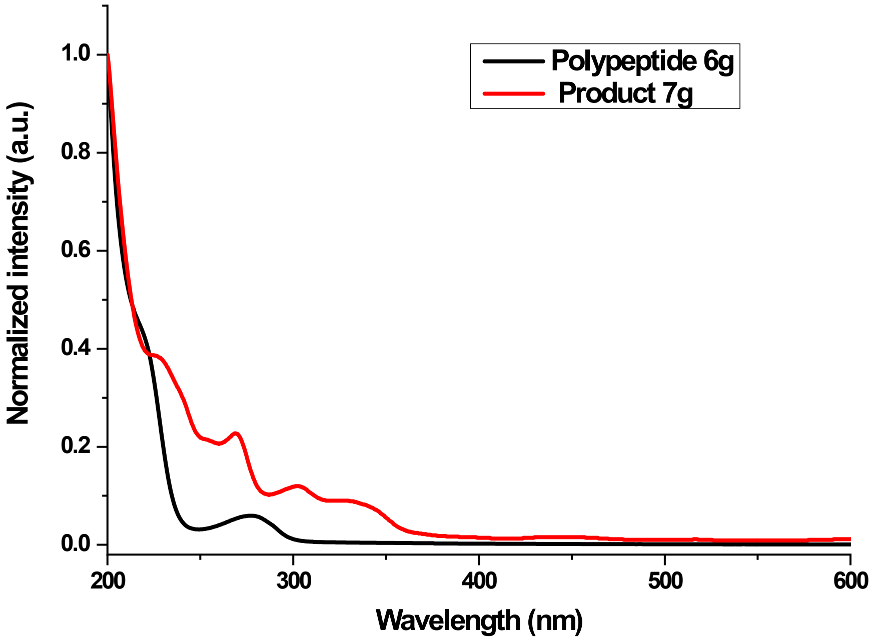
Polypeptide 6g
Product 7g
Normalized intensity (a.u.)
Wavelength (nm)
0.0
0.2
0.4
0.6
0.8
1.0
200
300
400
500
600

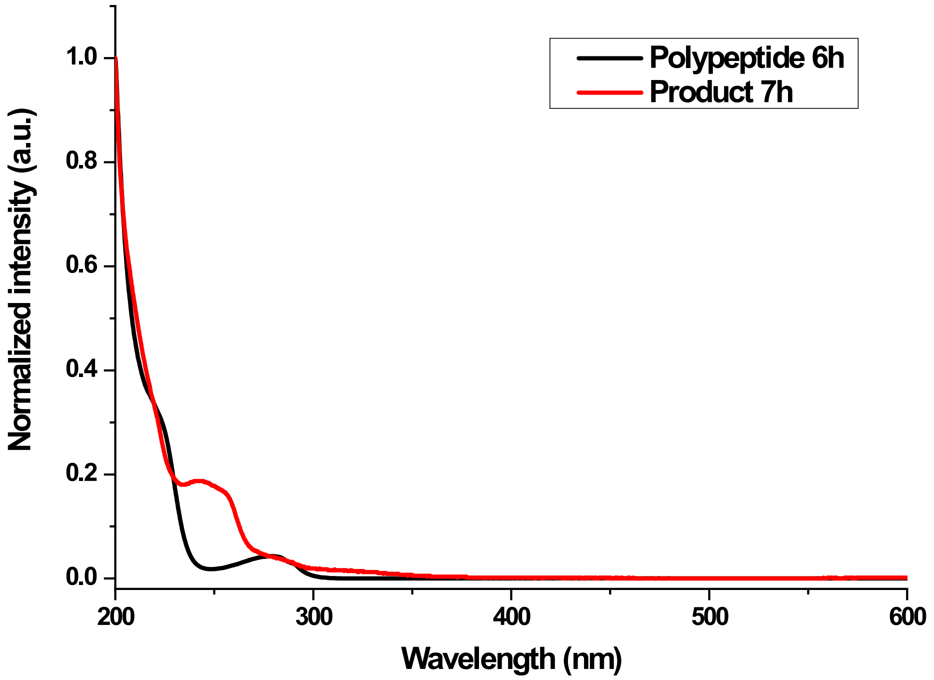
Polypeptide 6h
Product 7h
Normalized intensity (a.u.)
Wavelength (nm)
0.0
0.2
0.4
0.6
0.8
1.0
200
300
400
500
600

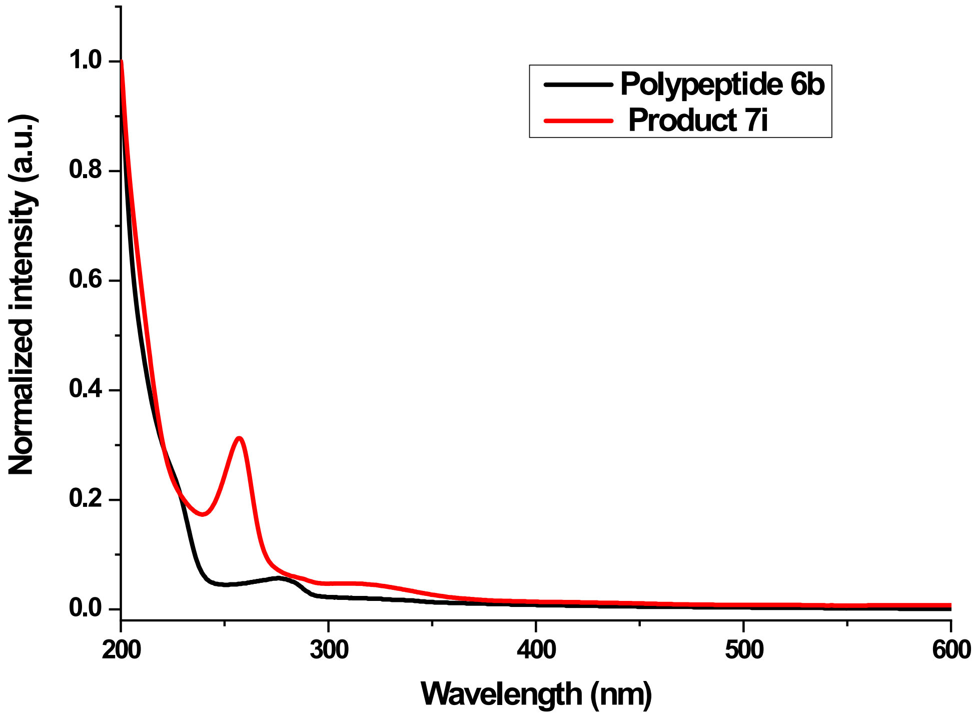
Polypeptide 6b
Product 7i
Normalized intensity (a.u.)
Wavelength (nm)
0.0
0.2
0.4
0.6
0.8
1.0
200
300
400
500
600

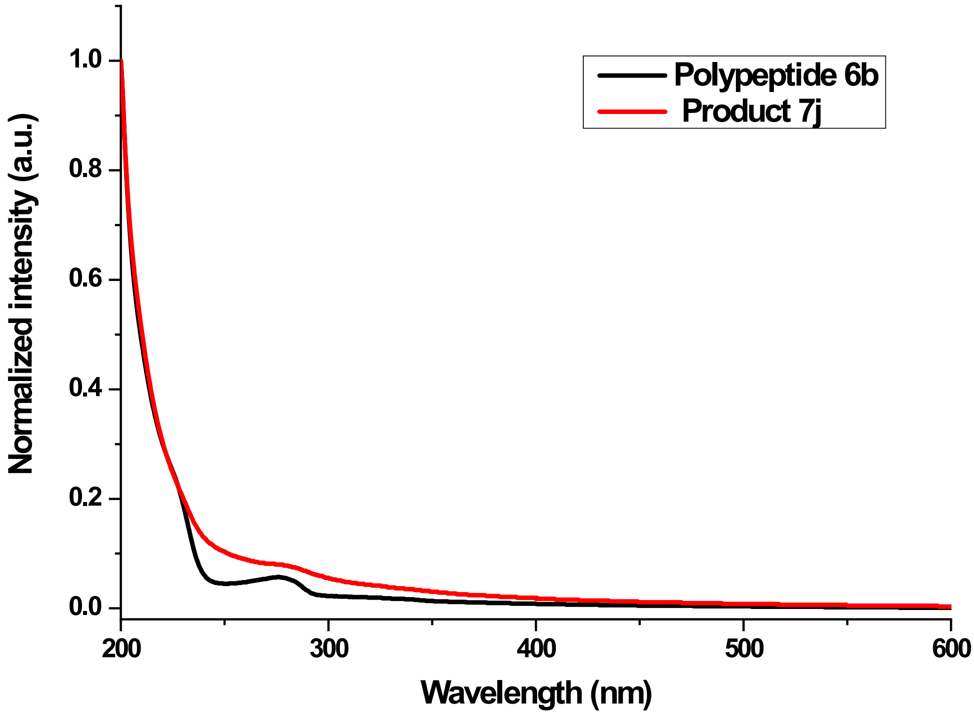

Polypeptide 6b
Product 7j
Normalized intensity (a.u.)
Wavelength (nm)
0.0
0.2
0.4
0.6
0.8
1.0
200
300
400
500
600

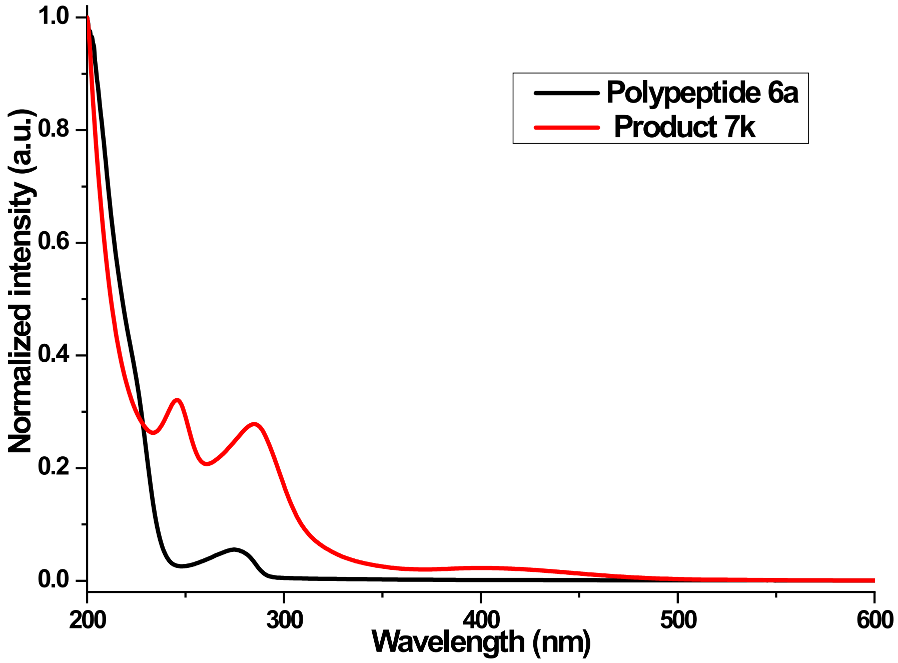

Polypeptide 6a
Product 7k
Normalized intensity (a.u.)
Wavelength (nm)
0.0
0.2
0.4
0.6
0.8
1.0
200
300
400
500
600

5.3 Fluorescence Measurement of Reactants and Products

Normalized emission (excited at 260 nm) and excitation (observed at 340 nm) spectra of **4a** and **5a**

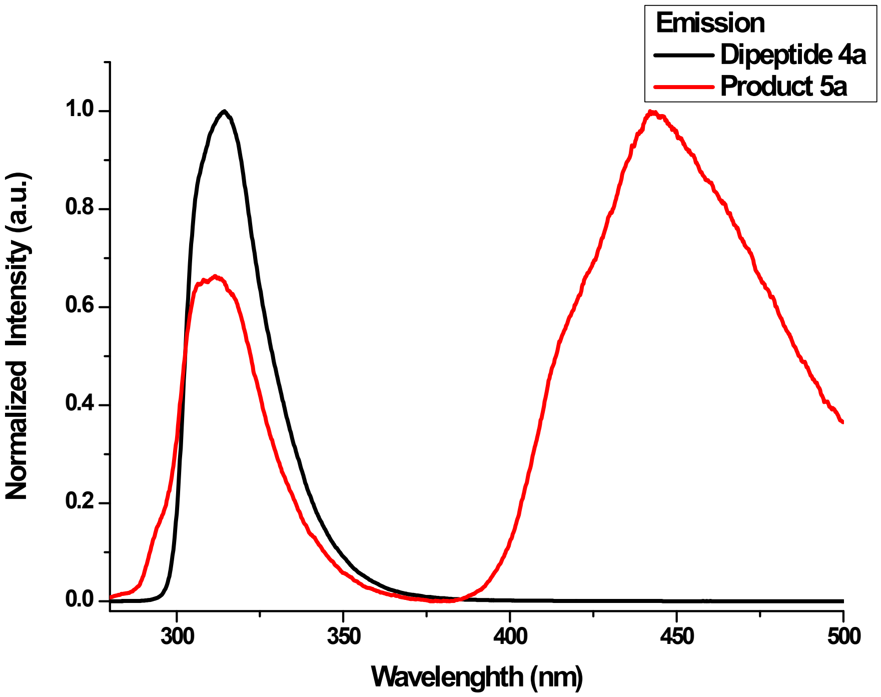

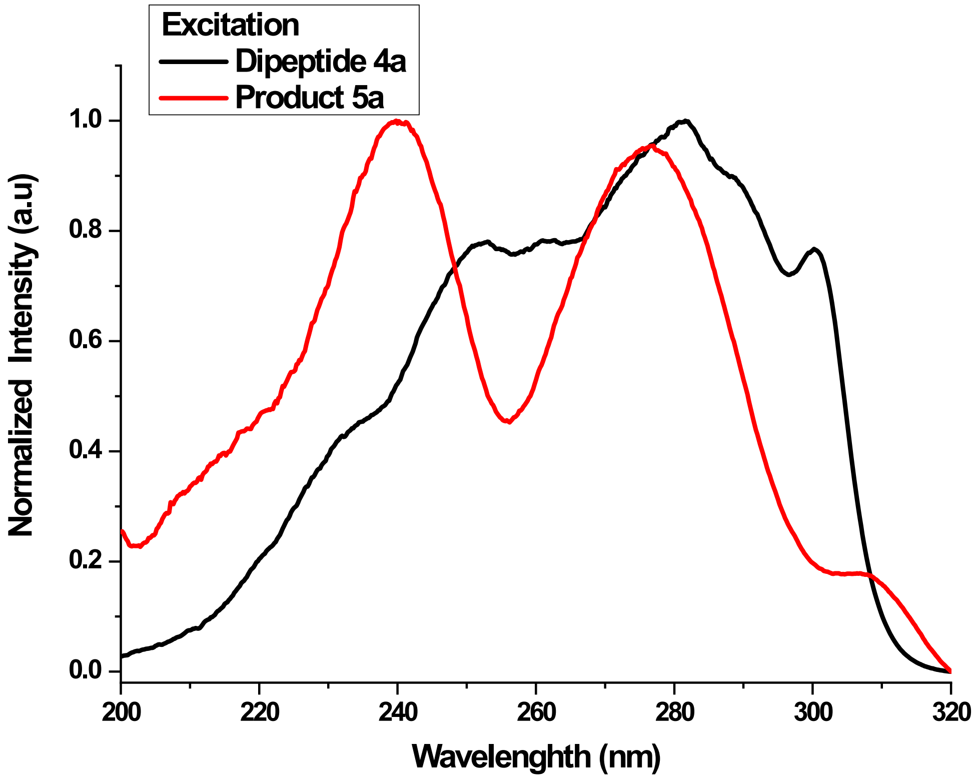

Normalized emission (excited at 270 nm) and excitation (observed at 340 nm) spectra of **4b** and **5b**

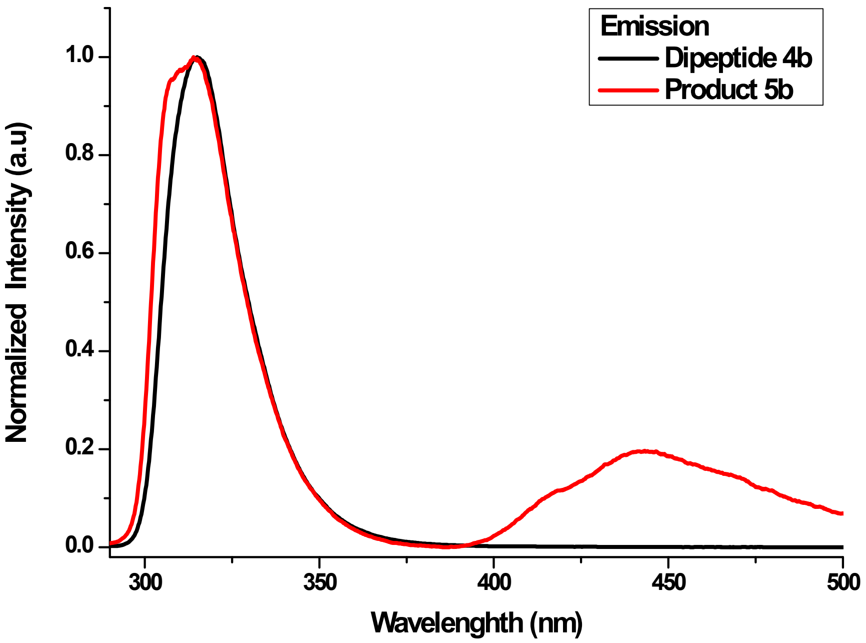

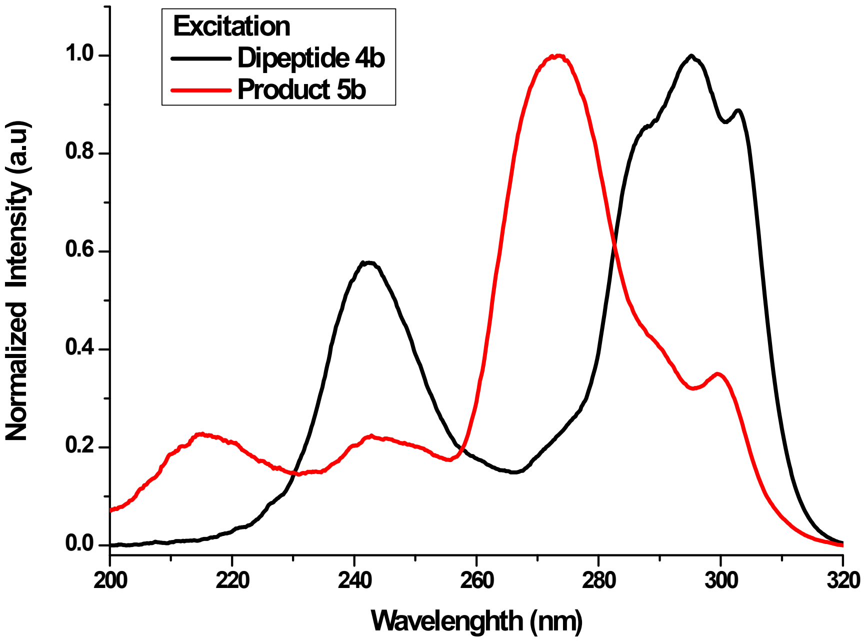

Normalized emission (excited at 270 nm) and excitation (observed at 340 nm) spectra of **4c** and **5c**

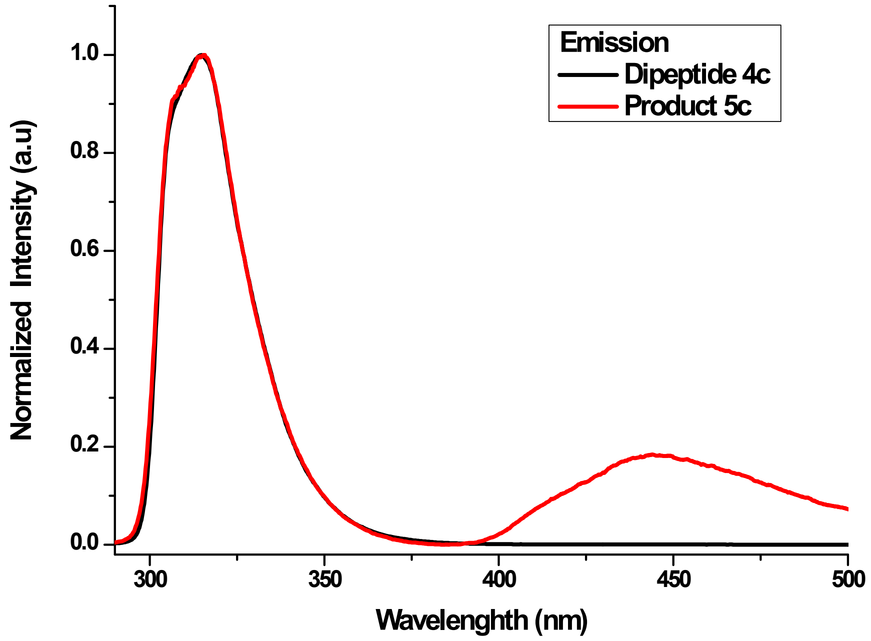

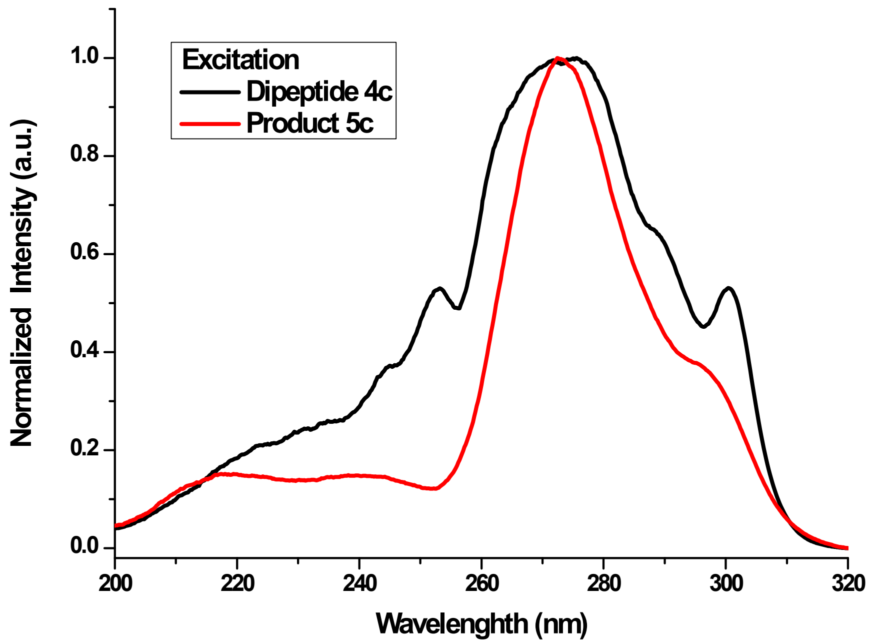

Normalized emission (excited at 260 nm) and excitation (observed at 340 nm) spectra of **4d** and **5d**

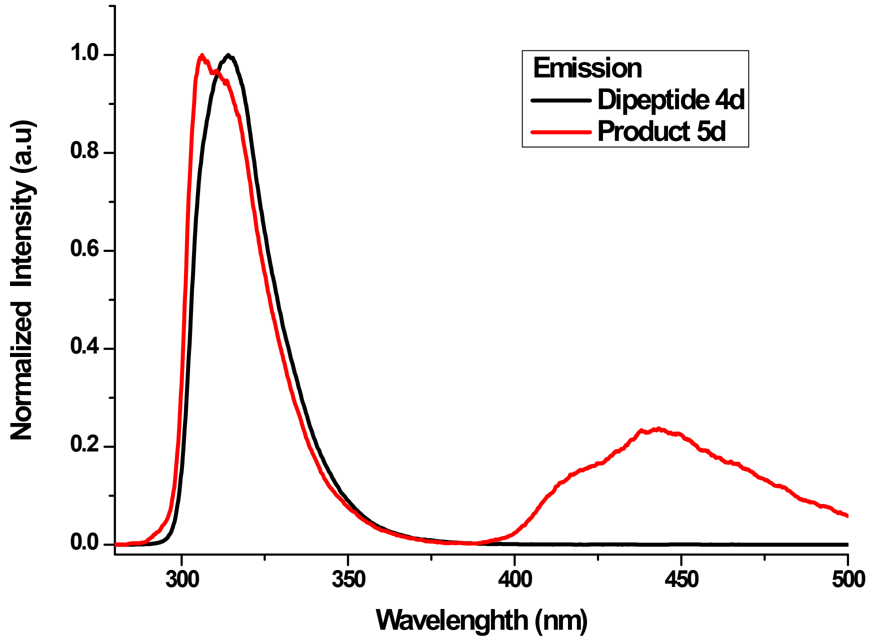

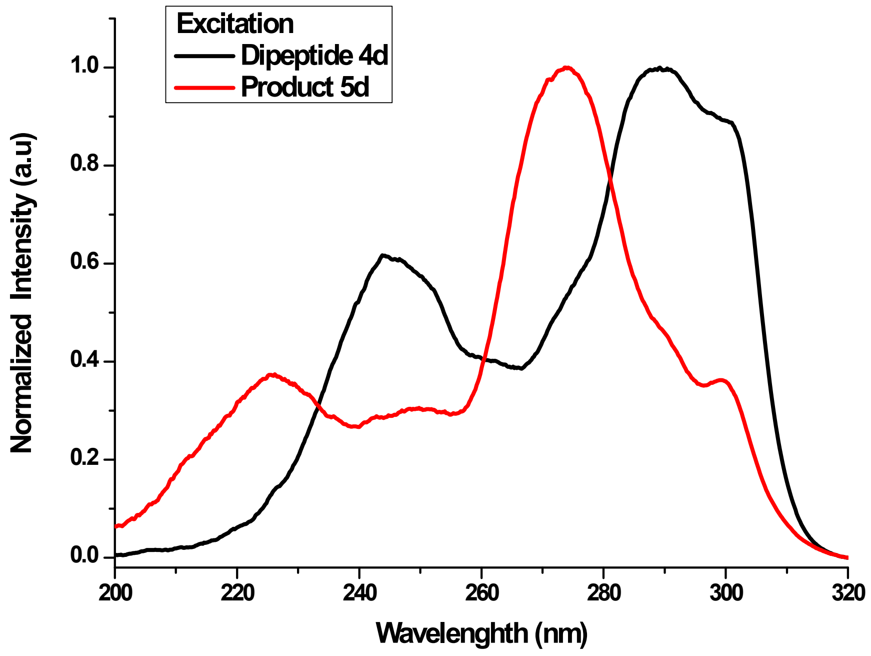

Normalized emission (excited at 260 nm) and excitation (observed at 340 nm) spectra of **4e** and **5e**

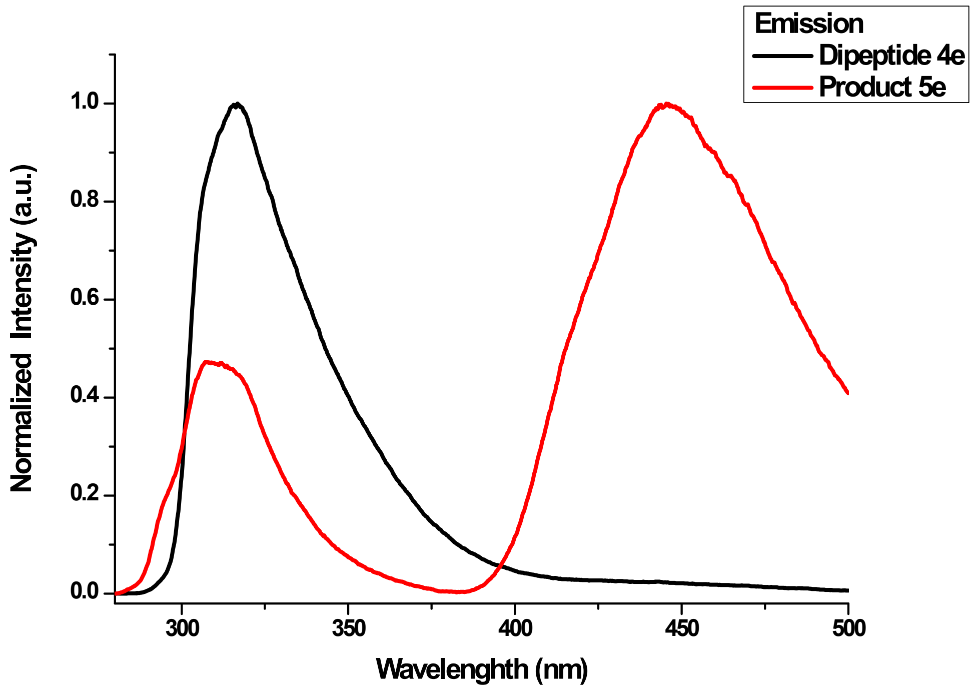

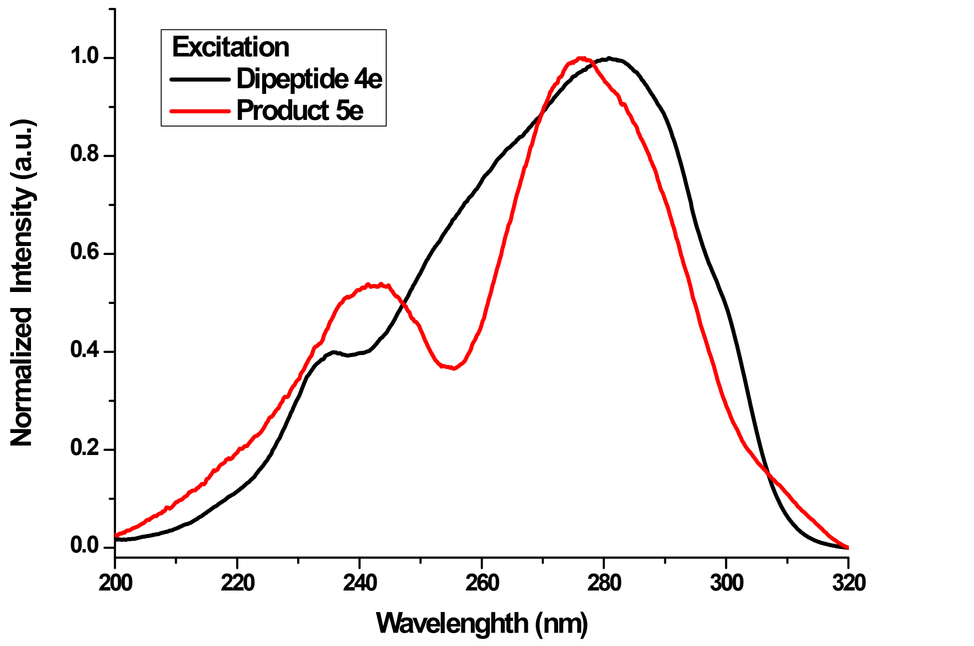

Normalized emission (excited at 260 nm) and excitation (observed at 340 nm) spectra of **4f** and **5f**

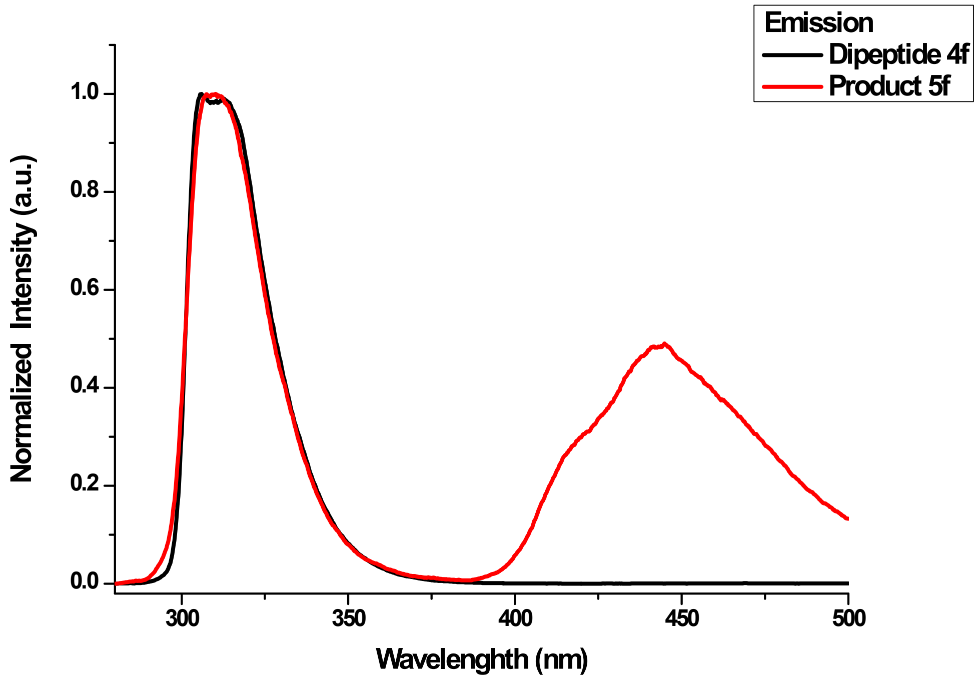

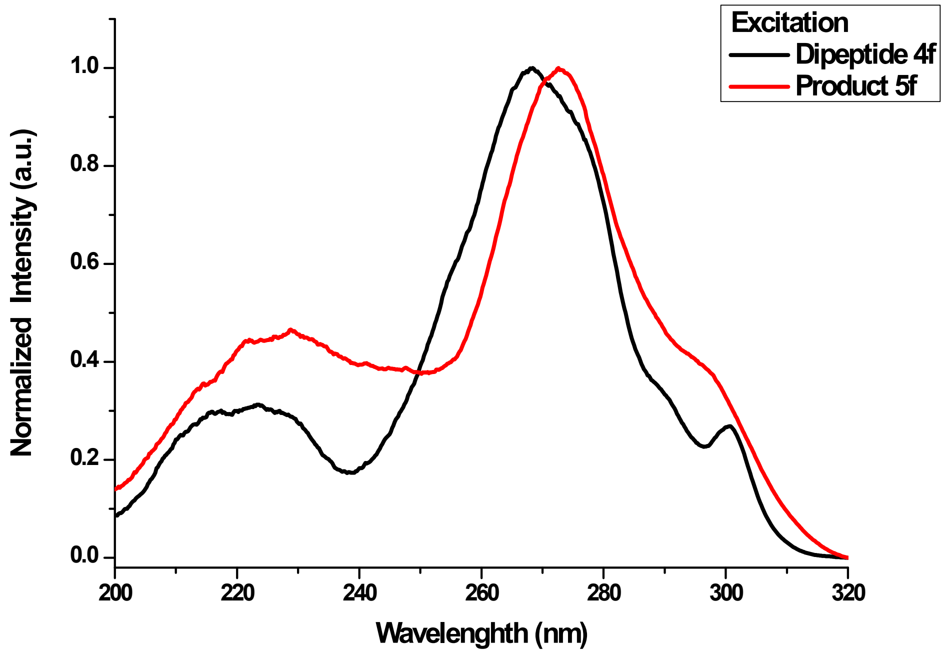

Normalized emission (excited at 260 nm) and excitation (observed at 340 nm) spectra of **4g** and **5g**

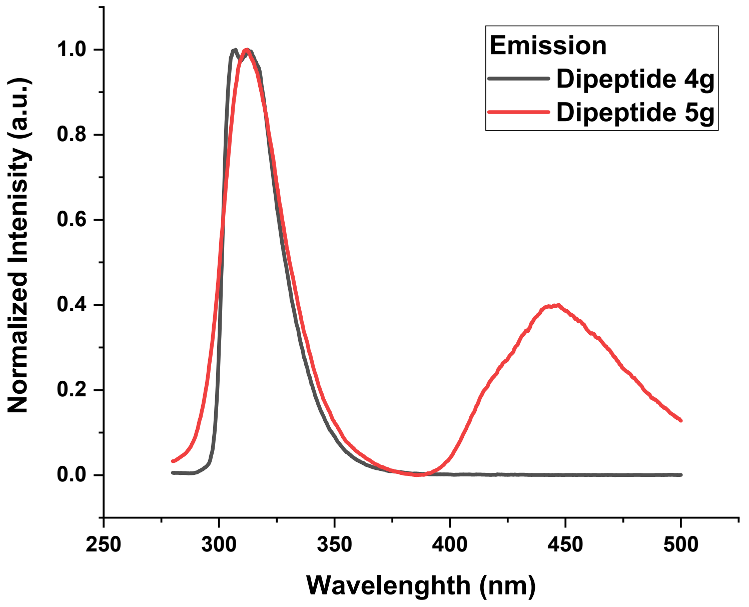

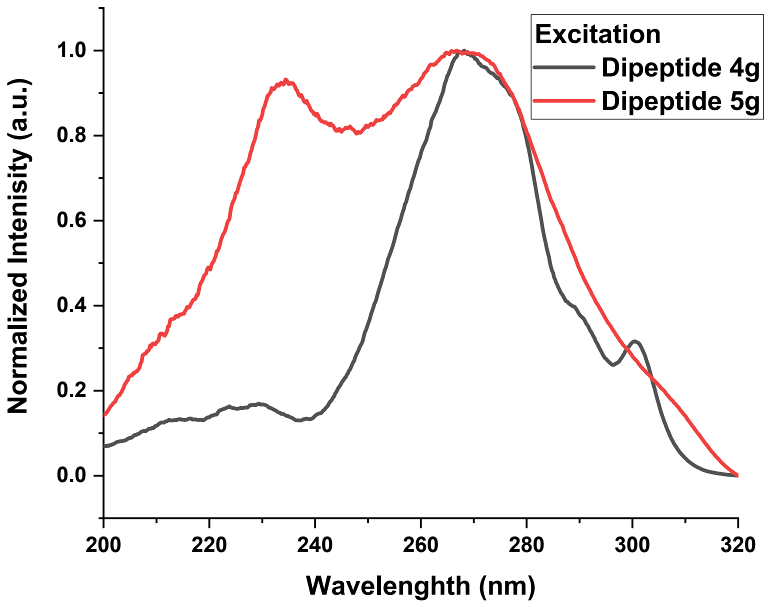

Normalized emission (excited at 270 nm) and excitation (observed at 340 nm) spectra of **4h** and **5h**

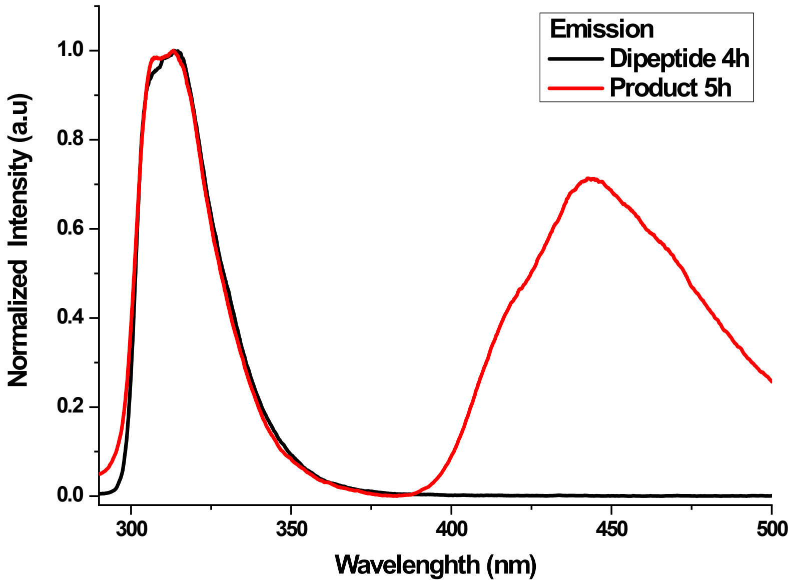

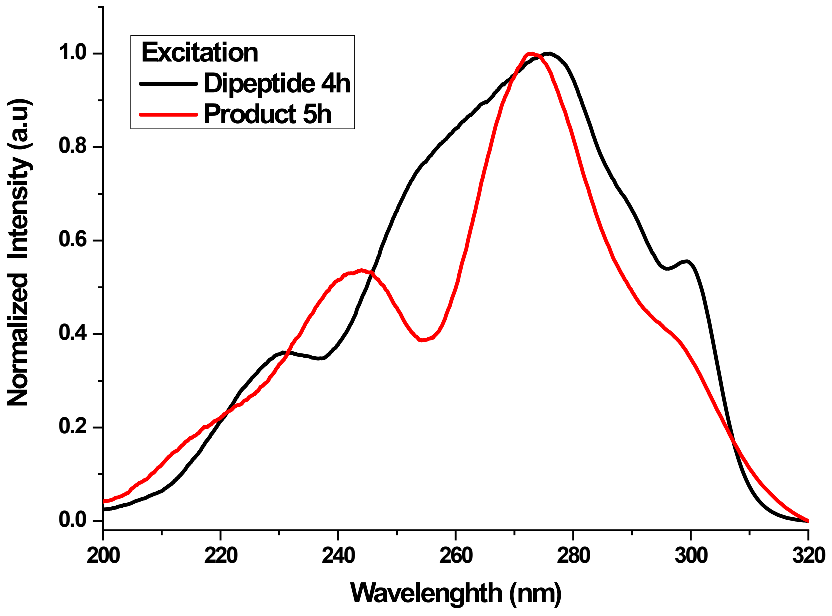

Normalized emission (excited at 270 nm) and excitation (observed at 340 nm) spectra of **4i** and **5i**

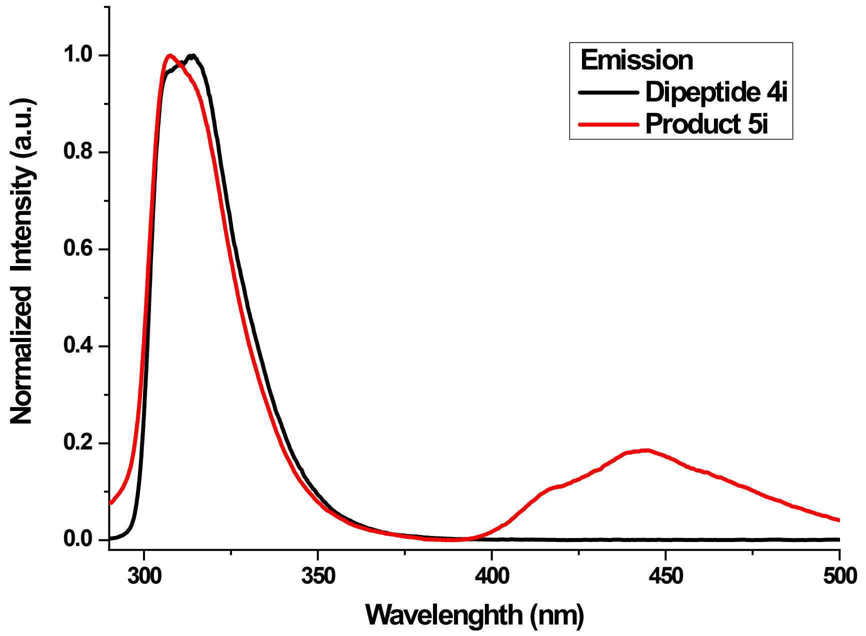

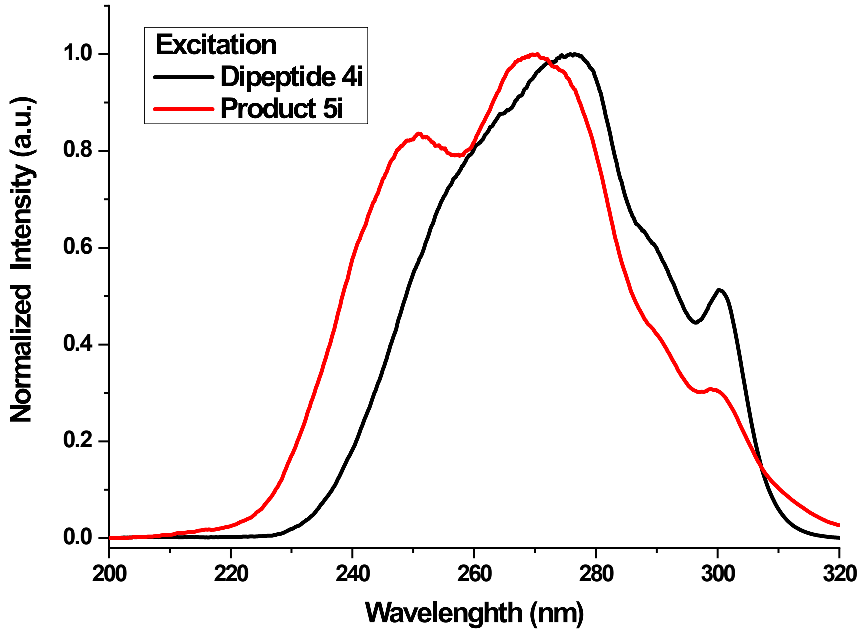

Normalized emission (excited at 320 nm) and excitation (observed at 450 nm) spectra of **6a** and **7a**

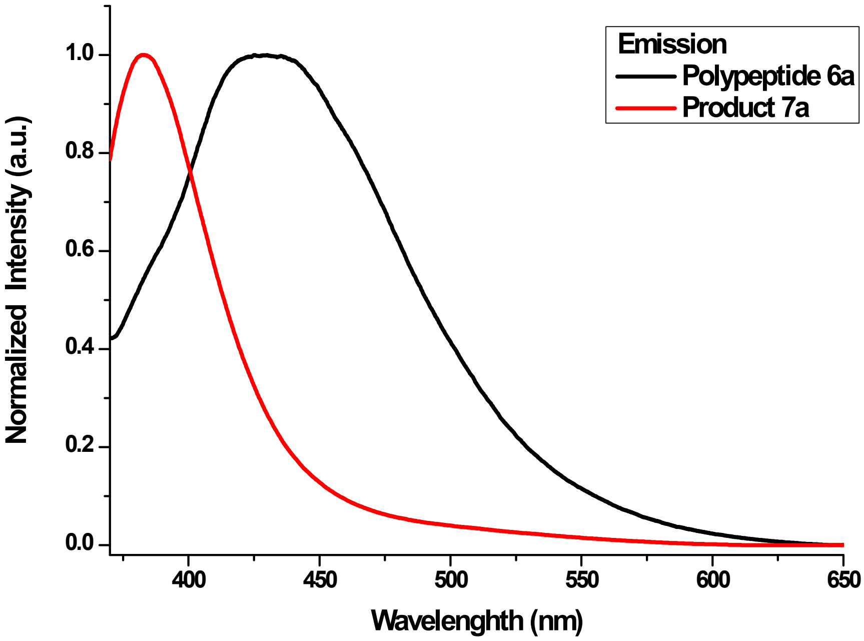

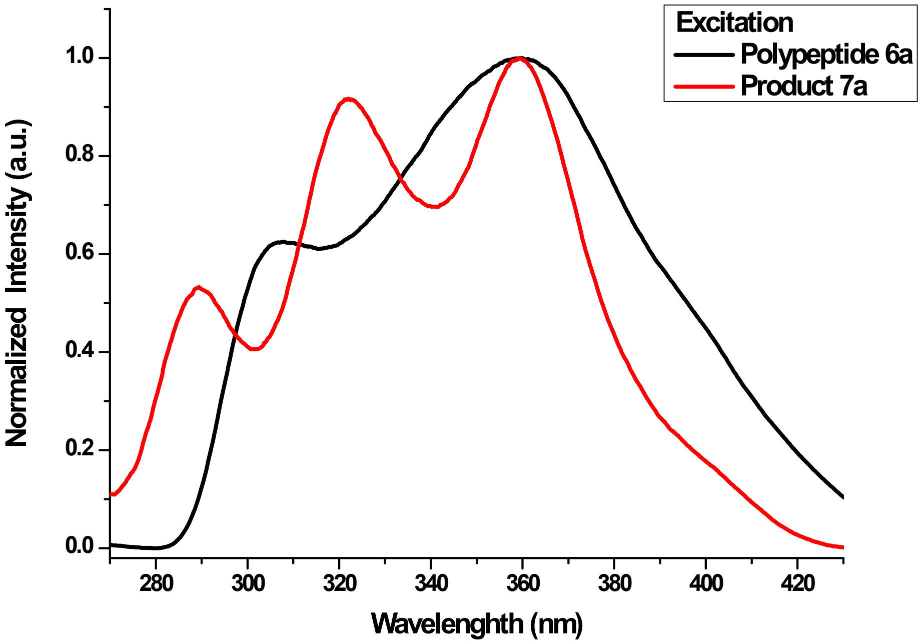

Normalized emission (excited at 320 nm) and excitation (observed at 450 nm) spectra of **6b** and **7b**

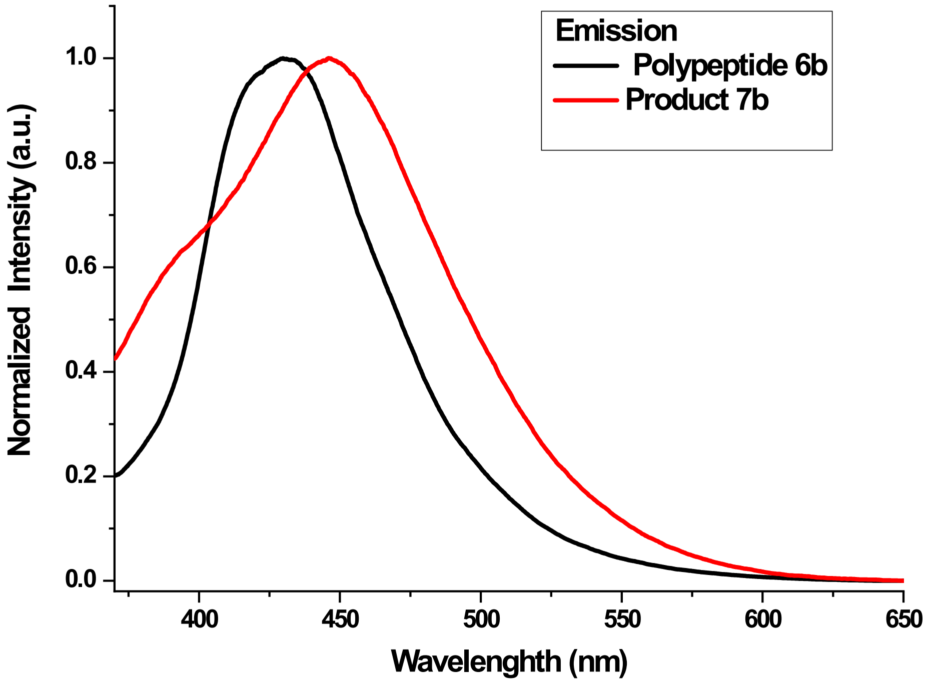

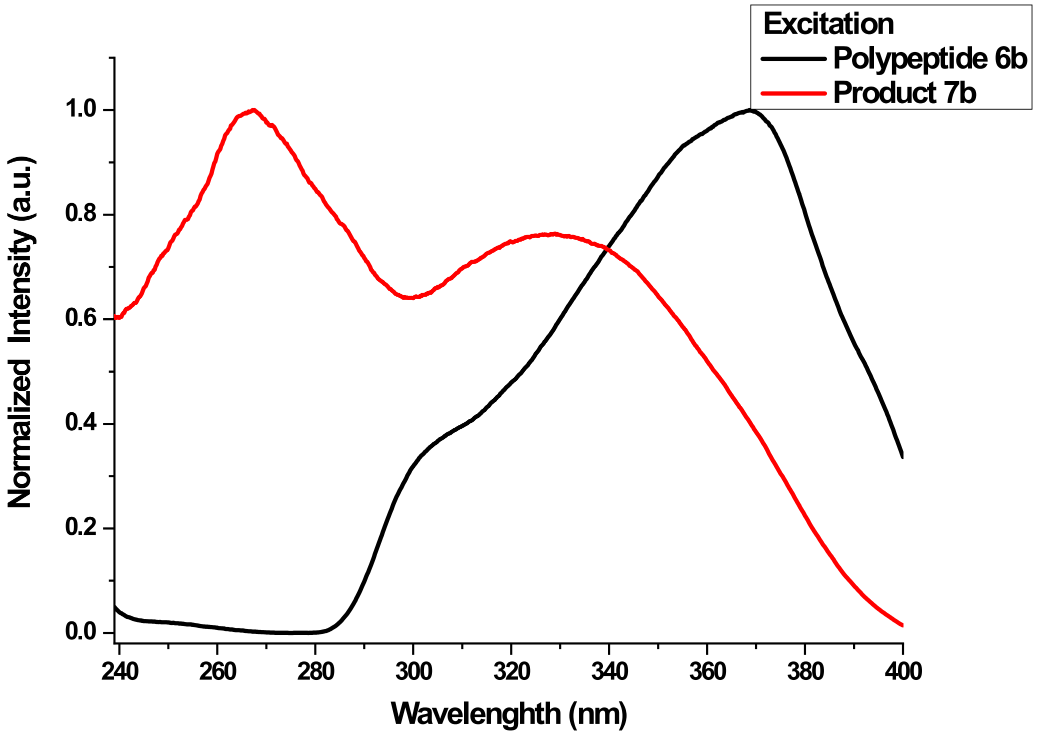

Normalized emission (excited at 320 nm) and excitation (observed at 450 nm) spectra of **6c** and **7c**

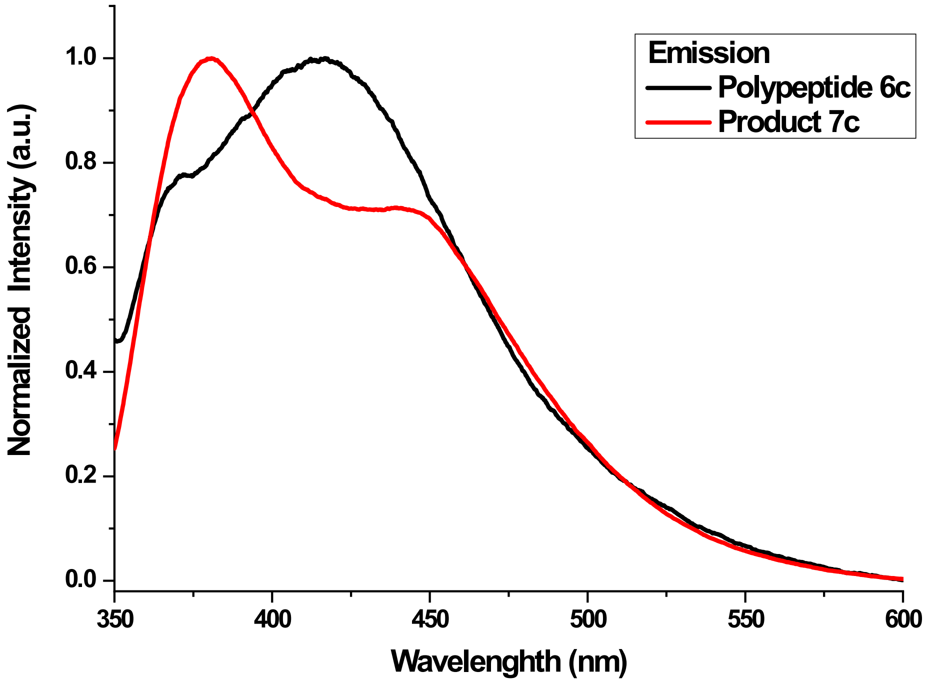

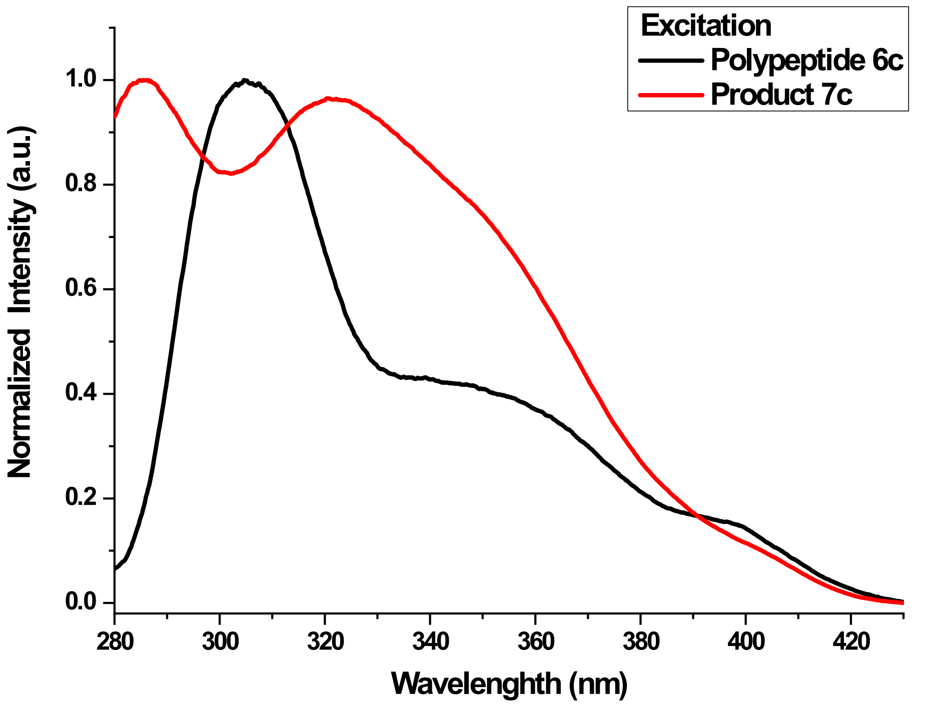

Normalized emission (excited at 320 nm) and excitation (observed at 450 nm) spectra of **6d** and **7d**

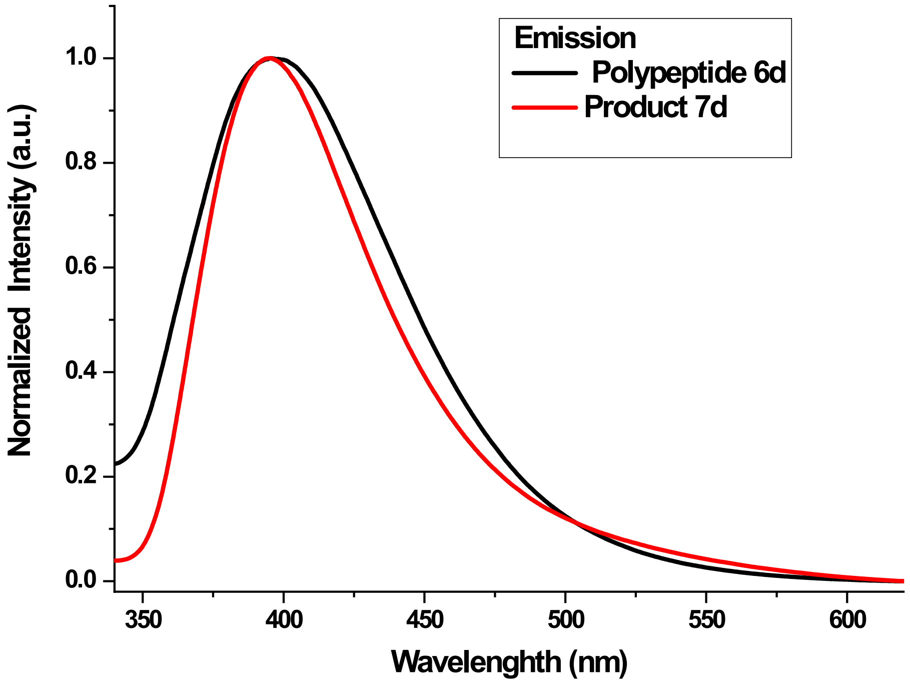

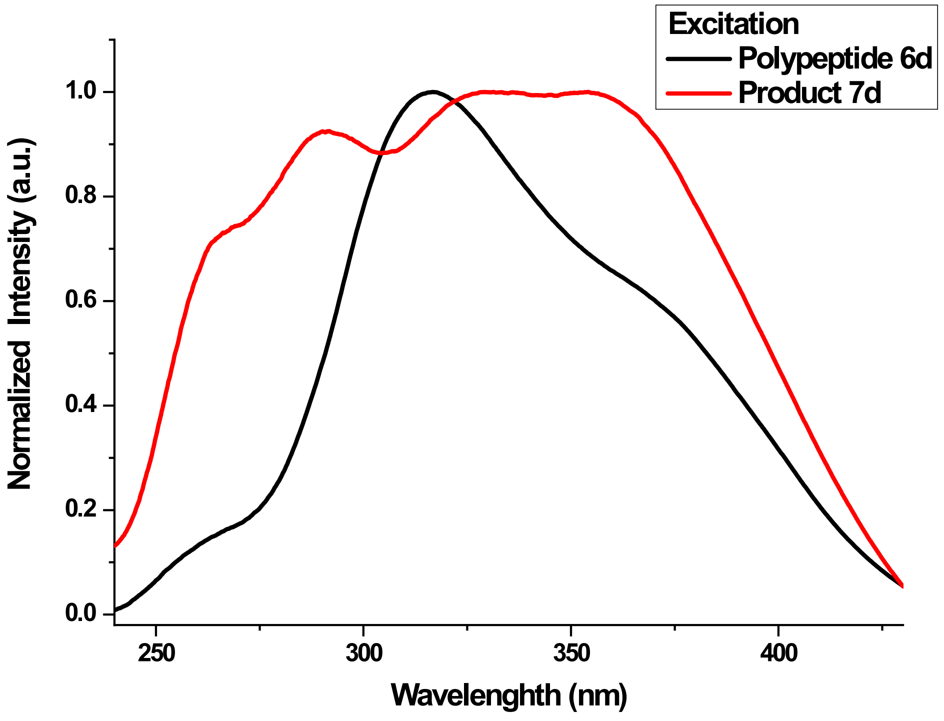

Normalized emission (excited at 320 nm) and excitation (observed at 450 nm) spectra of **6e** and **7e**

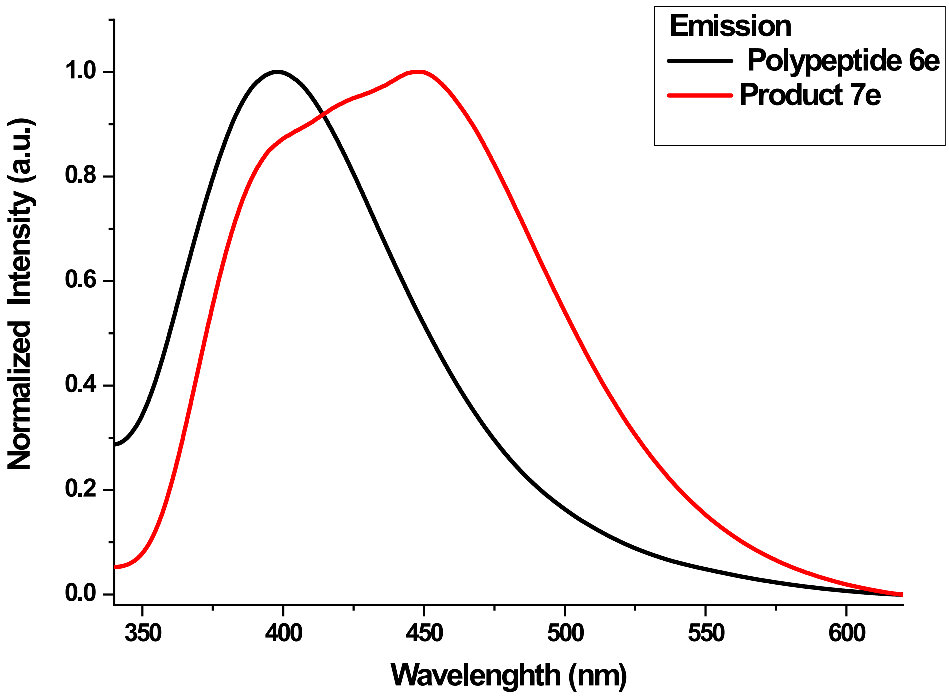

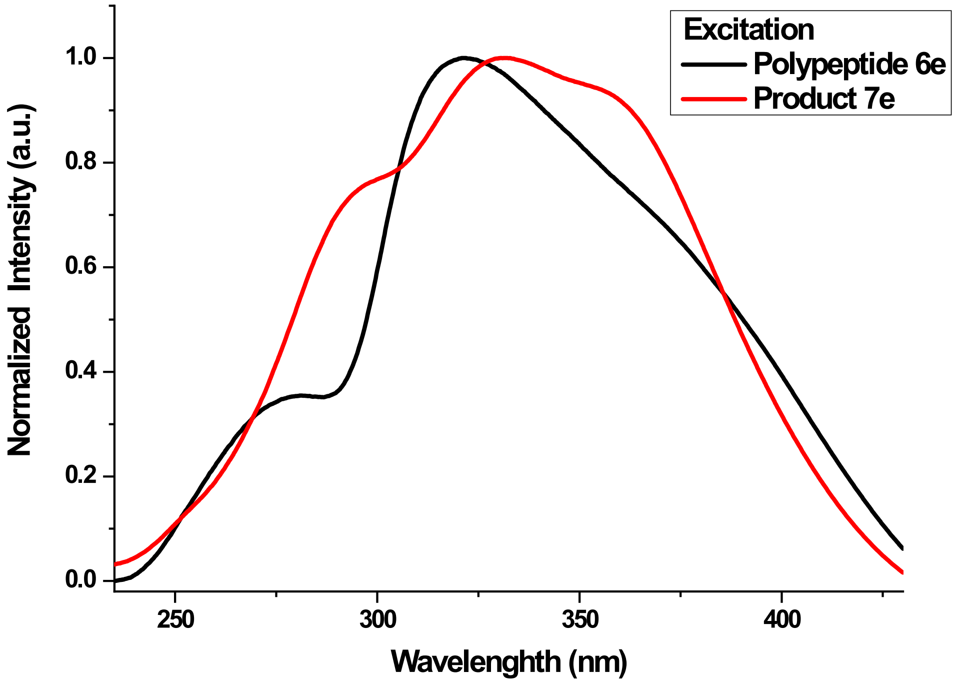

Normalized emission (excited at 320 nm) and excitation (observed at 450 nm) spectra of **6f** and **7f**

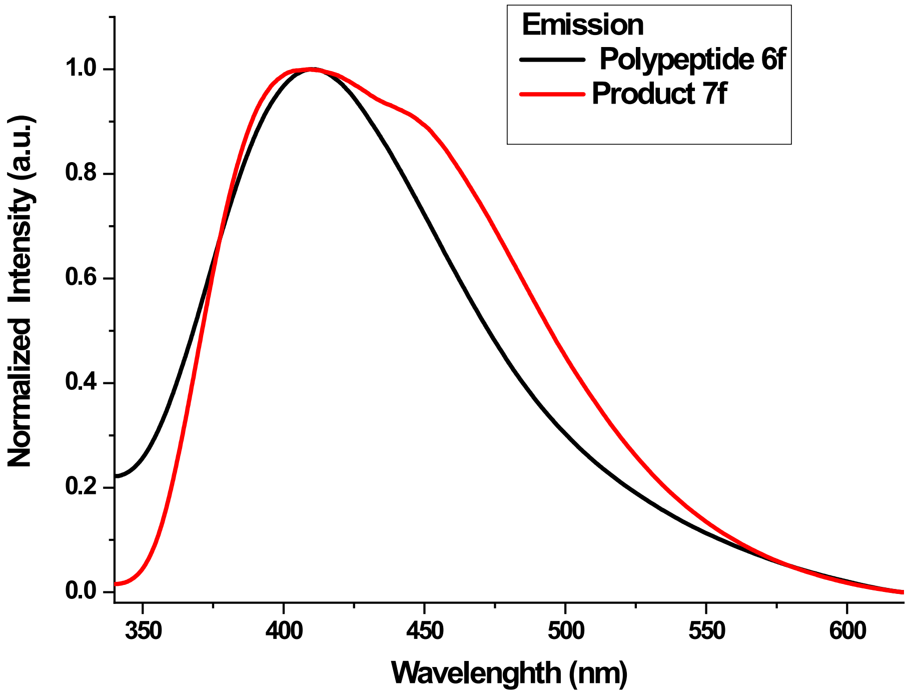

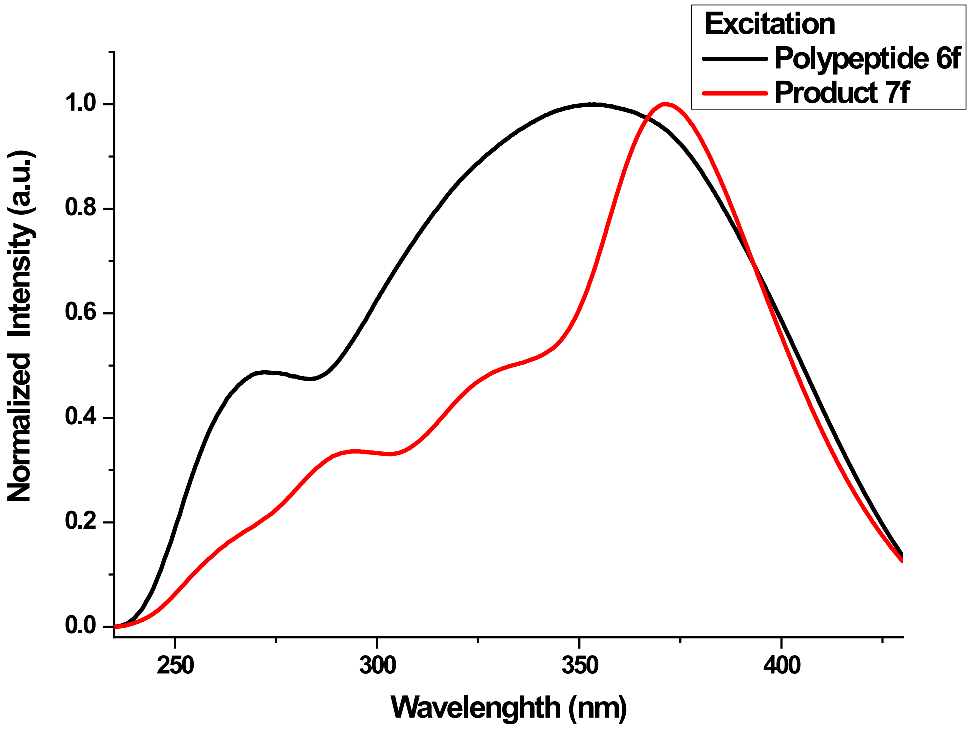

Normalized emission (excited at 320 nm) and excitation (observed at 450 nm) spectra of **6g** and **7g**

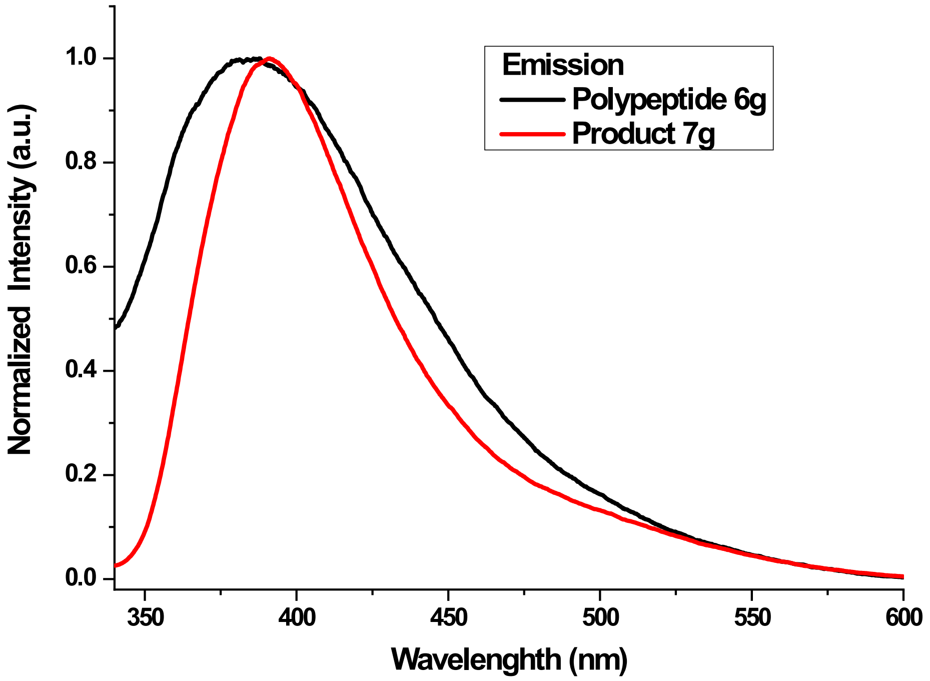

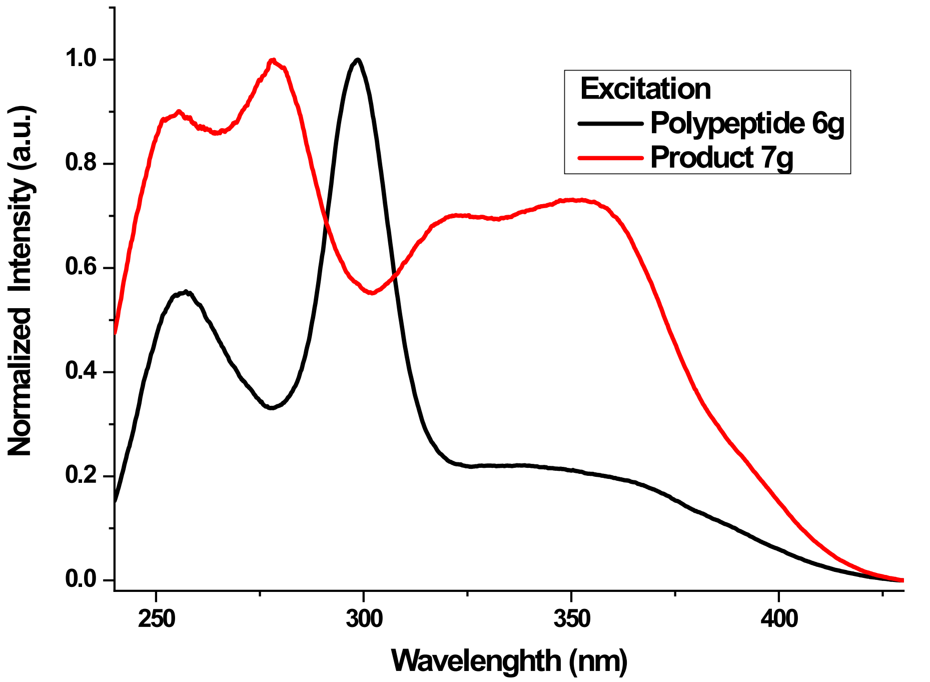

Normalized emission (excited at 320 nm) and excitation (observed at 450 nm) spectra of **6h** and **7h**

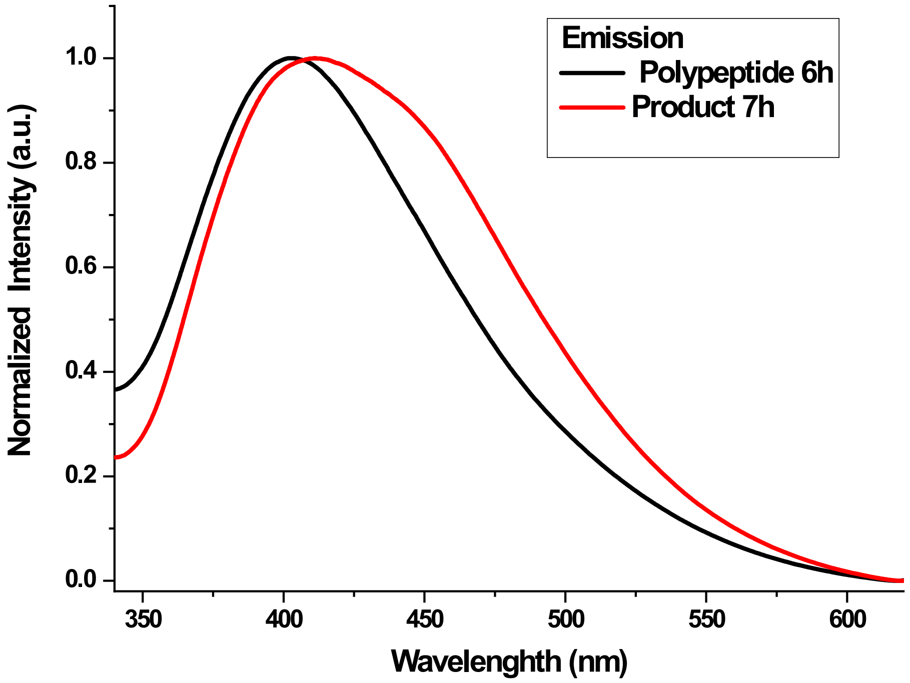

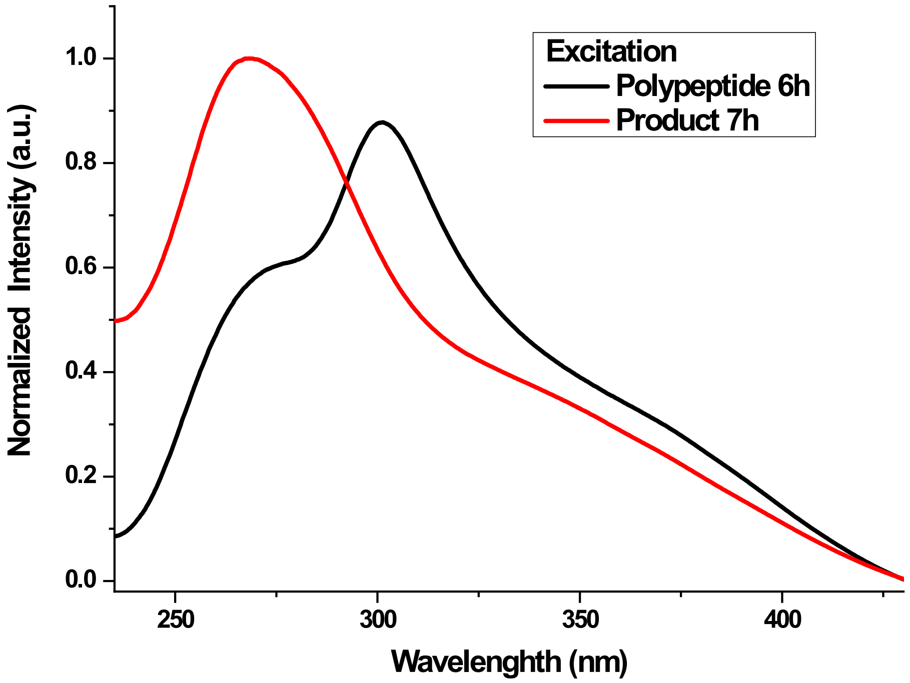

Normalized emission (excited at 320 nm) and excitation (observed at 450 nm) spectra of **6b** and **7i**

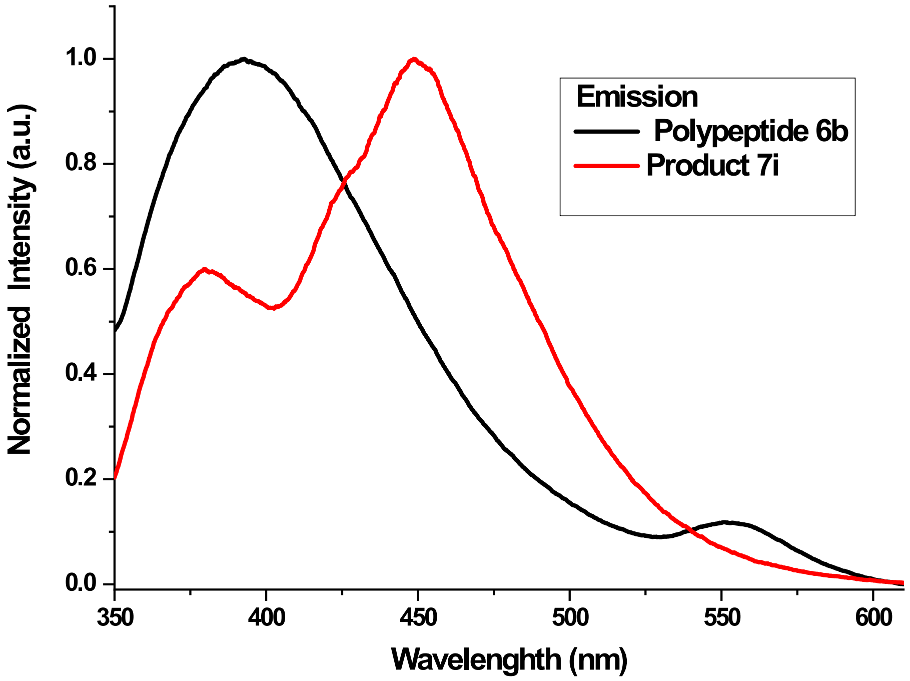

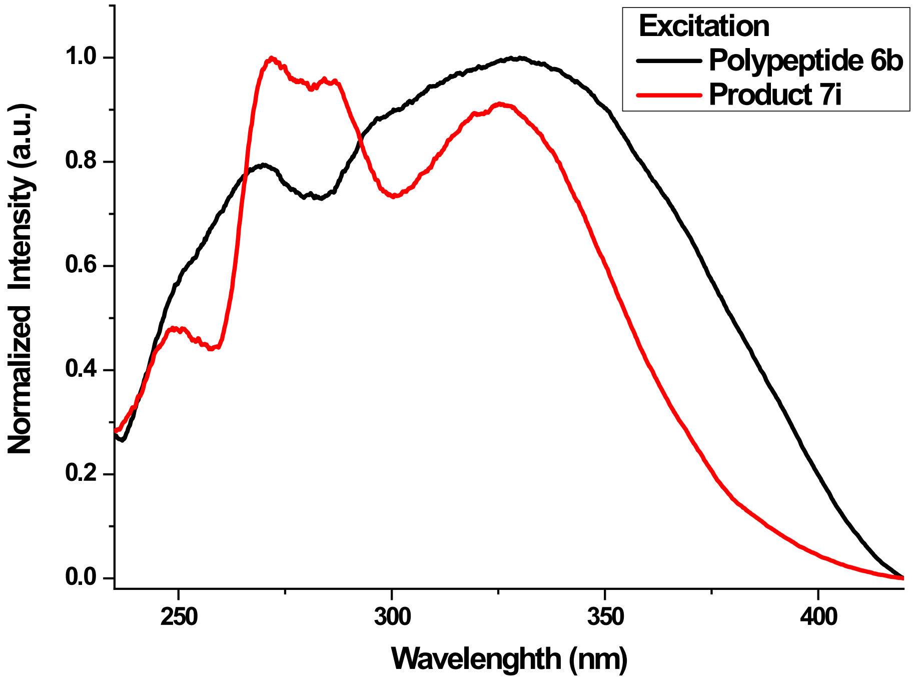

Normalized emission (excited at 320 nm) and excitation (observed at 450 nm) spectra of **6b** and **7j**

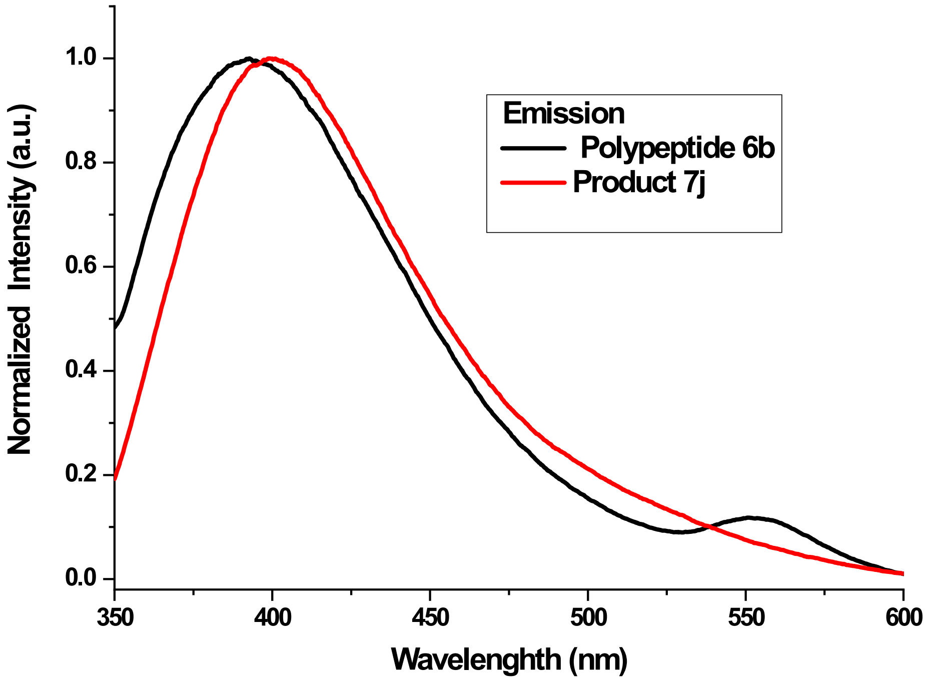

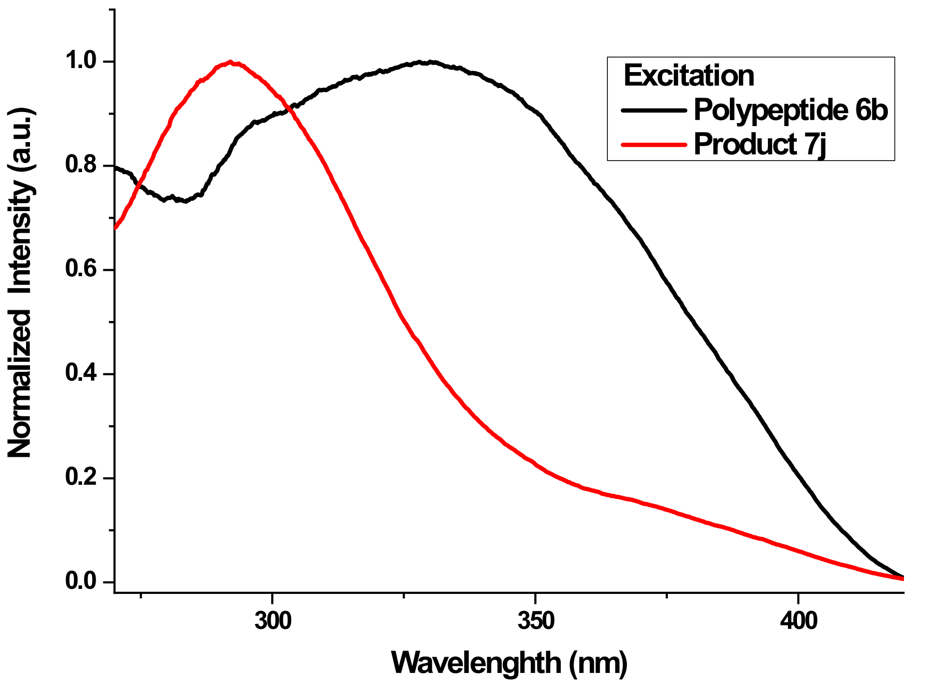

Normalized emission (excited at 360 nm) and excitation (observed at 450 nm) spectra of **6a** and **7k**

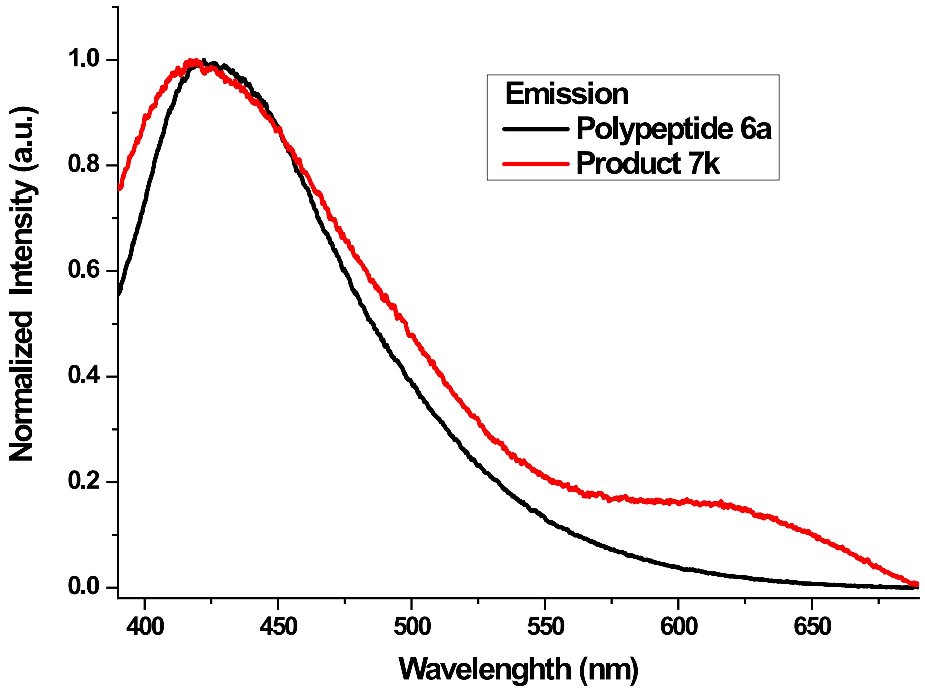

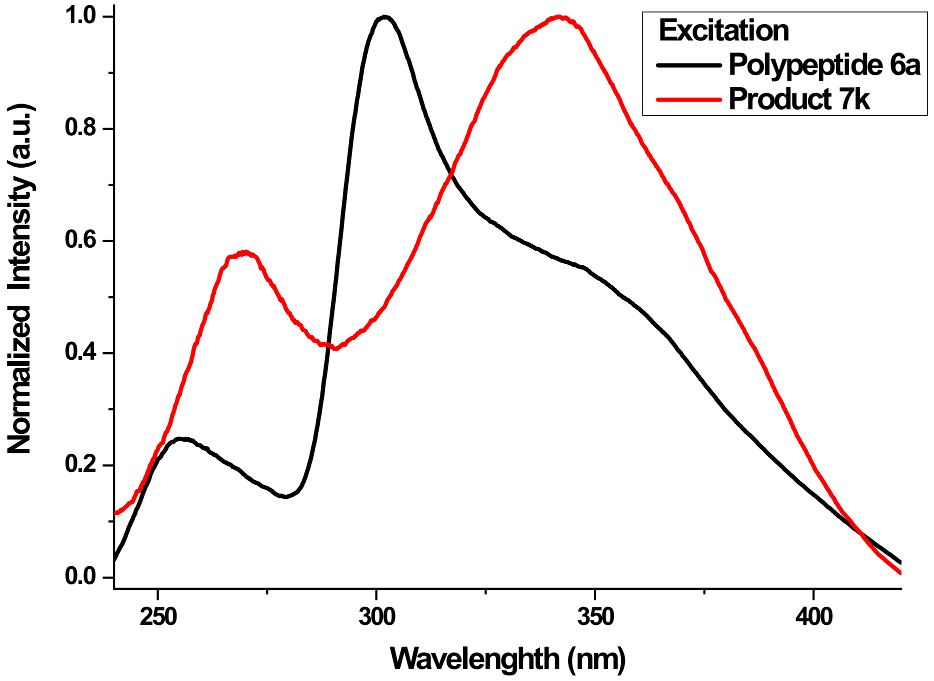

Supplement: Supplementary file 1 [file SC-010-C9SC02218J-s001.pdf]
